# Genetic basis of lacunar stroke: a pooled analysis of individual patient data and genome-wide association studies

**DOI:** 10.1016/S1474-4422(21)00031-4

**Published:** 2021-05

**Authors:** Matthew Traylor, Elodie Persyn, Liisa Tomppo, Sofia Klasson, Vida Abedi, Mark K Bakker, Nuria Torres, Linxin Li, Steven Bell, Loes Rutten-Jacobs, Daniel J Tozer, Christoph J Griessenauer, Yanfei Zhang, Annie Pedersen, Pankaj Sharma, Jordi Jimenez-Conde, Tatjana Rundek, Raji P Grewal, Arne Lindgren, James F Meschia, Veikko Salomaa, Aki Havulinna, Christina Kourkoulis, Katherine Crawford, Sandro Marini, Braxton D Mitchell, Steven J Kittner, Jonathan Rosand, Martin Dichgans, Christina Jern, Daniel Strbian, Israel Fernandez-Cadenas, Ramin Zand, Ynte Ruigrok, Natalia Rost, Robin Lemmens, Peter M Rothwell, Christopher D Anderson, Joanna Wardlaw, Cathryn M Lewis, Hugh S Markus, Helsinki Stroke, Study Dutch Parelsnoer Institute-Cerebrovascular Accident (CVA) Study Group, National Institute of Neurological Disorders and Stroke (NINDS) Stroke Genetics Network, UK DNA Lacunar Stroke Study Investigators, International Stroke Genetics Consortium

**Affiliations:** a Clinical Pharmacology and The Barts Heart Centre and NIHR Barts Biomedical Research Centre, Barts Health NHS Trust, William Harvey Research Institute, Queen Mary University of London, London, UK; b Department of Medical and Molecular Genetics, King's College London, London, UK; c Social, Genetic, and Developmental Psychiatry Centre, King's College London, London, UK; d Department of Neurology, Helsinki University Hospital, Helsinki, Finland; e Department of Laboratory Medicine, Institute of Biomedicine, Sahlgrenska Academy at University of Gothenburg, Gothenburg, Sweden; f Department of Molecular and Functional Genomics, Weis Center for Research, Geisinger Health System, Danville, PA, USA; g Neuroscience Institute, Geisinger Health System, Danville, PA, USA; h Genomic Medicine Institute, Geisinger Health System, Danville, PA, USA; i Department of Neurology and Neurosurgery, Brain Center Rudolf Magnus, University Medical Center Utrecht, Utrecht, Netherlands; j Stroke Pharmacogenomics and Genetics, Sant Pau Institute of Research, Hospital de la Santa Creu I Sant Pau, Barcelona, Spain; k Centre for the Prevention of Stroke and Dementia, Nuffield Department of Clinical Neuroscience, University of Oxford, Oxford, UK; l Clinical Neurosciences, University of Cambridge, Cambridge, UK; m Product Development Personalized Health Care, F Hoffmann-La Roche, Basel, Switzerland; n Institute of Neurointervention, Paracelsus Medical University, Salzburg, Austria; o Institute of Cardiovascular Research, Royal Holloway University of London, London, UK; p Neurovascular Research Group, Department of Neurology of Hospital del Mar-IMIM (Institut Hospital del Mar d'Investigacions Mediques), Universitat Autonoma de Barcelona/DCEXS-Universitat Pompeu Fabra, Barcelona, Spain; q Evelyn F McKnight Brain Institute, Department of Neurology, University of Miami Miller School of Medicine, Miami, FL, USA; r Neuroscience Institute, Saint Francis Medical Center, School of Health and Medical Sciences, Seton Hall University, South Orange, NJ, USA; s Department of Neurology, Skane University Hospital, Lund, Sweden; t Department of Clinical Sciences Lund, Neurology, Lund University, Lund, Sweden; u Department of Neurology, Mayo Clinic, Jacksonville, FL, USA; v Department of Public Health Solutions, Finnish Institute for Health and Welfare, Helsinki, Finland; w Institute for Molecular Medicine Finland (FIMM HiLIFE), Helsinki, Finland; x Center for Genomic Medicine, Massachusetts General Hospital, Boston, MA, USA; y Henry and Allison McCance Center for Brain Health, Massachusetts General Hospital, Boston, MA, USA; z Department of Neurology, Massachusetts General Hospital, Boston, MA, USA; aa J Philip Kistler Stroke Research Center, Massachusetts General Hospital, Boston, MA, USA; ab Program in Medical & Population Genetics, Broad Institute of Harvard and MIT, Cambridge, MA, USA; ac Division of Endocrinology, Diabetes and Nutrition, Department of Medicine, University of Maryland School of Medicine, Baltimore, MD, USA; ad Department of Neurology, University of Maryland School of Medicine, Baltimore, MD, USA; ae Geriatrics Research and Education Clinical Center, Baltimore Veterans Administration Medical Center, Baltimore, MD, USA; af Institute for Stroke and Dementia Research (ISD), LMU Munich, Munich, Germany; ag Munich Cluster for Systems Neurology (SyNergy), Munich, Germany; ah Clinical Neurosciences, University of Helsinki, Helsinki, Finland; ai Neurovascular Research Laboratory and Neurovascular Unit, Institut de Recerca, Hospital Vall d'Hebron, Universitat Autonoma de Barcelona, Barcelona, Spain; aj Experimental Neurology, Department of Neurosciences, KU Leuven, Leuven, Belgium; ak VIB Center for Brain & Disease Research, Department of Neurology, University Hospitals Leuven, Leuven, Belgium; al Centre for Clinical Brain Sciences, UK Dementia Research Institute and Row Fogo Centre for Research into the Ageing Brain, University of Edinburgh, Edinburgh, UK

## Abstract

**Background:**

The genetic basis of lacunar stroke is poorly understood, with a single locus on 16q24 identified to date. We sought to identify novel associations and provide mechanistic insights into the disease.

**Methods:**

We did a pooled analysis of data from newly recruited patients with an MRI-confirmed diagnosis of lacunar stroke and existing genome-wide association studies (GWAS). Patients were recruited from hospitals in the UK as part of the UK DNA Lacunar Stroke studies 1 and 2 and from collaborators within the International Stroke Genetics Consortium. Cases and controls were stratified by ancestry and two meta-analyses were done: a European ancestry analysis, and a transethnic analysis that included all ancestry groups. We also did a multi-trait analysis of GWAS, in a joint analysis with a study of cerebral white matter hyperintensities (an aetiologically related radiological trait), to find additional genetic associations. We did a transcriptome-wide association study (TWAS) to detect genes for which expression is associated with lacunar stroke; identified significantly enriched pathways using multi-marker analysis of genomic annotation; and evaluated cardiovascular risk factors causally associated with the disease using mendelian randomisation.

**Findings:**

Our meta-analysis comprised studies from Europe, the USA, and Australia, including 7338 cases and 254 798 controls, of which 2987 cases (matched with 29 540 controls) were confirmed using MRI. Five loci (ICA1L-WDR12-CARF-NBEAL1, ULK4, SPI1-SLC39A13-PSMC3-RAPSN, ZCCHC14, ZBTB14-EPB41L3) were found to be associated with lacunar stroke in the European or transethnic meta-analyses. A further seven loci (SLC25A44-PMF1-BGLAP, LOX-ZNF474-LOC100505841, FOXF2-FOXQ1, VTA1-GPR126, SH3PXD2A, HTRA1-ARMS2, COL4A2) were found to be associated in the multi-trait analysis with cerebral white matter hyperintensities (n=42 310). Two of the identified loci contain genes (COL4A2 and *HTRA1*) that are involved in monogenic lacunar stroke. The TWAS identified associations between the expression of six genes (*SCL25A44, ULK4, CARF, FAM117B, ICA1L, NBEAL1*) and lacunar stroke. Pathway analyses implicated disruption of the extracellular matrix, phosphatidylinositol 5 phosphate binding, and roundabout binding (false discovery rate <0·05). Mendelian randomisation analyses identified positive associations of elevated blood pressure, history of smoking, and type 2 diabetes with lacunar stroke.

**Interpretation:**

Lacunar stroke has a substantial heritable component, with 12 loci now identified that could represent future treatment targets. These loci provide insights into lacunar stroke pathogenesis, highlighting disruption of the vascular extracellular matrix *(COL4A2, LOX, SH3PXD2A, GPR126, HTRA1)*, pericyte differentiation (*FOXF2, GPR126*), TGF-β signalling *(HTRA1)*, and myelination (*ULK4, GPR126*) in disease risk.

**Funding:**

British Heart Foundation.

## Introduction

Lacunar strokes are small subcortical infarcts that arise from ischaemia in the territory of the deep perforating arteries of the brain.^1^, ^2^ They account for up to a quarter of all ischaemic strokes and are usually due to cerebral small vessel disease, which is also the most common pathology underlying intracerebral haemorrhage and vascular cognitive impairment. Radiologically, small vessel disease is also characterised by the presence of cerebral white matter hyperintensities, enlarged perivascular spaces, microbleeds, and brain atrophy.^3^ Few therapeutic interventions have been shown to reduce small vessel disease. One obstacle to developing new therapeutic approaches has been a lack of understanding of the underlying pathophysiology. One method that has been successfully used to discover pathophysiological processes and uncover potential treatment targets in other complex diseases is the use of genetic data derived from genome-wide association studies (GWAS). Recent GWAS have identified 35 loci associated with risk of ischaemic stroke and its major subtypes;^4^, ^5^, ^6^ but although many loci have been identified with the other major stroke subtypes (cardioembolic and large artery stroke), only one locus (16q24) has been robustly shown to be associated with lacunar stroke to date.^6^ This is surprising because lacunar stroke is the stroke subtype most likely to be caused by monogenic disease,^7^ and sporadic lacunar stroke has been strongly associated with a family history of stroke.^8^ Additionally, studies of other MRI markers of cerebral small vessel disease have shown a substantial genetic component; a recent GWAS identified 31 loci across three phenotypes.^9^

**Research in context**

**Evidence before this study**

Using the terms “stroke”, “small vessel stroke”, “lacunar stroke”, “small vessel disease”, “white matter hyperintensities”, “genetics”, and “GWAS”, we searched PubMed and GWAS Catalog for relevant reports published between Jan 1, 2010, and Jan 1, 2020. We considered only peer-reviewed reports in English. Previously, a single locus on chromosome 16q24 has been robustly associated specifically with lacunar stroke; by contrast, more than 30 loci have been associated with broad stroke phenotypes.

**Added value of this study**

The present findings substantially expand the number of genetic associations with lacunar stroke, with five loci now associated directly and a further seven associated with lacunar stroke jointly with white matter hyperintensities. These loci highlight several key mechanisms in lacunar stroke pathogenesis, including extracellular matrix dysfunction, myelination, and pericyte differentiation. The current findings also show that individuals with increased genetic predisposition to elevated blood pressure, smoking, and type 2 diabetes are at increased risk of lacunar stroke, suggesting a causal role for these factors.

**Implications of all the available evidence**

No treatments for prevention of lacunar stroke are available, aside from management of vascular risk factors, such as blood pressure. This is due in part to poor understanding of the mechanisms underlying the disease. Our findings highlight novel mechanisms underlying lacunar stroke pathogenesis, and therefore point to pathways that have potential to be targeted by therapeutics. Improved treatment of elevated blood pressure and type 2 diabetes, as well as prevention of smoking in the population, is likely to reduce the burden of lacunar stroke.

## Methods

### Study design and phenotype definitions

We did a GWAS of lacunar stroke to identify novel associations and provide mechanistic insights into lacunar stroke. We recruited cases of lacunar stroke from hospitals across the UK as part of the UK DNA Lacunar Stroke studies 1 and 2,^10^ and from collaborators within the International Stroke Genetics Consortium; and re-analysed data from previous studies.^11^, ^12^, ^13^, ^14^ Because MRI confirmation of lacunar stroke is more reliable than standard phenotyping,^15^, ^16^ we focused on recruiting MRI-confirmed cases. First, we did a GWAS of these data to identify novel genetic loci associated with lacunar stroke. Second, we used a multi-trait approach to detect additional genetic variation associated with lacunar stroke in a joint analysis with cerebral white matter hyperintensities from a large-scale GWAS.^9^ Third, we did a transcriptome-wide association study (TWAS) to identify transcribed genes for which expression is associated with lacunar stroke, and used mendelian randomisation to assess common cardiovascular risk factors that contribute to the disease. We did separate analyses in MRI-confirmed and standard phenotyping groups to assess whether MRI confirmation improves power to detect genetic associations.

For the GWAS, lacunar stroke cases were recruited from a combination of acute stroke admissions and outpatient services from Europe, the USA, South America, and Australia. Study inclusion criteria are detailed in the appendix (pp 99–114). Cases and controls were stratified by ancestry (European, south Asian, African, Hispanic) and analysed separately. We did two meta-analyses: a European ancestry analysis, and a transethnic analysis that included all ancestry groups. For each contributing study, approval for inclusion in this analysis complied with local ethical standards and with local institutional review board or ethics committee oversight. All participants provided written informed consent for genetic studies.

Lacunar stroke samples were divided into two strata: the MRI-confirmed group and the standard phenotyping group. In the MRI-confirmed group, lacunar stroke was defined as a clinical lacunar syndrome^17^ with an anatomically compatible lesion on MRI (subcortical infarct, ≤15 mm in diameter), either as a region of high intensity on diffusion-weighted imaging for acute infarcts or as a region of low intensity on fluid-attenuated inversion recovery or T1 imaging for non-acute infarcts,^3^ and the absence of causes of stroke other than small vessel disease. MRIs were centrally reviewed according to a standard proforma to confirm the diagnosis of lacunar stroke and identify any exclusion criteria. All patients underwent comprehensive stroke investigation, including brain MRI, imaging of the carotid arteries, and electrocardiogram. Echocardiography was done if appropriate. Exclusion criteria were stenosis of more than 50% in the extracranial or intracranial cerebral vessels, or previous carotid endarterectomy; cardioembolic source of stroke, defined according to criteria from the TOAST trial^11^ as high or moderate probability; cortical infarct on MRI; subcortical infarct of more than 15 mm in diameter, because these can be caused by embolic mechanisms (striatocapsular infarcts); and any other specific cause of stroke (eg, lupus anticoagulant, cerebral vasculitis, dissection, and monogenic cause of stroke).

In the standard phenotyping group, lacunar stroke was defined according to the TOAST criteria,^11^ based on a clinical lacunar syndrome and the absence of other causes of stroke, or non-lacunar infarction on CT.

### Genotyping and imputation

Genotyping arrays, quality control filters, and imputation reference panels are listed in the appendix (pp 7–8). All studies inferred the genetic ancestry of samples by comparison with reference populations using principal components analysis. European samples in this study are defined as those that segregated with European ancestry reference samples. The majority of studies were imputed to the Haplotype Reference Consortium build. If this was not possible due to logistical or ethical reasons, imputation to 1000 Genomes Phase 3 (all ancestry groups) panels was used.^14^, ^18^

### Statistical analysis

All studies used logistic regression to assess the association of single nucleotide polymorphism (SNP) allele dosages with lacunar stroke, including ancestry informative principal components as covariates as appropriate. All studies included cases with geographically matched controls, as confirmed by principal components analysis. Some studies had a combination of cases based on TOAST diagnosis of lacunar stroke, which were re-analysed for MRI confirmation of lacunar stroke, and cases based on TOAST diagnosis of lacunar stroke only, for which MRI was either not acquired or was inaccessible. In these circumstances, we analysed the MRI-confirmed and TOAST only (standard phenotyping) groups separately and divided the study controls between the two groups to avoid any sample overlap. Any cases with subsequent MRI confirmation of lacunar stroke were omitted from the TOAST only group; all individuals were analysed only once.

Meta-analysis was done based on a fixed-effects inverse-variance weighted procedure using the METAL tool.^19^ Meta-analysis was done in the MRI-confirmed and standard phenotyping groups separately, and in all studies combined. We used the principles described by Winkler and colleagues^20^ to scrutinise datasets used in the meta-analysis. For each study, we filtered out SNPs with imputation INFO scores of less than 0·7 or minor allele frequency (MAF) of less than 0·01. Additionally, we removed low frequency or poorly imputed SNPs in smaller studies by removing variants for which INFO × MAF × number of cases equalled less than 2.^5^ Genomic control correction based on genomic inflation factor λ was used for each study to adjust for any residual inflation.^21^ Linkage disequilibrium (LD) score intercept values were used to assess whether population structure had been sufficiently resolved at the meta-analysis level.^22^ After meta-analysis, we excluded SNPs that were not present in at least 50% of cases.

We defined significant loci as those containing SNPs with a p value of less than 5 × 10^−8^ and in linkage equilibrium (r^2^>0·1) with other lead SNPs. If multiple loci met these criteria within 1 Mb we used conditional and joint multiple SNP analysis (GCTA-cojo) to evaluate whether these SNPs remained genome-wide significant in a joint modelling scenario.^23^ We used Nagelkerke R^2^ values to calculate the proportion of variance explained by genome-wide significant SNPs using the NagelkerkeR2 function in the R fmsb library.^24^ To obtain an estimate of R^2^, we subtracted the R^2^ estimate for the model that included only principal components from the model that also contained the genome-wide significant SNPs. We used a genome-based restricted maximum likelihood (GREML) approach, implemented in GCTA software,^25^, ^26^ and LD score regression,^22^ to estimate the heritability of lacunar stroke (MRI-confirmed and non-confirmed).

We did multi-trait analysis of GWAS (MTAG),^27^ performing a joint analysis with a large study of cerebral white matter hyperintensities on MRI (n=42 310),^9^ which shares a common cause with lacunar stroke through cerebral small vessel disease, to uncover additional genetic variation associated with lacunar stroke. We considered associations significant if they had a p value of less than 5 × 10^−8^ in MTAG, had a p value of less than 0·05 for association with white matter hyperintensities and lacunar stroke in univariate analysis, and showed greater significance in MTAG than in univariate analyses for white matter hyperintensities or lacunar stroke. To confirm our associations, we used an alternative approach, Bayesian multivariate analysis of summary statistics (using R package BMASS).^28^

We used the TWAS approach to identify genes for which genetically altered expression was associated with lacunar stroke. Analyses were done using FUSION,^29^ from gene expression models derived from the Genotype-Tissue Expression (GTEx) portal V7,^30^ CommonMind Consortium (CMC),^31^ and Young Finns Study (YFS) datasets.^32^ The CMC gene expression tissues (labelled as CMC brain) were collected from the dorsolateral prefrontal cortex of individuals with schizophrenia or controls (TWAS n=452). In the YFS study (labelled as YFS whole blood), peripheral blood gene expression was analysed (TWAS n=1264). Among the available GTEx tissues, we focused our TWAS analysis on the aortic artery (TWAS n=267), coronary artery (TWAS n=152), tibial artery (TWAS n=388), and whole blood (TWAS n=369), based on the assumption that these tissues would be the most relevant for lacunar stroke pathogenesis. Bonferroni correction for multiple testing was applied, taking into account the total number of tested genes across the tissues (cutoff for significance: p<1·5 × 10^−6^). TWAS results were further investigated with colocalisation analysis of expression quantitative trait loci and GWAS signals with the R package COLOC,^33^ to assess whether the observed expression quantitative trait loci and GWAS associations were consistent with a shared association.

In a bioinformatics analysis for novel associations, we used Phenoscanner to query whether genome-wide significant SNPs were associated with DNA methylation,^34^, ^35^ metabolite levels, or protein levels from genome-wide studies at genome-wide significance (p<5 × 10^−8^) in other GWAS. We scanned DrugBank and the Drug Gene Interaction Database to assess the therapeutic potential of targeting associated genes.^36^, ^37^

To identify biological pathways significantly associated with risk of lacunar stroke, we used the multi-marker analysis of genomic annotation (MAGMA) tool.^38^ We first used MAGMA to calculate the significance of each gene based on association results, and then used these gene-level statistics to estimate enrichment of Gene Ontology pathways from the Molecular Signatures Database using a gene-set enrichment analysis approach.^39^ We investigated only Gene Ontology terms containing at least four and fewer than 200 genes and considered pathways attaining a false discovery rate (FDR) of less than 0·05 as being significantly associated with lacunar stroke.

We did mendelian randomisation analyses to evaluate whether any lipid (LDL, HDL, or triglycerides),^40^ blood pressure (systolic blood pressure, diastolic blood pressure, pulse pressure),^41^ metabolic (type 2 diabetes, body-mass index),^42^, ^43^ or lifestyle risk factors (ever smoking) have a causal impact on lacunar stroke based on genetics.^44^, ^45^ Instrumental variables were independent (LD r^2^<0·01) genome-wide significant (p<5 × 10^−8^) variants associated with each trait from previous analyses, and are listed in the appendix (pp 9–74). For blood pressure traits, we included SNPs associated at genome-wide significance with any of the three traits in all analyses. For body-mass index, we used the set of independent SNPs provided by study authors.^42^ We calculated the ratio of the SNP risk factor effect size by the corresponding effect size for lacunar stroke and aggregated effects across all risk factor-associated SNPs using an inverse-variance weighted procedure. As secondary analyses, we used median, weighted median, and MR-Egger approaches to aggregate across SNPs. We used the MR-Egger intercept to assess evidence of directional pleiotropy. In all analyses, we used the MendelianRandomization package in R.^46^ Results are presented as odds ratios (ORs) per genetically predicted increase in each risk factor (original scale).

### Role of the funding source

The funder had no role in study design, data collection, data analysis, data interpretation, or writing of the report.

## Results

**Figure 1:**
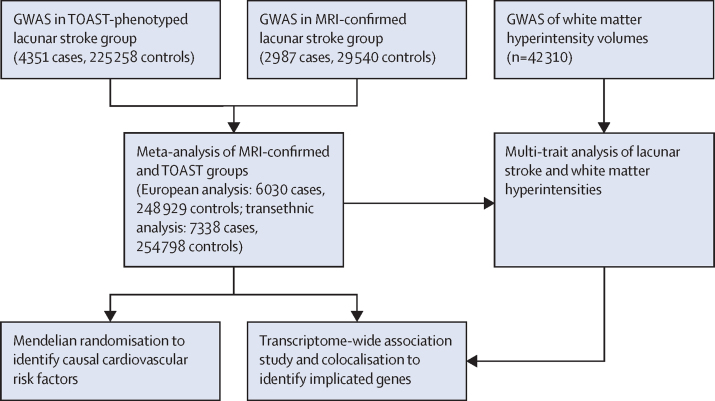
Analysis pipeline GWAS=genome-wide association study.

We meta-analysed studies from Europe, the USA, and Australia, including previous GWAS and additional cases and controls from the UK DNA Lacunar Stroke studies and the International Stroke Genetics Consortium, comprising a total of 6030 cases and 248 929 controls of European ancestry, and 7338 cases and 254 798 controls in the transethnic analysis (figure 1). 2987 (40·7%) cases (matched with 29 540 controls) had confirmation by MRI. Study cohorts, including genomic inflation λ values, are described in the appendix (pp 5–6, 100–11). Following meta-analysis, the LD score intercept value in the European analysis was 1·046 (SE 0·008) and the λ_1000_ value was 1·007, whereas in the transethnic analysis, the λ_1000_ value was 1·005, indicating no substantial inflation. SNP heritability (h^2^) of MRI-confirmed lacunar stroke, calculated using GREML methods in a European ancestry subset of 1693 cases and 10 171 controls genotyped on the same array, was 0·17–0·21 (SE 0·02), assuming stroke prevalence of 1–3%, and that 20% of these are lacunar strokes. Using LD score regression, estimates of SNP heritability were lower than GREML estimates, but were higher in the MRI-confirmed population (h^2^=0·065, SE 0·017) than in the non-MRI-confirmed population (0·0081, 0·0025). The genetic correlation between MRI-confirmed and non MRI-confirmed groups using LD score regression was significant (rg=0·61, SE 0·21, p=0·0033).

**Figure 2:**
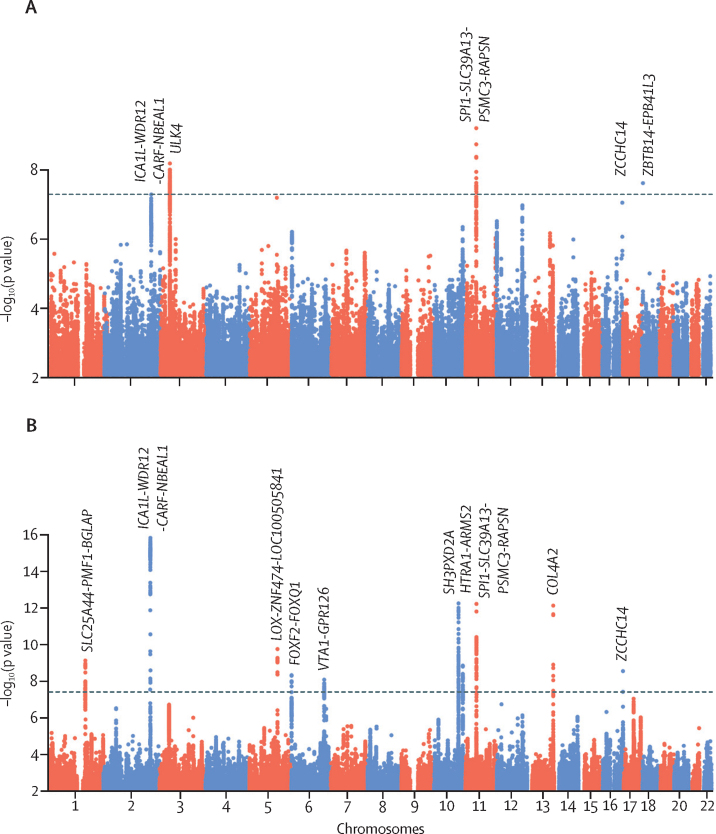
Manhattan plot of genome-wide SNP associations by genomic position (A) Lacunar stroke transethnic analysis. (B) Lacunar stroke multi-trait analysis. SNP=single nucleotide polymorphism. Dashed lines indicate the genome-wide significance threshold of 5 × 10^−8^.

Five loci were associated with lacunar stroke: three in European samples and three in the transethnic analysis, with one locus associated in both (table, figure 2). Regional association plots and forest plots for these loci are provided in the appendix (pp 115–134). Four of the loci were novel and one had been identified previously (16q24).^6^ One other locus (*ICA1L-WDR12-CARF-NBEAL1*) was associated in gene-based analyses in MEGASTROKE, and was associated in a recent multi-trait analysis of intracerebral haemorrhage and lacunar stroke.^5^, ^47^

**Table:** Genome-wide significant loci for lacunar stroke in univariate or multi-trait analysis

	**Chromosome**	Base position*	**Genomic context**	**Identifier**	**RA/OA**	**RAF**	**Lacunar stroke (European: 6030 cases, 219 389 controls)**			**Lacunar stroke (transethnic: 7338 cases, 225 258 controls)**		**White matter hyperintensities (n=42 310)**		**MTAG**
							OR (SE)	p value	Studies	OR (SE)	p value	β (SE)	p value	p value
**Genome-wide significance for lacunar stroke**														
*ICA1L-WDR12-CARF-NBEAL1*†	2	203 968 973	Intronic	rs72934535	T/C	0·89	1·25 (0·04)	3·7 × 10^−9^	12	1·22 (0·04)	5·2 × 10^−8^	0·070 (0·01)	2·8 × 10^−10^	5·3 × 10^−16^
*ULK4*†	3	41 839 370	Intronic	rs4621303	T/A	0·83	1·15 (0·03)	1·7 × 10^−7^	14	1·16 (0·03)	6·4 × 10^−9^	0·015 (0·01)	0·12	2·2 × 10^−7^‡
*SPI1-SLC39A13-PSMC3-RAPSN*	11	47 434 986	Exonic	rs2293576	G/A	0·67	1·14 (0·02)	7·2 × 10^−10^	14	1·14 (0·02)	6·0 × 10^−10^	0·030 (0·01)	3·1 × 10^−5^	6·4 × 10^−13^
*ZCCHC14*	16	87 575 332	Intergenic	rs12445022	A/G	0·34	1·13 (0·02)	2·5 × 10^−8^	13	1·12 (0·02)	9·0 × 10^−8^	0·019 (0·01)	0·0078	3·1 × 10^−9^
*ZBTB14-EPB41L3*	18	5 389 832	Intergenic	rs9958650	G/A	0·10	1·18 (0·03)	9·9 × 10^−7^	12	1·19 (0·03)	2·4 × 10^−8^	-0·011 (0·01)	0·33	0·0005
**Genome-wide significance in multi-trait analysis**														
*SLC25A44-PMF1-BGLAP*†	1	156 197 380	Intronic	rs2984613	C/T	0·64	1·10 (0·02)	2·5 × 10^−5^	13	1·09 (0·02)	1·4 × 10^−5^	0·037 (0·01)	2·3 × 10^−7^	8·2 × 10^−10^
LOX-ZNF474-LOC100505841	5	121 518 378	Downstream	rs2303655	T/C	0·81	1·14 (0·03)	3·6 × 10^−5^	11	1·12 (0·03)	0·00014	0·050 (0·01)	1·4 × 10^−8^	1·9 × 10^−10^
*FOXF2-FOXQ1*	6	1 366 718	Intergenic	rs7766042	C/T	0·11	1·17 (0·03)	3·7 × 10^−6^	11	1·18 (0·03)	1·2 × 10^−6^	0·045 (0·01)	7·1 × 10^−5^	5·2 × 10^−9^
*VTA1-GPR126*	6	142 562 417	Intergenic	rs225744	C/T	0·77	1·11 (0·03)	3·5 × 10^−5^	12	1·09 (0·02)	0·00050	0·037 (0·01)	5·8 × 10^−6^	9·2 × 10^−9^
*SH3PXD2A*	10	105 447 838	Intronic	rs61000833	T/C	0·60	1·10 (0·02)	1·7 × 10^−5^	12	1·07 (0·02)	0·0024	0·049 (0·01)	2·0 × 10^−12^	6·0 × 10^−13^
HTRA1-ARMS2	10	124 233 181	Intronic	rs79043147	T/C	0·07	1·21 (0·04)	3·2 × 10^−6^	11	1·22 (0·04)	1·1 × 10^−6^	0·057 (0·01)	1·8 × 10^−5^	1·6 × 10^−9^
*COL4A2*	13	111 040 681	Intronic	rs11838776	A/G	0·29	1·11 (0·02)	4·3 × 10^−6^	12	1·11 (0·02)	1·6 × 10^−6^	0·050 (0·01)	7·9 × 10^−11^	7·9 × 10^−13^

RA=risk allele. OA=other allele. RAF=risk allele frequency. MTAG=multi-trait analysis of genome-wide association study. SNP=single nucleotide polymorphism.

* Positions based on reference genome hg19.

† *ICA1L, CARF, NBEAL1, ULK4*, and *SLC25A44* were associated in TWAS analysis and confirmed by colocalization.

‡ Because A→T and C→G SNPs are removed by MTAG, results are presented for the SNP in highest linkage disequilibrium (rs9842261).

We next applied MTAG to identify additional genetic variation underlying lacunar stroke in a joint analysis with an aetiologically related trait, cerebral white matter hyperintensities (n=42 310). Genetic correlation between lacunar stroke and cerebral white matter hyperintensities, calculated using LD score regression,^22^ was substantial for the MRI-confirmed group (rg=0·46, SE 0·10, p=4·6 × 10^−6^) and slightly lower when including all lacunar strokes (0·37, 0·09, p=4·0 × 10^−5^). In the joint analysis with cerebral white matter hyperintensities, variants in seven additional loci reached genome-wide significance for lacunar stroke overall (table, figure 2). Four of these loci (*SLC25A44-PMF1-BGLAP, LOX-ZNF474-LOC100505841, SH3PXD2A*, and *COL4A2*) have previously been associated with white matter hyperintensities.^9^, ^48^ Regional association plots and forest plots for the loci are provided in the appendix (pp 120–134).

None of the 12 loci reaching genome-wide significance showed evidence of heterogeneity (p=0·05 to p=0·98; appendix pp 123–134). In two regions (*SH3PXD2A* and *HTRA1-ARMS2)* multiple apparently independent (LD r^2^<0·1) SNPs reached genome-wide significance. However, in a joint modelling scenario that was done using GCTA-cojo, only a single SNP at each of these regions had genome-wide significance, showing that a single variant remains the most parsimonious explanation of the association at this locus.^23^ We discarded two regions according to our protocol: one region on chromosome 17q25 showed an association solely with white matter hyperintensities, with no association with lacunar stroke (lead SNP p=0·39); a second region on chromosome 14 (*EVL-DEGS2*), was not as significant in MTAG analysis (p=1·2 × 10^−9^) as in white matter hyperintensities alone (p=1·2 × 10^−12^), so an independent contribution of lacunar stroke to the association could not be determined. Further evidence is required to determine whether these two regions are associated with lacunar stroke, so they were discarded from this analysis. The *ZBTB14-EPB41L3* locus that was associated with lacunar stroke was not associated with white matter hyperintensities (p=0·33 and effect in the opposite direction). Similarly, for the *ULK4* locus associated with lacunar stroke, the lead SNP did not reach significance for white matter hyperintensities (p=0·12), but it was in the consistent effect direction and thus could reflect a lack of study power.

The 12 loci showed stronger effects in the MRI-confirmed group than in the standard phenotyping group (in European ancestry analysis, appendix p 86), although the difference was not statistically significant (one-tailed p=0·07), with a median proportional increase in OR of 3·4%. The 12 loci explained 1·4% of the overall heritability, and 6·5–8·1% of the lacunar stroke heritability from GWAS arrays, as calculated in this study.

**Figure 3:**
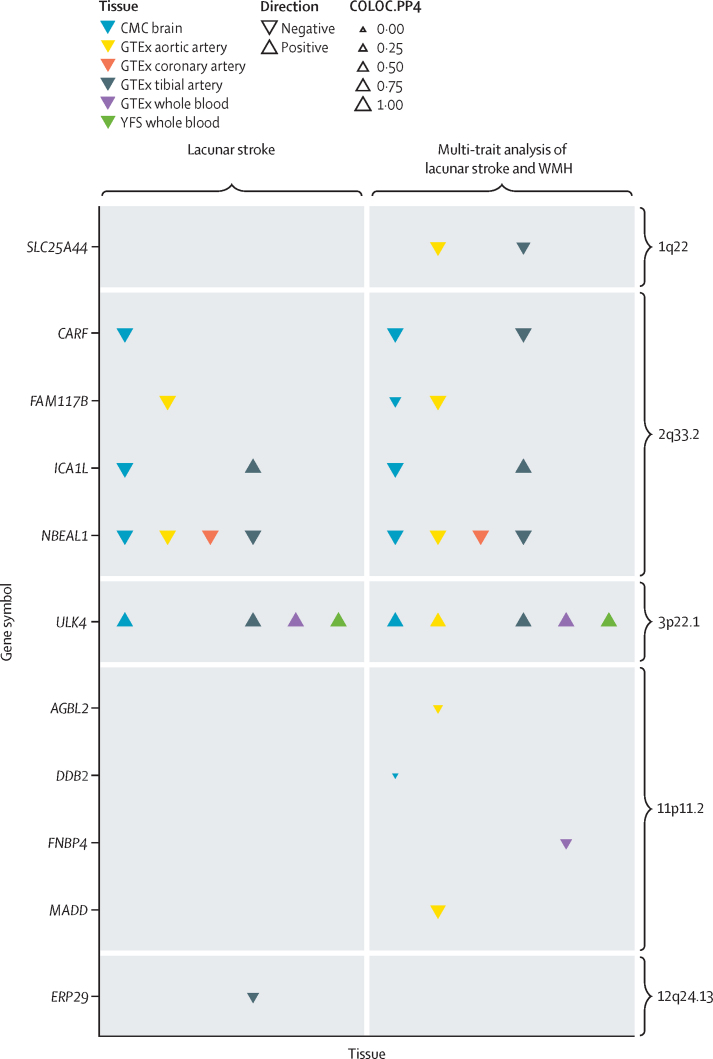
Genes for which expression is associated with lacunar stroke in six tissues from transcriptome-wide association analysis Evidence of colocalisation of gene expression and lacunar stroke signals is shown by triangle size, with larger triangles indicating stronger evidence of colocalisation. CMC=CommonMind Consortium. COLOC.PP4=posterior probability of hypothesis 4 in colocalisation analysis (that there is a consistent association between lacunar stroke and expression of the given gene). GTEx=Genotype-Tissue Expression portal. WMH=white matter hyperintensities. YFS=Young Finns Study.

We did a TWAS to identify genes for which expression was associated with lacunar stroke (figure 3). Genetically elevated expression of *SLC25A44* was associated with lacunar stroke in multi-trait analysis in arterial tissues, whereas genetically decreased expression of *ULK4* was associated with lacunar stroke in arterial tissues, whole blood, and the brain. At the 2q33·2 locus, genetically elevated expression of *CARF, FAM117B, ICA1L*, and *NBEAL1* were all associated with lacunar stroke. All associations were confirmed by colocalisation analysis between the gene expression and lacunar stroke associations (posterior probability >0·7). Five other associations were identified in the TWAS, but these were not confirmed by colocalisation analysis (posterior probability <0·7, figure 3).

We used Phenoscanner to evaluate whether the 12 lead SNPs were associated with DNA methylation, metabolite levels, or protein levels from large-scale studies.^49^, ^50^, ^51^ 11 of the 12 lead SNPs showed associations with DNA methylation at genome-wide significance, which was more than expected by chance based on randomly selected SNPs across the genome (p<0·01). Ten of the SNPs were associated in multiple independent studies (appendix pp 87–97). Conversely, none of the 12 SNPs were associated with metabolite or protein levels.

Querying databases that catalogue drug-gene relationships showed that 11 of the genes listed in table are categorised as druggable, indicating that they have potential for therapeutic development (appendix p 98). However, no existing drugs target any of the genes identified in this study.

A pathway analysis based on the multi-trait analysis results using MAGMA identified five significantly associated Gene Ontology gene sets: phosphatidylinositol 5 phosphate binding (p=2·2 × 10^−6^, FDR 0·020), extracellular matrix structural constituent (p=6·2 × 10^−6^, 0·027), extracellular matrix constituent conferring elasticity (p=8·9 × 10^−6^, 0·027), middle ear morphogenesis (p=2·3 × 10^−5^, 0·049), and roundabout binding (p=2·7 × 10^−5^, 0·049). No pathways were significant when based solely on lacunar stroke results. Results for all pathways with FDR of less than 0·5 are presented in the appendix (pp 75–76).

**Figure 4:**
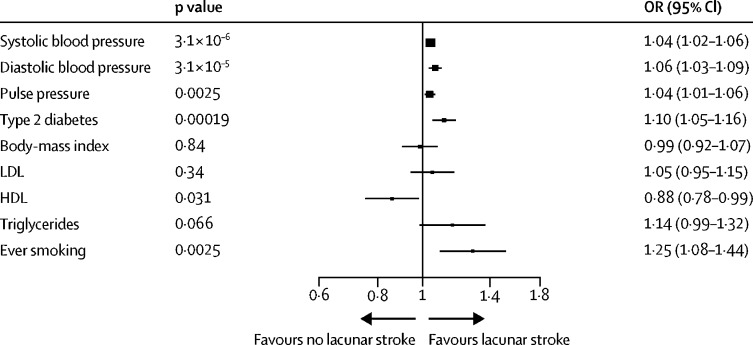
Associations between genetically proxied cardiovascular risk factors and lacunar stroke from mendelian randomisation analysis using the inverse variance weighted method Estimates are presented as ORs per genetically proxied increase in each risk factor (original scale). OR=odds ratio.

Mendelian randomisation analyses using an inverse variance weighted approach found positive associations of diastolic, systolic, and pulse pressure, type 2 diabetes, and ever smoking with lacunar stroke (figure 4). No significant finding showed any evidence of pleiotropy, as assessed using the MR-Egger intercept. There was some evidence of a negative association between HDL and lacunar stroke, but this result did not reach Bonferroni-corrected significance. There was no evidence of an association with body-mass index, low density lipoprotein or triglycerides. Secondary analysis for all risk factors using median, weighted median, and MR-Egger approaches are presented in the appendix (pp 135–143).

## Discussion

Despite the public health importance of lacunar stroke as the cause of a quarter of all strokes, previous GWAS studies have only identified one genetic locus associated with the disease, in contrast with the 35 identified for ischaemic stroke and its major subtypes.^5^ We did a GWAS of lacunar stroke, including the largest number of cases with MRI confirmation to date, identifying 11 novel loci in addition to replicating the one previously reported locus.

The primary analysis identified four novel loci. One association on chromosome 11, encompassing *SPI1-SLC39A13-PSMC3-RAPSN*, was identified in both European and transethnic analyses. The lead SNP is a synonymous variant in *SLC39A13*, which encodes for solute carrier family 39 member 13, a transmembrane protein with roles in zinc transport. Mutations in this gene cause a form of Ehlers-Danlos syndrome, a group of connective tissues disorders that affect the vasculature and can cause stroke;^52^ vascular abnormalities have been reported in *SLC39A13*-knockout mice.^53^ We additionally identified a locus for which the lead SNP resides in an intron of *ULK4* (encoding for serine/threonine-protein kinase ULK4) on chromosome 3. The TWAS analysis suggests *ULK4* is the most likely implicated gene, with genetically decreased expression of *ULK4* being associated with lacunar stroke. Variants in close LD with the lead SNP have been implicated in diastolic blood pressure in large-scale GWAS.^54^ However, the direction of effect was opposite to that for lacunar stroke, suggesting this likely reflects pleiotropy rather than a causal pathway. Variants in close LD have also been associated with another cardiovascular disease, acute aortic dissection.^55^ ULK4 belongs to the family of serine-threonine protein kinases, a group of phosphorylating kinases involved in diverse processes including cell proliferation and differentiation, apoptosis, and embryonic development. Its deficiency leads to hypomyelination,^56^ and it has been associated with neuropsychiatric traits.^57^ Finally, we report a novel association on chromosome 18, located between *ZBTB14*, encoding a zinc finger transcription factor (zinc finger and BTB domain-containing protein 14), and *EPB41L3*, encoding for a membrane protein that inhibits cell proliferation and promotes apoptosis (erythrocyte membrane protein band 4·1-like 3).

In multi-trait analysis we identified seven further loci, all of which are reported as being associated with lacunar stroke at genome-wide significance for the first time. Two have not been reported as being associated with any cerebrovascular disease previously. One locus lies in an intergenic region between the *VTA1* (vacuolar protein sorting-associated protein VTA1 homolog) and *GPR126* (G protein-coupled receptor 126) genes. GPR126 is activated by type IV collagen and has an important role in myelination.^58^ GPR126 also binds laminin-211,^59^ an extracellular matrix protein produced by astrocytes and present in the brain, with key roles in development and function of the blood-brain barrier,^60^ in part through regulation of pericyte differentiation, a mechanism previously implicated through the *FOXF2* gene.^61^, ^62^ Small vessel disease-related endothelial dysfunction has also been shown to prevent oligodendrocyte precursor cell maturation, contributing to impaired myelination.^63^ One hypothesis is that the *GPR126* variant might exacerbate this process, inhibiting repair from myelin damage. The second previously unreported association lies in an intergenic region, the nearest gene to which is *HTRA1* (encoding for serine protease HTRA1), a gene in which rare homozygous variation leads to cerebral autosomal recessive arteriopathy with subcortical infarcts and leucoencephalopathy.^64^ HTRA1, through processing of LTBP-1, promotes TGF-β signalling in the vascular extracellular matrix (ECM).^65^ The presence of both rare and common risk variants in *HTRA1* points to it being a key molecule in lacunar stroke pathogenesis, and is a feature shared with another gene identified in this study, *COL4A2*, in which rare variants also cause monogenic forms of cerebral small vessel disease.^7^ Candidate gene studies have previously shown associations not reaching genome-wide significance in *COL4A2* with lacunar stroke and the same region has also previously been associated with intracerebral haemorrhage in multi-trait analysis, and coronary artery disease.^47^, ^66^, ^67^ Four other loci identified (*SH3PXD2A, LOX-ZNF474-LOC100505841, SLC25A44-PMF1-BGLAP, FOXF2-FOXQ1*) were associated with broad stroke in MEGASTROKE (associations of all SNPs in MEGASTROKE are shown in the appendix pp 11–18) or a previous meta-analysis,^5^, ^62^ although, to our knowledge, this is the first study to confirm their association specifically with lacunar stroke. At the *SLC25A44-PMF1-BGLAP* locus, the TWAS results point to an association of genetically elevated expression of *SLC25A44* with lacunar stroke, which was validated in colocalisation analysis. *SLC25A44* has an important role in catabolism of branched-chain amino acids in brown adipose tissue by transporting them into mitochondria,^68^ and thus has potential as a mediating factor in the relationship between metabolic disease and lacunar stroke. However, variants in close LD have also been associated with mosaic Y chromosome loss,^69^ highlighting mosaicism as an alternative mechanism. Further functional studies will be required to untangle these relationships with lacunar stroke. The strength of the association for all associated variants was moderate to large in the context of GWAS (OR of 1·10 to 1·25 in Europeans) and notably larger than effects previously reported for variants associated with broad stroke phenotypes.^5^ This observation is consistent with the variants acting specifically on the lacunar stroke subtype rather than on stroke as a whole.

We also found that 11 of the 12 lead SNPs influence DNA methylation at genome-wide significance, pointing to epigenetic changes being one source through which risk of lacunar stroke is conferred. Whether this genetically altered DNA methylation influences transcription of nearby genes, and which genes are affected, should be the focus of further study. A pathway analysis implicated several biological processes in lacunar stroke pathophysiology. Two pathways involved the ECM, the network of extracellular molecules that provide scaffolding and biochemical support to surrounding tissues. Disruption of the vascular ECM has been hypothesised to be a key component in pathogenesis of cerebral small vessel disease, particularly in monogenic forms, and several of the genes implicated in this study (*COL4A2, LOX, SH3PXD2A, GPR126, HTRA1*) have key roles in the ECM.^70^ Therefore, our findings support this hypothesis and suggest ECM dysfunction also has a key role in sporadic cerebral small vessel disease.

We did Mendelian randomisation to assess whether cardiovascular risk factors showed evidence of a causal association with lacunar stroke. We found support for genetically predicted elevated blood pressure (systolic, diastolic, and pulse pressure), type 2 diabetes, and smoking being associated with lacunar stroke. The results are consistent with those from observational studies and suggest that targeting these factors would reduce risk of lacunar stroke.^71^ There was evidence, not reaching Bonferroni-corrected significance, for a protective effect of increased HDL on risk of lacunar stroke, and no association with LDL, replicating findings in previous studies.^72^, ^73^ Overall these findings show that the impact of the direct effects of LDL-lowering medications, such as statins, on the incidence of lacunar stroke is likely to be small.

Our study emphasises the benefit of accurate phenotyping using MRI. Using this approach, the heritability of lacunar stroke using GREML was substantial, and larger than previous estimates based on TOAST subtyping.^74^ Using LD score, the heritability was larger in the MRI-confirmed group, but estimates were considerably lower than for GREML. The use of MRI subtyping also increased the strength of association for the lacunar stroke-associated variants, although this increase was not statistically significant. These results suggest that further genetic risk factor studies in lacunar stroke are likely to be more successful if MRI subtyping is used.

Our study has limitations. The analysis was done in a predominantly European ancestry population. Large studies including diverse ancestries should be done to assess the generalisability of our findings to all ethnic groups. The MTAG approach relies on the relatively strong assumption that associated variants act on both traits, which might not always be the case for white matter hyperintensities and lacunar stroke, because they reflect downstream effects of a shared common ancestor, small vessel disease. To control for this, we considered only SNPs showing an association with both traits and showing greater significance in MTAG analysis than with white matter hyperintensities or lacunar stroke alone, as being significant. However, independent replication will remain the gold standard for confirming these and all other reported associations in this Article. Previous studies have suggested that a more conservative threshold of a p value less than 1 × 10^−8^ should be considered in GWAS using larger imputation panels such as this study.^75^ If we were to use this threshold, one locus (*ZBTB14-EPB41L3*) would no longer be significant. Additional caution should therefore be applied when interpreting this finding, particularly as it was not significant in MTAG analysis. To increase sample size and study power, we used publicly available controls in analyses. As such, we could not determine whether these individuals had a history of lacunar stroke. Our analyses did not adjust for age and sex, and there is an ongoing debate about the importance of including such covariates in genetic studies.^76^ In analyses with substantial differences between case and control populations, subtle biases could occur.

In summary, these findings represent substantial progress in identifying the genetic mechanisms underlying lacunar stroke, a disease for which little is known about the molecular causes. Our findings highlight diverse mechanisms contributing to the disease, implicating disruption of the vascular ECM (*COL4A2, LOX, SH3PXD2A, GPR126, HTRA1*), pericyte differentiation (*FOXF2, GPR126*), TGF-β signalling (*HTRA1*), and myelination (*ULK4, GPR126*) in disease risk. These results provide novel insights into the pathogenesis of lacunar stroke, and highlight multiple candidates for functional experiments to identify the specific mechanisms conferring risk of lacunar stroke that could be targeted therapeutically.

For **FUSION software** see https://github.com/gusevlab/fusion_twas

## Data sharing

GWAS summary statistics from these analyses are available at GWAS Catalog and on the Cerebrovascular Disease Knowledge Portal. Individual-level data from the NINDS Stroke Genetics Network study are available to researchers through dbGAP.

## References

[1] Pantoni L (2010). Cerebral small vessel disease: from pathogenesis and clinical characteristics to therapeutic challenges. Lancet Neurol.

[2] Wardlaw JM, Smith C, Dichgans M (2019). Small vessel disease: mechanisms and clinical implications. Lancet Neurol.

[3] Wardlaw JM, Smith EE, Biessels GJ (2013). Neuroimaging standards for research into small vessel disease and its contribution to ageing and neurodegeneration. Lancet Neurol.

[4] Malik R, Rannikmäe K, Traylor M (2018). Genome-wide meta-analysis identifies 3 novel loci associated with stroke. Ann Neurol.

[5] Malik R, Chauhan G, Traylor M (2018). Multiancestry genome-wide association study of 520,000 subjects identifies 32 loci associated with stroke and stroke subtypes. Nat Genet.

[6] Traylor M, Malik R, Nalls MA (2017). Genetic variation at 16q24.2 is associated with small vessel stroke. Ann Neurol.

[7] Tan R, Traylor M, Rutten-Jacobs L, Markus H (2017). New insights into mechanisms of small vessel disease stroke from genetics. Clin Sci (Lond).

[8] Jerrard-Dunne P, Cloud G, Hassan A, Markus HS (2003). Evaluating the genetic component of ischemic stroke subtypes: a family history study. Stroke.

[9] Persyn E, Hanscombe KB, Howson JMM, Lewis CM, Traylor M, Markus HS (2020). Genome-wide association study of MRI markers of cerebral small vessel disease in 42,310 participants. Nat Commun.

[10] Kilarski LL, Rutten-Jacobs LCA, Bevan S (2015). Prevalence of CADASIL and Fabry disease in a cohort of MRI defined younger onset lacunar stroke. PLoS One.

[11] Adams HP, Bendixen BH, Kappelle LJ (1993). Classification of subtype of acute ischemic stroke. Definitions for use in a multicenter clinical trial. TOAST. Trial of Org 10172 in acute stroke treatment. Stroke.

[12] Bellenguez C, Bevan S, Gschwendtner A (2012). Genome-wide association study identifies a variant in HDAC9 associated with large vessel ischemic stroke. Nat Genet.

[13] NINDS Stroke Genetics Network (SiGN), International Stroke Genetics Consortium (ISGC) (2016). Loci associated with ischaemic stroke and its subtypes (SiGN): a genome-wide association study. Lancet Neurol.

[14] McCarthy S, Das S, Kretzschmar W (2016). A reference panel of 64,976 haplotypes for genotype imputation. Nat Genet.

[15] Rajajee V, Kidwell C, Starkman S (2008). Diagnosis of lacunar infarcts within 6 hours of onset by clinical and CT criteria versus MRI. J Neuroimaging.

[16] Markus HS, Khan U, Birns J (2007). Differences in stroke subtypes between black and white patients with stroke: the South London Ethnicity and Stroke Study. Circulation.

[17] Bamford J, Sandercock P, Dennis M, Burn J, Warlow C (1991). Classification and natural history of clinically identifiable subtypes of cerebral infarction. Lancet.

[18] Auton A, Brooks LD, Durbin RM (2015). A global reference for human genetic variation. Nature.

[19] Willer CJ, Li Y, Abecasis GR (2010). METAL: fast and efficient meta-analysis of genomewide association scans. Bioinformatics.

[20] Winkler TW, Day FR, Croteau-Chonka DC (2014). Quality control and conduct of genome-wide association meta-analyses. Nat Protoc.

[21] Devlin B, Roeder K (1999). Genomic control for association studies. Biometrics.

[22] Bulik-Sullivan BK, Loh PR, Finucane HK (2015). LD score regression distinguishes confounding from polygenicity in genome-wide association studies. Nat Genet.

[23] Yang J, Ferreira T, Morris AP (2012). Conditional and joint multiple-SNP analysis of GWAS summary statistics identifies additional variants influencing complex traits. Nat Genet.

[24] Nagelkerke NJ (1991). A note on a general definition of the coefficient of determination. Biometrika.

[25] Lee SH, DeCandia TR, Ripke S (2012). Estimating the proportion of variation in susceptibility to schizophrenia captured by common SNPs. Nat Genet.

[26] Yang J, Lee SH, Goddard ME, Visscher PM (2011). GCTA: a tool for genome-wide complex trait analysis. Am J Hum Genet.

[27] Turley P, Walters RK, Maghzian O (2018). Multi-trait analysis of genome-wide association summary statistics using MTAG. Nat Genet.

[28] Turchin MC, Stephens M (2019). Bayesian multivariate reanalysis of large genetic studies identifies many new associations. PLoS Genet.

[29] Gusev A, Ko A, Shi H (2016). Integrative approaches for large-scale transcriptome-wide association studies. Nat Genet.

[30] Battle A, Brown CD, Engelhardt BE, Montgomery SB (2017). Genetic effects on gene expression across human tissues. Nature.

[31] Fromer M, Roussos P, Sieberts SK (2016). Gene expression elucidates functional impact of polygenic risk for schizophrenia. Nat Neurosci.

[32] Raitakari OT, Juonala M, Rönnemaa T (2008). Cohort profile: the cardiovascular risk in Young Finns Study. Int J Epidemiol.

[33] Giambartolomei C, Vukcevic D, Schadt EE (2014). Bayesian test for colocalisation between pairs of genetic association studies using summary statistics. PLoS Genet.

[34] Staley JR, Blackshaw J, Kamat MA (2016). PhenoScanner: a database of human genotype-phenotype associations. Bioinformatics.

[35] Kamat MA, Blackshaw JA, Young R (2019). PhenoScanner V2: an expanded tool for searching human genotype-phenotype associations. Bioinformatics.

[36] Wishart DS, Feunang YD, Guo AC (2018). DrugBank 5.0: a major update to the DrugBank database for 2018. Nucleic Acids Res.

[37] Cotto KC, Wagner AH, Feng YY (2018). DGIdb 3.0: a redesign and expansion of the drug-gene interaction database. Nucleic Acids Res.

[38] de Leeuw CA, Mooij JM, Heskes T, Posthuma D (2015). MAGMA: generalized gene-set analysis of GWAS data. PLoS Comput Biol.

[39] Subramanian A, Tamayo P, Mootha VK (2005). Gene set enrichment analysis: a knowledge-based approach for interpreting genome-wide expression profiles. Proc Natl Acad Sci USA.

[40] Teslovich TM, Musunuru K, Smith AV (2010). Biological, clinical and population relevance of 95 loci for blood lipids. Nature.

[41] Hoffmann TJ, Ehret GB, Nandakumar P (2017). Genome-wide association analyses using electronic health records identify new loci influencing blood pressure variation. Nat Genet.

[42] Yengo L, Sidorenko J, Kemper KE (2018). Meta-analysis of genome-wide association studies for height and body mass index in ∼700000 individuals of European ancestry. Hum Mol Genet.

[43] Mahajan A, Taliun D, Thurner M (2018). Fine-mapping type 2 diabetes loci to single-variant resolution using high-density imputation and islet-specific epigenome maps. Nat Genet.

[44] Liu M, Jiang Y, Wedow R (2019). Association studies of up to 1.2 million individuals yield new insights into the genetic etiology of tobacco and alcohol use. Nat Genet.

[45] Haycock PC, Burgess S, Wade KH, Bowden J, Relton C, Davey Smith G (2016). Best (but oft-forgotten) practices: the design, analysis, and interpretation of Mendelian randomization studies. Am J Clin Nutr.

[46] Yavorska OO, Burgess S (2017). MendelianRandomization: an R package for performing Mendelian randomization analyses using summarized data. Int J Epidemiol.

[47] Chung J, Marini S, Pera J (2019). Genome-wide association study of cerebral small vessel disease reveals established and novel loci. Brain.

[48] Verhaaren BF, Debette S, Bis JC (2015). Multiethnic genome-wide association study of cerebral white matter hyperintensities on MRI. Circ Cardiovasc Genet.

[49] Bonder MJ, Luijk R, Zhernakova DV (2017). Disease variants alter transcription factor levels and methylation of their binding sites. Nat Genet.

[50] Gaunt TR, Shihab HA, Hemani G (2016). Systematic identification of genetic influences on methylation across the human life course. Genome Biol.

[51] Chen L, Ge B, Casale FP (2016). Genetic drivers of epigenetic and transcriptional variation in human immune cells. Cell.

[52] Bin BH, Hojyo S, Hosaka T (2014). Molecular pathogenesis of spondylocheirodysplastic Ehlers-Danlos syndrome caused by mutant ZIP13 proteins. EMBO Mol Med.

[53] Hirose T, Shimazaki T, Takahashi N (2019). Morphometric analysis of thoracic aorta in Slc39a13/Zip13-KO mice. Cell Tissue Res.

[54] Ehret GB, Munroe PB, Rice KM (2011). Genetic variants in novel pathways influence blood pressure and cardiovascular disease risk. Nature.

[55] Guo DC, Grove ML, Prakash SK (2016). Genetic variants in LRP1 and ULK4 are associated with acute aortic dissections. Am J Hum Genet.

[56] Liu M, Xu P, Guan Z (2018). Ulk4 deficiency leads to hypomyelination in mice. Glia.

[57] Liu M, Xu P, O'Brien T, Shen S (2017). Multiple roles of Ulk4 in neurogenesis and brain function. Neurogenesis (Austin).

[58] Paavola KJ, Sidik H, Zuchero JB, Eckart M, Talbot WS (2014). Type IV collagen is an activating ligand for the adhesion G protein-coupled receptor GPR126. Sci Signal.

[59] Mehta P, Piao X (2017). Adhesion G-protein coupled receptors and extracellular matrix proteins: Roles in myelination and glial cell development. Dev Dyn.

[60] Menezes MJ, McClenahan FK, Leiton CV, Aranmolate A, Shan X, Colognato H (2014). The extracellular matrix protein laminin α2 regulates the maturation and function of the blood-brain barrier. J Neurosci.

[61] Yao Y, Chen ZL, Norris EH, Strickland S (2014). Astrocytic laminin regulates pericyte differentiation and maintains blood brain barrier integrity. Nat Commun.

[62] Chauhan G, Arnold CR, Chu AY (2016). Identification of additional risk loci for stroke and small vessel disease: a meta-analysis of genome-wide association studies. Lancet Neurol.

[63] Rajani RM, Quick S, Ruigrok SR (2018). Reversal of endothelial dysfunction reduces white matter vulnerability in cerebral small vessel disease in rats. Sci Transl Med.

[64] Hara K, Shiga A, Fukutake T (2009). Association of HTRA1 mutations and familial ischemic cerebral small-vessel disease. N Engl J Med.

[65] Beaufort N, Scharrer E, Kremmer E (2014). Cerebral small vessel disease-related protease HtrA1 processes latent TGF-β binding protein 1 and facilitates TGF-β signaling. Proc Natl Acad Sci USA.

[66] Rannikmäe K, Sivakumaran V, Millar H (2017). *COL4A2* is associated with lacunar ischemic stroke and deep ICH: meta-analyses among 21,500 cases and 40,600 controls. Neurology.

[67] Nikpay M, Goel A, Won HH (2015). A comprehensive 1,000 Genomes-based genome-wide association meta-analysis of coronary artery disease. Nat Genet.

[68] Yoneshiro T, Wang Q, Tajima K (2019). BCAA catabolism in brown fat controls energy homeostasis through SLC25A44. Nature.

[69] Wright DJ, Day FR, Kerrison ND (2017). Genetic variants associated with mosaic Y chromosome loss highlight cell cycle genes and overlap with cancer susceptibility. Nat Genet.

[70] Joutel A, Haddad I, Ratelade J, Nelson MT (2016). Perturbations of the cerebrovascular matrisome: a convergent mechanism in small vessel disease of the brain?. J Cereb Blood Flow Metab.

[71] Rutten-Jacobs LCA, Markus HS (2017). Vascular risk factor profiles differ between magnetic resonance imaging-defined subtypes of younger-onset lacunar stroke. Stroke.

[72] Hindy G, Engström G, Larsson SC (2018). Role of blood lipids in the development of ischemic stroke and its subtypes: a mendelian randomization study. Stroke.

[73] Georgakis MK, Malik R, Anderson CD, Parhofer KG, Hopewell JC, Dichgans M (2020). Genetic determinants of blood lipids and cerebral small vessel disease: role of high-density lipoprotein cholesterol. Brain.

[74] Bevan S, Traylor M, Adib-Samii P (2012). Genetic heritability of ischemic stroke and the contribution of previously reported candidate gene and genomewide associations. Stroke.

[75] Wu Y, Zheng Z, Visscher PM, Yang J (2017). Quantifying the mapping precision of genome-wide association studies using whole-genome sequencing data. Genome Biol.

[76] Pirinen M, Donnelly P, Spencer CC (2012). Including known covariates can reduce power to detect genetic effects in case-control studies. Nat Genet.

