# Supplementary material for: Genetic basis of lacunar stroke: a pooled analysis of individual patient data and genome-wide association studies
Source: Lancet Neurol. 2021 May;20(5):351–61. doi: 10.1016/S1474-4422(21)00031-4 (PMC8062914; doi:10.1016/S1474-4422(21)00031-4)
Supplement: Supplementary appendix [file mmc1.pdf]

THE LANCET
Neurology

Supplementary appendix

This appendix formed part of the original submission and has been peer reviewed. We post it as supplied by the authors.

Supplement to: Traylor M, Persyn E, Tomppo L, et al. Genetic basis of lacunar stroke: a pooled analysis of individual patient data and genome-wide association studies. *Lancet Neurol* 2021; published online March 25. https://doi.org/10.1016/S1474-4422(21)00031-4.

Supplementary Data

Table of Contents

Supplementary Table 1 – Sample Characteristics 5

Supplementary Table 2 – Genotyping and Imputation 7

Supplementary Table 2 – Instrumental Variables used in Mendelian Randomization Analysis 9

Supplementary Table 3 – Pathway Analysis Results From Multi-trait analysis with FDR<0.5 75

Supplementary Table 4 - Associations of Genome-wide Significant SNPs identified in MEGASTROKE with Lacunar Stroke (Europeans) in this analysis 77

Supplementary Table 5 - Associations of Genome-wide Significant SNPs identified in MEGASTROKE with Lacunar Stroke (Transethnic) in this analysis 78

Supplementary Table 6 - Association Results for 12 SNPs associated with Lacunar Stroke in this analysis in MEGASTROKE All Stroke (European) Analysis 79

Supplementary Table 7 - Association Results for 12 SNPs associated with Lacunar Stroke in this analysis in MEGASTROKE All Stroke (Transethnic) Analysis 80

Supplementary Table 8 - Association Results for 12 SNPs associated with Lacunar Stroke in this analysis in MEGASTROKE All Ischaemic Stroke (European) Analysis 81

Supplementary Table 9 - Association Results for 12 SNPs associated with Lacunar Stroke in this analysis in MEGASTROKE All Ischaemic Stroke (Transethnic) Analysis 82

Supplementary Table 10 – Association Results for 12 SNPs associated with Lacunar Stroke in this analysis in MEGASTROKE Small Vessel Stroke (European) Analysis 83

Supplementary Table 11 - Association Results for 12 SNPs associated with Lacunar Stroke in this analysis in MEGASTROKE Small Vessel Stroke (Transethnic) Analysis 84

Supplementary Table 12 – Full Results for 12 Associated Loci, Including BMASS Results 85

Supplementary Table 13 – Association Statistics for 12 Variants Associated with Lacunar Stroke from MRI Confirmed and Standard Phenotyping Groups Separately in European ancestry individuals 86

OR, Odds Ratio; SE, standard error; OR Ratio, Ratio of Odds Ratios between MRI Confirmed and Standard Phenotyping groups. 86

Supplementary Table 14 – Associations of Genome-wide Significant Variants identified in this analysis with Epigenetic markers from Genome wide Association Studies reaching p<5e-8 87

Description of Study Populations 99

Supplementary Figures 1-2. QQ-plots of Observed against Expected -log10(p-values) for European (Left) and Transethnic (Right) Genome-wide Analyses 115

Supplementary Figure 3. SPI1-SLC39A13-PSMC3-RAPSN Region in European (left) and Transethnic (right) analysis 116

Supplementary Figure 4. ICA1L-WDR12-CARF-NBEAL1 Region in European (left) and Transethnic (right) analysis 117

Supplementary Figure 5. ZCCHC14 Region in European (left) and Transethnic (right) analysis 118

....118

Supplementary Figure 6. ULK4 Region in European (left) and Transethnic (right) analysis 119
.... 119
Supplementary Figure 7. ZBTB14-EPB41L3 Region in European (left) and Transethnic (right) analysis 120
Supplementary Figures 8-11. Regions achieving genome-wide significance in multi-trait analysis 121
Supplementary Figures 12-15. Regions achieving genome-wide significance in multi-trait analysis 122
.... 122
.... 122
Supplementary Figures 16-17. Regions achieving genome-wide significance in multi-trait analysis 123
.... 123
.... 123
Supplementary Figure 18 – Forest Plot for Association of rs72934535 at ICA1L-WDR12-CARF-NBEAL1 locus 124
Supplementary Figure 19 – Forest Plot for Association of rs9958650 at ZBTB14-EPB41L3 locus 125
Supplementary Figure 20 – Forest Plot for Association of rs12445022 at ZCCHC14 locus 126
Supplementary Figure 21 – Forest Plot for Association of rs2293576 at SPI1-SLC39A13-PSMC3-RAPSN locus 127
Supplementary Figure 22 – Forest Plot for Association of rs4621303 at ULK4 locus 128
Supplementary Figure 23 – Forest Plot for Association of rs2984613 at SLC25A44-PMF1-BGLAP locus 129
Supplementary Figure 24 – Forest Plot for Association of rs2303655 at LOX-LOC10058541-ZNF474 locus 130
Supplementary Figure 25 – Forest Plot for Association of rs12524544 at FOXF2-FOXQ1 locus 131
Supplementary Figure 26 – Forest Plot for Association of rs225744 at GPR126-VTA1 locus 132
Supplementary Figure 27 – Forest Plot for Association of rs79043147 at HTRA1/ARMS2 locus 133
Supplementary Figure 28 – Forest Plot for Association of rs11838776 at COL4A2 locus 134
Supplementary Figure 29 - Forest Plot for Association of rs61000833 at SH3PXD2A locus 135
Supplementary Figure 30 – Mendelian Randomization Results for Causal Impact of Body Mass Index on Lacunar Stroke 136
.... 136
.... 136
Supplementary Figure 31 – Mendelian Randomization Results for Causal Impact of Diastolic Blood Pressure on Lacunar Stroke 137
.... 137

Supplementary Figure 32 – Mendelian Randomization Results for Causal Impact of High Density Lipoprotein on Lacunar Stroke **138**
.... **138**
Supplementary Figure 33 – Mendelian Randomization Results for Causal Impact of Low Density Lipoprotein on Lacunar Stroke **139**
.... **139**
Supplementary Figure 34 – Mendelian Randomization Results for Causal Impact of Pulse Pressure on Lacunar Stroke **140**
.... **140**
Supplementary Figure 35 – Mendelian Randomization Results for Causal Impact of Systolic Blood Pressure on Lacunar Stroke **141**
.... **141**
Supplementary Figure 36 – Mendelian Randomization Results for Causal Impact of Ever Smoking on Lacunar Stroke **142**
.... **142**
Supplementary Figure 37 – Mendelian Randomization Results for Causal Impact of Triglycerides on Lacunar Stroke **143**
.... **143**
.... **143**
Supplementary Figure 38 – Mendelian Randomization Results for Causal Impact of Type 2 Diabetes on Lacunar Stroke **144**
UK DNA Lacunar 2 Stroke Study Investigators **145**
UK DNA Lacunar Stroke Study Investigators **147**
The Dutch Parelsnoer Institute-Cerebrovascular accident (CVA) Study Group, The Netherlands **148**
Members of the Stroke Genetics Network (SiGN) **149**
Membership of The International Stroke Genetics Consortium **153**

Supplementary Table 1 – Sample Characteristics

			Cases		Controls		
**Study**	**N cases**	**N controls**	**% Male**	**Mean Age (SD)**	**% Male**	**Mean Age (SD)**	**Genomic Inflation lambda**
MRI Confirmed							
DNA Lacunar	911	977	70	57 (10)	53	60 (4)	
SGUL Stroke Register, GENESIS, Cambridge BioResource	302		62	68 (12)			
DNA Lacunar 2	296		62	66 (12)			
Leuven Stroke Genetics Study	27		63	69 (12)			
Edinburgh Mild Stroke Study	34		58	66 (11)			1.037
PRESERVE	44		51	68 (9)			
OXVASC	37		59	63 (13)			
MGH	32		70	68 (15)			
Understanding Society		9194			44	NA*	
NINDS-SIGN (Graz, SAHLSIS, GEOS, SPS3, BASICMAR, MIAMISR, Leuven, Krakow, GCNKSS, GASROS, BRAINS)	610		68	63 (12)			
Milano	9		67	55 (16)			1.016
Australian Stroke Genetics Collaborative	23		57	66 (11)			
WTCCC2 (UK & Germany)	287		47	69 (11)			
WTCCC2, SIGN, ASGC		17554			49	62 (17)*	
SLESS, DNA LACUNAR 2 (African ancestry)	141	434	56	66 (10)	47	59 (12)	0.978
SIGN (African ancestry)	179	1190	54	58 (9)	38	60 (13)	1.018
DNA LACUNAR 2 (South Asian ancestry)	55		65	63 (13)			0.756
UK Biobank (South Asian ancestry)		191			62	59 (9)	
**Total (MRI Confirmed)**	**2987**	**29540**					
Standard Phenotyping							

Australian Stroke Genetics Collaborative	229	600	57	72 (13)	50	66 (8)	1.010
Barcelona	163	198	68	66 (13)	46	68 (7)	1.058
Germany-WTCCC2	69	402	71	67 (13)	51	NA*	1.019
NINDS-SIGN-group1 (BRAINS, MGH-GASROS, ISGS, SWISS, HABC)	116	793	54	72 (13)	54	74 (3)	1.018
NINDS-SIGN-group3,4,5,6,7,9 (GEOS, BRAINS, MGH-GASROS, GCNKSS, ISGS, MCISS, MIAMISR, NHS, NOMAS, REGARDS, SPS3, SWISS, WHI, WUSTL, HRS, OAI, Krakow, LSGS, BASICMAR, ADHD, INMA, SAHLSIS, LSR, MDC)	840	8043	53	67 (13)	46	61 (19)	1.036
Utrecht (Dutch Parelsnoer Study)	229		56	67 (12)			
NINDS-SIGN-group8 (Graz)	32	407	53	69 (13)	43	65 (8)	1.031
UK-WTCCC2	225	2584	38	69 (13)	50	NA*	1.009
WTCCC2-Immunochip	332	4594	55	70 (13)	42	NA*	1.099
Geisinger MyCode	196	624	46	67 (12)	46	66 (12)	1.004
deCODE	628	198602	62	68 (12)	50	60 (16)	1.015
Helsinki Ischemic Stroke Genetics Study	161	3367	57	65 (11)	44	61 (14)	1.007
Sahlgrenska Academy Study on Ischemic Stroke (SAHLSIS)	198	990	60	58 (8)	66	56 (11)	1.006
SLESS (African ancestry)	178	434	44	66 (10)	53	59 (12)	1.024
SIGN (African ancestry) (GEOS, MGH-GASROS, GCNKSS, ISGS, MCISS, MIAMISR, NOMAS, OAI, REGARDS, SPS3, SWISS, WHI, WUSTL, HRS)	200	1192	63	60 (13)	59	58 (13)	1.009
SIGN (Hispanic ancestry) (BRAINS, MGH-GASROS, GCNKSS, ISGS, MCISS, MIAMISR, NOMAS, NHS, REGARDS, SPS3, SWISS, WHI, WUSTL, HRS, HCHS/SOL, Krakow, LSGS, BASICMAR, SAHLSIS, LSR, MDC)	555	2428	NA	NA	NA	NA	0.998
**Total (not MRI Confirmed)**	**4351**	**225258**					
**Total**	**7338**	**254798**					

* age not available in WTCCC2 and Understanding Society controls

Supplementary Table 2 – Genotyping and Imputation

**Study**	**Genotyping platforms - SNP panel**	**Sample call rate**	**SNP call rate**	**MAF**	**HWE p-value**	**Imputation reference panel**
DNA Lacunar	Illumina HumanCoreExome	> 97%	> 97%	<0.01	< 10-6	1000 Genomes Phase 3 (ALL)
Stroke Register, GENESIS, Cambridge BioResource	Illumina HumanCoreExome	> 97%	> 97%	<0.01	< 10-6	1000 Genomes Phase 3 (ALL)
DNA Lacunar 2	Illumina HumanCoreExome	> 97%	> 97%	<0.01	< 10-6	1000 Genomes Phase 3 (ALL)
Leuven Stroke Genetics Study	Illumina HumanCoreExome	> 97%	> 97%	<0.01	< 10-6	1000 Genomes Phase 3 (ALL)
Edinburgh Mild Stroke Study	Illumina HumanCoreExome	> 97%	> 97%	<0.01	< 10-6	1000 Genomes Phase 3 (ALL)
PRESERVE	Illumina HumanCoreExome	> 97%	> 97%	<0.01	< 10-6	1000 Genomes Phase 3 (ALL)
OXVASC	Illumina HumanCoreExome	> 97%	> 97%	<0.01	< 10-6	1000 Genomes Phase 3 (ALL)
MGH	Illumina HumanCoreExome	> 97%	> 97%	<0.01	< 10-6	1000 Genomes Phase 3 (ALL)
Understanding Society	Illumina HumanCoreExome					1000 Genomes Phase 3 (ALL)
Milano	Illumina Human610-Quad v1_B and Human660W-Quad v1_A chips.	> 95%	> 95%	<0.01	< 10-5	Haplotype Reference Consortium
SLESS	Illumina Multi-Ethnic-Genotyping-Array	> 97%	> 97%	<0.005	< 10-6	Haplotype Reference Consortium
NINDS-SIGN -all studies	Illumina HumanOmni5Exome-4v1	>95%	> 99%	< 0.01	<10-3	Haplotype Reference Consortium
NINDS-SIGN-BRAINS	Illumina 650Q	>95%	> 99%	< 0.01	<10-3	Haplotype Reference Consortium
NINDS-SIGN-GASROS	Illumina610	>95%	> 99%	< 0.01	<10-3	Haplotype Reference Consortium
NINDS-SIGN-ISGS	Illumina610	>95%	> 99%	< 0.01	<10-3	Haplotype Reference Consortium
NINDS-SIGN-SWISS	Illumina610	>95%	> 99%	< 0.01	<10-3	Haplotype Reference Consortium
NINDS-SIGN-ESS	Illumina660	>95%	> 99%	< 0.01	<10-3	Haplotype Reference Consortium
NINDS-SIGN-MUNICH	Illumina660	>95%	> 99%	< 0.01	<10-3	Haplotype Reference Consortium
NINDS-SIGN-OXVASC	Illumina660	>95%	> 99%	< 0.01	<10-3	Haplotype Reference Consortium
NINDS-SIGN-STGEORGE	Illumina660	>95%	> 99%	< 0.01	<10-3	Haplotype Reference Consortium
NINDS-SIGN-KORA	Illumina550	>95%	> 99%	< 0.01	<10-3	Haplotype Reference Consortium
NINDS-SIGN-WTCCC	Illumina 660	>95%	> 99%	< 0.01	<10-3	Haplotype Reference Consortium
NINDS-SIGN-GEOS	Illumina 1M	>95%	> 99%	< 0.01	<10-3	Haplotype Reference Consortium
NINDS-SIGN-CIDR*	Illumina 5M	>95%	> 99%	< 0.01	<10-3	Haplotype Reference Consortium
NINDS-SIGN-OAI	Illumina 2.5M	>95%	> 99%	< 0.01	<10-3	Haplotype Reference Consortium
NINDS-SIGN-KRAKOW	Illumina 5M	>95%	> 99%	< 0.01	<10-3	Haplotype Reference Consortium
NINDS-SIGN-LSGS	Illumina 5M	>95%	> 99%	< 0.01	<10-3	Haplotype Reference Consortium
NINDS-SIGN-BASICMAR	Illumina 5M	>95%	> 99%	< 0.01	<10-3	Haplotype Reference Consortium
NINDS-SIGN-GRAZ	Illumina 5M	>95%	> 99%	< 0.01	<10-3	Haplotype Reference Consortium

NINDS-SIGN-SAHLSIS	Illumina 5M	>95%	> 99%	< 0.01	<10-3	Haplotype Reference Consortium
NINDS-SIGN-LSR	Illumina 5M	>95%	> 99%	< 0.01	<10-3	Haplotype Reference Consortium
NINDS-SIGN-MDC	Illumina 750K Express	>95%	> 99%	< 0.01	<10-3	Haplotype Reference Consortium
NINDS-SIGN-ASGC	Illumina 610	>95%	> 99%	< 0.01	<10-3	Haplotype Reference Consortium
Barcelona	IlluminaHumanCoreExome	>95%	> 95%	< 0.01	< 10-6	Haplotype Reference Consortium
Germany-WTCCC2	Illumina Human660W-Quad	>98%	>95%	<0.01	< 10-5	Haplotype Reference Consortium
UK-WTCCC2	Illumina Human660W-Quad	>98%	>95%	<0.01	< 10-5	Haplotype Reference Consortium
WTCCC2-Immunochip	Illumina Immunochip	> 95%	> 95%	<0.01	< 10-6	Haplotype Reference Consortium
Utrecht (Dutch Parelsnoer Study)	Illumina GSA	>98%	>99%	<10 minor alleles	<10-10 in cases; <10-5 in controls	Haplotype Reference Consortium
Geisinger MyCode	Illumina HumanOmniExpress and Illumina GSA	>90%	>90%	<0.01	<10-15	Haplotype Reference Consortium
DECODE	Illumina HumanHap and OmniExpress arrays	> 97%	> 95%	<0.01	0.001	WGS Icelanders
Helsinki Stroke Study	IlluminaHumanCoreExome	>95%	> 95%	<0.01	<10-6	Haplotype Reference Consortium
Sahlgrenska Academy Study on Ischemic Stroke (SAHLSIS)	Illumina Human Omni 5M array	> 97%	> 95%	= 0	<10-5	Haplotype Reference Consortium

Supplementary Table 2 – Instrumental Variables used in Mendelian Randomization Analysis

**BMI**				
SNP	A1	A2	Beta	SE
rs1000096	T	C	-0.0134	0.0018
rs1000940	A	G	-0.0115	0.0019
rs1003081	T	C	0.0105	0.0017
rs10035289	A	G	0.0114	0.0017
rs10037047	A	G	-0.0450	0.0030
rs1006353	A	G	0.0112	0.0019
rs10083803	T	C	-0.0112	0.0019
rs10110727	A	G	0.0118	0.0020
rs10118701	A	G	-0.0134	0.0019
rs10131890	A	C	-0.0252	0.0039
rs10132280	A	C	-0.0201	0.0019
rs1014194	A	C	0.0109	0.0018
rs1014312	C	G	-0.0229	0.0024
rs10146527	T	C	0.0130	0.0017
rs1015362	T	C	0.0196	0.0034
rs1015363	A	G	0.0342	0.0037
rs10168197	C	G	-0.0136	0.0021
rs10182181	A	G	-0.0277	0.0018
rs10190332	T	G	0.0133	0.0022
rs10203277	A	G	0.0200	0.0033
rs1020548	A	G	-0.0152	0.0023
rs10211055	T	C	-0.0140	0.0018
rs10268050	T	C	-0.0141	0.0021
rs10269783	A	G	0.0137	0.0017
rs1035010	T	C	0.0139	0.0020
rs1037587	T	C	0.0102	0.0018
rs1038088	T	G	-0.0128	0.0017
rs10408013	T	C	0.0133	0.0019

rs1040881	T	C	0.0110	0.0018
rs10438964	T	C	-0.0141	0.0020
rs1045411	T	C	-0.0153	0.0019
rs10456637	A	T	0.0431	0.0040
rs10459012	A	C	0.0277	0.0025
rs10460960	A	G	0.0187	0.0025
rs10467530	C	G	0.0191	0.0027
rs1048365	T	C	0.0145	0.0025
rs1048775	C	G	0.0115	0.0019
rs1048932	A	C	-0.0164	0.0018
rs10497807	C	G	-0.0184	0.0018
rs10499276	T	C	0.0150	0.0025
rs10500548	T	C	0.0218	0.0036
rs10510321	T	C	0.0124	0.0021
rs10511093	T	C	-0.0270	0.0041
rs10515050	T	C	-0.0104	0.0018
rs10519151	A	T	0.0307	0.0049
rs1057452	A	G	0.0170	0.0024
rs10733051	A	G	0.0097	0.0016
rs10744146	A	G	-0.0135	0.0017
rs10745785	T	C	-0.0113	0.0018
rs10754210	A	G	-0.0124	0.0018
rs1075901	T	C	-0.0127	0.0016
rs10769165	T	C	-0.0108	0.0018
rs10772055	C	G	-0.0174	0.0024
rs10773049	T	C	-0.0103	0.0017
rs10779751	A	G	0.0140	0.0018
rs10797115	T	C	0.0119	0.0017
rs10818810	A	G	0.0108	0.0018
rs10818938	A	G	0.0122	0.0017
rs10830452	A	G	-0.0116	0.0018

rs10832778	C	G	-0.0114	0.0017
rs10838122	T	C	-0.0104	0.0018
rs10838852	T	C	-0.0885	0.0045
rs10839472	T	C	0.0400	0.0028
rs10840606	A	G	-0.0174	0.0024
rs10840674	A	G	-0.0111	0.0018
rs10842240	C	G	0.0204	0.0028
rs10850031	T	G	-0.0445	0.0040
rs10865858	T	C	0.0106	0.0018
rs10867256	T	C	-0.0112	0.0017
rs10876418	T	C	-0.0189	0.0021
rs10878946	T	C	-0.0147	0.0019
rs10883553	A	C	0.0116	0.0018
rs10883759	A	G	-0.0120	0.0018
rs10886017	A	C	0.0131	0.0020
rs10887578	C	G	0.0125	0.0017
rs10915840	A	G	-0.0127	0.0020
rs10920678	A	G	0.0155	0.0017
rs10923724	T	C	-0.0114	0.0016
rs10929925	A	C	-0.0106	0.0017
rs10930641	A	G	-0.0156	0.0019
rs10935143	A	G	-0.0100	0.0017
rs10938397	A	G	-0.0328	0.0016
rs10953620	A	C	-0.0099	0.0017
rs10955841	A	G	0.0141	0.0019
rs10962549	T	C	0.0184	0.0024
rs10971712	T	C	-0.0181	0.0028
rs10989568	A	G	0.0129	0.0019
rs11030385	A	G	-0.0194	0.0022
rs11039014	A	G	0.0156	0.0022
rs11066301	A	G	0.0124	0.0019

rs1106908	A	G	-0.0156	0.0016
rs11074446	T	C	0.0248	0.0024
rs11075489	T	C	-0.0110	0.0018
rs11075986	C	G	0.0304	0.0047
rs11079849	T	C	-0.0149	0.0020
rs11115176	T	C	0.0171	0.0022
rs11118308	A	G	0.0101	0.0016
rs11128760	A	G	0.0115	0.0017
rs11150911	A	C	0.0139	0.0018
rs11172702	A	G	0.0320	0.0039
rs11190661	T	C	-0.0182	0.0025
rs11208662	C	G	0.0211	0.0031
rs11250076	A	G	0.0642	0.0032
rs11251352	A	G	-0.0118	0.0018
rs11259933	A	G	-0.0130	0.0018
rs11496125	T	C	0.0146	0.0018
rs11577179	A	G	-0.0129	0.0018
rs1158103	A	G	-0.0100	0.0017
rs11583122	T	C	0.0173	0.0030
rs11590474	T	C	-0.0361	0.0037
rs11600990	T	C	-0.0151	0.0025
rs11611246	T	G	0.0253	0.0021
rs11611496	A	G	-0.0150	0.0020
rs11629783	C	G	0.0150	0.0021
rs11635675	T	G	0.0171	0.0019
rs11642001	A	G	-0.0251	0.0031
rs11659764	A	T	-0.0305	0.0042
rs11668301	A	G	0.0175	0.0024
rs11672660	T	C	-0.0355	0.0022
rs11711337	A	T	-0.0117	0.0019
rs11736228	A	T	0.0122	0.0021

rs11753081	T	G	0.0133	0.0022
rs11756653	C	G	-0.0231	0.0039
rs11773362	T	C	-0.0112	0.0018
rs1178060	A	G	-0.1046	0.0065
rs11781222	T	C	0.0155	0.0024
rs11781699	T	C	-0.0142	0.0022
rs11783247	T	C	-0.0890	0.0035
rs11790280	T	C	-0.0110	0.0019
rs11792069	A	G	0.0151	0.0024
rs11844682	C	G	-0.0104	0.0018
rs11855853	T	C	-0.0205	0.0021
rs11866815	T	C	-0.0172	0.0019
rs11889536	A	G	0.0185	0.0025
rs11915371	A	C	-0.0145	0.0021
rs11929028	T	G	0.0787	0.0044
rs11945861	A	G	-0.0143	0.0020
rs11971041	A	G	-0.0191	0.0032
rs11987383	A	C	-0.0159	0.0026
rs12033257	A	G	0.0135	0.0018
rs12035149	C	G	-0.0158	0.0021
rs12035349	A	G	-0.0204	0.0029
rs12039524	A	G	-0.0487	0.0046
rs12041258	T	C	0.0148	0.0021
rs12042908	A	G	0.0170	0.0016
rs12042959	A	G	0.0183	0.0025
rs12044597	A	G	-0.0130	0.0017
rs1205106	A	G	-0.0100	0.0017
rs12098284	T	C	0.0158	0.0027
rs12101393	C	G	0.0161	0.0024
rs12147845	T	C	0.0201	0.0027
rs12148386	T	C	0.0476	0.0042

rs1218822	A	G	0.0151	0.0017
rs12189178	T	C	0.0277	0.0048
rs12207241	A	G	-0.0414	0.0037
rs12215331	T	C	0.0208	0.0023
rs1229057	T	C	0.0160	0.0028
rs12327272	A	G	0.0278	0.0034
rs12364470	T	G	-0.0171	0.0022
rs12369009	T	G	0.0614	0.0050
rs12411886	A	C	0.0339	0.0032
rs12417072	A	G	-0.0163	0.0027
rs1241986	A	G	-0.0149	0.0024
rs12420725	A	G	-0.0216	0.0037
rs12422552	C	G	-0.0143	0.0020
rs12429545	A	G	0.0337	0.0026
rs12439798	T	G	0.0129	0.0017
rs12443621	A	G	-0.0117	0.0017
rs12446632	A	G	-0.0335	0.0024
rs12448257	A	G	0.0222	0.0021
rs12448738	A	C	-0.0150	0.0025
rs12453418	A	G	-0.0109	0.0018
rs12458	A	T	-0.0252	0.0021
rs12479233	A	T	-0.0100	0.0017
rs12480713	T	C	0.0129	0.0020
rs12488237	T	C	-0.0233	0.0036
rs12514473	T	C	0.0162	0.0020
rs12564992	A	G	-0.0384	0.0036
rs12574668	A	C	0.0351	0.0036
rs12575252	C	G	-0.0157	0.0019
rs12577642	A	T	-0.0215	0.0018
rs12587412	T	G	0.0113	0.0018
rs12593036	A	G	0.0156	0.0020

rs12595158	T	C	-0.0399	0.0054
rs12595749	A	G	0.0150	0.0017
rs12602912	T	C	0.0182	0.0021
rs12609744	T	C	-0.0134	0.0020
rs12615778	A	G	0.0126	0.0020
rs12625413	T	C	-0.0112	0.0019
rs12631248	C	G	-0.0457	0.0036
rs12637576	T	C	0.0273	0.0037
rs12638746	A	G	-0.0258	0.0032
rs12655756	A	T	0.0124	0.0019
rs12659802	A	G	0.0149	0.0020
rs12666574	A	G	0.0130	0.0018
rs1266922	A	G	-0.0158	0.0023
rs12675063	A	T	-0.0157	0.0026
rs12680842	A	G	0.0114	0.0018
rs12682565	A	G	0.0344	0.0047
rs12694021	A	C	0.0103	0.0017
rs12705987	A	T	0.0211	0.0022
rs12713433	T	C	0.0147	0.0023
rs12731372	T	C	0.0121	0.0019
rs12776880	A	T	0.0117	0.0019
rs12885454	A	C	-0.0150	0.0018
rs12888545	A	G	-0.0171	0.0021
rs12902742	A	T	0.0211	0.0033
rs12920590	T	C	0.0376	0.0032
rs12922346	C	G	0.0121	0.0020
rs12939549	A	G	0.0163	0.0016
rs1296328	A	C	0.0149	0.0019
rs12964689	A	G	0.0228	0.0018
rs12991989	C	G	0.0111	0.0018
rs13021737	A	G	-0.0596	0.0024

rs13041173	A	G	-0.0561	0.0045
rs13045538	T	C	-0.0665	0.0066
rs13047416	C	G	0.0117	0.0019
rs13072095	T	C	0.0104	0.0017
rs13085472	T	C	0.0110	0.0018
rs13095652	T	C	-0.0133	0.0021
rs13107325	T	C	0.0449	0.0033
rs13110266	A	G	-0.0114	0.0018
rs13163306	A	G	-0.0108	0.0018
rs13174863	A	G	-0.0197	0.0024
rs13175892	T	C	0.0998	0.0075
rs13191362	A	G	0.0206	0.0027
rs13201877	A	G	-0.0146	0.0024
rs13203153	A	G	-0.0156	0.0023
rs13203286	T	G	0.0334	0.0043
rs13207082	A	G	-0.1012	0.0061
rs1320903	A	G	0.0160	0.0022
rs13209753	A	G	-0.0221	0.0037
rs13209872	C	G	-0.0127	0.0019
rs13209968	C	G	0.0119	0.0017
rs13227433	T	G	-0.0145	0.0021
rs13227658	T	C	-0.0206	0.0018
rs1323068	A	G	-0.0130	0.0019
rs13240600	A	G	0.0215	0.0025
rs1324110	C	G	-0.0115	0.0018
rs13247665	T	C	-0.0120	0.0019
rs13263601	A	C	-0.0121	0.0018
rs13290794	A	G	-0.0130	0.0019
rs13292976	T	C	0.0108	0.0018
rs13321566	A	G	-0.0155	0.0023
rs13329567	T	C	-0.0354	0.0023

rs1333423	A	T	0.0148	0.0022
rs13417156	T	C	-0.0135	0.0017
rs13432055	T	C	-0.0133	0.0018
rs1345148	T	C	-0.0101	0.0017
rs1358808	C	G	-0.0305	0.0022
rs1362910	A	G	0.0154	0.0019
rs1365466	T	C	-0.0146	0.0019
rs1371108	A	C	0.0157	0.0019
rs1375561	T	C	0.0131	0.0020
rs1394	A	G	-0.0488	0.0023
rs1399054	A	G	0.0218	0.0027
rs1403846	T	C	0.0121	0.0021
rs1405348	A	G	-0.0135	0.0020
rs1412235	C	G	0.0325	0.0021
rs1423627	T	C	-0.0642	0.0038
rs1431659	A	G	0.0204	0.0019
rs143384	A	G	0.0153	0.0020
rs1436343	A	G	-0.0123	0.0018
rs1437929	A	G	0.0119	0.0019
rs1451533	A	G	0.0144	0.0020
rs1452075	T	C	0.0143	0.0018
rs1452134	T	C	-0.0102	0.0017
rs1454148	T	C	0.0115	0.0019
rs1454687	C	G	0.0197	0.0017
rs1455137	A	C	-0.0110	0.0018
rs1473579	A	G	-0.0858	0.0047
rs1475774	A	G	0.0751	0.0074
rs1477887	A	G	-0.0128	0.0018
rs1492767	T	C	0.0097	0.0016
rs1498139	A	C	0.0131	0.0021
rs1503139	A	G	-0.0134	0.0021

rs150353	T	G	-0.0112	0.0018
rs1512065	A	G	0.0145	0.0021
rs1522569	T	G	0.0148	0.0022
rs1544459	T	C	-0.0104	0.0016
rs1549293	T	C	-0.0175	0.0019
rs1552717	A	T	0.0175	0.0026
rs1554193	A	T	-0.0105	0.0017
rs1558236	C	G	0.0560	0.0072
rs156151	C	G	-0.0146	0.0022
rs1564981	A	G	0.0113	0.0017
rs159032	T	C	0.0123	0.0020
rs1593304	A	G	0.0128	0.0022
rs1594830	C	G	-0.0125	0.0021
rs1600136	A	C	-0.0320	0.0030
rs160401	T	C	0.0214	0.0030
rs1608445	A	G	-0.0104	0.0018
rs1625427	T	C	0.0134	0.0018
rs1650586	T	G	0.0180	0.0029
rs1657930	A	G	-0.0173	0.0024
rs1658820	T	G	0.0146	0.0021
rs1668633	T	C	0.0109	0.0017
rs16823670	A	G	0.0309	0.0052
rs16828086	C	G	0.0106	0.0018
rs16851483	T	G	0.0339	0.0039
rs16906845	A	G	-0.0232	0.0038
rs16907751	T	C	-0.0201	0.0030
rs16932761	A	G	-0.0139	0.0020
rs16951319	T	C	-0.0165	0.0027
rs16952479	A	T	-0.0357	0.0045
rs16965062	T	C	0.0099	0.0017
rs16966801	A	G	-0.0143	0.0022

rs1700137	T	C	-0.0134	0.0019
rs17014375	T	G	-0.0162	0.0025
rs17035438	A	G	0.0191	0.0032
rs17056301	T	C	-0.0173	0.0021
rs17069831	T	C	-0.0125	0.0020
rs17080319	T	C	0.0569	0.0039
rs17091470	T	G	-0.0512	0.0050
rs17094222	T	C	-0.0275	0.0027
rs17096549	A	G	0.0533	0.0063
rs17096552	A	G	-0.0203	0.0029
rs17105272	T	C	0.0114	0.0019
rs1712517	T	C	-0.0118	0.0019
rs17197116	T	C	0.0348	0.0048
rs17199978	A	G	0.0217	0.0033
rs17203016	A	G	-0.0122	0.0020
rs17207196	T	C	-0.0184	0.0020
rs17238110	A	G	0.0352	0.0050
rs17285919	T	C	0.1203	0.0064
rs1730859	A	G	-0.0112	0.0017
rs17327461	T	C	0.0119	0.0016
rs17351791	A	C	0.0109	0.0018
rs17391694	T	C	0.0293	0.0026
rs174415	A	T	0.0129	0.0022
rs17446091	T	C	-0.0122	0.0021
rs17448682	T	C	0.0131	0.0021
rs17522122	T	G	0.0164	0.0017
rs17525725	A	G	-0.0102	0.0018
rs17531363	A	C	0.0127	0.0020
rs17538472	T	C	0.0129	0.0022
rs17551974	A	C	-0.0127	0.0022
rs17591778	A	G	0.0148	0.0023

rs17608150	T	C	0.0193	0.0033
rs17636031	T	C	-0.0169	0.0021
rs17681451	A	G	-0.0268	0.0034
rs17681708	T	C	-0.0104	0.0018
rs17724992	A	G	0.0180	0.0019
rs17739298	C	G	0.0351	0.0032
rs17757975	T	C	0.0160	0.0025
rs17795934	T	C	-0.0114	0.0018
rs17806379	T	C	-0.0248	0.0023
rs17820822	T	G	0.0113	0.0019
rs1782507	T	G	-0.0130	0.0020
rs1787267	C	G	-0.0241	0.0036
rs1789165	A	G	0.0125	0.0017
rs1791253	T	G	0.0221	0.0037
rs1814170	A	T	0.0207	0.0029
rs181732	T	G	-0.0301	0.0028
rs1819844	A	G	0.0141	0.0021
rs1852006	A	G	-0.0133	0.0021
rs1853639	A	G	-0.0121	0.0018
rs1862451	A	G	0.0156	0.0020
rs1865341	T	C	0.0130	0.0020
rs186543	C	G	-0.0349	0.0023
rs1866510	T	C	-0.0108	0.0018
rs1884389	T	C	-0.0109	0.0017
rs1884897	A	G	-0.0191	0.0017
rs1899689	T	C	0.0121	0.0016
rs1899951	T	C	0.0155	0.0025
rs1903579	C	G	0.0125	0.0017
rs1909586	T	G	-0.0111	0.0018
rs1927790	T	C	-0.0127	0.0017
rs1928295	T	C	0.0152	0.0016

rs1937684	A	T	0.0112	0.0018
rs1941697	A	G	0.0137	0.0017
rs1942866	C	G	0.0187	0.0031
rs1943477	T	C	-0.0223	0.0036
rs1951455	T	C	-0.0136	0.0019
rs1956151	A	G	-0.0156	0.0023
rs1973993	T	C	-0.0204	0.0018
rs1982441	T	G	0.0155	0.0027
rs1983864	T	G	0.0119	0.0019
rs2000746	A	G	0.0153	0.0021
rs2003616	T	G	0.0125	0.0020
rs200968	T	C	0.0790	0.0050
rs2010281	A	G	-0.0109	0.0019
rs2012927	A	G	0.0106	0.0018
rs2030342	T	C	0.0169	0.0018
rs2047632	C	G	-0.0379	0.0059
rs2075205	A	T	0.0131	0.0017
rs2075650	A	G	0.0224	0.0023
rs208015	T	C	0.0311	0.0035
rs2080454	A	C	-0.0112	0.0018
rs2098618	A	T	0.0500	0.0042
rs2100814	A	G	0.0116	0.0017
rs2122042	T	G	0.0242	0.0023
rs2124499	C	G	-0.0126	0.0017
rs2140418	T	C	-0.0429	0.0034
rs2143253	A	G	-0.0189	0.0026
rs2154297	T	C	-0.0111	0.0019
rs215632	A	G	0.0115	0.0020
rs2160077	A	G	0.0121	0.0017
rs2163188	C	G	0.0105	0.0018
rs2170382	T	C	0.0168	0.0027

rs2174307	C	G	0.0114	0.0017
rs217671	A	G	-0.0126	0.0019
rs2185027	A	C	-0.0132	0.0018
rs2190788	T	G	0.0181	0.0020
rs2192158	A	G	0.0150	0.0017
rs2194385	A	C	0.0101	0.0017
rs2228213	A	G	-0.0140	0.0017
rs223051	T	C	0.0105	0.0018
rs2230590	T	C	-0.0562	0.0023
rs2230929	A	G	0.0474	0.0034
rs2236950	A	C	-0.0190	0.0027
rs2240108	T	C	-0.0159	0.0026
rs2275426	A	G	0.0157	0.0020
rs2282231	T	C	0.0134	0.0021
rs2283093	T	C	0.0126	0.0021
rs2293605	T	C	-0.0164	0.0028
rs2295896	A	G	-0.0149	0.0025
rs2304130	A	G	0.0214	0.0035
rs2304607	A	G	-0.0493	0.0028
rs2307022	A	G	0.0513	0.0033
rs2307111	T	C	0.0218	0.0017
rs2356865	T	C	-0.0122	0.0020
rs2357760	A	G	0.0107	0.0018
rs2365389	T	C	-0.0132	0.0019
rs2367112	T	G	0.0115	0.0017
rs2386802	A	C	-0.0254	0.0029
rs2397061	T	C	-0.0386	0.0037
rs2409730	A	C	0.1059	0.0038
rs2412107	T	G	0.0136	0.0021
rs2425241	T	C	-0.0243	0.0035
rs2425857	A	G	0.0138	0.0017

rs2429150	A	C	-0.0125	0.0018
rs2440885	A	G	0.0124	0.0019
rs2459359	C	G	0.0658	0.0061
rs2467110	T	C	-0.0141	0.0022
rs2479958	A	G	0.0130	0.0018
rs248139	A	G	0.0160	0.0023
rs2481665	T	C	0.0152	0.0017
rs2491864	A	G	0.0133	0.0021
rs2504674	C	G	-0.0146	0.0019
rs2516739	A	G	-0.0129	0.0022
rs2534760	A	T	-0.0144	0.0021
rs2537847	A	G	-0.0119	0.0020
rs2543132	C	G	0.0175	0.0023
rs254428	T	G	0.0102	0.0017
rs25832	A	G	0.0148	0.0020
rs2590942	T	G	0.0207	0.0022
rs2593280	A	G	0.0154	0.0025
rs2605603	A	G	-0.0102	0.0016
rs2611742	T	C	-0.0194	0.0021
rs2619976	T	C	0.0107	0.0018
rs262956	T	G	0.0109	0.0019
rs2631681	T	C	-0.0106	0.0017
rs2634047	C	G	-0.0135	0.0020
rs2635727	T	C	-0.0506	0.0034
rs2653365	T	C	-0.0177	0.0028
rs2680648	T	C	0.0144	0.0021
rs2710323	T	C	-0.0153	0.0017
rs2721965	A	C	0.0143	0.0019
rs2724861	A	G	-0.0153	0.0021
rs2731222	A	C	0.0120	0.0019
rs2733287	A	C	-0.0162	0.0017

rs273504	A	G	-0.0131	0.0018
rs273697	A	G	-0.0107	0.0017
rs2768950	A	G	0.0116	0.0019
rs2781668	T	C	0.0194	0.0023
rs2814992	A	G	-0.0519	0.0032
rs2820311	A	G	-0.0180	0.0022
rs28350	A	G	0.0139	0.0024
rs2836961	A	C	-0.0121	0.0018
rs2838006	T	C	-0.0127	0.0018
rs284227	T	C	-0.0152	0.0019
rs2842385	A	G	-0.0152	0.0023
rs2850969	T	C	-0.0144	0.0024
rs2863981	A	G	-0.0414	0.0035
rs2866816	T	C	0.0124	0.0021
rs2875762	C	G	0.0124	0.0020
rs2890652	T	C	-0.0169	0.0023
rs2902021	T	C	0.0232	0.0035
rs293566	T	C	-0.0162	0.0024
rs2974255	A	G	0.0237	0.0026
rs29938	T	C	-0.0133	0.0018
rs310618	T	C	-0.0119	0.0018
rs3134353	A	T	-0.0120	0.0018
rs3134438	A	C	0.0121	0.0019
rs316611	T	C	0.0127	0.0021
rs326889	T	C	-0.0124	0.0019
rs329124	A	G	0.0133	0.0017
rs329277	T	G	-0.0099	0.0017
rs329651	T	G	0.0174	0.0022
rs331949	T	C	-0.0124	0.0018
rs340025	T	C	-0.0121	0.0017
rs346722	T	C	0.0251	0.0038

rs355777	C	G	0.0131	0.0018
rs3734572	T	C	-0.0308	0.0043
rs3736485	A	G	0.0116	0.0016
rs3739555	T	G	0.0137	0.0024
rs3739733	A	G	0.0130	0.0021
rs3746429	T	C	0.0666	0.0046
rs3751813	T	G	0.0175	0.0025
rs3753549	T	C	-0.0229	0.0026
rs3762396	A	G	0.0110	0.0019
rs3768486	A	G	-0.0125	0.0021
rs3772934	T	C	0.0110	0.0019
rs3781099	T	C	0.0204	0.0031
rs3794702	A	T	0.0137	0.0024
rs3800229	T	G	0.0154	0.0019
rs3800649	A	G	0.0111	0.0019
rs3802924	A	C	0.0138	0.0023
rs3803286	A	G	0.0168	0.0018
rs3807049	T	C	0.0399	0.0033
rs3807566	T	G	-0.0122	0.0017
rs3808434	A	G	0.0105	0.0016
rs3809272	A	G	0.0201	0.0023
rs3810291	A	G	0.0294	0.0021
rs3814883	T	C	0.0234	0.0017
rs3819299	T	G	0.0213	0.0035
rs3821841	T	C	-0.0225	0.0034
rs3822683	A	G	0.0125	0.0021
rs3826705	T	C	-0.0157	0.0027
rs3829849	T	C	0.0120	0.0018
rs3844598	A	G	-0.0120	0.0018
rs3849570	A	C	0.0160	0.0018
rs3850422	A	G	-0.0112	0.0016

rs3851083	A	G	-0.0093	0.0016
rs3887080	A	G	0.0180	0.0027
rs3902840	A	G	0.0254	0.0032
rs3902951	T	G	-0.0120	0.0020
rs3915844	A	G	0.0197	0.0027
rs3922853	A	C	0.0144	0.0023
rs39654	A	G	-0.0154	0.0017
rs40067	A	G	-0.0230	0.0023
rs40245	A	T	0.0117	0.0018
rs403656	A	G	0.0720	0.0056
rs4076358	A	G	0.0104	0.0017
rs4077093	T	G	0.0131	0.0022
rs411717	T	C	-0.0106	0.0018
rs4148155	A	G	0.0189	0.0026
rs419261	T	C	0.0101	0.0018
rs4273371	T	C	-0.0112	0.0017
rs4278019	A	T	0.0114	0.0020
rs427943	A	C	-0.0166	0.0018
rs4284600	T	C	-0.0100	0.0017
rs4303732	T	C	0.0137	0.0018
rs4307239	A	G	-0.0106	0.0017
rs4339513	T	C	0.0449	0.0051
rs4342060	T	C	-0.0197	0.0026
rs4366093	T	C	-0.0109	0.0018
rs4372296	A	C	-0.0191	0.0022
rs4372836	T	C	0.0150	0.0018
rs4383818	T	G	0.0247	0.0038
rs450231	A	G	-0.0142	0.0020
rs4515655	T	C	-0.0113	0.0019
rs4516268	A	C	-0.0190	0.0021
rs4518345	A	G	-0.0119	0.0019

rs4524456	A	G	-0.0106	0.0018
rs453520	T	C	-0.0154	0.0017
rs4542429	T	C	-0.0286	0.0028
rs4624596	T	C	0.0140	0.0023
rs4639527	A	G	-0.0161	0.0020
rs4643949	T	C	0.0224	0.0031
rs4653017	T	C	0.0107	0.0018
rs4670626	T	C	-0.0120	0.0018
rs4671328	T	G	0.0176	0.0020
rs4671358	A	T	0.0146	0.0020
rs4673553	T	G	-0.0130	0.0017
rs4676084	A	G	0.0107	0.0017
rs4678297	T	C	0.0243	0.0027
rs4718966	T	C	0.0119	0.0019
rs4722398	T	C	0.0182	0.0026
rs4722672	T	C	-0.0127	0.0021
rs4725984	T	C	-0.0125	0.0017
rs4737183	A	G	0.0115	0.0017
rs4740383	A	G	0.0118	0.0019
rs4744275	A	G	0.0114	0.0019
rs4759073	A	G	-0.0116	0.0017
rs4759228	C	G	-0.0145	0.0019
rs4764949	A	G	0.0193	0.0019
rs4766710	A	G	0.0228	0.0036
rs4783241	C	G	-0.0105	0.0017
rs4796243	A	G	-0.0125	0.0019
rs4814512	A	C	0.0156	0.0021
rs4820408	T	G	0.0196	0.0019
rs4841659	T	C	0.1181	0.0038
rs4854326	A	G	0.0201	0.0021
rs4858193	T	C	0.0125	0.0019

rs4864201	T	C	0.0128	0.0017
rs487060	T	C	0.0106	0.0016
rs4886506	T	G	0.0399	0.0040
rs4886869	A	G	-0.0290	0.0024
rs4889782	T	C	-0.0122	0.0018
rs4906908	T	G	-0.0103	0.0017
rs4916661	T	G	-0.0256	0.0022
rs4936175	T	C	-0.0130	0.0017
rs4936671	C	G	0.0120	0.0018
rs4939051	C	G	0.0665	0.0058
rs4969387	C	G	-0.0172	0.0022
rs497417	A	T	0.0728	0.0031
rs4981693	A	G	0.0161	0.0022
rs498240	A	G	-0.0246	0.0035
rs4985155	A	G	0.0120	0.0019
rs4986044	T	C	-0.0159	0.0016
rs524281	A	C	-0.0146	0.0020
rs5396	T	C	-0.0152	0.0018
rs543874	A	G	-0.0475	0.0021
rs551137	T	C	0.0596	0.0050
rs555267	T	G	0.0112	0.0018
rs559267	A	G	-0.0125	0.0017
rs573455	A	G	-0.0117	0.0017
rs5750913	A	G	-0.0126	0.0022
rs577525	T	C	-0.0132	0.0018
rs580438	T	C	0.0118	0.0018
rs587230	A	G	0.0180	0.0024
rs6011457	A	T	-0.0117	0.0017
rs6019482	T	C	-0.0185	0.0023
rs6058635	C	G	0.0267	0.0031
rs6060151	T	G	-0.0619	0.0043

rs6061162	T	C	0.0498	0.0053
rs6076348	A	G	-0.0226	0.0030
rs6088529	A	C	0.0488	0.0036
rs6088943	A	T	0.0254	0.0039
rs6121381	A	T	-0.0256	0.0028
rs6132918	T	C	-0.0231	0.0029
rs6138482	T	C	0.0199	0.0022
rs6142096	A	G	0.0196	0.0029
rs621042	A	C	-0.0109	0.0017
rs6235	C	G	-0.0141	0.0020
rs6265	T	C	-0.0356	0.0022
rs629443	T	G	0.0111	0.0019
rs6442021	T	C	-0.0110	0.0017
rs6442101	T	C	-0.0194	0.0018
rs6448587	A	C	0.0151	0.0024
rs6449531	A	G	-0.0155	0.0019
rs6459326	C	G	-0.0219	0.0038
rs6461115	A	G	0.0146	0.0019
rs6463489	T	C	0.0153	0.0026
rs6474945	T	G	-0.0171	0.0016
rs6477694	T	C	-0.0123	0.0018
rs6490055	A	G	-0.0530	0.0040
rs6494481	T	G	0.0221	0.0033
rs6495252	T	C	0.0134	0.0019
rs651548	A	G	0.0125	0.0020
rs6545714	A	G	-0.0199	0.0017
rs6548221	A	G	0.0153	0.0020
rs6551410	A	T	-0.0493	0.0044
rs6564360	A	G	-0.0130	0.0022
rs6569648	T	C	-0.0127	0.0019
rs6577584	T	G	-0.0120	0.0018

rs6580755	T	C	-0.0327	0.0038
rs6587552	A	G	0.0150	0.0021
rs6595205	C	G	0.0116	0.0016
rs6606686	C	G	-0.0144	0.0019
rs663129	A	G	0.0327	0.0028
rs6681627	A	C	-0.0574	0.0053
rs6690764	A	G	-0.0154	0.0022
rs6700816	A	T	0.0328	0.0052
rs6700838	T	C	-0.0188	0.0018
rs6710871	A	G	0.0156	0.0024
rs6711584	A	G	0.0195	0.0020
rs6713781	C	G	-0.0108	0.0019
rs6720868	T	C	0.0142	0.0018
rs6738445	T	C	-0.0166	0.0021
rs6753170	T	C	0.0137	0.0021
rs6764533	A	G	0.0110	0.0018
rs676749	A	T	-0.0126	0.0018
rs6767619	C	G	0.0121	0.0018
rs6786125	C	G	-0.0158	0.0023
rs6786582	T	C	-0.0698	0.0044
rs6803870	T	C	-0.0330	0.0041
rs6804181	A	T	0.0144	0.0024
rs6804842	A	G	-0.0157	0.0018
rs6818414	T	C	-0.0105	0.0017
rs6827083	A	G	-0.0124	0.0018
rs6843738	A	G	-0.0114	0.0017
rs6879326	T	C	-0.0100	0.0017
rs6888159	C	G	0.0142	0.0019
rs6900723	T	C	-0.0121	0.0018
rs6908295	A	C	-0.0223	0.0028
rs6919443	A	G	-0.0101	0.0018

rs6921533	T	C	0.0129	0.0019
rs6922214	A	G	-0.0148	0.0025
rs6922855	A	G	0.0196	0.0024
rs6938239	A	G	0.0292	0.0038
rs6963840	T	C	0.0159	0.0024
rs696606	A	G	0.0117	0.0018
rs6968554	A	G	-0.0100	0.0017
rs7006629	T	C	0.0123	0.0018
rs7024334	T	G	0.0124	0.0020
rs7042372	A	G	0.0128	0.0018
rs7083450	T	C	0.0190	0.0029
rs7084454	A	G	0.0200	0.0019
rs7102454	T	C	-0.0125	0.0019
rs710355	C	G	-0.0266	0.0040
rs711347	A	T	-0.0122	0.0020
rs7117238	A	G	-0.0143	0.0022
rs7120873	T	C	0.0540	0.0037
rs7122539	A	G	-0.0130	0.0019
rs7123876	T	C	-0.0114	0.0019
rs7124442	T	C	-0.0205	0.0018
rs7124681	A	C	0.0411	0.0021
rs7131262	A	T	-0.0221	0.0022
rs7133378	A	G	0.0108	0.0018
rs7134375	A	C	0.0120	0.0019
rs7138803	A	G	0.0287	0.0021
rs7144011	T	G	0.0297	0.0024
rs7147503	T	C	-0.0123	0.0018
rs7164727	T	C	0.0175	0.0017
rs7181498	T	C	0.0155	0.0018
rs7181610	A	T	0.0148	0.0025
rs7187776	A	G	-0.0269	0.0016

rs7195386	T	C	0.0153	0.0018
rs719802	T	C	0.0109	0.0018
rs7200919	A	G	0.0546	0.0037
rs7206608	C	G	-0.0125	0.0019
rs7209235	A	G	-0.0118	0.0019
rs7211567	T	C	-0.0154	0.0021
rs7217226	T	G	-0.0113	0.0018
rs7239114	A	G	0.0121	0.0017
rs7243357	T	G	0.0212	0.0021
rs7313924	C	G	0.0118	0.0019
rs7334078	T	C	0.0129	0.0019
rs733594	T	C	0.0136	0.0018
rs7377083	A	C	0.0159	0.0018
rs740157	A	G	0.0106	0.0016
rs7425440	T	C	0.0214	0.0025
rs7444298	A	G	0.1244	0.0050
rs7478904	T	C	-0.0517	0.0043
rs753010	C	G	-0.0438	0.0059
rs7531118	T	C	-0.0221	0.0018
rs7531656	A	G	0.0194	0.0018
rs7535528	A	G	-0.0146	0.0018
rs7550711	T	C	0.0636	0.0051
rs7556169	A	G	-0.0120	0.0018
rs7557796	T	C	0.0176	0.0018
rs7560871	A	G	0.0203	0.0034
rs7561278	T	C	0.0160	0.0021
rs7564679	A	G	-0.0120	0.0016
rs756717	A	G	-0.0150	0.0019
rs757608	A	G	-0.0100	0.0017
rs7598402	C	G	0.0145	0.0018
rs7599312	A	G	-0.0178	0.0020

rs7600699	C	G	-0.0141	0.0024
rs7601895	C	G	0.0174	0.0021
rs7607351	T	C	0.0121	0.0017
rs7607369	A	G	0.0110	0.0016
rs7607490	A	G	0.0178	0.0029
rs761423	T	C	0.0123	0.0017
rs7615297	C	G	0.0143	0.0024
rs7616371	A	G	-0.0213	0.0037
rs762147	A	G	-0.0122	0.0019
rs7640424	T	C	-0.0125	0.0018
rs765332	T	G	-0.0246	0.0033
rs7674623	T	C	0.0150	0.0022
rs7683836	A	G	-0.0116	0.0017
rs7692088	C	G	0.0103	0.0018
rs7694732	A	G	0.0115	0.0018
rs769674	A	T	0.0120	0.0018
rs7702514	T	C	-0.1243	0.0061
rs7710595	A	C	0.0115	0.0017
rs7711753	A	G	-0.0144	0.0016
rs7715256	T	G	-0.0150	0.0017
rs7716275	T	G	-0.0150	0.0021
rs7727781	T	C	0.0099	0.0017
rs7730004	T	C	0.0118	0.0019
rs7730898	A	G	0.0129	0.0019
rs7748777	A	G	0.0093	0.0016
rs775731	T	C	-0.0107	0.0017
rs7760082	A	G	-0.0125	0.0018
rs7777102	A	G	-0.0141	0.0024
rs7779498	T	C	-0.0328	0.0046
rs7784465	T	C	-0.0175	0.0025
rs7796608	A	G	0.0154	0.0026

rs7805441	T	C	0.0127	0.0018
rs7827182	C	G	0.0155	0.0021
rs7832003	C	G	-0.0224	0.0027
rs7844647	T	C	0.0126	0.0018
rs785278	A	T	-0.0148	0.0023
rs7865157	T	C	0.0175	0.0028
rs7869771	A	C	0.0133	0.0019
rs7871866	C	G	0.0150	0.0025
rs7874154	T	C	-0.0119	0.0018
rs7893571	T	G	0.0136	0.0018
rs7899106	A	G	-0.0317	0.0037
rs7903146	T	C	-0.0131	0.0020
rs7919	A	C	-0.0121	0.0018
rs7924371	T	C	-0.0498	0.0036
rs793520	A	G	0.0124	0.0019
rs7941030	T	C	-0.0111	0.0017
rs7948120	T	C	-0.0132	0.0021
rs7958206	A	G	0.0182	0.0030
rs7965658	A	G	-0.0140	0.0023
rs7968230	A	G	0.0120	0.0018
rs7973955	A	G	-0.0116	0.0019
rs7975187	A	G	-0.0137	0.0021
rs799132	A	T	-0.0124	0.0021
rs799449	T	C	0.0136	0.0017
rs8016771	T	G	-0.0217	0.0031
rs8016859	C	G	0.0332	0.0044
rs8024932	T	G	0.0213	0.0031
rs8033510	T	C	0.0116	0.0018
rs8036040	A	C	0.0109	0.0017
rs8046061	T	C	0.0103	0.0017
rs8047395	A	G	0.0410	0.0036

rs8069296	T	C	-0.0129	0.0021
rs8070454	T	C	-0.0101	0.0018
rs8071182	A	G	0.0134	0.0022
rs8075273	A	C	-0.0114	0.0019
rs8079034	T	C	0.0140	0.0024
rs8081039	T	C	0.0241	0.0038
rs8087550	A	C	0.0127	0.0021
rs8089514	A	T	0.0138	0.0020
rs8092503	A	G	-0.0136	0.0020
rs8094523	A	G	-0.0440	0.0039
rs8097544	A	G	-0.0211	0.0025
rs8102137	T	C	-0.0187	0.0018
rs8121840	A	G	0.0105	0.0018
rs8123881	A	G	-0.0222	0.0025
rs816364	A	G	-0.0111	0.0019
rs816533	A	G	-0.0308	0.0050
rs820077	A	G	0.0279	0.0029
rs823074	T	C	0.0118	0.0017
rs825680	A	T	0.0107	0.0018
rs827092	T	C	0.0121	0.0017
rs845084	A	G	0.0129	0.0020
rs847747	T	G	-0.0113	0.0020
rs849135	A	G	0.0117	0.0016
rs853679	A	C	0.0728	0.0042
rs8567	A	G	-0.0099	0.0016
rs868554	C	G	-0.0172	0.0020
rs874454	A	G	-0.0139	0.0021
rs879620	T	C	0.0156	0.0023
rs881301	T	C	-0.0101	0.0017
rs884282	T	C	-0.0108	0.0018
rs886444	A	G	-0.0130	0.0019

rs889398	T	C	-0.0182	0.0017
rs892261	T	C	-0.0122	0.0020
rs895330	C	G	0.0199	0.0023
rs900144	T	C	0.0161	0.0017
rs901630	T	C	-0.0103	0.0018
rs902695	A	G	-0.0136	0.0018
rs903959	A	T	0.0107	0.0018
rs905938	T	C	-0.0161	0.0020
rs907011	T	G	0.0115	0.0019
rs9077	A	G	-0.0158	0.0021
rs912768	C	G	0.0421	0.0036
rs919433	A	G	0.0142	0.0021
rs925018	C	G	-0.0115	0.0018
rs925421	A	G	0.0132	0.0021
rs9289499	T	C	0.0258	0.0033
rs9291467	T	C	0.0129	0.0017
rs9294260	A	G	0.0132	0.0017
rs929641	A	G	0.0143	0.0020
rs9299	T	C	0.0147	0.0019
rs930295	A	C	0.0166	0.0024
rs9332817	C	G	-0.0347	0.0060
rs934515	A	G	0.0178	0.0027
rs9349239	A	G	0.0106	0.0018
rs9362662	A	G	0.0112	0.0019
rs9367368	T	C	0.0138	0.0018
rs9370042	C	G	-0.0183	0.0029
rs9394312	C	G	0.0483	0.0034
rs9396763	A	C	0.0135	0.0023
rs9419958	T	C	-0.0154	0.0026
rs9426003	A	G	-0.0112	0.0019
rs9460306	T	C	0.0192	0.0032

rs9463175	T	C	-0.0115	0.0017
rs946526	T	C	-0.0319	0.0047
rs9475173	A	G	0.0119	0.0020
rs9507983	T	C	-0.0133	0.0021
rs952159	A	G	0.0180	0.0027
rs9527706	A	G	-0.0115	0.0020
rs9530843	A	C	0.0140	0.0018
rs9538141	A	G	0.0134	0.0019
rs954018	A	G	-0.0140	0.0018
rs9540493	A	G	0.0169	0.0018
rs9544915	T	C	0.0154	0.0025
rs9554263	C	G	-0.0122	0.0021
rs9571687	A	C	-0.0174	0.0020
rs9595908	T	C	0.0158	0.0017
rs9603697	T	C	0.0148	0.0018
rs9615905	T	C	0.0103	0.0017
rs9630985	A	C	-0.0130	0.0019
rs9657542	C	G	0.1020	0.0036
rs9675376	A	G	0.0215	0.0022
rs967605	T	C	-0.0173	0.0024
rs9688431	T	C	0.0222	0.0035
rs968972	A	G	0.0108	0.0018
rs972283	A	G	0.0096	0.0016
rs972540	A	G	-0.0117	0.0018
rs977540	A	G	0.0120	0.0019
rs9787495	A	G	-0.0112	0.0019
rs980329	T	C	-0.0171	0.0021
rs9806058	A	T	0.0150	0.0026
rs9814633	A	G	0.0129	0.0018
rs9816226	A	T	-0.0235	0.0027
rs9827072	A	G	0.1008	0.0055

rs9832305	T	C	-0.0228	0.0029
rs9838283	A	G	0.1109	0.0049
rs9846123	T	C	0.0494	0.0038
rs984902	A	T	0.0188	0.0029
rs987237	A	G	-0.0393	0.0027
rs9881036	A	G	0.0369	0.0034
rs9905991	A	G	0.0106	0.0017
rs9922708	T	C	0.0306	0.0032
rs9927848	A	C	-0.0130	0.0020
rs9931407	T	C	0.0456	0.0058
rs9931967	T	G	0.0149	0.0017
rs993931	A	G	-0.0112	0.0019
rs993954	T	G	0.0115	0.0020
rs9951893	T	C	-0.0112	0.0018
rs9961813	A	C	0.0449	0.0043
rs9979651	C	G	-0.0209	0.0035
rs998584	A	C	-0.0156	0.0018
rs998732	A	G	0.0163	0.0022

TRIGLYCERIDES

SNP	A1	A2	Beta	SE
rs1004712	C	T	0.0206	0.0034
rs10401969	T	C	0.121	0.0065
rs10440120	C	A	0.0306	0.0044
rs10501321	T	C	0.0216	0.0035
rs10761762	T	C	0.027	0.0033
rs10790162	A	G	0.2305	0.0065
rs11057408	G	T	0.0258	0.0035
rs11613352	C	T	0.028	0.0039
rs11855284	C	T	0.0286	0.0042
rs11974409	A	G	0.0899	0.0042

rs12294259	T	C	0.219	0.0069
rs1260326	T	C	0.1148	0.0034
rs12678919	A	G	0.1702	0.0056
rs12708967	C	T	0.0289	0.0046
rs12748152	T	C	0.0372	0.0059
rs1321257	G	A	0.0402	0.0034
rs13276972	A	G	0.0494	0.0035
rs13389219	C	T	0.0271	0.0034
rs16948098	A	G	0.08	0.0089
rs174535	C	T	0.047	0.0034
rs17513135	T	C	0.022	0.0039
rs1800775	C	A	0.0396	0.0035
rs1832007	A	G	0.0327	0.0047
rs2068888	G	A	0.0241	0.0034
rs2075650	G	A	0.0436	0.0049
rs2250802	A	G	0.023	0.0037
rs2665357	C	A	0.0212	0.0033
rs2696089	A	G	0.0442	0.0072
rs287621	T	C	0.0222	0.0037
rs2954022	C	A	0.078	0.0033
rs2972146	T	G	0.0281	0.0034
rs2976940	T	G	0.0205	0.0034
rs3130564	C	T	0.033	0.0046
rs3198697	C	T	0.0198	0.0034
rs3289	C	T	0.1447	0.0111
rs3761445	A	G	0.0232	0.0034
rs38855	A	G	0.0187	0.0033
rs439401	C	T	0.0659	0.0038
rs4417316	C	T	0.0475	0.0054
rs442177	T	G	0.0309	0.0033
rs4587594	G	A	0.0694	0.0035

rs4635554	G	T	0.0228	0.0038
rs4646246	G	A	0.0285	0.0043
rs4719841	G	A	0.0232	0.0034
rs4738684	A	G	0.0205	0.0035
rs4803750	G	A	0.0423	0.007
rs4804311	A	G	0.0392	0.006
rs4810479	C	T	0.0474	0.0038
rs4871624	G	T	0.0254	0.0037
rs588136	C	T	0.0495	0.0041
rs589428	G	T	0.0335	0.0036
rs6029143	C	T	0.0388	0.0071
rs6066141	T	C	0.0297	0.0053
rs634869	T	C	0.0272	0.0033
rs645040	T	G	0.0293	0.004
rs676210	G	A	0.0733	0.0039
rs6831256	G	A	0.0258	0.0035
rs6882076	C	T	0.0286	0.0035
rs6995541	G	A	0.0265	0.0037
rs719726	T	C	0.0199	0.0035
rs7248104	G	A	0.0222	0.0034
rs731839	G	A	0.0224	0.0036
rs749671	G	A	0.0211	0.0034
rs75919952	C	T	0.0683	0.0104
rs76366838	A	G	0.1077	0.015
rs7942717	G	A	0.0393	0.0067
rs7943309	G	A	0.0605	0.0087
rs799158	T	C	0.102	0.013
rs8077889	C	A	0.0252	0.0042
rs947988	C	G	0.0422	0.0061
rs9686661	T	C	0.0379	0.0044
rs998584	A	C	0.0293	0.0037

**HIGH DENSITY LIPOPROTEIN**				
SNP	A1	A2	Beta	SE
rs10019888	A	G	0.027	0.0046
rs10087900	G	A	0.0231	0.0036
rs102275	T	C	0.0391	0.0035
rs103294	T	C	0.0523	0.0044
rs10468017	T	C	0.1179	0.0038
rs1047891	C	A	0.0269	0.0039
rs10488698	A	G	0.061	0.0071
rs10761771	C	T	0.0198	0.0034
rs10808546	T	C	0.0409	0.0034
rs11040329	C	T	0.038	0.0054
rs11045163	G	A	0.0217	0.0035
rs1121980	G	A	0.0196	0.0034
rs11229606	G	T	0.0335	0.0051
rs11246602	C	T	0.034	0.0052
rs11765979	C	A	0.0412	0.0048
rs11789603	T	C	0.06	0.006
rs12133576	A	G	0.0243	0.0035
rs12145743	G	T	0.0203	0.0036
rs12291885	C	T	0.0857	0.0149
rs12720917	C	T	0.0976	0.0055
rs12740374	T	G	0.0343	0.0041
rs12748152	C	T	0.0506	0.0062
rs12801636	A	G	0.0235	0.0042
rs13076253	A	C	0.0283	0.0048
rs13107325	C	T	0.0708	0.0078
rs13292026	G	A	0.0666	0.0108
rs13326165	A	G	0.0289	0.0043
rs13702	C	T	0.1058	0.0038

rs1515110	G	T	0.0323	0.0035
rs1689797	C	A	0.0358	0.0036
rs16942887	A	G	0.0831	0.0051
rs17135399	A	G	0.0483	0.0077
rs17145738	T	C	0.0408	0.0053
rs17173637	T	C	0.0363	0.0057
rs17695224	G	A	0.029	0.0039
rs1800961	C	T	0.127	0.0099
rs181360	T	G	0.0376	0.0042
rs185481	C	T	0.0366	0.0035
rs1877031	A	G	0.0336	0.0036
rs1883025	C	T	0.0698	0.0041
rs1936800	C	T	0.02	0.0034
rs2013208	T	C	0.0254	0.0036
rs2040293	A	G	0.0438	0.0039
rs205262	A	G	0.0283	0.0039
rs2075650	A	G	0.0554	0.0051
rs2148489	T	C	0.0283	0.0041
rs2241210	G	A	0.0332	0.0035
rs2241770	T	C	0.0989	0.0057
rs2250802	G	A	0.034	0.0038
rs2278236	A	G	0.0331	0.0035
rs2293889	G	T	0.0312	0.0035
rs2454722	G	A	0.0351	0.0044
rs2602836	A	G	0.0192	0.0034
rs2606736	C	T	0.0246	0.0043
rs2642438	G	A	0.0303	0.0039
rs2853579	T	G	0.0499	0.0053
rs289723	C	A	0.022	0.0038
rs2925979	C	T	0.0351	0.0037
rs3111576	T	C	0.0448	0.0054

rs333947	G	A	0.0296	0.0047
rs3741414	T	C	0.0296	0.004
rs3822072	G	A	0.0251	0.0034
rs3861397	A	G	0.024	0.0036
rs3936511	A	G	0.0308	0.0046
rs4142995	G	T	0.0263	0.0037
rs4148005	T	G	0.0283	0.0036
rs4240624	A	G	0.0818	0.0058
rs4465830	A	G	0.0597	0.0044
rs459193	A	G	0.0235	0.0039
rs4599828	T	C	0.0232	0.0035
rs4650994	G	A	0.021	0.0034
rs4660293	A	G	0.0353	0.004
rs4783961	A	G	0.0997	0.0036
rs4783972	C	A	0.022	0.0036
rs4846914	A	G	0.0479	0.0034
rs4917014	G	T	0.0222	0.0036
rs492571	T	C	0.0663	0.009
rs4939883	C	T	0.0799	0.0045
rs4969178	G	A	0.0263	0.0035
rs499974	C	A	0.0263	0.0044
rs5167	G	T	0.032	0.0037
rs6450176	G	A	0.0254	0.0039
rs653178	T	C	0.0263	0.0035
rs6567160	T	C	0.0257	0.0041
rs676210	A	G	0.066	0.004
rs6805251	T	C	0.02	0.0035
rs686030	A	C	0.055	0.0049
rs687339	C	T	0.0316	0.0042
rs7014168	G	A	0.0267	0.0041
rs7016529	T	C	0.2186	0.0141

rs702485	G	A	0.0243	0.0034
rs7117842	C	T	0.0272	0.0035
rs7128597	C	A	0.0398	0.0065
rs7179726	C	T	0.022	0.0034
rs7306660	G	A	0.0345	0.0036
rs731839	A	G	0.022	0.0037
rs737337	T	C	0.0565	0.0061
rs7607980	C	T	0.0447	0.0052
rs783149	C	A	0.0313	0.0046
rs7942717	A	G	0.0397	0.0068
rs7959530	C	T	0.0281	0.0048
rs7973683	A	C	0.0286	0.0036
rs8033940	A	G	0.0978	0.0038
rs8093249	A	G	0.0384	0.0051
rs838876	A	G	0.0493	0.0039
rs9457931	A	G	0.0552	0.0073
rs964184	C	G	0.1065	0.0071
rs970548	C	A	0.0258	0.0039
rs9955201	A	G	0.0638	0.0081
rs998584	C	A	0.026	0.0038
rs9989419	G	A	0.1473	0.0036
**LOW DENSITY LIPOPROTEIN**				
SNP	A1	A2	Beta	SE
rs10102164	A	G	0.0316	0.0045
rs10195252	T	C	0.0238	0.0039
rs1025447	C	T	0.0418	0.0048
rs10401969	T	C	0.1184	0.0072
rs10403668	G	A	0.0439	0.0052
rs10490626	G	A	0.0508	0.0069
rs10832962	T	C	0.032	0.004

rs10893499	A	G	0.0521	0.0053
rs10903129	G	A	0.0328	0.0037
rs11206510	T	C	0.0831	0.005
rs11485618	A	G	0.05	0.0039
rs11563251	T	C	0.0345	0.0062
rs11591147	G	T	0.497	0.018
rs11600380	T	C	0.0558	0.0081
rs1169288	C	A	0.0375	0.004
rs117733303	G	A	0.1551	0.022
rs1250229	C	T	0.0243	0.0042
rs12670798	C	T	0.0344	0.0043
rs12721109	G	A	0.4462	0.0183
rs12748152	T	C	0.0499	0.0066
rs12916	C	T	0.0733	0.0038
rs12974306	T	G	0.0221	0.0037
rs12983889	A	G	0.056	0.0068
rs13206249	G	A	0.0378	0.0062
rs13277801	C	T	0.0338	0.0038
rs1367117	A	G	0.1186	0.004
rs1475701	C	T	0.0904	0.0092
rs1531517	G	A	0.2202	0.008
rs1564348	C	T	0.0481	0.005
rs16872670	A	G	0.0553	0.0084
rs17404153	G	T	0.0336	0.0054
rs174583	C	T	0.0522	0.0038
rs1800562	G	A	0.0615	0.008
rs1800961	C	T	0.0685	0.0106
rs1883025	C	T	0.0296	0.0044
rs2000999	A	G	0.065	0.0046
rs2030746	T	C	0.0214	0.0038
rs2073547	G	A	0.0485	0.0049

rs2075650	G	A	0.1767	0.0055
rs2328223	C	A	0.0299	0.005
rs2419604	A	G	0.0302	0.004
rs247616	C	T	0.0547	0.0041
rs2587534	A	G	0.0391	0.0037
rs2642438	G	A	0.0352	0.0042
rs267733	A	G	0.0331	0.0053
rs2710642	A	G	0.0239	0.0038
rs2737252	G	A	0.0314	0.0041
rs2886232	T	C	0.0451	0.0064
rs289714	G	A	0.0358	0.0054
rs2954029	A	T	0.0564	0.0036
rs3125055	A	T	0.0468	0.0055
rs3184504	C	T	0.0268	0.0038
rs364585	G	A	0.0249	0.0038
rs3745148	G	A	0.0497	0.0089
rs3757354	C	T	0.0382	0.0044
rs3780181	A	G	0.0445	0.0074
rs4246169	A	C	0.0319	0.0051
rs4253776	G	A	0.0311	0.0059
rs4530754	A	G	0.0275	0.0036
rs461473	G	A	0.0427	0.0064
rs4665790	T	A	0.0259	0.0036
rs4722551	C	T	0.0391	0.0049
rs492602	G	A	0.0293	0.0039
rs4927207	G	A	0.0692	0.0049
rs4942486	T	C	0.0243	0.0037
rs4970712	C	A	0.0339	0.0044
rs520861	G	A	0.0843	0.0042
rs5763662	T	C	0.0767	0.0121
rs579459	C	T	0.0665	0.0045

rs6016373	A	G	0.0349	0.0037
rs6065311	C	T	0.0417	0.0036
rs646776	T	C	0.1602	0.0044
rs6504872	T	C	0.0274	0.0037
rs6511720	G	T	0.2209	0.0061
rs6544713	T	C	0.0806	0.0041
rs6818397	T	G	0.0224	0.004
rs688	T	C	0.054	0.0037
rs6882076	C	T	0.0456	0.0038
rs6909746	C	T	0.0263	0.0037
rs7254892	G	A	0.4853	0.0119
rs7264396	C	T	0.0246	0.0045
rs76366838	A	G	0.1704	0.0167
rs7640978	C	T	0.0392	0.0069
rs7774197	C	A	0.0508	0.0073
rs780093	T	C	0.0223	0.0037
rs7832643	T	G	0.0339	0.0038
rs8017377	A	G	0.0303	0.0038
rs964184	G	C	0.0855	0.0078
rs9875338	G	A	0.027	0.0037
rs9938506	G	A	0.0283	0.004
rs9987289	G	A	0.0714	0.0066
**EVER SMOKING**				
SNP	A1	A2	Beta	SE
rs10042827	C	T	0.0167	0.0027
rs1004787	A	G	0.0284	0.0026
rs10060196	A	C	0.0183	0.0026
rs1008078	T	C	0.0228	0.0026
rs1022376	C	T	-0.0147	0.0026
rs1022528	A	G	0.0174	0.0027

rs10233018	G	A	0.0246	0.0025
rs10272990	C	T	-0.0209	0.0027
rs10279261	A	G	-0.0189	0.0026
rs1030015	T	G	0.0143	0.0026
rs10446419	G	A	-0.0196	0.0031
rs10490159	T	C	0.0172	0.0026
rs1050847	T	C	-0.0148	0.0026
rs1059490	C	T	-0.0186	0.0026
rs10698713	A	G	-0.0335	0.0056
rs10789369	G	A	-0.0234	0.0026
rs10805858	T	A	0.0181	0.0027
rs10853981	A	G	0.0148	0.0027
rs10858334	G	C	0.0229	0.0038
rs10873871	G	A	0.0175	0.0031
rs10885480	C	T	-0.0187	0.0028
rs10905461	C	T	-0.0164	0.0029
rs10914684	A	G	-0.0158	0.0027
rs10935779	T	C	-0.0143	0.0026
rs10945141	A	G	0.0181	0.0029
rs10953957	A	G	0.0144	0.0026
rs10966092	C	T	-0.0205	0.0029
rs10969352	A	T	0.0143	0.0025
rs11057005	G	A	-0.0157	0.0026
rs1106363	T	C	0.0174	0.0027
rs11076962	C	T	0.0183	0.0028
rs11078713	G	A	-0.0146	0.0026
rs1108130	A	T	0.0239	0.0031
rs1109480	A	G	-0.0167	0.0026
rs11128203	A	T	0.0204	0.0026
rs11162019	T	C	-0.0155	0.0026
rs1116690	G	A	0.0163	0.0029

rs11191269	G	C	0.0176	0.0032
rs11192347	A	G	-0.0265	0.0043
rs11258417	T	C	-0.0145	0.0026
rs1126757	T	C	0.0142	0.0026
rs112725451	T	C	0.0261	0.0034
rs113230003	A	G	-0.0189	0.0029
rs114976176	C	A	-0.0155	0.0027
rs1150668	G	T	-0.0185	0.0026
rs11587399	T	A	-0.0178	0.0031
rs11594623	C	T	0.0274	0.0030
rs1160685	G	C	0.0153	0.0026
rs11611651	A	G	0.0271	0.0045
rs11642231	A	G	-0.0156	0.0026
rs11651955	A	G	-0.0140	0.0025
rs11678980	A	G	0.0177	0.0026
rs11692435	A	G	0.0251	0.0046
rs11713899	C	A	0.0187	0.0034
rs1173461	T	C	0.0166	0.0027
rs117657830	G	A	-0.0378	0.0064
rs11766326	C	T	-0.0175	0.0026
rs11768481	A	C	-0.0186	0.0027
rs117734003	C	G	0.0303	0.0051
rs11780471	A	G	-0.0387	0.0052
rs11783093	T	C	-0.0471	0.0035
rs11791671	T	C	0.0279	0.0051
rs118202	T	G	-0.0367	0.0033
rs11872397	A	G	-0.0171	0.0029
rs1187820	T	C	-0.0143	0.0026
rs11889814	C	A	-0.0210	0.0038
rs11956866	G	T	-0.0148	0.0026
rs12022778	C	A	0.0268	0.0032

rs12027999	C	T	-0.0244	0.0039
rs12053870	G	T	0.0156	0.0026
rs12130857	A	G	-0.0180	0.0027
rs12195240	A	G	0.0249	0.0028
rs12244388	A	G	0.0258	0.0027
rs12474587	T	G	0.0242	0.0026
rs12517438	G	T	0.0154	0.0026
rs12530388	C	A	-0.0184	0.0025
rs12563365	A	G	0.0166	0.0026
rs12633090	C	G	-0.0230	0.0033
rs12642744	T	G	-0.0166	0.0030
rs12714017	C	T	0.0154	0.0026
rs12739243	C	T	-0.0213	0.0031
rs12740789	A	G	-0.0285	0.0033
rs12755632	G	A	-0.0154	0.0027
rs12855717	T	C	0.0155	0.0026
rs12878369	A	C	0.0174	0.0026
rs12918191	G	A	-0.0197	0.0030
rs1291821	G	A	0.0145	0.0026
rs13007361	A	G	0.0175	0.0031
rs13066050	T	C	0.0188	0.0031
rs13109980	A	G	-0.0222	0.0027
rs13110073	C	T	-0.0246	0.0026
rs13237637	C	G	-0.0237	0.0025
rs13261666	T	G	-0.0200	0.0025
rs13319205	A	T	0.0165	0.0028
rs13392222	C	A	-0.0234	0.0037
rs13437771	G	A	-0.0271	0.0035
rs1373178	G	T	-0.0203	0.0026
rs1381287	T	C	0.0180	0.0026
rs1381775	C	T	-0.0156	0.0028

rs1385108	T	C	0.0187	0.0030
rs1389171	A	T	-0.0175	0.0030
rs13906	T	C	-0.0245	0.0041
rs139896	C	T	0.0154	0.0027
rs1413119	T	C	-0.0153	0.0026
rs1435479	T	G	0.0164	0.0028
rs1435672	C	T	0.0141	0.0026
rs1435741	A	G	0.0183	0.0026
rs1445649	C	T	0.0206	0.0026
rs1449012	T	C	-0.0154	0.0026
rs147052174	T	G	0.0623	0.0098
rs1514176	A	G	-0.0193	0.0026
rs1518393	C	A	0.0169	0.0026
rs1549979	T	C	-0.0245	0.0026
rs1555445	T	A	0.0188	0.0027
rs1561112	C	T	-0.0152	0.0026
rs1565735	A	T	-0.0192	0.0032
rs160631	G	T	-0.0173	0.0029
rs1632941	C	T	-0.0158	0.0026
rs16826827	C	T	-0.0222	0.0039
rs16828799	T	G	0.0198	0.0035
rs1713676	G	A	-0.0167	0.0026
rs1714521	C	A	-0.0163	0.0026
rs17165769	G	A	0.0159	0.0026
rs17197663	A	G	-0.0216	0.0039
rs1722666	T	C	0.0161	0.0029
rs17229285	T	C	-0.0155	0.0025
rs1733760	C	T	0.0148	0.0025
rs1737329	G	C	0.0170	0.0029
rs17554906	C	G	0.0142	0.0026
rs1759433	A	G	0.0154	0.0026

rs17616642	G	A	-0.0166	0.0030
rs17692129	T	C	0.0196	0.0027
rs1772572	A	C	-0.0169	0.0027
rs1799068	T	G	0.0166	0.0026
rs1811739	A	G	0.0183	0.0030
rs1834306	G	A	-0.0145	0.0026
rs1863161	A	G	0.0153	0.0026
rs1889571	G	T	0.0222	0.0038
rs1901477	G	A	0.0304	0.0026
rs1910236	A	G	0.0146	0.0026
rs1927901	C	T	-0.0142	0.0026
rs1930371	T	C	-0.0172	0.0030
rs1931431	C	G	0.0182	0.0026
rs1935571	G	T	-0.0157	0.0026
rs1937443	G	C	0.0204	0.0026
rs1944689	T	G	0.0177	0.0031
rs2010921	A	G	0.0174	0.0028
rs2028269	A	G	0.0162	0.0026
rs2063976	T	C	-0.0202	0.0027
rs2155646	C	T	0.0378	0.0026
rs2173019	A	T	0.0282	0.0033
rs2196356	C	G	-0.0188	0.0028
rs221988	C	A	-0.0149	0.0026
rs2276825	C	T	0.0189	0.0030
rs2279829	T	C	-0.0174	0.0031
rs2289791	T	G	-0.0177	0.0030
rs2306866	T	A	-0.0167	0.0026
rs2319545	A	C	0.0232	0.0036
rs2344976	C	T	-0.0151	0.0026
rs2378662	A	G	0.0152	0.0026
rs238896	A	G	-0.0169	0.0025

rs2526390	T	C	0.0205	0.0027
rs2539706	A	G	0.0162	0.0026
rs2637869	A	G	0.0182	0.0028
rs2710634	C	T	-0.0178	0.0026
rs2734390	G	A	0.0148	0.0026
rs2796793	A	G	0.0145	0.0026
rs281296	A	G	0.0247	0.0027
rs28408682	G	A	0.0167	0.0026
rs28441558	C	T	-0.0356	0.0055
rs28717373	T	C	-0.0165	0.0027
rs2901785	A	G	-0.0173	0.0026
rs290601	T	C	0.0163	0.0029
rs2925128	T	C	0.0168	0.0027
rs2938134	A	C	-0.0175	0.0028
rs2939756	A	G	-0.0157	0.0026
rs2952251	G	A	0.0164	0.0030
rs2959084	A	G	0.0171	0.0028
rs301807	G	A	0.0180	0.0026
rs3098272	C	A	-0.0178	0.0032
rs3115418	C	T	-0.0142	0.0026
rs3172494	T	G	-0.0291	0.0040
rs3218116	T	C	-0.0198	0.0029
rs329124	G	A	-0.0164	0.0026
rs34342129	C	T	-0.0143	0.0025
rs34399632	G	A	0.0194	0.0030
rs34553878	G	A	0.0247	0.0041
rs34940743	G	A	0.0159	0.0027
rs34970111	T	C	-0.0146	0.0026
rs35375873	C	G	-0.0270	0.0041
rs35656245	A	G	0.0159	0.0029
rs357304	C	T	0.0167	0.0029

rs359247	T	A	0.0220	0.0027
rs359431	T	C	-0.0142	0.0026
rs3740977	C	T	0.0195	0.0034
rs3764351	A	G	-0.0147	0.0027
rs3800227	G	A	0.0172	0.0029
rs3810496	C	T	0.0159	0.0026
rs3811038	C	T	0.0191	0.0028
rs3820277	T	G	-0.0188	0.0026
rs3843905	T	C	-0.0151	0.0026
rs3847244	T	C	0.0187	0.0026
rs3850736	G	C	0.0191	0.0026
rs3909281	G	T	0.0211	0.0026
rs3934797	A	G	-0.0213	0.0033
rs4044321	G	A	-0.0226	0.0027
rs4140932	A	T	-0.0140	0.0026
rs42417	T	C	0.0169	0.0028
rs4264267	T	C	0.0148	0.0026
rs4275621	G	A	-0.0214	0.0026
rs4310804	G	C	-0.0182	0.0030
rs4326350	G	C	-0.0176	0.0026
rs4476253	A	G	-0.0185	0.0030
rs4543050	T	A	0.0222	0.0033
rs45444697	G	C	0.0197	0.0031
rs4674916	A	C	-0.0180	0.0027
rs4674993	G	A	-0.0240	0.0032
rs4727189	C	T	0.0149	0.0027
rs4752018	A	C	0.0189	0.0030
rs4759229	G	A	0.0156	0.0027
rs4785187	A	G	0.0200	0.0031
rs4788676	C	T	-0.0177	0.0030
rs4790874	T	C	0.0174	0.0026

rs4818005	A	G	-0.0204	0.0026
rs4822102	T	C	-0.0165	0.0026
rs4837631	T	C	-0.0154	0.0026
rs4877285	A	G	-0.0181	0.0027
rs4886207	C	T	-0.0162	0.0026
rs4912332	T	C	0.0141	0.0025
rs540860	G	A	0.0176	0.0026
rs55786907	G	A	0.0194	0.0035
rs55913542	T	G	0.0186	0.0034
rs55944129	C	T	-0.0176	0.0029
rs56208390	G	A	0.0216	0.0039
rs56367474	T	C	-0.0173	0.0028
rs56902655	G	T	-0.0219	0.0037
rs57153235	G	T	-0.0194	0.0027
rs58400863	A	G	-0.0202	0.0027
rs586699	A	G	-0.0148	0.0026
rs59537158	T	C	0.0225	0.0031
rs6011779	T	C	-0.0192	0.0032
rs6050446	G	A	0.0544	0.0076
rs6058782	T	C	0.0297	0.0044
rs6073075	A	T	-0.0187	0.0034
rs60833441	G	A	-0.0143	0.0026
rs61533748	C	T	0.0174	0.0026
rs61884449	T	C	0.0200	0.0036
rs61886926	T	C	-0.0179	0.0026
rs619087	G	A	0.0143	0.0026
rs61959481	A	G	-0.0203	0.0031
rs62007780	T	G	-0.0159	0.0026
rs62052916	T	A	-0.0319	0.0050
rs62098013	A	G	0.0177	0.0026
rs62137126	G	A	-0.0237	0.0039

rs62180324	A	G	-0.0195	0.0031
rs62193862	A	G	0.0238	0.0042
rs62246017	A	G	-0.0162	0.0027
rs62340589	C	G	0.0174	0.0032
rs62618693	T	C	-0.0353	0.0063
rs6265	T	C	-0.0293	0.0033
rs6437769	T	C	0.0142	0.0026
rs6438436	T	C	0.0247	0.0033
rs644740	T	C	-0.0141	0.0026
rs6452785	T	C	-0.0269	0.0026
rs6474609	A	T	-0.0156	0.0026
rs6497840	A	G	0.0228	0.0029
rs6568832	A	G	0.0189	0.0030
rs67050670	G	A	-0.0203	0.0030
rs6730325	A	G	-0.0146	0.0026
rs6731872	G	T	0.0316	0.0034
rs6750107	A	G	0.0146	0.0026
rs6750529	T	C	0.0199	0.0029
rs6756212	T	C	-0.0339	0.0026
rs67777803	T	G	-0.0246	0.0034
rs6782116	T	C	-0.0147	0.0026
rs6874731	G	T	0.0153	0.0025
rs6890961	T	C	-0.0193	0.0026
rs6932350	A	T	0.0150	0.0026
rs6936160	T	C	0.0201	0.0028
rs6948707	G	T	0.0243	0.0026
rs6968380	A	G	-0.0234	0.0027
rs6986430	C	T	-0.0243	0.0031
rs6993429	A	C	-0.0191	0.0026
rs7024924	C	T	0.0189	0.0034
rs7026534	G	T	-0.0166	0.0028

rs7072776	G	A	-0.0220	0.0028
rs7134009	C	T	-0.0158	0.0029
rs71367544	T	C	0.0206	0.0032
rs71592686	C	T	0.0207	0.0029
rs71602617	T	C	-0.0178	0.0032
rs7188873	G	A	0.0203	0.0026
rs7192140	C	T	-0.0169	0.0025
rs72780746	C	T	-0.0258	0.0034
rs72789626	A	T	-0.0256	0.0037
rs72790288	A	G	-0.0455	0.0077
rs72898831	G	A	-0.0244	0.0035
rs72938304	A	G	-0.0272	0.0040
rs73008357	C	A	-0.0223	0.0040
rs7333559	A	G	-0.0232	0.0031
rs73831818	G	A	0.0320	0.0055
rs74697736	A	G	0.0223	0.0028
rs748832	G	A	0.0172	0.0026
rs7505855	T	C	-0.0170	0.0026
rs75210106	T	C	-0.0187	0.0033
rs75674569	A	G	-0.0253	0.0043
rs7585579	G	C	0.0204	0.0026
rs75919030	C	T	-0.0210	0.0029
rs7598402	G	C	-0.0147	0.0025
rs7600835	A	G	-0.0151	0.0027
rs7631379	C	T	0.0208	0.0032
rs7640107	T	C	-0.0142	0.0026
rs7657022	G	A	0.0183	0.0025
rs76841737	G	C	-0.0231	0.0042
rs7696257	A	G	0.0153	0.0026
rs77215829	C	A	-0.0240	0.0038
rs77283305	A	G	-0.0152	0.0028

rs7743165	G	T	0.0193	0.0025
rs7802996	T	C	-0.0209	0.0034
rs7809303	A	G	-0.0214	0.0027
rs7836565	T	C	-0.0155	0.0028
rs7867822	G	A	-0.0151	0.0027
rs7901883	A	G	-0.0193	0.0030
rs7920501	A	T	-0.0155	0.0026
rs7921378	C	G	-0.0233	0.0025
rs7929518	G	A	0.0192	0.0030
rs7943721	A	G	-0.0212	0.0034
rs79476395	G	A	0.0334	0.0049
rs7969559	G	A	-0.0170	0.0028
rs8005334	G	T	0.0167	0.0027
rs8027457	C	T	0.0153	0.0025
rs8050598	T	C	0.0187	0.0029
rs8083764	T	G	-0.0160	0.0028
rs8096225	C	A	0.0155	0.0028
rs8103660	C	T	0.0158	0.0027
rs876793	C	T	-0.0179	0.0027
rs910912	C	T	-0.0168	0.0029
rs925524	G	A	0.0156	0.0028
rs9288999	A	G	0.0174	0.0029
rs9302604	G	A	0.0187	0.0026
rs9323328	G	A	-0.0142	0.0026
rs951740	A	G	0.0295	0.0026
rs9538162	C	T	0.0174	0.0026
rs9540731	T	C	-0.0177	0.0025
rs9545155	C	T	-0.0161	0.0026
rs963354	A	C	0.0150	0.0027
rs9787523	C	T	-0.0156	0.0026
rs9826984	A	G	-0.0141	0.0026

rs9841807	T	C	0.0163	0.0029
rs9850597	A	G	-0.0186	0.0033
rs986714	T	A	-0.0160	0.0026
rs9922607	T	C	-0.0222	0.0032
rs9936784	G	T	0.0140	0.0026
rs9941217	G	C	-0.0186	0.0027
rs9987376	G	T	-0.0205	0.0026

TYPE 2 DIABETES

SNP	A1	A2	Beta	SE
rs1005752	A	C	0.0770	0.0047
rs10096633	C	T	0.0677	0.0096
rs10097617	T	C	0.0392	0.0049
rs10195252	T	C	0.0677	0.0048
rs10228066	T	C	0.0677	0.0048
rs10406327	C	G	0.0392	0.0099
rs10406431	A	G	0.0488	0.0049
rs1061810	A	C	0.0488	0.0049
rs10811660	G	A	0.2390	0.0122
rs10830963	G	C	0.0953	0.0047
rs10842994	C	T	0.0770	0.0095
rs10882101	T	C	0.0583	0.0097
rs10908278	T	A	0.0770	0.0047
rs10937721	C	G	0.0583	0.0097
rs10938398	A	G	0.0488	0.0098
rs10954772	T	C	0.0392	0.0049
rs10974438	C	A	0.0488	0.0049
rs11070332	A	G	0.0488	0.0049
rs11137820	C	G	0.0392	0.0099
rs11257655	T	C	0.0862	0.0047
rs1127215	C	T	0.0488	0.0049

rs11496066	T	C	0.0770	0.0144
rs115505614	T	C	0.1740	0.0174
rs11642430	G	C	0.0392	0.0049
rs11688682	G	C	0.0488	0.0098
rs11699802	C	T	0.0392	0.0049
rs117001013	C	T	0.0677	0.0145
rs11708067	A	G	0.0862	0.0047
rs11709077	G	A	0.1310	0.0136
rs117483894	G	A	0.0953	0.0189
rs11759026	G	A	0.0677	0.0096
rs11820019	T	C	0.1484	0.0225
rs11842871	G	T	0.0392	0.0049
rs11926707	C	T	0.2390	0.0418
rs12001437	C	T	0.0392	0.0049
rs12048743	G	C	0.0392	0.0049
rs12140153	G	T	0.0677	0.0145
rs1260326	C	T	0.0677	0.0048
rs12640250	C	A	0.0392	0.0049
rs12680028	C	G	0.0392	0.0099
rs12811407	A	G	0.0488	0.0049
rs12910825	G	A	0.0488	0.0049
rs12920022	A	T	0.0488	0.0049
rs1296328	A	C	0.0392	0.0099
rs13041756	C	T	0.0583	0.0097
rs13085136	C	T	0.0770	0.0144
rs1316776	C	A	0.0488	0.0098
rs13262861	C	A	0.0677	0.0096
rs13426680	A	G	0.0862	0.0142
rs1359790	G	A	0.0862	0.0094
rs13737	G	T	0.0488	0.0098
rs1377807	C	G	0.0488	0.0049

rs1412234	C	T	0.0392	0.0049
rs141521721	A	C	0.1222	0.0231
rs1421085	C	T	0.1222	0.0045
rs1426371	G	A	0.0488	0.0049
rs145678014	G	T	0.1044	0.0187
rs1493694	T	C	0.0862	0.0094
rs1531583	T	G	0.1222	0.0184
rs1561927	C	T	0.0392	0.0049
rs1562396	G	A	0.0583	0.0048
rs1580278	C	A	0.0392	0.0049
rs1708302	C	T	0.0953	0.0094
rs17122772	G	C	0.0392	0.0049
rs17522122	T	G	0.0392	0.0049
rs17684074	G	C	0.0392	0.0049
rs17689007	G	A	0.0392	0.0049
rs17791513	A	G	0.0953	0.0094
rs17802463	G	T	0.0392	0.0049
rs1783541	T	C	0.0583	0.0048
rs17836088	C	G	0.0583	0.0097
rs1796330	G	C	0.0488	0.0049
rs1800961	T	C	0.1655	0.0131
rs1903002	G	C	0.0392	0.0099
rs2102278	G	A	0.0392	0.0099
rs2197973	T	C	0.0392	0.0099
rs2237895	C	A	0.1133	0.0046
rs2249105	A	G	0.0953	0.0094
rs2258238	T	A	0.0953	0.0094
rs2268078	A	G	0.0392	0.0049
rs2272163	C	A	0.0392	0.0099
rs2280141	T	G	0.0488	0.0098
rs2307111	T	C	0.0488	0.0049

rs243024	A	G	0.0583	0.0048
rs2456530	T	C	0.0583	0.0097
rs2581787	T	G	0.0392	0.0099
rs2642588	G	T	0.0488	0.0049
rs2767036	C	A	0.0392	0.0099
rs2796441	G	A	0.0677	0.0096
rs2800733	A	G	0.0488	0.0098
rs2820446	C	G	0.0583	0.0097
rs2872246	A	C	0.0392	0.0099
rs28819812	C	A	0.0392	0.0049
rs291367	G	A	0.0392	0.0049
rs2925979	T	C	0.0488	0.0049
rs2972144	G	A	0.0953	0.0094
rs3111316	A	G	0.0488	0.0098
rs329122	A	G	0.0392	0.0049
rs340874	C	T	0.0677	0.0096
rs34298980	T	C	0.0392	0.0049
rs34454109	A	T	0.0392	0.0049
rs34584161	A	G	0.0488	0.0098
rs34715063	C	T	0.0953	0.0141
rs348330	G	A	0.0488	0.0049
rs34855406	C	G	0.0488	0.0049
rs34965774	A	G	0.0583	0.0097
rs35352848	T	C	0.0677	0.0096
rs35895680	C	A	0.0583	0.0097
rs35999103	T	C	0.0488	0.0098
rs362307	T	C	0.0770	0.0144
rs3751837	T	C	0.0392	0.0049
rs3772071	T	C	0.0488	0.0098
rs3774723	G	A	0.0677	0.0096
rs3798519	C	A	0.0583	0.0097

rs3802177	G	A	0.1044	0.0046
rs3810291	A	G	0.0488	0.0098
rs3811978	G	A	0.0583	0.0097
rs3887925	T	C	0.0677	0.0096
rs39328	T	C	0.0392	0.0099
rs4148856	C	G	0.0488	0.0098
rs4279506	G	C	0.0583	0.0097
rs429358	T	C	0.0770	0.0095
rs4457053	G	A	0.0583	0.0048
rs465002	T	C	0.1044	0.0093
rs4686471	C	T	0.0583	0.0048
rs4688760	T	C	0.0392	0.0049
rs4709746	C	T	0.0583	0.0097
rs474513	A	G	0.0392	0.0049
rs4776970	A	T	0.0392	0.0049
rs4804833	A	G	0.0488	0.0098
rs4925109	A	G	0.0488	0.0098
rs4929965	A	G	0.0677	0.0048
rs4932265	T	C	0.0677	0.0096
rs4946812	G	A	0.0392	0.0049
rs4977213	C	T	0.0488	0.0049
rs505922	C	T	0.0488	0.0098
rs523288	T	A	0.0488	0.0049
rs539515	C	A	0.0488	0.0049
rs55653563	A	C	0.0392	0.0049
rs56337234	C	T	0.0583	0.0097
rs56348580	G	C	0.0488	0.0049
rs57327348	A	T	0.0392	0.0099
rs5758223	A	G	0.0392	0.0049
rs576674	G	A	0.0488	0.0049
rs58432198	C	T	0.0677	0.0096

rs58730668	T	C	0.0677	0.0096
rs601945	G	A	0.0583	0.0097
rs60276348	T	C	0.0488	0.0098
rs6063048	G	A	0.0488	0.0098
rs6070625	G	C	0.0488	0.0049
rs61676547	C	G	0.0583	0.0097
rs62007683	G	T	0.0392	0.0099
rs62080313	C	T	0.0583	0.0097
rs62271373	A	T	0.0862	0.0142
rs62492368	A	G	0.0488	0.0098
rs6458354	C	T	0.0488	0.0049
rs6459733	G	C	0.0583	0.0048
rs649961	T	C	0.0392	0.0049
rs6518681	G	A	0.0862	0.0142
rs6545714	G	A	0.0392	0.0099
rs6600191	T	C	0.0583	0.0048
rs67232546	T	C	0.0583	0.0097
rs6780171	A	T	0.1310	0.0090
rs6821438	A	G	0.0392	0.0049
rs6884702	G	A	0.0392	0.0049
rs6976111	A	C	0.0392	0.0049
rs7022807	G	A	0.0392	0.0049
rs702634	A	G	0.0488	0.0049
rs703972	G	C	0.0677	0.0048
rs7115753	A	G	0.0392	0.0049
rs7124681	A	C	0.0392	0.0049
rs71372253	C	T	0.0770	0.0144
rs7178762	C	T	0.0392	0.0049
rs718314	G	A	0.0488	0.0098
rs7222481	C	G	0.0392	0.0049
rs7240767	C	T	0.0392	0.0099

rs7249758	A	G	0.0488	0.0098
rs72802342	C	A	0.1570	0.0133
rs72926932	C	A	0.0862	0.0094
rs738408	T	C	0.0488	0.0098
rs75253922	C	T	0.0488	0.0098
rs76263492	T	G	0.0862	0.0142
rs7629630	A	T	0.0488	0.0098
rs7669833	T	A	0.0583	0.0097
rs7719891	G	A	0.0392	0.0049
rs77464186	A	C	0.1044	0.0093
rs7756992	G	A	0.1398	0.0090
rs77864822	A	G	0.0770	0.0144
rs7867635	C	T	0.0392	0.0099
rs7903146	T	C	0.3148	0.0075
rs7987740	T	C	0.0392	0.0099
rs8010382	G	A	0.0392	0.0049
rs80147536	A	T	0.1222	0.0091
rs8017808	G	T	0.0392	0.0049
rs8037894	G	C	0.0488	0.0098
rs8046545	G	A	0.0392	0.0099
rs8107974	T	A	0.0953	0.0141
rs862320	C	T	0.0392	0.0049
rs878521	A	G	0.0583	0.0097
rs917195	C	T	0.0488	0.0049
rs9379084	G	A	0.1044	0.0140
rs9430095	C	G	0.0392	0.0099
rs9494624	A	G	0.0392	0.0049
rs9537803	C	T	0.0392	0.0049
rs9563615	A	T	0.0488	0.0098
rs963740	A	T	0.0392	0.0049
rs9828772	C	G	0.0583	0.0097

rs9860730	A	G	0.0583	0.0097	
rs9873618	G	A	0.0677	0.0096	
rs9957145	G	A	0.0488	0.0098	

BLOOD PRESSURE

SNP	A1	A2	Beta_SBP	Beta_DBP	Beta_PP
rs10057188	G	A	0.182	0.03	0.153
rs10103353	C	T	0.18	0.147	0.04
rs1015538	A	G	0.098	0.137	-0.03
rs10198275	A	C	0.171	0.135	0.046
rs10199082	T	C	0.121	-0.15	0.26
rs10418305	C	G	-0.225	0.125	-0.33
rs1053711	G	A	0.079	0.153	-0.073
rs1063281	C	T	0.247	0.17	0.083
rs10747570	A	G	0.256	0.184	0.066
rs10751962	C	T	-0.242	-0.219	-0.025
rs10784502	C	T	-0.235	-0.043	-0.188
rs10818775	C	T	0.332	0.052	0.263
rs10838433	G	A	-0.144	0.057	-0.195
rs10859580	A	T	-0.113	0.044	-0.143
rs10958717	G	C	-0.113	0.042	-0.168
rs11008355	G	C	0.104	-0.072	0.168
rs11021221	T	A	0.064	0.213	-0.143
rs11030119	G	A	0.196	0.18	0.03
rs111304266	C	G	-0.459	-0.37	-0.114
rs111630016	C	T	0.386	0.324	0.052
rs11168244	C	T	0.318	0.165	0.147
rs112204826	C	T	-0.598	-0.395	-0.189
rs11222386	G	C	-0.108	0.051	-0.178
rs112875651	G	A	0.226	0.11	0.116
rs113161639	G	T	0.451	0.288	0.16

rs114407963	A	T	-0.087	0.162	-0.234
rs114534	G	A	0.159	0.001	0.154
rs11486794	C	T	0.304	0.219	0.061
rs1152958	G	A	0.145	0.138	0.014
rs11556924	C	T	0.193	0.181	0.021
rs11631778	G	A	-0.033	-0.141	0.107
rs11638064	G	A	-0.141	0.02	-0.168
rs11677932	G	A	0.103	-0.041	0.151
rs11690961	A	C	0.185	-0.127	0.313
rs11701033	C	G	-0.253	-0.083	-0.178
rs11708647	G	A	0.131	-0.058	0.178
rs117204111	G	A	0.428	-0.048	0.491
rs11993898	T	C	-0.326	-0.159	-0.162
rs12037669	T	G	0.162	-0.062	0.22
rs12050260	T	C	0.21	0.014	0.193
rs12206253	C	T	0.168	-0.135	0.287
rs12208834	A	G	-0.175	-0.021	-0.15
rs12248718	A	G	-0.21	-0.054	-0.154
rs12405515	G	T	0.189	0.17	0.02
rs12485003	G	A	-0.169	0.147	-0.327
rs1250247	C	G	0.31	0.013	0.279
rs12504699	G	A	0.221	0.109	0.103
rs12538229	C	T	0.298	0.004	0.289
rs12596053	A	C	-0.275	-0.162	-0.111
rs12606620	G	T	0.292	0.041	0.229
rs1261744	T	C	-0.196	0.077	-0.264
rs12630213	C	T	0.276	0.153	0.107
rs12636552	A	G	0.241	0.087	0.144
rs12670854	A	G	0.36	0.171	0.18
rs12906962	T	C	-0.179	-0.16	-0.033
rs12928482	G	A	0.196	0.147	0.046

rs13024657	C	T	-0.296	-0.064	-0.216
rs13050325	A	G	-0.258	-0.106	-0.146
rs13104866	G	A	0.278	0.123	0.151
rs13112725	G	C	-0.297	-0.15	-0.136
rs13192976	A	T	-0.291	0.149	-0.43
rs13205180	C	T	-0.045	-0.143	0.094
rs1322639	G	A	-0.199	0.117	-0.302
rs1333047	A	T	-0.127	0.037	-0.162
rs13403122	C	T	0.25	0.195	0.043
rs13420463	A	G	0.282	0.131	0.159
rs1350100	A	G	0.108	-0.057	0.152
rs137993948	T	G	-0.15	0.056	-0.196
rs139703184	T	A	-0.275	0.018	-0.271
rs1449544	A	C	0.171	-0.035	0.2
rs1475130	T	C	-0.175	0	-0.168
rs147696085	G	A	0.184	-0.122	0.29
rs1544935	T	G	0.18	0.176	0.004
rs1570350	A	G	0.162	-0.042	0.201
rs1630266	G	A	-0.333	-0.252	-0.118
rs168643	T	C	0.196	0.139	0.067
rs169287	C	A	0.007	0.215	-0.19
rs1694068	T	A	-0.245	-0.093	-0.164
rs17046596	A	C	-0.181	-0.151	-0.044
rs17082391	C	G	0.544	0.375	0.196
rs17210898	G	A	0.556	0.351	0.203
rs17286052	A	G	0.36	0.188	0.167
rs17423264	C	T	0.366	0.184	0.166
rs17471509	A	G	0.191	0.056	0.143
rs17617337	C	T	0.13	0.172	-0.046
rs1761870	G	A	0.119	0.175	-0.049
rs17831815	T	C	0.203	0.032	0.176

rs1848797	A	G	0.265	0.191	0.065
rs1876487	A	C	-0.205	-0.162	-0.05
rs1923409	G	A	-0.137	0.024	-0.164
rs1938598	T	C	0.33	0.127	0.21
rs1966203	C	G	-0.105	0.035	-0.143
rs200541	A	G	-0.281	-0.095	-0.175
rs2012714	C	T	-0.222	-0.038	-0.182
rs2034618	C	T	0.137	0.188	-0.052
rs2049814	A	G	-0.152	0.001	-0.153
rs2050663	T	C	-0.229	-0.174	-0.062
rs210156	A	G	-0.149	-0.148	-0.001
rs2178452	G	A	0.259	0.133	0.137
rs2193635	C	T	-0.274	0.073	-0.324
rs2215590	C	T	-0.079	0.09	-0.161
rs2236973	T	C	-0.255	-0.182	-0.088
rs2244643	A	C	-0.146	0.073	-0.219
rs2246438	G	A	0.139	0.16	-0.018
rs2280861	A	G	-0.175	-0.173	-0.01
rs2289125	A	C	-0.274	0.091	-0.347
rs2360970	G	C	-0.215	-0.124	-0.103
rs2393455	C	A	-0.171	-0.02	-0.153
rs2404715	C	T	0.4	0.066	0.338
rs2424908	C	T	-0.196	-0.009	-0.189
rs2440907	G	T	-0.199	-0.061	-0.14
rs2498323	G	A	-0.292	0.021	-0.29
rs2540950	C	T	0.193	0.017	0.172
rs258494	C	G	0.141	0.216	-0.06
rs2585810	G	A	-0.22	-0.117	-0.112
rs2645466	A	C	-0.173	-0.029	-0.152
rs2706110	T	C	0.122	-0.051	0.182
rs273957	C	T	-0.145	0.003	-0.147

rs2759308	G	A	-0.276	-0.136	-0.145
rs2761436	C	T	-0.2	-0.03	-0.154
rs28594215	G	A	-0.133	0.014	-0.144
rs28663144	A	C	-0.694	-0.322	-0.366
rs2899463	T	C	0.189	0.027	0.153
rs2971669	C	T	-0.159	0.015	-0.169
rs3011549	A	C	0.326	0.137	0.174
rs303343	C	T	-0.229	-0.095	-0.122
rs34331990	T	G	-0.096	-0.154	0.036
rs34457140	T	G	-0.183	-0.015	-0.163
rs34489224	C	G	0.108	-0.092	0.185
rs34594435	C	T	0.187	0.183	0.008
rs34872471	T	C	-0.25	-0.043	-0.204
rs35261357	C	T	-0.265	-0.051	-0.216
rs35410524	C	T	-0.305	-0.121	-0.166
rs35565381	T	C	-0.157	-0.14	-0.02
rs35654783	T	C	0.185	0.166	0.02
rs36010659	T	C	0.281	0.042	0.254
rs360158	G	A	-0.301	-0.165	-0.13
rs36061333	C	G	0.107	0.16	-0.047
rs36226649	T	C	-0.156	-0.297	0.149
rs3731818	G	A	0.24	0.117	0.127
rs3749237	G	A	-0.208	-0.165	-0.041
rs3790227	C	A	-0.127	0.11	-0.22
rs3820068	A	G	0.255	0.066	0.199
rs3915499	G	A	0.169	0.02	0.152
rs3923097	T	A	0.345	0.273	0.086
rs4110517	A	G	-0.282	-0.135	-0.148
rs4141663	C	T	0.258	0.126	0.125
rs4292285	T	A	0.23	0.154	0.072
rs4295	C	G	0.24	0.14	0.097

rs4475250	G	A	0.243	0.136	0.097
rs4551692	G	A	-0.419	-0.255	-0.177
rs460105	T	C	0.175	0.178	-0.008
rs4653889	A	G	0.205	0.152	0.057
rs4686683	T	G	-0.183	-0.172	-0.019
rs4709746	C	T	0.249	0.19	0.051
rs4712656	G	C	-0.227	-0.124	-0.094
rs4788913	G	A	-0.283	-0.122	-0.154
rs4803457	T	C	0.081	-0.075	0.149
rs4808569	C	A	0.235	0.162	0.074
rs4923910	G	C	-0.141	-0.178	0.046
rs4972805	C	T	0.184	0.143	0.038
rs4980877	C	T	-0.165	-0.014	-0.162
rs4984497	T	C	0.098	0.148	-0.046
rs507666	G	A	0.025	0.169	-0.145
rs55940751	C	T	0.216	0.087	0.129
rs57400569	G	A	0.244	0.049	0.204
rs58117425	G	A	-0.137	-0.192	0.036
rs6019378	C	T	0.156	0.156	-0.003
rs60199046	A	G	0.24	-0.083	0.313
rs6031435	A	G	-0.214	-0.052	-0.17
rs6060114	T	C	0.259	0.237	0.009
rs6090040	A	C	0.265	0.1	0.15
rs6129880	T	G	0.264	0.174	0.086
rs61448762	G	A	0.358	0.172	0.172
rs61735998	G	T	-0.589	-0.035	-0.519
rs61823001	A	G	0.099	-0.215	0.312
rs61879810	A	G	0.285	0.198	0.079
rs62011052	T	C	-0.207	0.105	-0.28
rs62162674	G	C	0.14	-0.042	0.181
rs62270945	C	T	-0.608	-0.041	-0.534

rs62361303	C	T	0.262	0.015	0.235
rs62524579	G	A	0.244	0.147	0.1
rs6428947	C	G	0.253	0.205	0.047
rs6429422	T	G	-0.158	-0.249	0.075
rs6434404	A	G	0.311	0.125	0.185
rs6479908	C	G	-0.092	-0.142	0.041
rs649472	T	C	0.165	0.01	0.161
rs6595838	G	A	-0.267	-0.133	-0.136
rs66887589	T	C	-0.214	-0.206	-0.026
rs670463	A	G	-0.187	-0.023	-0.158
rs6747874	G	A	-0.163	0.003	-0.171
rs6782694	C	A	-0.178	0.024	-0.19
rs6793656	A	G	-0.197	0.032	-0.224
rs6795735	C	T	0.169	0.159	0.019
rs6803322	C	A	0.228	0.102	0.13
rs685149	A	G	-0.311	-0.098	-0.186
rs6957161	A	G	0.256	0.157	0.103
rs7008914	T	C	0.238	0.104	0.127
rs7019055	A	G	0.18	0.031	0.15
rs7041664	C	A	-0.247	-0.083	-0.166
rs7107356	A	G	-0.309	-0.149	-0.159
rs7116797	A	G	0.311	0.214	0.11
rs7161323	C	T	-0.275	-0.147	-0.132
rs7225219	T	A	-0.248	-0.094	-0.137
rs7255	T	C	-0.164	0.036	-0.205
rs72799341	G	A	-0.031	-0.161	0.132
rs72812846	T	A	0.198	0.202	-0.008
rs72930293	C	T	0.257	0.234	0.03
rs72958213	C	T	0.101	-0.097	0.177
rs7312132	G	C	0.354	0.077	0.283
rs73605614	A	C	-0.175	0.016	-0.177

rs74237369	G	T	0.315	0.162	0.144
rs74482535	C	T	0.27	-0.005	0.271
rs74621754	G	A	0.132	-0.229	0.36
rs7500448	A	G	0.192	-0.135	0.301
rs7546498	G	T	0.257	0.147	0.095
rs7575523	T	G	0.154	0.011	0.142
rs7586597	G	T	-0.072	0.088	-0.154
rs7590201	G	T	-0.216	-0.138	-0.082
rs7591091	T	C	0.079	0.131	-0.055
rs7605066	C	T	0.156	0.004	0.149
rs76398786	C	T	-0.437	-0.382	-0.057
rs76627715	T	C	0.14	0.216	-0.062
rs7665304	A	C	0.22	0.128	0.079
rs7666150	T	C	0.206	0.05	0.15
rs76735299	G	A	-0.429	-0.249	-0.204
rs76785029	C	T	0.324	-0.072	0.379
rs7714219	G	C	-0.143	0.018	-0.164
rs7734334	C	A	-0.162	-0.154	-0.009
rs783621	A	G	0.289	0.082	0.207
rs78378222	T	G	0.336	-0.638	0.875
rs7838781	A	G	0.227	0.048	0.183
rs7856420	G	C	0.13	-0.035	0.159
rs786919	A	G	0.262	0.109	0.156
rs7914287	T	C	-0.202	-0.035	-0.187
rs7927515	C	A	-0.235	-0.084	-0.149
rs7928655	C	G	-0.158	-0.143	-0.014
rs7951348	C	T	-0.252	-0.12	-0.122
rs7977389	T	C	0.381	0.104	0.274
rs7980687	G	A	0.118	0.196	-0.061
rs80073370	A	T	0.404	0.163	0.219
rs8073626	C	T	0.217	0.092	0.122

rs8103992	A	C	0.137	-0.055	0.198
rs8105753	A	C	0.249	0.137	0.107
rs813412	C	T	0.202	0.029	0.178
rs8139817	A	C	-0.155	-0.143	-0.012
rs869396	C	A	0.166	-0.05	0.222
rs8904	G	A	-0.259	-0.082	-0.167
rs893929	G	A	0.233	0.124	0.113
rs903432	A	G	0.387	0.313	0.073
rs917275	A	G	-0.162	0.013	-0.182
rs9303241	T	A	0.204	0.047	0.159
rs9314907	C	T	-0.292	-0.122	-0.16
rs9337951	G	A	-0.156	0.109	-0.265
rs9349379	A	G	0.187	-0.018	0.195
rs937213	T	C	0.256	0.131	0.122
rs956006	C	T	0.158	-0.06	0.215
rs9565436	A	C	-0.303	-0.1	-0.196
rs9662255	C	A	0.2	0.013	0.177
rs9708177	C	T	-0.276	0.018	-0.274
rs9729719	G	A	-0.13	0.053	-0.188
rs9844972	G	C	-0.441	-0.148	-0.296
rs9845655	T	C	0.215	0.161	0.042
rs9864898	C	T	-0.271	-0.19	-0.093
rs9882772	T	C	-0.154	-0.133	-0.018
rs9935770	C	T	0.223	0.111	0.11

Supplementary Table 3 – Pathway Analysis Results From Multi-trait analysis with FDR<0.5

FULL_NAME	NGENES	BETA	BETA _STD	SE	P	fdr
GO_PHOSPHATIDYLINOSITOL_5_PHOSPHATE_BINDING	13	1.18	0.0322	0.258	2.24E-06	0.020
GO_EXTRACELLULAR_MATRIX_STRUCTURAL_CONSTITUENT	149	0.316	0.029	0.0724	6.19E-06	0.027
GO_EXTRACELLULAR_MATRIX_CONSTITUENT_CONFERRING_ELASTICITY	9	1.18	0.0268	0.276	8.91E-06	0.027
GO_MIDDLE_EAR_MORPHOGENESIS	18	0.835	0.0267	0.205	2.27E-05	0.049
GO_ROUNDABOUT_BINDING	5	1.71	0.0288	0.423	2.72E-05	0.049
GO_COLLAGEN_TYPE_IV_TRIMER	4	1.98	0.0298	0.508	5.01E-05	0.075
GO_STRUCTURAL_MOLECULE_ACTIVITY_CONFERRING_ELASTICITY	11	0.933	0.0233	0.256	1.34E-04	0.173
GO_VOLTAGE_GATED_CALCIUM_CHANNEL_ACTIVITY_INVOLVED_IN_CARDIAC_MUSCLE_CELL_ACTION_POTENTIAL	5	1.55	0.0262	0.434	1.72E-04	0.194
GO_REGULATION_OF_VOLTAGE_GATED_CALCIUM_CHANNEL_ACTIVITY	32	0.499	0.0213	0.144	2.69E-04	0.245
GO_EXTRACELLULAR_MATRIX_COMPONENT	45	0.46	0.0232	0.135	3.19E-04	0.245
GO_PHOSPHATIDYLINOSITOL_3_PHOSPHATE_BINDING	34	0.491	0.0216	0.144	3.21E-04	0.245
GO_POSITIVE_REGULATION_OF_CGMP_MEDIATED_SIGNALING	8	0.854	0.0182	0.252	3.49E-04	0.245
GO_ELASTIC_FIBER_ASSEMBLY	7	0.857	0.0171	0.254	3.80E-04	0.245
GO_REGULATION_OF_HIGH_VOLTAGE_GATED_CALCIUM_CHANNEL_ACTIVITY	16	0.68	0.0205	0.203	4.04E-04	0.245
GO_NEGATIVE_REGULATION_OF_RUFFLE_ASSEMBLY	5	1.34	0.0226	0.401	4.14E-04	0.245
GO_90S_PRERIBOSOME	28	0.416	0.0166	0.125	4.34E-04	0.245
GO_REGULATION_OF_CGMP_MEDIATED_SIGNALING	15	0.661	0.0193	0.201	0.001	0.260
GO_MATURATION_OF_SSU_RRNA_FROM_TRICISTRONIC_RRNA_TRANSCRIPT_SSU_RRNA_5_8S_RRNA_LSU_RRNA_	33	0.422	0.0183	0.129	0.001	0.260
GO_MEMBRANE_DEPOLARIZATION_DURING_AV_NODE_CELL_ACTION_POTENTIAL	5	1.37	0.0231	0.43	0.001	0.342
GO_CALCIUM_ION_TRANSMEMBRANE_TRANSPORT_VIA_HIGH_VOLTAGE_GATED_CALCIUM_CHANNEL	12	0.815	0.0213	0.257	0.001	0.342
GO_HIGH_VOLTAGE_GATED_CALCIUM_CHANNEL_ACTIVITY	8	0.976	0.0208	0.315	0.001	0.409
GO_REGULATION_OF_CHOLESTEROL_ESTERIFICATION	11	0.785	0.0196	0.255	0.001	0.409
GO_BASEMENT_MEMBRANE	85	0.302	0.0209	0.0981	0.001	0.409
GO_CGMP_MEDIATED_SIGNALING	31	0.46	0.0193	0.15	0.001	0.409
GO_POSITIVE_REGULATION_OF_LONG_TERM_NEURONAL_SYNAPTIC_PLASTICITY	5	1.31	0.0221	0.432	0.001	0.425
GO_NEGATIVE_REGULATION_OF_MACROPHAGE_CHEMOTAXIS	7	0.902	0.018	0.3	0.001	0.430
GO_ASCENDING_AORTA_DEVELOPMENT	4	1.11	0.0167	0.368	0.001	0.430
GO_NEGATIVE_REGULATION_OF_DNA_DAMAGE_CHECKPOINT	5	1.35	0.0228	0.451	0.001	0.430

GO_POSITIVE_REGULATION_OF_MAST_CELL_ACTIVATION	17	0.664	0.0206	0.223	0.001	0.435
GO_CHORIONIC_TROPHOBLAST_CELL_DIFFERENTIATION	5	1.26	0.0212	0.424	0.001	0.435
GO_ANCHORED_COMPONENT_OF_POSTSYNAPTIC_MEMBRANE	5	1.3	0.022	0.439	0.001	0.435
GO_NEGATIVE_REGULATION_OF_SYSTEMIC_ARTERIAL_BLOOD_PRESSURE	17	0.633	0.0197	0.215	0.002	0.458
GO_POSITIVE_REGULATION_OF_EPIDERMAL_GROWTH_FACTOR_ACTIVATED_RECEPTOR_ACTIVITY	9	0.84	0.019	0.288	0.002	0.483
GO_CLATHRIN_COATED_VESICLE_MEMBRANE	101	0.238	0.018	0.082	0.002	0.483
GO_CALMODULIN_DEPENDENT_PROTEIN_PHOSPHATASE_ACTIVITY	5	1.3	0.022	0.45	0.002	0.483
GO_PRERIBOSOME	68	0.243	0.0151	0.0845	0.002	0.498
GO_EXTRACELLULAR_MATRIX_ASSEMBLY	22	0.481	0.017	0.169	0.002	0.499
GO_SUPEROXIDE_GENERATING_NADPH_OXIDASE_ACTIVATOR_ACTIVITY	6	1.1	0.0203	0.387	0.002	0.499
GO_MODULATION_OF_AGE_RELATED_BEHAVIORAL_DECLINE	7	0.826	0.0165	0.291	0.002	0.499
GO_PEBOW_COMPLEX	5	0.864	0.0146	0.306	0.002	0.499
GO_POSITIVE_REGULATION_OF_HIGH_VOLTAGE_GATED_CALCIUM_CHANNEL_ACTIVITY	5	0.922	0.0155	0.327	0.002	0.499
GO_POSITIVE_REGULATION_OF_SYNAPSE_MATURATION	8	0.85	0.0181	0.302	0.002	0.499
GO_POSITIVE_REGULATION_OF_MAPKKK_CASCADE_BY_FIBROBLAST_GROWTH_FACTOR_RECEPTOR_SIGNALING_PATHWAY	5	1.01	0.017	0.359	0.002	0.499
GO_RNA_CAP_BINDING	18	0.491	0.0157	0.175	0.003	0.499
GO_CALCIUM_DEPENDENT_PROTEIN_SERINE_THREONINE_PHOSPHATASE_ACTIVITY	7	0.868	0.0173	0.311	0.003	0.499
GO_RESPONSE_TO_CORTISOL	5	1.31	0.0221	0.471	0.003	0.499
GO_K63_LINKED_POLYUBIQUITIN_MODIFICATION_DEPENDENT_PROTEIN_BINDING	20	0.475	0.016	0.171	0.003	0.499
GO_PHAGOPHORE_ASSEMBLY_SITE	31	0.412	0.0173	0.148	0.003	0.499
GO_POSITIVE_REGULATION_OF_CATION_CHANNEL_ACTIVITY	61	0.302	0.0177	0.109	0.003	0.499
GO_ROUNDABOUT_SIGNALING_PATHWAY	7	1.06	0.021	0.381	0.003	0.499
GO_POSITIVE_REGULATION_OF_T_CELL_RECEPTOR_SIGNALING_PATHWAY	13	0.582	0.0158	0.211	0.003	0.499
GO_NEGATIVE_REGULATION_OF_CIRCADIAN_SLEEP_WAKE_CYCLE_SLEEP	5	0.934	0.0157	0.338	0.003	0.499
GO_MODIFICATION_OF_POSTSYNAPTIC_ACTIN_CYTOSKELETON	10	0.749	0.0178	0.272	0.003	0.499
GO_NEUROLOGICAL_SYSTEM_PROCESS_INVOLVED_IN_REGULATION_OF_SYSTEMIC_ARTERIAL_BLOOD_PRESSURE	8	0.855	0.0182	0.311	0.003	0.499
GO_NEGATIVE_REGULATION_OF_SODIUM_ION_TRANSMEMBRANE_TRANSPORT	13	0.709	0.0192	0.258	0.003	0.499

Supplementary Table 4 - Associations of Genome-wide Significant SNPs identified in MEGASTROKE with Lacunar Stroke (Europeans) in this analysis

SNP	CHR	BP	RA	OA	RAF	beta	SE	pval	TotalSampleSize	TotalCases
rs12037987	1	113042822	t	c	0.918	0.0423	0.0404	0.2946	248766	5470
rs12124533	1	115657799	t	c	0.2453	-0.0163	0.0246	0.5074	249205	5502
rs880315	1	10796866	t	c	0.652	0.0851	0.0218	9.27E-05	250023	5706
rs13143308	4	111714419	t	g	0.2092	0.0315	0.0251	0.2092	254952	6038
rs17612742	4	148414651	t	c	0.8649	0.0312	0.0306	0.308	249205	5502
rs6825454	4	155501188	t	c	0.7419	0.0251	0.0242	0.2981	249205	5502
rs11957829	5	121515195	a	g	0.8319	-0.129	0.0338	0.0001369	240101	4433
rs6891174	5	172640590	a	g	0.3664	0.017	0.0224	0.4472	249205	5502
rs16896398	6	43262704	a	t	0.7041	0.0194	0.0232	0.4028	249205	5502
rs4959130	6	1356916	a	g	0.1423	-0.1339	0.0291	4.23E-06	254132	5834
rs2107595	7	19049388	a	g	0.164	-0.0214	0.0283	0.4491	250021	5706
rs42039	7	92244422	t	c	0.2458	0.0393	0.0238	0.09818	254952	6038
rs10820405	9	106010237	a	g	0.1898	-0.0149	0.0264	0.5729	250023	5706
rs635634	9	136155000	t	c	0.1903	-0.0081	0.0264	0.7608	253764	5840
rs7859727	9	22102165	t	c	0.4858	-0.0582	0.0203	0.004088	254950	6038
rs2295786	10	105616482	a	t	0.6518	-0.0455	0.0223	0.04108	249205	5502
rs3184504	12	111884608	t	c	0.4678	-0.0958	0.0228	2.67E-05	245847	4969
rs35436	12	115554523	t	c	0.3874	0.0543	0.0219	0.01319	249205	5502
rs7304841	12	20577593	a	c	0.5984	-0.024	0.0222	0.2801	249205	5502
rs9526212	13	47225745	a	g	0.2393	0.0488	0.0238	0.04003	254952	6038
rs4932370	15	91404705	a	g	0.3382	-0.0165	0.0227	0.4676	253771	5671
rs12445022	16	87575332	a	g	0.3376	-0.1212	0.0217	2.48E-08	250025	5706
rs12932445	16	73069888	t	c	0.823	0.0023	0.0273	0.9315	254132	5834
rs11867415	17	1571818	a	g	0.9439	0.1125	0.0496	0.02327	231099	4745
rs2229383	19	10794630	t	g	0.6454	-0.0307	0.0212	0.1487	254946	6038
rs8103309	19	11174935	t	c	0.6471	2.00E-04	0.0211	0.9907	254945	6038

CHR, chromosome; BP, base position; RA, reference allele; OA, other allele; RAF, reference allele frequency; SE, standard error.

Supplementary Table 5 - Associations of Genome-wide Significant SNPs identified in MEGASTROKE with Lacunar Stroke (Transethnic) in this analysis

SNP	CHR	BP	RA	OA	RAF	beta	SE	pval	TotalSampleSize	TotalCases
rs12037987	1	113042822	t	c	0.9205	0.0541	0.0392	0.1678	252954	6223
rs12124533	1	115657799	t	c	0.2375	-0.023	0.024	0.3379	253147	6200
rs880315	1	10796866	t	c	0.6631	0.0739	0.0211	0.0004462	253965	6404
rs13143308	4	111714419	t	g	0.2214	0.0344	0.0235	0.1421	259137	6791
rs17612742	4	148414651	t	c	0.8601	0.024	0.0287	0.4039	253147	6200
rs6825454	4	155501188	t	c	0.7442	0.0269	0.023	0.242	253147	6200
rs11957829	5	121515195	a	g	0.8215	-0.1125	0.0309	0.0002704	244281	5186
rs6891174	5	172640590	a	g	0.3563	0.0159	0.022	0.4701	253147	6200
rs16896398	6	43262704	a	t	0.6655	0.0206	0.022	0.3484	253393	6255
rs4959130	6	1356916	a	g	0.14	-0.1367	0.0288	2.13E-06	258317	6587
rs2107595	7	19049388	a	g	0.1696	-0.0178	0.0265	0.5028	253963	6404
rs42039	7	92244422	t	c	0.2388	0.0389	0.0232	0.09409	259139	6791
rs10820405	9	106010237	a	g	0.1847	-0.0171	0.0258	0.5076	254208	6459
rs635634	9	136155000	t	c	0.1848	-0.0063	0.0256	0.8045	257952	6593
rs7859727	9	22102165	t	c	0.5049	-0.0585	0.0195	0.00273	259133	6791
rs2295786	10	105616482	a	t	0.663	-0.0462	0.0216	0.03241	253392	6255
rs3184504	12	111884608	t	c	0.4555	-0.1015	0.0224	6.07E-06	250035	5722
rs35436	12	115554523	t	c	0.3962	0.0572	0.0207	0.005719	253392	6255
rs7304841	12	20577593	a	c	0.5914	-0.0286	0.0209	0.1723	253147	6200
rs9526212	13	47225745	a	g	0.2532	0.0426	0.0223	0.05629	259137	6791
rs4932370	15	91404705	a	g	0.3621	-0.0267	0.0214	0.2118	257959	6424
rs12445022	16	87575332	a	g	0.3262	-0.1139	0.0211	6.62E-08	253967	6404
rs12932445	16	73069888	t	c	0.8257	0.0097	0.0259	0.7081	258074	6532
rs11867415	17	1571818	a	g	0.7676	0.1117	0.0392	0.004391	235286	5498
rs2229383	19	10794630	t	g	0.6481	-0.0256	0.0202	0.2066	259134	6791
rs8103309	19	11174935	t	c	0.6247	-0.0162	0.02	0.418	259126	6791

CHR, chromosome; BP, base position; RA, reference allele; OA, other allele; RAF, reference allele frequency; SE, standard error.

Supplementary Table 6 - Association Results for 12 SNPs associated with Lacunar Stroke in this analysis in MEGASTROKE All Stroke (European) Analysis

SNP	CHR	BP	RA	OA	RAF	Beta	SE	P	TotalSampleSIze	TotalCases
rs2984613	1	156197380	t	c	0.3579	-0.0641	0.0095	1.72E-11	444629	40453
rs72934535	2	203968973	t	c	0.8936	0.0502	0.0151	0.0009157	442265	40404
rs4621303	3	41839370	a	t	0.1623	-0.0486	0.0127	0.0001339	444629	40453
rs2303655	5	121518378	t	c	0.8135	0.052	0.0122	2.01E-05	442142	39067
rs7766042	6	1366718	t	c	0.8978	-0.0847	0.016	1.24E-07	435591	39527
rs225744	6	142562417	t	c	0.2374	-0.0171	0.0108	0.1131	443916	39978
rs61000833	10	105447838	t	c	0.6032	0.0189	0.0095	0.04757	444629	40453
rs79043147	10	124233181	t	c	0.0642	0.078	0.0188	3.44E-05	429911	40093
rs2293576	11	47434986	a	g	0.3281	-0.0369	0.0097	0.0001362	443926	40528
rs11838776	13	111040681	a	g	0.2855	0.0245	0.0102	0.01622	442255	39854
rs12445022	16	87575332	a	g	0.3346	0.052	0.0098	1.03E-07	438714	39884
rs9958650	18	5389832	a	g	0.8957	-0.0441	0.0157	0.004896	433748	38659

CHR, chromosome; BP, base position; RA, reference allele; OA, other allele; RAF, reference allele frequency; SE, standard error.

Supplementary Table 7 - Association Results for 12 SNPs associated with Lacunar Stroke in this analysis in MEGASTROKE All Stroke (Transethnic) Analysis

SNP	CHR	BP	RA	OA	RAF	Beta	SE	P	TotalSampleSIze	TotalCases
rs2984613	1	156197380	t	c	0.3531	-0.0579	0.0081	1.12E-12	522198	67030
rs72934535	2	203968973	t	c	0.8964	0.0496	0.0148	0.0008144	469963	48195
rs4621303	3	41839370	a	t	0.2063	-0.038	0.0107	0.0003802	522001	67030
rs2303655	5	121518378	t	c	0.7641	0.0384	0.0106	0.0002891	517463	64414
rs7766042	6	1366718	t	c	0.8971	-0.0766	0.0152	4.57E-07	463918	48062
rs225744	6	142562417	t	c	0.224	-0.0113	0.0098	0.2509	521553	66555
rs79043147	10	124233181	t	c	0.0627	0.0754	0.0185	4.64E-05	457617	47884
rs61000833	10	105447838	t	c	0.6055	0.0153	0.0083	0.06535	521069	66473
rs2293576	11	47434986	a	g	0.3195	-0.0311	0.0086	0.0002859	521132	66913
rs11838776	13	111040681	a	g	0.2662	0.0283	0.0092	0.00211	519755	66431
rs12445022	16	87575332	a	g	0.3089	0.0574	0.0089	1.05E-10	516372	66461
rs9958650	18	5389832	a	g	0.8583	-0.0362	0.0128	0.004654	506396	63589

CHR, chromosome; BP, base position; RA, reference allele; OA, other allele; RAF, reference allele frequency; SE, standard error.

Supplementary Table 8 - Association Results for 12 SNPs associated with Lacunar Stroke in this analysis in MEGASTROKE All Ischaemic Stroke (European) Analysis

SNP	CHR	BP	RA	OA	RAF	Beta	SE	P	TotalSampleSIze	TotalCases
rs2984613	1	156197380	t	c	0.358	-0.0582	0.0103	1.76E-08	434418	34217
rs72934535	2	203968973	t	c	0.8933	0.0588	0.0165	0.0003606	421859	34031
rs4621303	3	41839370	a	t	0.1625	-0.0545	0.0139	8.61E-05	432531	34171
rs2303655	5	121518378	t	c	0.813	0.0571	0.0133	1.78E-05	430355	32792
rs7766042	6	1366718	t	c	0.898	-0.0916	0.0176	1.87E-07	411371	32971
rs225744	6	142562417	t	c	0.2377	-0.0216	0.0117	0.06619	424955	33512
rs61000833	10	105447838	t	c	0.6036	0.0199	0.0103	0.05307	434418	34217
rs79043147	10	124233181	t	c	0.064	0.0921	0.0203	5.63E-06	419706	33972
rs2293576	11	47434986	a	g	0.3285	-0.0387	0.0106	0.0002467	432060	34174
rs11838776	13	111040681	a	g	0.2854	0.0212	0.0111	0.0557	432044	33618
rs12445022	16	87575332	a	g	0.3347	0.0555	0.0106	1.81E-07	432044	33618
rs9958650	18	5389832	a	g	0.8956	-0.0376	0.0171	0.02758	413427	32408

CHR, chromosome; BP, base position; RA, reference allele; OA, other allele; RAF, reference allele frequency; SE, standard error.

Supplementary Table 9 - Association Results for 12 SNPs associated with Lacunar Stroke in this analysis in MEGASTROKE All Ischaemic Stroke (Transethnic) Analysis

SNP	CHR	BP	RA	OA	RAF	Beta	SE	P	TotalSampleSIze	TotalCases
rs2984613	1	156197380	t	c	0.3528	-0.0533	0.0086	6.68E-10	511534	60341
rs72934535	2	203968973	t	c	0.8965	0.0576	0.0161	0.0003587	449247	41512
rs4621303	3	41839370	a	t	0.2124	-0.0408	0.0114	0.0003447	509450	60295
rs2303655	5	121518378	t	c	0.757	0.0402	0.0113	0.0003664	505676	58139
rs7766042	6	1366718	t	c	0.8971	-0.0807	0.0165	1.06E-06	439388	41196
rs225744	6	142562417	t	c	0.2224	-0.0127	0.0105	0.2255	502139	59636
rs61000833	10	105447838	t	c	0.6059	0.0159	0.0088	0.07045	509301	59372
rs79043147	10	124233181	t	c	0.0623	0.0892	0.0199	7.64E-06	447102	41453
rs2293576	11	47434986	a	g	0.3183	-0.0318	0.0092	0.0005283	508813	60106
rs11838776	13	111040681	a	g	0.2634	0.0258	0.0099	0.008765	509091	59742
rs12445022	16	87575332	a	g	0.3057	0.0609	0.0095	1.28E-10	509249	59742
rs9958650	18	5389832	a	g	0.8539	-0.0312	0.0135	0.0209	486075	57338

CHR, chromosome; BP, base position; RA, reference allele; OA, other allele; RAF, reference allele frequency; SE, standard error.

Supplementary Table 10 – Association Results for 12 SNPs associated with Lacunar Stroke in this analysis in MEGASTROKE Small Vessel Stroke (European) Analysis

SNP	CHR	BP	Ra	OA	RAF	Beta	SE	P	TotalSampleSIze	TotalCases
rs2984613	1	156197380	t	c	0.3564	-0.0923	0.0238	0.0001044	254558	5386
rs72934535	2	203968973	t	c	0.8916	0.1885	0.0385	9.97E-07	244659	5316
rs4621303	3	41839370	a	t	0.1609	-0.1062	0.0323	0.0009933	254558	5386
rs2303655	5	121518378	t	c	0.8146	0.1077	0.0316	0.0006495	251540	5006
rs7766042	6	1366718	t	c	0.8984	-0.2129	0.0397	7.97E-08	241890	5169
rs225744	6	142562417	t	c	0.2382	-0.0823	0.0271	0.002405	252646	5249
rs61000833	10	105447838	t	c	0.6043	0.0794	0.0239	0.000885	254558	5386
rs79043147	10	124233181	t	c	0.0663	0.1471	0.046	0.001377	242784	5254
rs2293576	11	47434986	a	g	0.3275	-0.0987	0.0243	4.98E-05	254558	5386
rs11838776	13	111040681	a	g	0.2859	0.115	0.0252	4.92E-06	252646	5249
rs12445022	16	87575332	a	g	0.3367	0.1301	0.0244	9.26E-08	252646	5249
rs9958650	18	5389832	a	g	0.8951	-0.0913	0.0392	0.01996	239766	4874

CHR, chromosome; BP, base position; RA, reference allele; OA, other allele; RAF, reference allele frequency; SE, standard error.

Supplementary Table 11 - Association Results for 12 SNPs associated with Lacunar Stroke in this analysis in MEGASTROKE Small Vessel Stroke (Transethnic) Analysis

SNP	CHR	BP	RA	OA	RAF	Beta	SE	P	TotalSampleSIze	TotalCases
rs2984613	1	156197380	t	c	0.354	-0.0819	0.0173	2.29E-06	298777	11710
rs72934535	2	203968973	t	c	0.894	0.1751	0.0379	3.94E-06	248332	5809
rs4621303	3	41839370	a	t	0.2054	-0.0989	0.0232	1.98E-05	298777	11710
rs2303655	5	121518378	t	c	0.7674	0.0951	0.0243	8.99E-05	294514	11110
rs7766042	6	1366718	t	c	0.9	-0.201	0.0381	1.28E-07	245352	5741
rs225744	6	142562417	t	c	0.2052	-0.054	0.0228	0.01814	296590	11529
rs79043147	10	124233181	t	c	0.0663	0.1471	0.046	0.001385	242784	5254
rs61000833	10	105447838	t	c	0.63	0.0467	0.0181	0.009917	297532	11490
rs2293576	11	47434986	a	g	0.3074	-0.064	0.0184	0.0004975	298502	11666
rs11838776	13	111040681	a	g	0.2403	0.1101	0.0207	1.09E-07	296865	11573
rs12445022	16	87575332	a	g	0.2764	0.1198	0.0201	2.55E-09	296590	11529
rs9958650	18	5389832	a	g	0.8551	-0.0705	0.0261	0.006906	282465	10934

CHR, chromosome; BP, base position; RA, reference allele; OA, other allele; RAF, reference allele frequency; SE, standard error.

Supplementary Table 12 – Full Results for 12 Associated Loci, Including BMASS Results

						Lacunar Stroke (European: 6030 Cases, 249,389 Controls)		White Matter Hyperintensities (N=42,310)		MTAG	BMASS
**Nearest Genes**	**CHR**	**BP**	**rsid**	**RA/OA**	**RAF**	**OR(SE)**	**P value**	**Beta(SE)**	**P value**	**P value**	**logBF**
A.											
ICA1L-WDR12-CARF-NBEAL1	2	203,968,973	rs72934535	G/A	0.88	1.25(0.04)	$3.7x10^{-9}$	0.070(0.01)	$2.8x10^{-10}$	$5.3x10^{-16}$	13.9
ULK4	3	41,839,370	rs4621303	T/A	0.83	1.15(0.03)	$1.7x10^{-7}$	0.015(0.01)	0.12	$2.2\ x10^{-7}$*	4.8
SPI1-SLC39A13-PSMC3-RAPSN	11	47,434,986	rs2293576	G/A	0.67	1.14(0.02)	$7.2x10^{-10}$	0.030(0.01)	$3.1x10^{-5}$	$6.4x10^{-13}$	9.9
ZCCHC14	16	87,575,332	rs12445022	A/G	0.34	1.13(0.02)	$2.5x10^{-8}$	0.019(0.01)	0.0078	$3.1x10^{-9}$	6.3
ZBTB14-EPB41L3	18	5,389,832	rs9958650	G/A	0.10	1.18(0.03)	$9.9x10^{-7}$	-0.011(0.01)	0.33	0.0005	3.9
B.											
SLC25A44-PMF1-BGLAP	1	156,197,380	rs2984613	T/C	0.36	1.10(0.02)	$2.5x10^{-5}$	0.037(0.01)	$2.3x10^{-7}$	$8.2x10^{-10}$	7.7
LOX-ZNF474-LOC100505841	5	121,518,378	rs2303655	T/C	0.81	1.14(0.03)	$3.6x10^{-5}$	0.050(0.01)	$1.4x10^{-8}$	$1.9x10^{-10}$	8.7
FOXF2-FOXQ1	6	1,366,718	rs7766042	C/T	0.11	1.17(0.03)	$3.7x10^{-6}$	0.045(0.01)	$7.1x10^{-5}$	$5.2x10^{-9}$	6.3
VTA1-GPR126	6	142,562,417	rs225744	C/T	0.77	1.11(0.03)	$3.5x10^{-5}$	0.037(0.01)	$5.8x10^{-6}$	$9.2x10^{-9}$	6.3
SH3PXD2A	10	105,447,838	rs61000833	T/C	0.60	1.10(0.02)	$1.7x10^{-5}$	0.049(0.01)	$2.0x10^{-12}$	$6.0x10^{-13}$	13.5
HTRA1-ARMS2	10	124,233,181	rs79043147	T/C	0.07	1.21(0.04)	$3.2x10^{-6}$	0.057(0.01)	$1.8x10^{-5}$	$1.6x10^{-9}$	6.9
COL4A2	13	111,040,681	rs11838776	A/G	0.29	1.11(0.02)	$4.3x10^{-6}$	0.050(0.01)	$7.9x10^{-11}$	$7.9x10^{-13}$	11.6

A, Associations reaching genome-wide significance for Lacunar Stroke; B, Associations reaching genome-wide significance in multi-trait analysis; CHR, chromosome; BP, base position; RA, reference allele; OA, other allele; RAF, reference allele frequency; OR, odds ratio; SE, standard error. MTAG, multi-trait analysis of GWAS; BMASS, Bayesian multivariate analysis of summary statistics; logBF, log (Bayes factor); *, As A/T and C/G SNPs are removed by MTAG, results are presented for SNP in highest LD (rs9842261).

Supplementary Table 13 – Association Statistics for 12 Variants Associated with Lacunar Stroke from MRI Confirmed and Standard Phenotyping Groups Separately in European ancestry individuals

SNP	MRI Confirmed (2612 Cases, 27725 Controls)			Standard Phenotyping (3418 Cases, 221,204 Controls)			OR Ratio
	OR	SE	P-value	OR	SE	P-value	
18:5389832:AG	1.169	0.049	1.55E-03	1.231	0.055	1.49E-04	0.950
11:47434986:AG	1.121	0.033	4.44E-04	1.172	0.031	2.01E-07	0.957
6:1366718:CT	1.169	0.053	3.09E-03	1.186	0.048	3.65E-04	0.986
10:105447838:CT	1.095	0.032	3.91E-03	1.103	0.031	1.42E-03	0.993
1:156197380:CT	1.119	0.032	4.59E-04	1.091	0.031	4.50E-03	1.025
2:203968973:CT	1.265	0.053	9.99E-06	1.228	0.052	8.86E-05	1.031
6:142562417:CT	1.132	0.037	7.39E-04	1.093	0.036	1.27E-02	1.036
13:111040681:AG	1.135	0.034	1.66E-04	1.093	0.032	5.95E-03	1.039
10:124233181:CT	1.233	0.058	2.87E-04	1.187	0.058	3.03E-03	1.039
16:87575332:AG	1.170	0.032	6.98E-07	1.104	0.031	1.24E-03	1.060
3:41839370:AT	1.211	0.043	7.40E-06	1.109	0.039	8.29E-03	1.093
5:121518378:CT	1.234	0.042	6.39E-07	1.023	0.051	6.53E-01	1.206

OR, Odds Ratio; SE, standard error; OR Ratio, Ratio of Odds Ratios between MRI Confirmed and Standard Phenotyping groups.

Supplementary Table 14 – Associations of Genome-wide Significant Variants identified in this analysis with Epigenetic markers from Genome wide Association Studies reaching p<5e-8

snp	hg19_coordinates	a1	a2	pmid	tissue	marker	beta	se	p	direction	n
rs12445022	chr16:87575332	A	G	27918535	Whole blood	cg16596957	NA	NA	3.63E-63	-	3841
rs12445022	chr16:87575332	A	G	27918535	Whole blood	cg04245248	NA	NA	1.75E-56	-	3841
rs12445022	chr16:87575332	A	G	27918535	Whole blood	cg08031982	NA	NA	8.59E-55	+	3841
rs12445022	chr16:87575332	A	G	27918535	Whole blood	cg03020503	NA	NA	1.14E-41	+	3841
rs12445022	chr16:87575332	A	G	27918535	Whole blood	cg10312981	NA	NA	3.44E-25	+	3841
rs12445022	chr16:87575332	A	G	27918535	Whole blood	cg00555085	NA	NA	1.02E-24	+	3841
rs12445022	chr16:87575332	A	G	27918535	Whole blood	cg00512563	NA	NA	8.31E-18	+	3841
rs12445022	chr16:87575332	A	G	27918535	Whole blood	cg09880724	NA	NA	1.15E-11	+	3841
rs12445022	chr16:87575332	A	G	27918535	Whole blood	cg04980478	NA	NA	1.68E-09	-	3841
rs12445022	chr16:87575332	A	G	27918535	Whole blood	cg27522573	NA	NA	5.96E-09	+	3841
rs12445022	chr16:87575332	A	G	27918535	Whole blood	cg06750038	NA	NA	1.34E-08	-	3841
rs12445022	chr16:87575332	A	G	27918535	Whole blood	cg16788656	NA	NA	2.17E-08	+	3841
rs12445022	chr16:87575332	A	G	27918535	Whole blood	cg02093708	NA	NA	3.23E-08	+	3841
rs12445022	chr16:87575332	A	G	27918535	Whole blood	cg06655861	NA	NA	3.56E-08	+	3841
rs12445022	chr16:87575332	A	G	27036880	Whole blood	cg04245248	-0.2809	0.04418	3.35E-10	-	837
rs12445022	chr16:87575332	A	G	27036880	Whole blood	cg08031982	0.2917	0.04587	3.32E-10	+	837
rs12445022	chr16:87575332	A	G	27036880	Cord blood	cg04245248	-0.3104	0.04366	2.66E-12	-	771
rs12445022	chr16:87575332	A	G	27036880	Cord blood	cg08031982	0.387	0.04695	7.25E-16	+	771
rs12445022	chr16:87575332	A	G	27036880	Whole blood	cg00555085	0.24	0.0411	7.45E-09	+	834
rs12445022	chr16:87575332	A	G	27036880	Whole blood	cg08031982	0.3391	0.04415	4.50E-14	+	834
rs12445022	chr16:87575332	A	G	27036880	Whole blood	cg08031982	0.2891	0.04841	3.65E-09	+	742
rs72934535	chr2:203968973	C	T	27918535	Whole blood	cg16796381	NA	NA	3.71E-94	+	3841
rs72934535	chr2:203968973	C	T	27918535	Whole blood	cg13521797	NA	NA	4.11E-49	+	3841
rs72934535	chr2:203968973	C	T	27918535	Whole blood	cg14367014	NA	NA	2.36E-25	+	3841
rs72934535	chr2:203968973	C	T	27918535	Whole blood	cg05158606	NA	NA	2.28E-20	-	3841
rs72934535	chr2:203968973	C	T	27918535	Whole blood	cg17681313	NA	NA	6.40E-18	+	3841
rs72934535	chr2:203968973	C	T	27918535	Whole blood	cg26648948	NA	NA	1.39E-13	-	3841
rs72934535	chr2:203968973	C	T	27918535	Whole blood	cg27413025	NA	NA	2.72E-13	+	3841

rs72934535	chr2:203968973	C	T	27036880	Whole blood	cg16796381	0.5507	0.05864	5.51E-20	+	837
rs72934535	chr2:203968973	C	T	27036880	Whole blood	cg16796381	0.4645	0.05783	3.64E-15	+	837
rs72934535	chr2:203968973	C	T	27036880	Cord blood	cg15057621	0.3435	0.05613	1.49E-09	+	771
rs72934535	chr2:203968973	C	T	27036880	Cord blood	cg16796381	0.3774	0.05824	1.64E-10	+	771
rs72934535	chr2:203968973	C	T	27036880	Whole blood	cg15057621	0.395	0.05505	1.59E-12	+	834
rs72934535	chr2:203968973	C	T	27036880	Whole blood	cg16796381	0.5303	0.05639	4.93E-20	+	834
rs72934535	chr2:203968973	C	T	27036880	Whole blood	cg15057621	0.3615	0.05528	1.16E-10	+	742
rs72934535	chr2:203968973	C	T	27036880	Whole blood	cg16796381	0.4292	0.05859	6.26E-13	+	742
rs2293576	chr11:47434986	A	G	27918535	Whole blood	cg13308137	NA	NA	1.04E-57	-	3841
rs2293576	chr11:47434986	A	G	27918535	Whole blood	cg15270729	NA	NA	4.46E-50	+	3841
rs2293576	chr11:47434986	A	G	27918535	Whole blood	cg18512352	NA	NA	2.39E-47	+	3841
rs2293576	chr11:47434986	A	G	27918535	Whole blood	cg04411041	NA	NA	1.30E-35	-	3841
rs2293576	chr11:47434986	A	G	27918535	Whole blood	cg19460817	NA	NA	1.22E-31	-	3841
rs2293576	chr11:47434986	A	G	27918535	Whole blood	cg25953688	NA	NA	1.88E-27	-	3841
rs2293576	chr11:47434986	A	G	27918535	Whole blood	cg20135002	NA	NA	4.20E-27	+	3841
rs2293576	chr11:47434986	A	G	27918535	Whole blood	cg18589874	NA	NA	1.37E-21	-	3841
rs2293576	chr11:47434986	A	G	27918535	Whole blood	cg07698783	NA	NA	1.66E-16	-	3841
rs2293576	chr11:47434986	A	G	27918535	Whole blood	cg12613344	NA	NA	1.15E-15	-	3841
rs2293576	chr11:47434986	A	G	27918535	Whole blood	cg04959790	NA	NA	2.97E-15	-	3841
rs2293576	chr11:47434986	A	G	27918535	Whole blood	cg19668951	NA	NA	3.47E-13	+	3841
rs2293576	chr11:47434986	A	G	27918535	Whole blood	cg05377527	NA	NA	2.79E-12	-	3841
rs2293576	chr11:47434986	A	G	27918535	Whole blood	cg26139080	NA	NA	9.43E-12	+	3841
rs2293576	chr11:47434986	A	G	27918535	Whole blood	cg03393444	NA	NA	1.28E-11	+	3841
rs2293576	chr11:47434986	A	G	27918535	Whole blood	cg17614165	NA	NA	2.50E-10	+	3841
rs2293576	chr11:47434986	A	G	27918535	Whole blood	cg05585544	NA	NA	7.98E-10	+	3841
rs2293576	chr11:47434986	A	G	27918535	Whole blood	cg04692506	NA	NA	9.49E-10	+	3841
rs2293576	chr11:47434986	A	G	27863251	Monocytes	H3K4me1	0.8317	0.1036	9.91E-16	+	172
rs2293576	chr11:47434986	A	G	27863251	Neutrophils	H3K4me1	0.817	0.106	1.30E-14	+	173
rs2293576	chr11:47434986	A	G	27863251	Monocytes	cg20307385	1.029	0.0931	2.27E-28	+	196
rs2293576	chr11:47434986	A	G	27863251	Neutrophils	cg20307385	1.009	0.0931	2.33E-27	+	197
rs2293576	chr11:47434986	A	G	27863251	T cells	cg20307385	1.02	0.1068	1.30E-21	+	132

rs2293576	chr11:47434986	A	G	27036880	Whole blood	cg04959790	-0.26	0.03871	3.47E-11	-	837
rs2293576	chr11:47434986	A	G	27036880	Whole blood	cg07425170	0.3606	0.04411	1.09E-15	+	837
rs2293576	chr11:47434986	A	G	27036880	Whole blood	cg10504702	-0.2761	0.04508	1.40E-09	-	837
rs2293576	chr11:47434986	A	G	27036880	Whole blood	cg13308137	-0.3675	0.04601	4.54E-15	-	837
rs2293576	chr11:47434986	A	G	27036880	Whole blood	cg20307385	0.5318	0.03469	6.54E-47	+	837
rs2293576	chr11:47434986	A	G	27036880	Whole blood	cg07425170	0.5009	0.04371	3.69E-28	+	837
rs2293576	chr11:47434986	A	G	27036880	Whole blood	cg13308137	-0.3323	0.04659	2.30E-12	-	837
rs2293576	chr11:47434986	A	G	27036880	Whole blood	cg20307385	0.5589	0.03333	6.01E-54	+	837
rs2293576	chr11:47434986	A	G	27036880	Cord blood	cg07425170	0.5057	0.04483	2.06E-27	+	771
rs2293576	chr11:47434986	A	G	27036880	Cord blood	cg13308137	-0.3585	0.04737	1.09E-13	-	771
rs2293576	chr11:47434986	A	G	27036880	Cord blood	cg20307385	0.6095	0.03732	1.06E-51	+	771
rs2293576	chr11:47434986	A	G	27036880	Whole blood	cg04959790	-0.2274	0.03993	1.72E-08	-	834
rs2293576	chr11:47434986	A	G	27036880	Whole blood	cg07425170	0.376	0.04238	4.32E-18	+	834
rs2293576	chr11:47434986	A	G	27036880	Whole blood	cg13308137	-0.3618	0.04694	3.66E-14	-	834
rs2293576	chr11:47434986	A	G	27036880	Whole blood	cg20307385	0.5421	0.0328	2.82E-53	+	834
rs2293576	chr11:47434986	A	G	27036880	Whole blood	cg04959790	-0.2294	0.04033	1.85E-08	-	742
rs2293576	chr11:47434986	A	G	27036880	Whole blood	cg07425170	0.4449	0.04356	5.40E-23	+	742
rs2293576	chr11:47434986	A	G	27036880	Whole blood	cg13308137	-0.3662	0.04649	1.19E-14	-	742
rs2293576	chr11:47434986	A	G	27036880	Whole blood	cg20307385	0.5646	0.03633	2.35E-47	+	742
rs4621303	chr3:41839370	A	T	27918535	Whole blood	cg02108620	NA	NA	6.31E-205	-	3841
rs4621303	chr3:41839370	A	T	27918535	Whole blood	cg02648746	NA	NA	4.14E-116	-	3841
rs4621303	chr3:41839370	A	T	27918535	Whole blood	cg19010139	NA	NA	1.58E-95	+	3841
rs4621303	chr3:41839370	A	T	27918535	Whole blood	cg22222413	NA	NA	4.52E-80	+	3841
rs4621303	chr3:41839370	A	T	27918535	Whole blood	cg03022575	NA	NA	5.34E-38	+	3841
rs4621303	chr3:41839370	A	T	27918535	Whole blood	cg04831656	NA	NA	1.10E-32	+	3841
rs4621303	chr3:41839370	A	T	27918535	Whole blood	cg05590619	NA	NA	8.80E-32	+	3841
rs4621303	chr3:41839370	A	T	27918535	Whole blood	cg09809224	NA	NA	4.75E-28	+	3841
rs4621303	chr3:41839370	A	T	27918535	Whole blood	cg12084388	NA	NA	2.70E-26	+	3841
rs4621303	chr3:41839370	A	T	27918535	Whole blood	cg05999828	NA	NA	2.73E-19	+	3841
rs4621303	chr3:41839370	A	T	27918535	Whole blood	cg22988851	NA	NA	2.73E-18	+	3841
rs4621303	chr3:41839370	A	T	27918535	Whole blood	cg17224181	NA	NA	3.45E-18	+	3841

rs4621303	chr3:41839370	A	T	27918535	Whole blood	cg21185355	NA	NA	5.69E-17	-	3841
rs4621303	chr3:41839370	A	T	27918535	Whole blood	cg02527375	NA	NA	8.02E-17	+	3841
rs4621303	chr3:41839370	A	T	27918535	Whole blood	cg04010712	NA	NA	6.42E-12	-	3841
rs4621303	chr3:41839370	A	T	27863251	Monocytes	H3K27ac	-0.8781	0.1484	3.25E-09	-	162
rs4621303	chr3:41839370	A	T	27863251	Neutrophils	H3K27ac	0.8788	0.1395	3.01E-10	+	174
rs4621303	chr3:41839370	A	T	27863251	Monocytes	H3K4me1	-0.8346	0.1407	3.00E-09	-	172
rs4621303	chr3:41839370	A	T	27863251	Monocytes	Percent-splice-in	1.529	0.1147	1.46E-40	+	194
rs4621303	chr3:41839370	A	T	27863251	Monocytes	Percent-splice-in	1.532	0.1144	6.58E-41	+	194
rs4621303	chr3:41839370	A	T	27863251	Neutrophils	Percent-splice-in	1.377	0.1203	2.59E-30	+	192
rs4621303	chr3:41839370	A	T	27863251	Neutrophils	Percent-splice-in	1.372	0.1206	5.25E-30	+	192
rs4621303	chr3:41839370	A	T	27863251	T cells	Percent-splice-in	1.482	0.1232	2.51E-33	+	169
rs4621303	chr3:41839370	A	T	27863251	T cells	Percent-splice-in	1.469	0.1241	2.60E-32	+	169
rs4621303	chr3:41839370	A	T	27863251	T cells	Percent-splice-in	1.066	0.1382	1.23E-14	+	169
rs4621303	chr3:41839370	A	T	27863251	Monocytes	cg02108620	-0.9479	0.1324	8.15E-13	-	196
rs4621303	chr3:41839370	A	T	27863251	Monocytes	cg03022575	1.125	0.1292	3.19E-18	+	196
rs4621303	chr3:41839370	A	T	27863251	Neutrophils	cg02108620	-1.188	0.1267	6.99E-21	-	197
rs4621303	chr3:41839370	A	T	27863251	Neutrophils	cg03022575	0.8301	0.1343	6.30E-10	+	197
rs4621303	chr3:41839370	A	T	27036880	Whole blood	cg02108620	-0.3079	0.04446	8.73E-12	-	837
rs4621303	chr3:41839370	A	T	27036880	Whole blood	cg03022575	0.3323	0.05101	1.25E-10	+	837
rs4621303	chr3:41839370	A	T	27036880	Whole blood	cg02108620	-0.4246	0.04672	8.65E-19	-	837
rs4621303	chr3:41839370	A	T	27036880	Whole blood	cg03022575	0.3111	0.04851	2.49E-10	+	837
rs4621303	chr3:41839370	A	T	27036880	Cord blood	cg02108620	-0.4453	0.05369	4.89E-16	-	771
rs4621303	chr3:41839370	A	T	27036880	Cord blood	cg03022575	0.3089	0.05307	8.61E-09	+	771
rs4621303	chr3:41839370	A	T	27036880	Whole blood	cg02108620	-0.331	0.04343	6.83E-14	-	834
rs4621303	chr3:41839370	A	T	27036880	Whole blood	cg03022575	0.3305	0.0496	4.90E-11	+	834
rs4621303	chr3:41839370	A	T	27036880	Whole blood	cg22222413	0.2735	0.04799	1.66E-08	+	834
rs4621303	chr3:41839370	A	T	27036880	Whole blood	cg02108620	-0.3131	0.04126	9.70E-14	-	742
rs4621303	chr3:41839370	A	T	27036880	Whole blood	cg22222413	0.3191	0.0551	1.03E-08	+	742
rs61000833	chr10:105447838	C	T	27918535	Whole blood	cg16669339	NA	NA	1.10E-93	+	3841
rs61000833	chr10:105447838	C	T	27918535	Whole blood	cg18735015	NA	NA	1.99E-87	+	3841
rs61000833	chr10:105447838	C	T	27918535	Whole blood	cg00492979	NA	NA	6.95E-84	+	3841

rs61000833	chr10:105447838	C	T	27918535	Whole blood	cg19726408	NA	NA	2.47E-79	+	3841
rs61000833	chr10:105447838	C	T	27918535	Whole blood	cg19007269	NA	NA	7.71E-64	+	3841
rs61000833	chr10:105447838	C	T	27918535	Whole blood	cg20669641	NA	NA	8.15E-46	+	3841
rs61000833	chr10:105447838	C	T	27918535	Whole blood	cg00126946	NA	NA	2.27E-44	+	3841
rs61000833	chr10:105447838	C	T	27918535	Whole blood	cg23901918	NA	NA	2.54E-38	+	3841
rs61000833	chr10:105447838	C	T	27918535	Whole blood	cg13634090	NA	NA	2.14E-31	+	3841
rs61000833	chr10:105447838	C	T	27918535	Whole blood	cg12477923	NA	NA	5.13E-26	-	3841
rs61000833	chr10:105447838	C	T	27918535	Whole blood	cg03880642	NA	NA	2.45E-23	-	3841
rs61000833	chr10:105447838	C	T	27918535	Whole blood	cg26550162	NA	NA	4.16E-12	+	3841
rs61000833	chr10:105447838	C	T	27918535	Whole blood	cg07709475	NA	NA	4.54E-12	+	3841
rs61000833	chr10:105447838	C	T	27918535	Whole blood	cg02432295	NA	NA	6.72E-12	+	3841
rs61000833	chr10:105447838	C	T	27918535	Whole blood	cg20386580	NA	NA	2.21E-11	+	3841
rs61000833	chr10:105447838	C	T	27918535	Whole blood	cg02324920	NA	NA	3.09E-10	+	3841
rs61000833	chr10:105447838	C	T	27918535	Whole blood	cg07869830	NA	NA	5.36E-10	+	3841
rs61000833	chr10:105447838	C	T	27918535	Whole blood	cg25066490	NA	NA	7.37E-10	+	3841
rs61000833	chr10:105447838	C	T	27918535	Whole blood	cg26378073	NA	NA	8.65E-10	-	3841
rs61000833	chr10:105447838	C	T	27918535	Whole blood	cg04788957	NA	NA	1.28E-08	+	3841
rs61000833	chr10:105447838	C	T	27863251	Monocytes	cg00126946	0.5842	0.0987	3.30E-09	+	196
rs61000833	chr10:105447838	C	T	27036880	Whole blood	cg00492979	0.3201	0.04118	2.24E-14	+	837
rs61000833	chr10:105447838	C	T	27036880	Whole blood	cg16669339	0.3272	0.04037	1.87E-15	+	837
rs61000833	chr10:105447838	C	T	27036880	Whole blood	cg18735015	0.3473	0.04033	3.58E-17	+	837
rs61000833	chr10:105447838	C	T	27036880	Whole blood	cg19726408	0.3695	0.04016	2.77E-19	+	837
rs61000833	chr10:105447838	C	T	27036880	Whole blood	cg00492979	0.2935	0.04228	8.27E-12	+	837
rs61000833	chr10:105447838	C	T	27036880	Whole blood	cg16669339	0.2943	0.04362	2.96E-11	+	837
rs61000833	chr10:105447838	C	T	27036880	Whole blood	cg18735015	0.3461	0.043	3.17E-15	+	837
rs61000833	chr10:105447838	C	T	27036880	Whole blood	cg19359398	0.2445	0.04324	2.21E-08	+	837
rs61000833	chr10:105447838	C	T	27036880	Whole blood	cg19726408	0.3408	0.04482	8.41E-14	+	837
rs61000833	chr10:105447838	C	T	27036880	Cord blood	cg00492979	0.3125	0.04212	3.14E-13	+	771
rs61000833	chr10:105447838	C	T	27036880	Cord blood	cg16669339	0.3351	0.04205	5.82E-15	+	771
rs61000833	chr10:105447838	C	T	27036880	Cord blood	cg18735015	0.3423	0.04099	3.17E-16	+	771
rs61000833	chr10:105447838	C	T	27036880	Cord blood	cg19726408	0.2909	0.04178	7.17E-12	+	771

rs61000833	chr10:105447838	C	T	27036880	Whole blood	cg00492979	0.3526	0.04223	2.87E-16	+	834
rs61000833	chr10:105447838	C	T	27036880	Whole blood	cg16669339	0.297	0.04193	2.98E-12	+	834
rs61000833	chr10:105447838	C	T	27036880	Whole blood	cg18735015	0.3395	0.04065	2.79E-16	+	834
rs61000833	chr10:105447838	C	T	27036880	Whole blood	cg19726408	0.3578	0.03829	8.30E-20	+	834
rs61000833	chr10:105447838	C	T	27036880	Whole blood	cg16669339	0.2602	0.04629	2.70E-08	+	742
rs61000833	chr10:105447838	C	T	27036880	Whole blood	cg18735015	0.2795	0.04581	1.70E-09	+	742
rs79043147	chr10:124233181	C	T	27918535	Whole blood	cg19863426	NA	NA	8.16E-28	+	3841
rs79043147	chr10:124233181	C	T	27036880	Whole blood	cg03623097	-0.4311	0.07628	2.19E-08	-	837
rs2984613	chr1:156197380	C	T	27918535	Whole blood	cg07384080	NA	NA	9.41E-47	-	3841
rs2984613	chr1:156197380	C	T	27918535	Whole blood	cg20302342	NA	NA	3.20E-36	+	3841
rs2984613	chr1:156197380	C	T	27918535	Whole blood	cg12036877	NA	NA	3.94E-27	+	3841
rs2984613	chr1:156197380	C	T	27918535	Whole blood	cg14500718	NA	NA	1.50E-26	-	3841
rs2984613	chr1:156197380	C	T	27918535	Whole blood	cg11580676	NA	NA	3.40E-26	+	3841
rs2984613	chr1:156197380	C	T	27918535	Whole blood	cg15685922	NA	NA	2.63E-24	+	3841
rs2984613	chr1:156197380	C	T	27918535	Whole blood	cg13282594	NA	NA	2.76E-24	+	3841
rs2984613	chr1:156197380	C	T	27918535	Whole blood	cg25133887	NA	NA	1.75E-23	+	3841
rs2984613	chr1:156197380	C	T	27918535	Whole blood	cg24843609	NA	NA	3.99E-19	+	3841
rs2984613	chr1:156197380	C	T	27918535	Whole blood	cg24674445	NA	NA	1.15E-18	-	3841
rs2984613	chr1:156197380	C	T	27918535	Whole blood	cg14663278	NA	NA	1.28E-18	+	3841
rs2984613	chr1:156197380	C	T	27918535	Whole blood	cg11519176	NA	NA	2.28E-17	+	3841
rs2984613	chr1:156197380	C	T	27918535	Whole blood	cg16558208	NA	NA	1.44E-16	+	3841
rs2984613	chr1:156197380	C	T	27918535	Whole blood	cg05532239	NA	NA	4.89E-15	-	3841
rs2984613	chr1:156197380	C	T	27918535	Whole blood	cg18973101	NA	NA	5.80E-15	+	3841
rs2984613	chr1:156197380	C	T	27918535	Whole blood	cg10585648	NA	NA	4.03E-14	-	3841
rs2984613	chr1:156197380	C	T	27918535	Whole blood	cg17419597	NA	NA	9.61E-14	+	3841
rs2984613	chr1:156197380	C	T	27918535	Whole blood	cg23729443	NA	NA	1.37E-13	+	3841
rs2984613	chr1:156197380	C	T	27918535	Whole blood	cg18993334	NA	NA	2.03E-13	-	3841
rs2984613	chr1:156197380	C	T	27918535	Whole blood	cg09996284	NA	NA	1.09E-12	+	3841
rs2984613	chr1:156197380	C	T	27918535	Whole blood	cg19372602	NA	NA	1.33E-11	+	3841
rs2984613	chr1:156197380	C	T	27918535	Whole blood	cg11075509	NA	NA	6.13E-10	+	3841
rs2984613	chr1:156197380	C	T	27918535	Whole blood	cg18276016	NA	NA	6.03E-09	-	3841

rs2984613	chr1:156197380	C	T	27863251	Monocytes	Percent-splice-in	1.053	0.0858	1.35E-34	+	194
rs2984613	chr1:156197380	C	T	27863251	Neutrophils	Percent-splice-in	0.6197	0.0975	2.08E-10	+	192
rs2984613	chr1:156197380	C	T	27863251	T cells	Percent-splice-in	1.076	0.0908	2.05E-32	+	169
rs2984613	chr1:156197380	C	T	27863251	Monocytes	cg24450063	-1.111	0.0826	3.23E-41	-	196
rs2984613	chr1:156197380	C	T	27863251	Monocytes	cg24849049	-0.7551	0.0945	1.31E-15	-	196
rs2984613	chr1:156197380	C	T	27863251	Monocytes	cg25208724	-1.092	0.0835	4.56E-39	-	196
rs2984613	chr1:156197380	C	T	27863251	Neutrophils	cg24450063	-1.085	0.0839	3.04E-38	-	197
rs2984613	chr1:156197380	C	T	27863251	Neutrophils	cg25208724	-1.038	0.0857	8.90E-34	-	197
rs2984613	chr1:156197380	C	T	27863251	T cells	cg06970220	-0.6947	0.1185	4.58E-09	-	132
rs2984613	chr1:156197380	C	T	27863251	T cells	cg24450063	-1.052	0.1067	6.45E-23	-	132
rs2984613	chr1:156197380	C	T	27863251	T cells	cg25208724	-1.098	0.1045	8.37E-26	-	132
rs2984613	chr1:156197380	C	T	27036880	Whole blood	cg06970220	-0.3518	0.02924	7.51E-31	-	837
rs2984613	chr1:156197380	C	T	27036880	Whole blood	cg24450063	-0.8452	0.03236	2.09E-110	-	837
rs2984613	chr1:156197380	C	T	27036880	Whole blood	cg25208724	-0.8534	0.03193	3.11E-114	-	837
rs2984613	chr1:156197380	C	T	27036880	Whole blood	cg06970220	-0.3362	0.03383	5.81E-22	-	837
rs2984613	chr1:156197380	C	T	27036880	Whole blood	cg24450063	-0.8714	0.03559	4.01E-98	-	837
rs2984613	chr1:156197380	C	T	27036880	Whole blood	cg25208724	-0.9032	0.03555	1.30E-103	-	837
rs2984613	chr1:156197380	C	T	27036880	Cord blood	cg06970220	-0.3984	0.03312	1.14E-30	-	771
rs2984613	chr1:156197380	C	T	27036880	Cord blood	cg07384080	-0.1946	0.0329	5.01E-09	-	771
rs2984613	chr1:156197380	C	T	27036880	Cord blood	cg24450063	-0.8201	0.03511	1.30E-91	-	771
rs2984613	chr1:156197380	C	T	27036880	Cord blood	cg25208724	-0.8496	0.03399	2.17E-101	-	771
rs2984613	chr1:156197380	C	T	27036880	Whole blood	cg06970220	-0.3934	0.03106	9.47E-34	-	834
rs2984613	chr1:156197380	C	T	27036880	Whole blood	cg24450063	-0.8368	0.03215	9.58E-110	-	834
rs2984613	chr1:156197380	C	T	27036880	Whole blood	cg25208724	-0.8583	0.03176	6.05E-116	-	834
rs2984613	chr1:156197380	C	T	27036880	Whole blood	cg06970220	-0.4337	0.03318	2.76E-35	-	742
rs2984613	chr1:156197380	C	T	27036880	Whole blood	cg07384080	-0.2002	0.03199	6.59E-10	-	742
rs2984613	chr1:156197380	C	T	27036880	Whole blood	cg24450063	-0.9013	0.03568	5.07E-102	-	742
rs2984613	chr1:156197380	C	T	27036880	Whole blood	cg25208724	-0.8736	0.03697	2.07E-92	-	742
rs11838776	chr13:111040681	A	G	27918535	Whole blood	cg05272587	NA	NA	2.50E-256	-	3841
rs11838776	chr13:111040681	A	G	27918535	Whole blood	cg15455643	NA	NA	9.68E-167	+	3841
rs11838776	chr13:111040681	A	G	27918535	Whole blood	cg06243866	NA	NA	2.41E-112	-	3841

rs11838776	chr13:111040681	A	G	27918535	Whole blood	cg03482866	NA	NA	4.37E-108	+	3841
rs11838776	chr13:111040681	A	G	27918535	Whole blood	cg08009184	NA	NA	1.93E-56	-	3841
rs11838776	chr13:111040681	A	G	27918535	Whole blood	cg08959039	NA	NA	4.09E-49	-	3841
rs11838776	chr13:111040681	A	G	27918535	Whole blood	cg24844377	NA	NA	4.86E-32	+	3841
rs11838776	chr13:111040681	A	G	27918535	Whole blood	cg08556772	NA	NA	3.47E-24	-	3841
rs11838776	chr13:111040681	A	G	27918535	Whole blood	cg18114040	NA	NA	7.25E-16	-	3841
rs11838776	chr13:111040681	A	G	27918535	Whole blood	cg07980027	NA	NA	2.99E-13	-	3841
rs11838776	chr13:111040681	A	G	27918535	Whole blood	cg24371954	NA	NA	4.55E-13	+	3841
rs11838776	chr13:111040681	A	G	27918535	Whole blood	cg14534089	NA	NA	1.43E-12	-	3841
rs11838776	chr13:111040681	A	G	27918535	Whole blood	cg04819250	NA	NA	9.76E-12	+	3841
rs11838776	chr13:111040681	A	G	27918535	Whole blood	cg02577394	NA	NA	1.55E-11	+	3841
rs11838776	chr13:111040681	A	G	27918535	Whole blood	cg11096515	NA	NA	1.56E-09	-	3841
rs11838776	chr13:111040681	A	G	27918535	Whole blood	cg13153710	NA	NA	2.35E-09	-	3841
rs11838776	chr13:111040681	A	G	27918535	Whole blood	cg11438287	NA	NA	6.92E-09	-	3841
rs11838776	chr13:111040681	A	G	27918535	Whole blood	cg03178404	NA	NA	1.28E-08	-	3841
rs11838776	chr13:111040681	A	G	27863251	Monocytes	cg06243866	-0.7364	0.1034	1.09E-12	-	196
rs11838776	chr13:111040681	A	G	27863251	Monocytes	cg12573318	0.7788	0.1024	2.84E-14	+	196
rs11838776	chr13:111040681	A	G	27863251	Monocytes	cg15455643	0.6236	0.1059	3.95E-09	+	196
rs11838776	chr13:111040681	A	G	27863251	Monocytes	cg26053697	0.8407	0.1011	9.00E-17	+	196
rs11838776	chr13:111040681	A	G	27863251	Neutrophils	cg00994389	0.6606	0.105	3.20E-10	+	197
rs11838776	chr13:111040681	A	G	27863251	Neutrophils	cg12573318	0.7196	0.1038	4.07E-12	+	197
rs11838776	chr13:111040681	A	G	27863251	Neutrophils	cg15455643	0.605	0.1059	1.13E-08	+	197
rs11838776	chr13:111040681	A	G	27863251	Neutrophils	cg26053697	0.8348	0.1009	1.26E-16	+	197
rs11838776	chr13:111040681	A	G	27863251	T cells	cg00994389	0.8049	0.1281	3.28E-10	+	132
rs11838776	chr13:111040681	A	G	27863251	T cells	cg12573318	0.8945	0.1255	1.04E-12	+	132
rs11838776	chr13:111040681	A	G	27863251	T cells	cg15455643	0.7789	0.1293	1.73E-09	+	132
rs11838776	chr13:111040681	A	G	27863251	T cells	cg26053697	0.9177	0.1252	2.28E-13	+	132
rs11838776	chr13:111040681	A	G	27036880	Whole blood	cg03482866	0.372	0.04533	8.53E-16	+	837
rs11838776	chr13:111040681	A	G	27036880	Whole blood	cg05272587	-0.5437	0.04434	6.69E-32	-	837
rs11838776	chr13:111040681	A	G	27036880	Whole blood	cg06243866	-0.3566	0.04437	3.14E-15	-	837
rs11838776	chr13:111040681	A	G	27036880	Whole blood	cg08009184	-0.2843	0.04739	2.97E-09	-	837

rs11838776	chr13:111040681	A	G	27036880	Whole blood	cg08959039	-0.2665	0.04759	2.92E-08	-	837
rs11838776	chr13:111040681	A	G	27036880	Whole blood	cg15455643	0.5009	0.04411	6.73E-28	+	837
rs11838776	chr13:111040681	A	G	27036880	Whole blood	cg26053697	0.6779	0.04128	1.01E-52	+	837
rs11838776	chr13:111040681	A	G	27036880	Whole blood	cg03482866	0.5567	0.0457	2.60E-31	+	837
rs11838776	chr13:111040681	A	G	27036880	Whole blood	cg05272587	-0.5228	0.04555	3.12E-28	-	837
rs11838776	chr13:111040681	A	G	27036880	Whole blood	cg06243866	-0.273	0.04554	3.17E-09	-	837
rs11838776	chr13:111040681	A	G	27036880	Whole blood	cg08959039	-0.3076	0.05029	1.52E-09	-	837
rs11838776	chr13:111040681	A	G	27036880	Whole blood	cg15455643	0.6115	0.04428	7.00E-39	+	837
rs11838776	chr13:111040681	A	G	27036880	Whole blood	cg26053697	0.7287	0.04186	2.08E-57	+	837
rs11838776	chr13:111040681	A	G	27036880	Cord blood	cg03482866	0.4402	0.049	2.00E-18	+	771
rs11838776	chr13:111040681	A	G	27036880	Cord blood	cg05272587	-0.3946	0.04865	1.98E-15	-	771
rs11838776	chr13:111040681	A	G	27036880	Cord blood	cg06243866	-0.3418	0.04761	1.66E-12	-	771
rs11838776	chr13:111040681	A	G	27036880	Cord blood	cg15455643	0.5924	0.04636	4.74E-34	+	771
rs11838776	chr13:111040681	A	G	27036880	Cord blood	cg26053697	0.6994	0.04466	3.47E-48	+	771
rs11838776	chr13:111040681	A	G	27036880	Whole blood	cg03482866	0.4539	0.04608	9.97E-22	+	834
rs11838776	chr13:111040681	A	G	27036880	Whole blood	cg05272587	-0.5137	0.04467	1.66E-28	-	834
rs11838776	chr13:111040681	A	G	27036880	Whole blood	cg06243866	-0.3231	0.04357	2.99E-13	-	834
rs11838776	chr13:111040681	A	G	27036880	Whole blood	cg08009184	-0.2977	0.04667	2.98E-10	-	834
rs11838776	chr13:111040681	A	G	27036880	Whole blood	cg15455643	0.5588	0.04294	2.27E-35	+	834
rs11838776	chr13:111040681	A	G	27036880	Whole blood	cg26053697	0.7431	0.03997	8.46E-65	+	834
rs11838776	chr13:111040681	A	G	27036880	Whole blood	cg03482866	0.5014	0.04867	2.41E-23	+	742
rs11838776	chr13:111040681	A	G	27036880	Whole blood	cg05272587	-0.442	0.04716	8.38E-20	-	742
rs11838776	chr13:111040681	A	G	27036880	Whole blood	cg06243866	-0.3124	0.04701	5.85E-11	-	742
rs11838776	chr13:111040681	A	G	27036880	Whole blood	cg15455643	0.6003	0.04763	3.85E-33	+	742
rs11838776	chr13:111040681	A	G	27036880	Whole blood	cg26053697	0.6574	0.0441	3.89E-44	+	742
rs2303655	chr5:121518378	T	C	27918535	Whole blood	cg02601475	NA	NA	7.44E-120	+	3841
rs2303655	chr5:121518378	T	C	27918535	Whole blood	cg22282672	NA	NA	1.34E-44	+	3841
rs2303655	chr5:121518378	T	C	27918535	Whole blood	cg11560600	NA	NA	2.32E-33	+	3841
rs2303655	chr5:121518378	T	C	27918535	Whole blood	cg18637698	NA	NA	8.15E-24	-	3841
rs2303655	chr5:121518378	T	C	27918535	Whole blood	cg05982301	NA	NA	4.46E-23	-	3841
rs2303655	chr5:121518378	T	C	27918535	Whole blood	cg03553758	NA	NA	6.72E-21	+	3841

rs2303655	chr5:121518378	T	C	27918535	Whole blood	cg24150325	NA	NA	7.28E-13	-	3841
rs2303655	chr5:121518378	T	C	27918535	Whole blood	cg19083497	NA	NA	2.37E-11	+	3841
rs2303655	chr5:121518378	T	C	27918535	Whole blood	cg04747322	NA	NA	6.02E-11	-	3841
rs2303655	chr5:121518378	T	C	27918535	Whole blood	cg01790628	NA	NA	2.92E-10	-	3841
rs2303655	chr5:121518378	T	C	27918535	Whole blood	cg00637695	NA	NA	9.00E-10	-	3841
rs2303655	chr5:121518378	T	C	27918535	Whole blood	cg07195301	NA	NA	1.72E-09	+	3841
rs2303655	chr5:121518378	T	C	27918535	Whole blood	cg14122403	NA	NA	4.69E-08	-	3841
rs2303655	chr5:121518378	T	C	27863251	Monocytes	cg22282672	0.7513	0.1342	2.15E-08	+	196
rs2303655	chr5:121518378	T	C	27863251	Neutrophils	cg02601475	0.7346	0.1347	4.95E-08	+	197
rs2303655	chr5:121518378	T	C	27036880	Whole blood	cg02601475	0.4891	0.05076	6.54E-21	+	837
rs2303655	chr5:121518378	T	C	27036880	Whole blood	cg02601475	0.6742	0.05203	7.55E-35	+	837
rs2303655	chr5:121518378	T	C	27036880	Cord blood	cg02601475	0.6112	0.05424	2.27E-27	+	771
rs2303655	chr5:121518378	T	C	27036880	Whole blood	cg02601475	0.4802	0.04944	3.40E-21	+	834
rs2303655	chr5:121518378	T	C	27036880	Whole blood	cg02601475	0.4402	0.051	3.67E-17	+	742
rs7766042	chr6:1366718	C	T	27918535	Whole blood	cg15105476	NA	NA	2.31E-267	-	3841
rs7766042	chr6:1366718	C	T	27918535	Whole blood	cg00664405	NA	NA	1.71E-25	+	3841
rs7766042	chr6:1366718	C	T	27918535	Whole blood	cg02359433	NA	NA	3.63E-24	+	3841
rs7766042	chr6:1366718	C	T	27918535	Whole blood	cg05964950	NA	NA	7.20E-12	+	3841
rs7766042	chr6:1366718	C	T	27918535	Whole blood	cg05804139	NA	NA	3.95E-10	-	3841
rs7766042	chr6:1366718	C	T	27918535	Whole blood	cg01764198	NA	NA	2.96E-08	-	3841
rs225744	chr6:142562417	C	T	27918535	Whole blood	cg26365690	NA	NA	7.39E-55	-	3841
rs225744	chr6:142562417	C	T	27918535	Whole blood	cg24924958	NA	NA	7.15E-48	-	3841
rs225744	chr6:142562417	C	T	27918535	Whole blood	cg21460020	NA	NA	1.17E-42	-	3841
rs225744	chr6:142562417	C	T	27918535	Whole blood	cg13202845	NA	NA	2.59E-34	-	3841
rs225744	chr6:142562417	C	T	27918535	Whole blood	cg23833425	NA	NA	2.27E-32	+	3841
rs225744	chr6:142562417	C	T	27918535	Whole blood	cg17345081	NA	NA	4.32E-15	-	3841
rs225744	chr6:142562417	C	T	27918535	Whole blood	cg18425786	NA	NA	8.29E-12	-	3841
rs225744	chr6:142562417	C	T	27918535	Whole blood	cg13379208	NA	NA	1.13E-08	+	3841
rs225744	chr6:142562417	C	T	27918535	Whole blood	cg17256157	NA	NA	1.21E-08	-	3841
rs225744	chr6:142562417	C	T	27036880	Whole blood	cg21460020	-0.273	0.04789	1.65E-08	-	837
rs225744	chr6:142562417	C	T	27036880	Whole blood	cg24924958	-0.3434	0.0472	7.90E-13	-	837

rs225744	chr6:142562417	C	T	27036880	Whole blood	cg24924958	-0.3765	0.05139	5.99E-13	-	837
rs225744	chr6:142562417	C	T	27036880	Cord blood	cg24924958	-0.3226	0.05091	4.00E-10	-	771
rs225744	chr6:142562417	C	T	27036880	Whole blood	cg24924958	-0.3102	0.04615	3.33E-11	-	834
rs225744	chr6:142562417	C	T	27036880	Whole blood	cg26365690	-0.2729	0.04675	7.61E-09	-	834
rs225744	chr6:142562417	C	T	27036880	Whole blood	cg24924958	-0.3402	0.05078	4.17E-11	-	742

Supplementary Table 15 – Potentially Druggable Genes Identified in DGIdb database

search_term	match_term	match_type	category	sources
COL4A2	COL4A2	Definite	DRUGGABLE GENOME	HingoraniCasas
ULK4	ULK4	Definite	SERINE THREONINE KINASE	dGene|GuideToPharmacologyGenes|GO
ULK4	ULK4	Definite	DRUGGABLE GENOME	dGene|RussLampel|HingoraniCasas
ULK4	ULK4	Definite	KINASE	GuideToPharmacologyGenes|GO
SPI1	SPI1	Definite	HISTONE MODIFICATION	GO
SPI1	SPI1	Definite	TRANSCRIPTION FACTOR BINDING	GO
SLC39A13	SLC39A13	Definite	TRANSPORTER	GuideToPharmacologyGenes|GO
PSMC3	PSMC3	Definite	TUMOR SUPPRESSOR	GO
PSMC3	PSMC3	Definite	TRANSCRIPTION FACTOR BINDING	GO
SLC25A44	SLC25A44	Definite	TRANSPORTER	GO|GuideToPharmacologyGenes
SLC25A44	SLC25A44	Definite	DRUGGABLE GENOME	RussLampel
PMF1	PMF1	Definite	TRANSCRIPTION FACTOR COMPLEX	GO
BGLAP	BGLAP	Definite	DRUGGABLE GENOME	HingoraniCasas
BGLAP	BGLAP	Definite	DRUG RESISTANCE	GO
LOX	LOX	Definite	DRUG RESISTANCE	GO
LOX	LOX	Definite	DRUGGABLE GENOME	HingoraniCasas
FOXF2	FOXF2	Definite	TRANSCRIPTION FACTOR COMPLEX	GO
FOXF2	FOXF2	Definite	TRANSCRIPTION FACTOR BINDING	GO
HTRA1	HTRA1	Definite	DRUGGABLE GENOME	HingoraniCasas|dGene|RussLampel
HTRA1	HTRA1	Definite	PROTEASE	dGene|GO

Description of Study Populations

UK-young lacunar stroke DNA resource (DNA Lacunar)

A total of 1,029 Caucasian patients with lacunar stroke, aged ≤70 years, were recruited from 72 specialist's stroke centres throughout the UK between 2002 and 2012, as part of the Young Lacunar Stroke DNA Resource. DNA samples were available in 930 patients. An additional 82 Caucasian patients of all ages with lacunar stroke were recruited from St. George's Hospital, London as part of the GENESIS study.83Lacunar stroke was defined as a clinical lacunar syndrome, with an anatomically compatible lesion on MRI (subcortical infarct ≤15 mm in diameter). All patients underwent full stroke investigation including brain MRI, imaging of the carotid arteries and ECG. Echocardiography was performed when appropriate. All MRIs and clinical histories were reviewed centrally by one physician. Exclusion criteria were: stenosis >50% in the extra-or intracranial cerebral vessels, or previous carotid endarterectomy; cardioembolic source of stroke, defined according to the TOAST (Trial of Org 10172 in Acute Stroke Treatment) criteria as high or moderate probability; cortical infarct on MRI; subcortical infarct > 15mm in diameter, as these can be caused by embolic mechanisms (striatocapsular infarcts); any other specific cause of stroke (e.g. lupus anticoagulant, cerebral vasculitis, dissection, monogenic cause of stroke). All cases were screened for NOTCH3 CADASIL and Fabry disease mutations and positive cases excluded. Unrelated Caucasian controls, free of clinical cerebrovascular disease, were obtained by random sampling, stratified for age and sex, from general practice lists from the same geographical location as the patients. All patients and controls underwent a standardized clinical assessment and completed a standardized study questionnaire. MRI was not performed in controls. The study was approved by the Multi-Centre Research Ethics Committee (04/MRE00/36) and informed consent was obtained from all participants.

DNA Lacunar 2

DNA Lacunar 2 is a prospective study that recruits patients with MRI confirmed lacunar stroke from 54 hospital within the UK. Patients are recruited from in and out-patient stroke, neurology and medical services. For entry patients need to have a clinical lacunar stroke with an anatomically corresponding lacunar infarct confirmed on MRI, with this infarct confirmed on diffusion weighted imaging for MRI performed in the lacunar stroke should have occurred within two years of recruitment, and the MRI should have been performed within one year of stroke. Exclusion criteria included: Patient unwilling or unable to consent; Any other potential coexistent cause of white matter disease (e.g. demyelination); Any cause of stroke other than SVD including cerebral large artery disease >50%, cardioembolic source- classified as moderate on TOAST classification or a subcortical infarct > 1.5cm diameter as these striatocapsular infarcts are often due to emboli; age <18 years; MRI scan of inadequate quality to allow image analysis. For inclusion patients needed to have, as a minimum, the following routine clinical investigations performed to allow determination of whether the patients meet the above inclusion/exclusion criteria: brain MRI; Imaging of the extracerebral arteries with Doppler ultrasound, or CT or MR angiography; and electrocardiography. For posterior circulation lacunes including thalamic lacunes, a CTA or MRA was required to allow exclusion of vertebro-basilar stenosis. All patients were reviewed in the co-ordinating

centre with original MRI scans to confirmed eligibility by a neurologist. The study was approved by the East of England - Cambridge Central Research Ethics Committee (16/EE/0201).

SGUL Stroke Register

This study recruited patients attending cerebrovascular services at St. George's Hospital, London between 2007-2011. All patients had clinically relevant diagnostic workup performed, including brain imaging with magnetic resonance imaging (MRI) as well as ancillary diagnostic investigations including duplex ultrasonography of the carotid and vertebral arteries, echocardiography, Holter monitoring, magnetic resonance angiography (MRA), CT-angiography (CTA) and blood tests

GENESIS

This study recruited patients attending cerebrovascular services at St. George's Hospital, London between 2011-2013. All patients had clinically relevant diagnostic workup performed, including brain imaging with magnetic resonance imaging (MRI) as well as ancillary diagnostic investigations including duplex ultrasonography of the carotid and vertebral arteries, echocardiography, Holter monitoring, magnetic resonance angiography (MRA), CT-angiography (CTA) and blood tests.

PRESERVE

The PRESERVE study was a 2-year, multicenter, randomized clinical trial that tests standard vs intensive BP treatment regimens in patients with severe SVD on outcomes of WM disease (assessed by diffusion-tensor imaging) and cognition. Patients with clinical and radiological features of cerebral small vessel disease were recruited from sites in the UK. Seventy patients with hypertension and with magnetic resonance imaging–confirmed symptomatic lacunar infarct and confluent white matter hyperintensities were recruited between February 29, 2012, and October 21, 2015.

Edinburgh Mild Stroke Study

The Edinburgh Mild Stroke Study (MSS) is a prospective study of patients with recent (within 3 months) clinical lacunar or mild cortical ischaemic stroke. All patients were assessed by an experienced stroke physician. Patients underwent investigations for stroke as indicated including magnetic resonance imaging (MRI) at presentation. Patients had diagnostic brain MRI at presentation, on a 1.5 T MR scanner (Signa LX; General Electric, GE Company, Fair- field, CT, US) with 22 mT/m maximum strength gradients. Diagnostic MRI included axial diffusion-weighted, T2-weighted, fluid-attenuated inversion recovery (FLAIR) and gradient echo sequences.

Australian Stroke Genetics Collaborative

Stroke cases comprised European-ancestry patients admitted to four clinical centres across Australia (The Neurosciences Department at Gosford Hospital, Gosford, New South Wales (NSW); the Neurology Department at John Hunter Hospital, Newcastle, NSW; The Queen Elizabeth Hospital, Adelaide; and the Royal Perth Hospital, Perth) between 2003 and 2008. Stroke was defined by WHO criteria as a sudden focal neurologic deficit of vascular origin, lasting more than 24 hours and confirmed by brain imaging. Other investigative tests such as electrocardiogram, carotid Doppler and trans-oesophageal echocardiogram were conducted to define stroke aetiology as clinically appropriate.

Leuven Stroke Genetics Study

We enrolled patients with a diagnosis of stroke admitted to the stroke unit of the University Hospitals Leuven (Belgium) in the Leuven Stroke Genetics Study (LSGS) between 2013 and 2017. All participants from the LSGS study underwent brain imaging and full diagnostic workup including extra- and intracranial vessel imaging and cardiac examination (echocardiography and ambulatory ECG monitoring) in all patients. Based on clinical presentation and results from the diagnostic work-up, cases were classified into ischemic stroke etiologic subtypes. In this study only patients presenting with a lacunar infarct on diffusion weighted imaging were included.

Massachusetts General Hospital (MGH)

Massachusetts General Hospital enrolls subjects as part of a single-center prospective cohort study of patients with ischemic stroke aged ≥18 years admitted to the Massachusetts General Hospital Stroke Unit (Boston, MA, U.S.A.) after presenting to the emergency department within 24 hours of symptom onset. Ischemic stroke is defined as a clinical syndrome of any duration associated with a radiographically proven acute infarct consistent with a vascular pattern of involvement and without radiographic evidence of a demyelinating or neoplastic disease or other structural disease, including vasculitis, subacute bacterial endocarditis, vasospasm due to subarachnoid hemorrhage or cocaine abuse, or primary intracerebral hemorrhage. Subjects included in the present study were recruited under the Genes Affecting Stroke Risk and Outcomes Study (GASROS) from 2000-2011, and under the Biorepository for Neurologic Injury between 2012 and 2017. Diagnosis of acute cerebral ischemia was confirmed for all subjects in this study using MRI completed within 48 hours of symptom onset. Expert reviewers subtyped ischemic strokes by systematic medical record review using the TOAST and CCS systems. Small artery occlusion stroke cases included in the present study were not previously genotyped or included under the NINDS-Stroke Genetics Network (SiGN) publication.

South London Ethnicity and Stroke Study (SLESS)

The South London Ethnicity and Stroke Study (SLESS) is a prospective study begun in 1999 that has recruited consecutive black patients with stroke from a contiguous catchment area covered by 3 hospitals in South London (Guy’s and St Thomas’ Hospitals, King’s College Hospital, and St George’s Hospital). Ethnicity was defined according to the UK Census 2001 definition and classified as Black African or Black Caribbean. Recruitment of black controls was done by random selection from General

Practice lists in the catchment areas of St George's, Guys and St Thomas, and King's College Hospital between 1999 and 2012. Potential controls were selected from age and gender strata matched to stroke cases. Furthermore controls were identified within St George's University of London and St George's Hospital staff and contacted via email. Additionally, posters inviting healthy Black African and Black Caribbean individuals were displayed in local leisure centres, General Practice surgeries, churches and communities centres within the same catchment area as that of the cases. The study was reviewed and approved by the Wandsworth Local Research Ethics Committee, and informed consent was obtained from all participants. One consultant neurologist performed stroke subtyping using data collected on a standard proforma with additional review of all original brain imaging in all patients, as well as review of original notes when necessary. The pathophysiological Trial of Org 10172 in Acute Stroke Treatment (TOAST) subtyping classification was used for subtyping of ischemic stroke. Stroke ascertainment in SLESS: One consultant neurologist performed stroke subtyping using data collected on a standard proforma with additional review of all original brain imaging in all patients, as well as review of original notes when necessary. The pathophysiological Trial of Org 10172 in Acute Stroke Treatment (TOAST) subtyping classification was used for subtyping of ischemic stroke.

Understanding Society

The United Kingdom Household Longitudinal Study (UKHLS), also known as Understanding Society (https://www.understandingsociety.ac.uk) is a longitudinal panel survey of 40,000 UK households (England, Scotland, Wales and Northern Ireland) representative of the UK population. Participants are surveyed annually since 2009 and contribute information relating to their socioeconomic circumstances, attitudes, and behaviours via a computer assisted interview. The study includes phenotypical data for a representative sample of participants for a wide range of social and economic indicators as well as a biological sample collection encompassing biometric, physiological, biochemical, and haematological measurements and self-reported medical history and medication use. The United Kingdom Household Longitudinal Study has been approved by the University of Essex Ethics Committee and informed consent was obtained from every participant. For a subset of individuals who took part in a nurse health assessment, blood samples were taken and genomic DNA extracted. Of these, 10,484 samples were genotyped at the Wellcome Trust Sanger Institute using the Illumina Infinium HumanCoreExome-12 v1.0BeadChip.

UK Biobank

UK Biobank is a long-term study of 500,000 UK participants aged 40-69 years at recruitment covering genetic, lifestyle and health outcome data (PMID: 25826379). In this analysis, South Asian samples from UK Biobank were used as controls for analysis of South Asian lacunar stroke patients. Samples were matched based on principal components analysis.

Wellcome Trust Case-Control Consortium 2 (WTCCC2)

The WTCCC2 samples were genotyped as part of the WTCCC 2 ischemic stroke study. Stroke cases were recruited from three centres in the UK (St. George's University London, Oxford and Edinburgh) and one centre in Germany, University and Klinikum Großhadern, Ludwig-Maximilians-University, Munich.
WTCCC2-UK: The St George's Stroke Study consecutively recruited ischemic stroke patients attending cerebrovascular services in London between 1995 and 2008. All patients had clinically relevant diagnostic workup performed, including brain imaging with computed tomography (CT) and/or magnetic resonance imaging (MRI) as well as ancillary diagnostic investigations including duplex ultrasonography of the carotid and vertebral arteries, echocardiography, Holter monitoring, magnetic resonance angiography (MRA), CT-angiography (CTA) and blood tests. The Oxford Vascular Study recruited patients with acute ischemic stroke or transient ischemic attack (TIA) with evidence of infarction on brain imaging between 2002 and 2008 as part of a population-based stud. All cases were phenotyped by one experienced stroke neurologist with review of original imaging. The Edinburgh Stroke Study prospectively recruited consecutive stroke inpatients and outpatients between 2002 and 2005. An experienced stroke physician assessed each patient as soon as possible after the stroke, prospectively recording demographic and clinical details, including vascular risk factors and results of brain imaging and other investigations
WTCCC2-Germany: The Munich study recruited consecutively between 2002 and 2008, from a single Stroke Unit with a high rate of MR imaging (>80%) (n=1383). All subjects were over 18 years of age, of self-reported European ancestry and with a diagnosis of ischemic stroke classified according to TOAST by an experienced neurologist or stroke physician. All patients had brain imaging as well as ancillary diagnostic investigations where clinically relevant.
Controls for the UK samples were drawn from shared WTCCC controls obtained from the 1958 Birth Cohort. This is a prospectively collected cohort of individuals born in 1958 (http://www.b58cgene.sgul.ac.uk/), and ascertained as part of the national child development study (http://www.cls.ioe.ac.uk/studies.asp?section=000100020003). Data from this cohort are available as a common control set for a number of genetic and epidemiological studies. For the German samples controls were Caucasians of German origin participating into the population KORAgen study (www.gsf.de/kora/en/english.html). This survey represents a gender- and age stratified random sample of all German residents of the Augsburg area and consists of individuals 25 to 74 years of age, with about 300 subjects for each 10-year increment. All controls were free of a history of stroke or transient ischemic attack.

Besta Stroke Study (Milano)

This study includes consecutive Italian patients referred to Besta Institute from 2000 to 2009 with stroke and included in the Besta Cerebrovascular Diseases Registry (CEDIR). Ischemic stroke cases, first ever or recurrent, confirmed on brain imaging, were selected for this study. An experienced stroke neurologist assessed all cases.

deCODE study

The 628 Icelandic cases with lacunar stroke were identified from a registry of individuals diagnosed with ischemic stroke or TIA at Landspitali University Hospital in Reykjavik, the only tertiary referral centre in Iceland, during the years 1993 to 2014. The ischemic stroke or TIA diagnoses were based on standard WHO criteria and imaging

evidence (either CT or MRI), and were clinically confirmed by neurologists. Eligible patients who survived the stroke were invited to participate the genetic study, either by attending a recruitment centre for deCODE's genetic studies, or they were visited at their home by a study nurse. Patients were classified into causative subtypes according to the Trial of Org 10172 in Acute Stroke Treatment (TOAST). The study is based on whole-genome sequencing of 27,633 Icelanders and Illumina SNP chip genotyping of 155,250 Icelanders. Genotypes for the chip-typed individuals are phased using the method of long-rage phasing (Kong et al. Detection of sharing by descent, long-range phasing and haplotype imputation Nat Genet 40, 1068-75, 2008) and genotype probabilities for un-typed variants are imputed into the chip-typed individuals, and their close relatives, using phased genotypes for the 27,633 WGS individuals as reference. Association testing for case–control analysis was performed using logistic regression, adjusting for age, gender and county of origin and assuming a multiplicative model of risk. About 45 million variants, all with imputation info over 0.8, were tested for association. To account for inflation in the test statistics due to cryptic relatedness and stratification within the case and control sample sets, we applied the method of LD score regression (Bulik-Sullivan B. K. et al. LD Score regression distinguishes confounding from polygenicity in genome-wide association studies. Nat. Genet. 47, 291–295, 2015). Control comprised 254,176 individuals recruited through different genetic projects at at deCODE. Individuals with confirmed stroke (identified by cross-matching with hospital lists) were excluded as controls. The study was approved by the Data Protection Commission of Iceland and the National Bioethics Committee of Iceland. All participants gave informed consent.

Dutch Parelsnoer initiative (PSI)

We included Dutch patients with the subtype small vessel disease of ischemic stroke who were enrolled in the Dutch Parelsnoer initiative (PSI) Cerebrovascular Disease (1). This study represents a collaboration of eight university medical centers in the Netherlands in which clinical data, imaging and biomaterials of patients with stroke are prospectively and uniformly collected (1). The present study includes patients enrolled between September 2009 and November 2014. Ischemic stroke was defined as focal neurologic deficits of sudden onset originating from the brain and persisting for more than 24 hours, in the absence of hemorrhage as confirmed by imaging. The subtype small vessel disease was determined according to the Trial of Org 10172 in Acute Stroke Treatment (TOAST) (2). Information on ancestry in patients was obtained by self-report. The Medical Ethics Committee of the different university medical centers in the Netherlands approved the study and all patients provided written informed consent.

1. Nederkoorn PJ, et al. The Dutch String-of-Pearls Stroke Study: Protocol of a large prospective multicenter genetic cohort study. Int. J. Stroke. 2015;10:120–122.
2. Adams H, et al. (TOAST) Classification of Subtype of Acute Ischemic Stroke. Stroke. 1993;23:35–41.

Barcelona

Stroke patients were recruited in Vall d'Hebron Hospital and Sant Pau Hospital (Barcelona, Spain). The patients were recruited if they had a stroke diagnosis performed by an experienced neurologist at each center and confirmed by neuroimaging, were older than 18 years of age. Etiologic subgroups were classified following TOAST criteria. Controls were subjects without a history of ischemic stroke, older than 18, who declared they were free of neurovascular diseases by direct interview before recruitment. The

control cohort was collected in primary care centers from Barcelona city and in hospitals throughout Spanish territory as a part of the GRECOS project (Fernandez-Cadenas et al Stroke 2017). All participants were genotyped with the Illumina® Human Core Exome chip.

SAHLSIS

SAHLSIS is a case-control study of ischemic stroke based in Gothenburg, Sweden. Adult subjects who presented with first-ever or recurrent acute ischemic stroke before 70 years of age were recruited consecutively at stroke units in western Sweden from 1998 to 2012. All participants were of European origin. Patients were not excluded based on stroke severity or whether they were enrolled in a treatment trial. All participants underwent ECG and neuroimaging at the acute stage (all by CT and 58% also by MRI). Additional diagnostic work-up was performed when clinically indicated. Inclusion criteria was ischemic stroke which was defined as an episode of focal neurological deficits with acute onset and lasting > 24 hours or until death, with no apparent non-vascular cause, and no signs of primary hemorrhage on brain imaging. Subjects were excluded if they had a diagnosis of cancer at advanced stage, infectious hepatitis or human immunodeficiency virus. Ischemic stroke was assigned according to modified TOAST criteria.

Helsinki Ischemic Stroke Genetics Study

Helsinki Ischemic Stroke Genetics Study was designed for investigating genetic factors underlying ischemic stroke in the Finnish population and in the long-term to be incorporated to multicenter multinational similar datasets. Ischemic stroke cases were recruited from 2012 to 2017 from the Helsinki University Hospital, Department of Neurology which is the only neurological emergency unit for a population of 1.7 million inhabitants. 1848 patients with positive neuroimaging findings for a new-onset brain infarction were included. All cases are of white, Caucasian origin. Stroke subtyping was performed according to the Trial of Org 10172 in Acute Stroke Treatment (TOAST) classification. 3560 control samples were obtained from the national FINRISK study 1992, 1997, 2002, 2007 and 2012 cohorts (Salomaa 2016 PMID: 27242092). Only participants residing in the same geographic area (Greater Helsinki region) and without history of ischemic or hemorrhagic stroke based on the National Hospital Discharge Register and the National Causes-of-Death Register (ICD-10 codes I61, I63.0-I63.5, I63.7-I63.9, I64 or respective in the earlier ICD versions) were included as controls. Cases and controls have provided a blood sample for the genetic analyses and have been genotyped on the Illumina HumanCoreExome or Illumina Global Screening array. The Helsinki Ischemic Stroke Genetics study and FINRISK study have been approved by the Ethics Committee of Medicine, Helsinki University Hospital and the Coordinating Ethics Committee of Helsinki University Hospital, respectively. All the participants have signed an informed consent.

MyCode Community Health Initiative (MyCode)

Geisinger MyCode Community Health Initiative (MyCode®) is a system-wide biorepository with more than >260,000 participants enrolled to date. Participants are consented to use their deidentified genetic and electronic health record (EHR) data for research purposes. Genotyping was performed in batches on the Illumina Infinium OmniExpress

Exome array and GSA-24v1-0 array and were imputed to HRC.r1-1 EUR reference genome (GRCh37 build) separately using the Michigan Imputation Server. Centralized data included the overlapped variants from the imputation and variants should have an info score ≥0.7.

Carey DJ, Fetterolf SN, Davis FD, et al. The Geisinger MyCode community health initiative: an electronic health record-linked biobank for precision medicine research. Genet Med. 2016;18(9):906-913.

Dewey FE, Murray MF, Overton JD, et al. Distribution and clinical impact of functional variants in 50,726 whole-exome sequences from the DiscovEHRstudy. Science. 2016;354(6319).

NINDS-SIGN consortium

Cases

BASEede datos de ICtus del hospital del MAR (BASICMAR)

BASICMAR is an ongoing prospective study of all acute strokes assessed since 2005 at the IMIM-Hospital Universitari del Mar (Barcelona, Spain). It includes both first-ever and recurrent strokes. There were no exclusion criteria regarding age or race-ethnicity of the individuals. All patients had an electrocardiogram (ECG), a blood analysis, and neuroimaging at the acute stage. Additional diagnostic procedures were s performed when clinically indicated. A follow-up of three months after stroke was completed for all survivors. Ischemic stroke etiologic subtypes were classified according to TOAST criteria.For this study, only individuals of European origin with ischemic stroke were selected from BASICMAR, with eligible events defined as a clinical syndrome of any duration associated with a radiographically proven acute infarct, without radiographic evidence of a demyelinating or neoplastic disease or other structural disease including primary intracerebral hemorrhage.

GCNKSS

The GCNKSS is a population-based epidemiologic study of stroke in blacks and whites that is designed to measure temporal trends and racial differences in incidence of stroke. The catchment area includes two southwestern Ohio, U.S.A., counties (Hamilton, which includes the city of Cincinnati, and Clermont to the east) and three Northern Kentucky, U.S.A., counties (Boone, Kenton, and Campbell)to the south of Cincinnati across the Ohio River. As part of the GCNKSS, for calendar years 1999 and 2005, prospective cohorts of first-ever and recurrent ischemic stroke cases were assembled using “hot pursuit” methodology at all local hospitals in the region (18 in 1999, and 17 in 2005), except for one hospital that is solely devoted to treating pediatric cases. Participants remained eligible if they were in a treatment trial, but participation in a treatment trial was not required for enrollment. Subjects with all degrees of severity of stroke were eligible, and no particular racial group was intentionally oversampled (about 80% were white participants and 20% black). Study research nurses prospectively screened inpatient admission and emergency department logs to identify acute ischemic stroke

patients. When a case was identified and the treating physician had given permission to approach the patient, a study nurse asked the subject or a proxy (the most closely related competent individual, preferably a person living with the subject prior to the stroke) to consent to participate in the cohort. After consent was granted, a study nurse performed an extensive interview, and a blood sample was obtained for genetic analysis. In addition, a study nurse abstracted information about the individual, the subject's medical history, the stroke event, and imaging studies from the hospital chart. A study physician reviewed every abstract, along with the imaging studies, to verify that an acute stroke had occurred, and to classify the event according to TOAST and CCS criteria.

Graz

Between 1994 and 2003, subjects with first-ever and recurrent ischemic strokes admitted to the stroke unit of the Department of Neurology, Medical University of Graz (Graz, Austria) were included. All race-ethnic groups were eligible and there was no intentional oversampling. All age groups were allowed, though only subjects above the age of 18 were admitted to our department. Ischemic stroke was defined as an episode of focal neurological deficits with acute onset and lasting > 24 hours. There were no selection criteria based on stroke severity. Those individuals in treatment trials were excluded. 685 subjects were eligible to participate in this study (278 women, 407 men). All cases were Caucasian. Mean age was 68.9 ± 13.8 years with an age range from 19 –101 years. In addition to a standardized protocol including a laboratory examination and carotid ultrasound or magnetic resonance angiography and ECG, 304 subjects underwent neuroimaging by CT and 381 by MRI. More extensive cardiac examination, including transesophageal echocardiography or transthoracic echocardiography and Holter, was performed in subjects with suspected cardiac embolism. Stroke subtypes were assessed according to modified TOAST criteria and were conducted by trained stroke neurologists.

Krakow

All consecutive subjects with ischemic stroke (fulfilling WHO criteria12(3)) who were admitted to the Stroke Unit at the Jagiellonian University (Krakow, Poland) and who provided informed consent were included in the study. The Stroke Unit serves as a stroke emergency center for one district of Krakow, Poland (200,000 inhabitants) and as a referral center for South East Poland (up to 15% of all admissions). For this on-going, prospective single-center, hospital-based study participants with first ever or recurrent strokes were recruited from January 22, 2002 to September 9, 2010. The local research ethics committee approved the study. Participants in treatment trials were excluded. All subjects were of European origin. Stroke severity was not a criterion for inclusion or exclusion. All cases had performed clinically relevant diagnostic workup, including brain imaging with CT (100%) and/or MRI (up to 20%) as well as ancillary diagnostic investigations including duplex ultrasonography of the carotid and vertebral arteries (approximately 90%), and transthoracic echocardiography (approximately 70%). Magnetic resonance angiography (MRA), computed tomographic angiography (CTA), and ambulatory ECG monitoring, transesophageal echocardiography and blood tests for hypercoagulability were performed. Stroke cases were classified into etiologic subtypes according to TOAST. All cases were phenotyped independently by two experienced stroke neurologists with review of original imaging. Cases were subsequently classified additionally using the CCS system.

Leuven Stroke Genetics Study (LSGS)

Cases of European descent with cerebral ischemia, defined as a clinical stroke with imaging confirmation or a TIA with a new ischemic lesion on diffusion-weighted imaging, who were admitted to the Stroke Unit of the University Hospitals (Leuven, Belgium) were enrolled in the LSGS between 2005 and 2009. All participants from the LSGS study underwent brain imaging (MRI in 91% of patients, CT in the remainder) and a standardized protocol including lab examination, carotid ultrasound or CTA and cardiac examination (echocardiography and ambulatory ECG monitoring) in all patients. Based on clinical presentation and results from the diagnostic work-up, cases were classified into ischemic stroke etiologic subtypes according to modified TOAST criteria by a single reviewer. The reviewer had access to all information and imaging. Large-vessel disease was defined as either occlusive or significant stenosis (corresponding to > 50% luminal diameter reduction according to North American Symptomatic Carotid Endarterectomy Trial (NASCET) criteria13of a clinically relevant pre-cerebral or cerebral artery, presumably due to atherosclerosis. Carotid ultrasound was used as a screening tool, and in principle, additional imaging with CTA or MRA was performed when a high-grade stenosis was identified. In case CTA was used as the primary imaging modality, stenosis was confirmed by carotid ultrasound. In case of posterior circulation infarcts on imaging, CTA or MRA was used as the primary imaging modality to determine the degree of stenosis. Probable causes of cardiac embolism were excluded. The presence of a patent foramen ovale was not considered a cardiac source in this context. Intracranial atherosclerosis was considered present only if repeat imaging after at least one week revealed a similar degree of stenosis or persistent occlusion. If not, the findings were interpreted as an embolism from a proximal source. Small-vessel disease was defined as a symptomatic infarct of < 20 mm on DWI in areas supplied by single, small penetrating branches from middle cerebral artery, posterior cerebral artery or basilar artery in the absence of both a cardioembolic source and significant stenosis/occlusion due to atherosclerosis of an appropriate major brain artery. Cardioembolic stroke was defined as ischemic stroke in the presence of atrial fibrillation, sick sinus syndrome, myocardial infarction in the past four weeks, cardiac thrombus, infective endocarditis, atrial myxoma, prosthetic mitral or aortic valve, valvular vegetations, left ventricular akinetic segment, dilated cardiomyopathy, or patent foramen ovale or atrial septal aneurysm. Significant stenosis/occlusion due to atherosclerosis of an appropriate pre-cerebral or cerebral artery should be excluded. Other determined cause of stroke included those with arterial dissection, vasculitis, hematologic disorders, monogenic syndromes and complications of cardiovascular procedures. Dissection was diagnosed by typical findings on contrast-enhanced MRA and T1-fat suppressed MRI. Cryptogenic stroke was defined when no cause was identified despite an extensive evaluation. Strokes associated with significant aortic arch atheroma with plaques of ≥ 4 mm were also considered cryptogenic strokes. In addition to this primary classification, cases were reclassified using CCS.

Lund Stroke Register (LSR)

The LSR is an ongoing study including consecutive subjects with first-ever stroke since March 1, 2001 from the local uptake area of Skåne University Hospital, Lund (Sweden). Stroke was defined using the WHO criteria. Subjects aged 18 years or older with stroke caused by cerebral infarct, intracerebral hemorrhage or subarachnoid hemorrhage are included. Cases are included regardless of stroke severity, race-ethnic group belonging, or participation in any treatment trial. Those with iatrogenic or

traumatic stroke are excluded. In the discovery phase of the SiGN study, subjects from LSR with first-ever ischemic stroke between March 1, 2001 and February 28, 2010 were included if they or their next of kin provided informed consent. Age over 90 years was set to 90 years to maintain anonymity. Every participant underwent CT, MRI, or autopsy of the brain; and ECG. Echocardiography, ultrasound, CTA or MRA of cerebral arteries was performed when judged clinically relevant. The subtype of ischemic stroke was determined using CCS. For the secondary phase of SiGN, LSR individuals not included in the SiGN discovery phase participated after genotyping in the South Swedish genome-wide association study as follows: first ever ischemic stroke cases recruited in 2006 and 2010 to 2012, and age-and sex-matched LSR control subjects without stroke recruited in 2001 to 2002 and 2006 to 2007 from the same geographical area with use of the official Swedish population register.

Middlesex County Ischemic Stroke Study (MCISS)

The MCISS was initiated as a prospective hospital based stroke registry at the New Jersey Neuroscience Institute (Edison, NJ, U.S.A.). All cases over age 18 years were included, and no specific ethnic/racial group was targeted or excluded. From 2000 to 2009, 1,139 subjects with ischemic strokes were enrolled in this registry. There was no selection criterion based upon stroke severity, and both first-ever and recurrent strokes were included. Cases that were participants in treatment trials were not excluded. The major race/ethnic groups are Whites (67.2%), African Americans (14.3%), Asian Indians (8.2%), Hispanic (5.5%) and others (4.8%, Chinese and other Asians). All subjects withclinical suspicion of a stroke were admitted through the emergency room to a dedicated stroke unit supervised by a vascular neurologist. After a history and neurological examination, a standardized series of investigations were performed: complete blood count and differential, comprehensive metabolic panel, electrolytes, blood urea nitrogen, creatinine, lipid panel (total cholesterol, low-density lipoprotein, high-density lipoprotein, triglyceride levels, homocysteine levels, a cerebral MRI/MRA (if the MRIcould not be performed, a head CT scan was done), carotid duplex ultrasound, ECG and an echocardiogram. The diagnosis of cerebral infarct was confirmed by the imaging studies. The epidemiological and clinical data on these participants was collected prospectively. Two independent investigators (one of which was a board-certified neurologist with expertise in vascular neurology) reviewed the data, and all strokes were classified into etiological subtypes using TOAST criteria. In addition, the Oxfordshire stroke classification14was applied, and the vascular distribution of stroke was tabulated. All procedures, including the generation of the databases and recruitment of the stroke subjects, were conducted following Institutional Review Board policies and procedures at the New Jersey Neuroscience Institute/JFK Hospital.

Miami Stroke Registry and Biorepository (MIAMISR)

The MIAMISR at the University of Miami/Jackson Memorial Hospital (Miami, FL, U.S.A.) is an ongoing prospective hospital registry of consecutive patients subjects with prevalent stroke (ischemic and hemorrhagic) and TIA with available neuroimaging (CT or MRI) who provide informed consent. There are no specific exclusion criteria with the respect to age, stroke severity, disability or participation in treatment trials. It was established in November of 2008 in order to investigate stroke type, ischemic stroke subtypes, stroke genetics and stroke outcomes in diverse ethnic population of Miami. The stroke population is predominately Hispanic (63%), with Cuba (32%), Nicaragua (4.8%), Colombia (4.8%), and Puerto Rico (4.1%) contributing the most subjects. Jackson Memorial Hospital is a 1,550-bed county hospital affiliated with the University of

Miami with approximately 900 stroke and TIA admissions per year. Demographic and clinical data along with blood samples for genetic and other research have been collected prospectively during the hospitalizations. Follow-up information was obtained at 90 days by telephone interview or in person. Trained research staff obtained written informed consent from the stroke patients or the health care proxy when available for participation in MIAMISR.

Nurses' Healthy Study (NHS)

The NHS cohort consists of 121,700 female registered nurses aged 30 –55 years who were residing in 11 U.S. states and who were enrolled in 1976 through responding to a mailed questionnaire on their medical history and lifestyle practices. They have been followed with biennial mailed questionnaires collecting information on disease risk factors and health status. From 1989 –1990, blood samples were collected from 32,826 participants. Among these participants, we prospectively identified incident strokes and confirmed ischemic stroke cases by medical record review. Clinical symptoms consistent with stroke and exclusion of alternate etiologies were required for classification of stroke. Virtually all cases had imaging, but confirmation on CT or MRI was not required. No participants were excluded based on race/ethnicity. Neither stroke severity nor enrollment in a treatment trial was part of the eligibility criteria.

Northern Manhattan Study (NOMAS)

NOMAS is an ongoing population-based study designed to determine stroke incidence, risk factors and outcome in an urban multiethnic population.16NOMAS started in 1993 as a case-control study of index ischemic stroke cases admitted to the Columbia University Presbyterian Medical Center (New York, NY, U.S.A.) and affiliated hospitals and matching community controls (Northern Manhattan Stroke Study, NOMASS) and continued as a prospective stroke incidence study by following up controls in 1997 (NOMAS). Demographic and clinical data were collected prospectively during the hospitalizations and annually by phone or in person. Genetic samples were derived from two sources: (a) the population-based case-control study conducted from 1993-98 (NOMASS) and (b) the ongoing prospective cohort study (NOMAS). First-ever ischemic stroke cases were identified for the case-control study by screening of patient admissions, discharge codes, and referrals for neuroimaging at 15 acute care hospitals in the defined study area and multiple approaches to monitor for non-hospitalized cases. Incident ischemic stroke cases were identified from the prospective cohort study through follow-up visits and scheduled telephone contacts. Ischemic stroke cases from both sources were followed at 6 months by telephone and then annually afterwards in order to assess functional status and other outcomes. The administrative coordinating center of NOMAS moved from New York to Miami in 2007. The Institutional Review Boards of both institutions, Columbia University and the University of Miami (Miami, FL, U.S.A.), approved the study.

Reasons for Geographic and Racial Differences in Stroke (REGARDS)

The REGARDS study is a U.S. national, population-based, longitudinal cohort of 30,239 African American and white adults aged ≥ 45 years, recruited January 2003 to October 2007 with ongoing follow-up. Suspected stroke is queried every six months and triggered by participant self-report of stroke, stroke symptom(s), hospitalization, or

proxy report of death. Stroke severity and participation in a treatment trial did not limit inclusion in this study. Medical records for these reported events are retrieved and reviewed by at least two members of a committee of stroke experts with disagreements resolved by a third adjudicator. A symptom-based approach, independent of neuroimaging outcome, is used to confirm events using the WHO definition of stroke. An infarct did not need to be seen on brain imaging to be included in this study. Ischemic stroke subtype classification is conducted using the TOAST system.

Secondary Prevention of Small Subcortical Strokes (SPS3)

The SPS3 trial (NCT00059306) is a randomized, multicenter, Phase 3 trial of antiplatelet therapy and antihypertensive therapy. Participants are randomized to aspirin alone or the combination of aspirin and clopidogrel. Participants are also randomized to two groups of blood pressure control: either to a target systolic blood pressure of 130 –149 mm Hg or < 130 mm Hg. Principal eligibility criteria include man or woman at least 30 years of age with clinical evidence of small subcortical stroke and brain MRI evidence of small subcortical infarct. Subjects were required to not have evidence of ipsilateral symptomatic cervical carotid stenosis or high-risk cardioembolic sources for embolism. Further details of eligibility criteria have been published. Primary outcomes include ischemic and hemorrhagic stroke. DNA samples were collected from 38% (1,139/3,020) of participants in the trial. These samples were obtained from 46% (37/81) participating centers across the U.S., Canada, Spain, Mexico, Chile, Ecuador and Peru. No additional eligibility criteria were necessary beyond informed consent for participating in the DNA sub-study. A total of 0.9% (10/1,139) of DNA donors gave sample at time of randomization, with the remainder donating at a later time point in follow-up.

Washington University St. Louis (WUSTL) Study

The WUSTL patient collection included ischemic stroke cases admitted to Barnes-Jewish Hospital/Washington University Medical Center (St. Louis, MO, U.S.A.) for genetic studies starting from August 1, 2008. Participants were identified for the genetic studies by screening admissions at our tertiary care hospital (both in the Emergency Department and on the Inpatient Stroke Service) without regard to age, race or ethnicity, including both first-ever and recurrent strokes. Subjects were retained in the study if their discharge diagnosis was ischemic stroke (without requirement for the stroke to be visualized on CT or MRI). Demographic and clinical data were collected prospectively during the hospitalization and at 90 days, by phone or in person. Genetic samples were derived from subjects enrolled in 3 different studies: (a) Acute tPA pharmacogenomics study (Ischemic stroke cases who received tPA and were admitted to BJH/Washington University; serial NIHSS scores, and data on hemorrhagic transformation was collected) (b) Recovery Genomics after Ischemic Stroke Study (ReGenesIS, Ischemic stroke cases with NIHSS > 3 points without underlying chronic neurological disease, and expected survival up to 3 months after stroke), and (c) the Cognitive Recovery and Rehabilitation Group (CRRG) Registry (all ischemic stroke cases admitted to BJH/Washington University who consent to entering their clinical data into a stroke registry, and the collection of blood for genetic analysis). Cases that were part of a treatment trial were excluded from the tissue plasminogen activator pharmacogenomics and ReGenesIS study, but not the CRRG registry.

Controls

Attention-deficit Hyperactivity Disorder (ADHD)

The Vall d'Hebron Research Institute (VHIR) cohort included 435 blood donors of Caucasian origin recruited from 2004 to 2008 at the Hospital Universitari Vall d'Hebron (Barcelona, Spain) to identify loci conferring susceptibility to Attention-Deficit Hyperactivity Disorder. Seventy-six percent of participants were male (N = 330) and the average age at assessment was 43.8 years (s.d. = 14.3). Genome-wide genotyping was performed with the Illumina HumanOmni1-Quad BeadChip platform. The study was approved by the ethics committee of the institution and informed consent was obtained from all participants in accordance with the Declaration of Helsinki.

Health ABC (HABC)

The Health Aging and Body Composition (Health ABC) Study is a National Institute on Aging (NIA)-sponsored cohort study of the factors that contribute to incident disability and the decline in function of healthier older persons, with a particular emphasis on changes in body composition in old age. Between April 15, 1997 and June 5, 1998, the Health ABC study recruited 3,075 70 –79 year old community-dwelling adults (41% African American), who initially had no indications of disability related to mobility and activities of daily living. The key components of Health ABC include a baseline exam, annual follow-up clinical exams, and phone contacts every six months to identify major health events and document functional status between clinic visits. The core yearly examination for Health ABC includes measurement of body composition by dual energy x-ray absorptiometry (DXA), walking ability, strength, an interview that includes self-report of limitations and weight, and a medication survey. At baseline, visceral adiposity was measured by computerized tomography (CT). Provision has been made for banking of blood specimens and extracted DNA (HealthABC repository). The overall goal of this project is to identify genetic determinants of visceral adiposity.

Hispanic Community Health Study/Study of Latinos (HCHS/SOL)

The Hispanic Community Health Study/Study of Latinos (HCHS/SOL) was initiated in 2006 to investigate the prevalence and risk factors affecting several health conditions, including heart, lung and blood disorders, kidney and liver function, diabetes, cognitive function, dental conditions and hearing disorders.24,25 Participants aged 18 –74 self-identified as Hispanic or Latino, with substantial representation of Mexican, Puerto Rican, Dominican, Cuban, Central and South American groups. They were recruited from four field centers in the United States: San Diego, CA; the Bronx, NY; Chicago, IL; and Miami, FL. 12,803 study participants consented to genetic studies and will be included in the HCHS/SOL dbGaP posting. Genotyping of the HCHS/SOL participants was performed at Illumina Microarray Services using the SOL HCHS Custom 15041502 array (annotation version “B3”, genome build 37), which includes 2,575,443 variants (of which 2,427,090 are in common with the Illumina HumanOmni2.5 and 148,353 are custom content).Health and Retirement Study (HRS)The University of Michigan Health and Retirement Study (HRS) is a longitudinal panel study that surveys a representative sample of more than 20,000 Americans over the age of 50 every two years. Supported by the National Institute on Aging (NIA U01AG009740) and the Social Security Administration, the HRS explores the changes in labor force participation and the health transitions that individuals undergo toward the end of their work lives and in

the years that follow. Since its launch in 1992, the study has collected information about income, work, assets, pension plans, health insurance, disability, physical health and functioning, cognitive functioning, and health care expenditures. HRS is intended to be a nationally representative sample with 2:1 oversampling of minority groups including African-American and Hispanic/Latino populations. In Phases I –II, 12,507 study participants were included in the dbGaP posting. Genotyping of the HRS Phase I –II participants was performed at CIDR using the Illumina HumanOmni2.5-4v1 array (annotation version "D", genome build 37) and released a total of 2,443,179 variants.

INfancia y Medio Ambiente (INMA)

The INfancia y Medio Ambiente (Environment and Childhood) project is a research project comprising a Spanish population-based birth cohort created to study the role of the environmental pollutants during pregnancy and first stages of life and their effects on childhood growth and development. The cohort was established between 2003 and 2008 from mothers enrolled in four regions within Spain and included their infants.

MONICA/KORA Augsburg Study

For the German MUNICH discovery samples, independent control groups were selected from Caucasians of German origin participating into the population KORAgen study. This survey represents a sex-and age stratified random sample of all German residents of the Augsburg area and consists of individuals 25 –74 years of age, with about 300 subjects for each 10-year increment. All controls were free of a history of stroke or transient ischemic attack. KORA samples were genotyped on the Illumina Human 550k platform. QC was identical for all WTCCC cohorts.

Krakow

The control group included unrelated subjects taken from the population of southern Poland. Control subjects had no apparent neurological disease based on the findings in a structured questionnaire and a neurological examination. Local research ethics committees approved the study and informed consent was obtained from all participants.

Leuven Stroke Genetics Study (LSGS)

Control individuals were recruited in the same population amongst healthy individuals, spouses of patients suffering from neurological diseases (amyotrophic lateral sclerosis, ischemic stroke or multiple sclerosis), and from the Leuven University Gerontology Database as previously described.

Osteoarthritis Initiative (OAI)

The OAI is a publicly and privately funded prospective longitudinal cohort with a primary objective of identifying risk factors for incidence and progression of tibiofemoral knee OA. The OAI utilized a focused population-based recruitment to enrol 4,674 men and women between the ages of 45 –79 years who either had radiographic

symptomatic knee OA or who were without radiographic symptomatic OA in both knees but were considered high risk for OA because they had two or more known risk factors for knee OA. Subjects were recruited into the baseline phase of the OAI at multiple sites throughout the US between 2004 and 2006. All subjects were invited back for follow-up examinations to assess incidence or progression of OA annually, for up to 5 years. Phenotype data from the baseline and follow-up examinations are available for public access from the Osteoarthritis Initiative (OAI) database. The Genetic Components of Knee Osteoarthritis (GeCKO) Study was initiated in 2009 as a genetic ancillary study to perform a genome-wide association study to identify genetic variants associated with radiographic osteoarthritis. This study included 4,482 individuals participating in the parent OAI study genotyped on the Illumina HumanOmni2.5M.

Supplementary Figures 1-2. QQ-plots of Observed against Expected -log10(p-values) for European (Left) and Transethnic (Right) Genome-wide Analyses

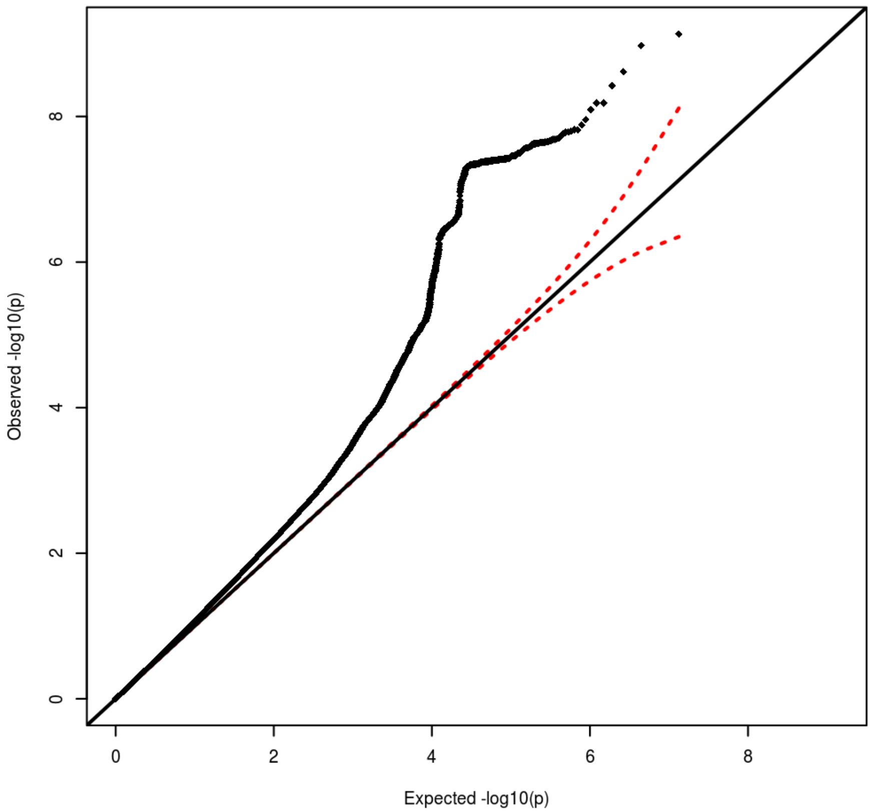

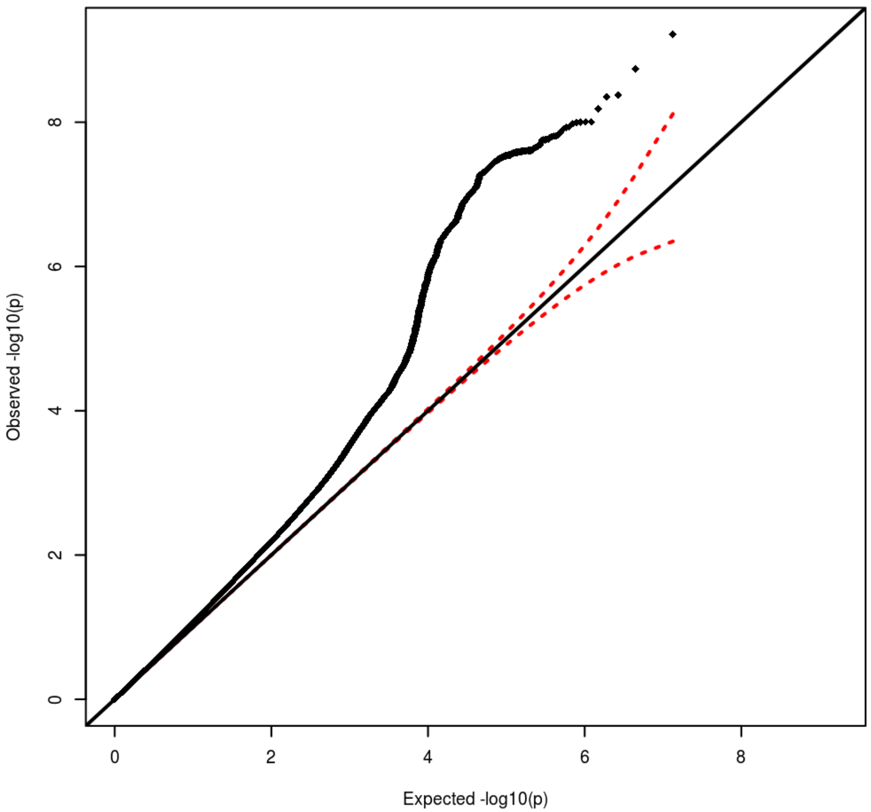

Supplementary Figure 3. SPI1-SLC39A13-PSMC3-RAPSN Region in European (left) and Transethnic (right) analysis

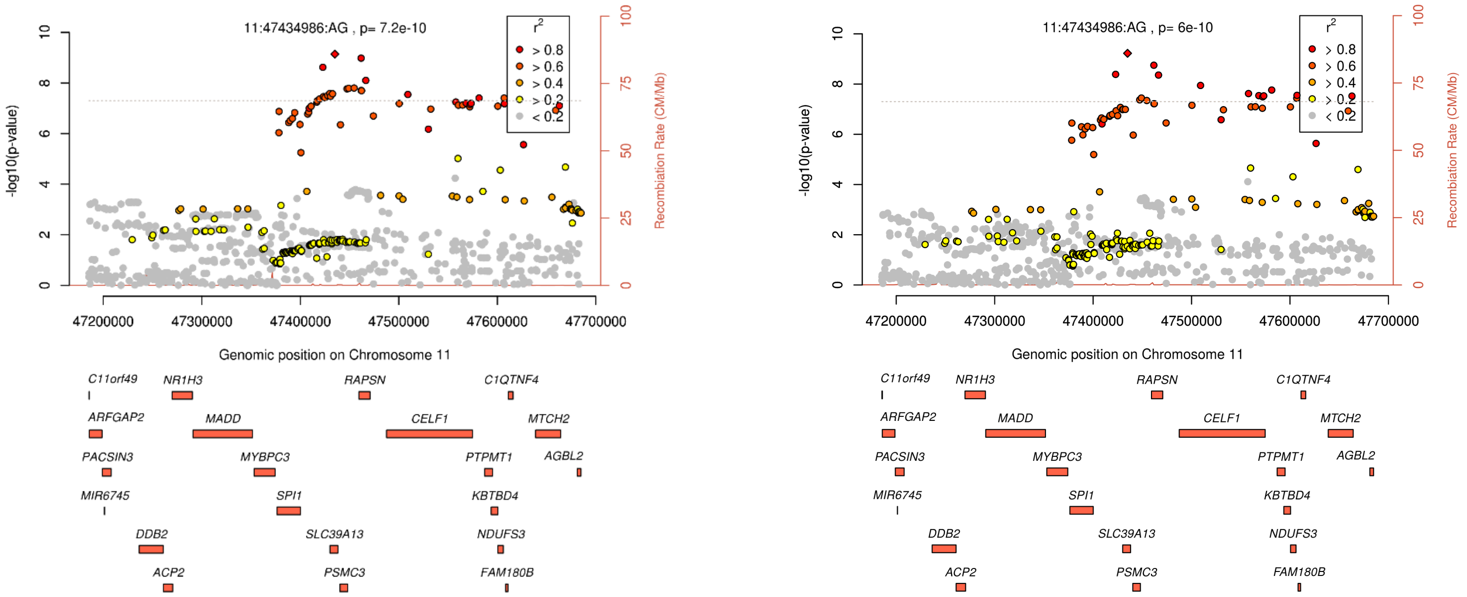

Supplementary Figure 4. ICA1L-WDR12-CARF-NBEAL1 Region in European (left) and Transethnic (right) analysis

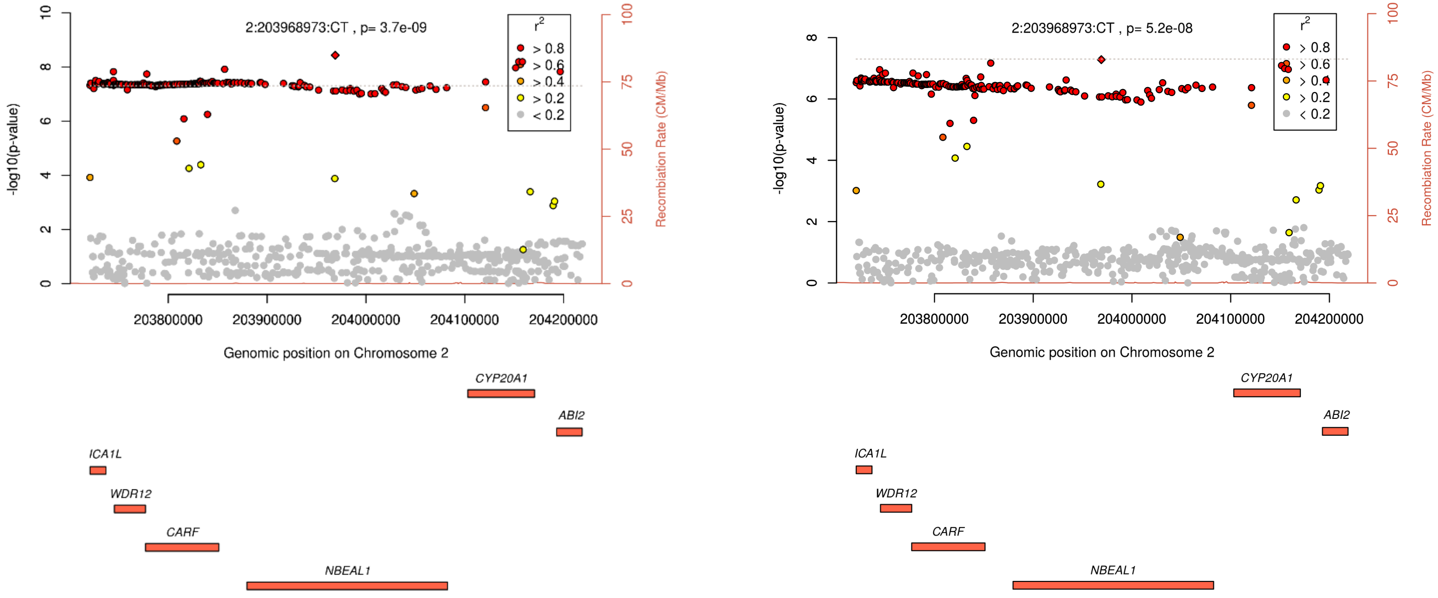

Supplementary Figure 5. ZCCHC14 Region in European (left) and Transethnic (right) analysis

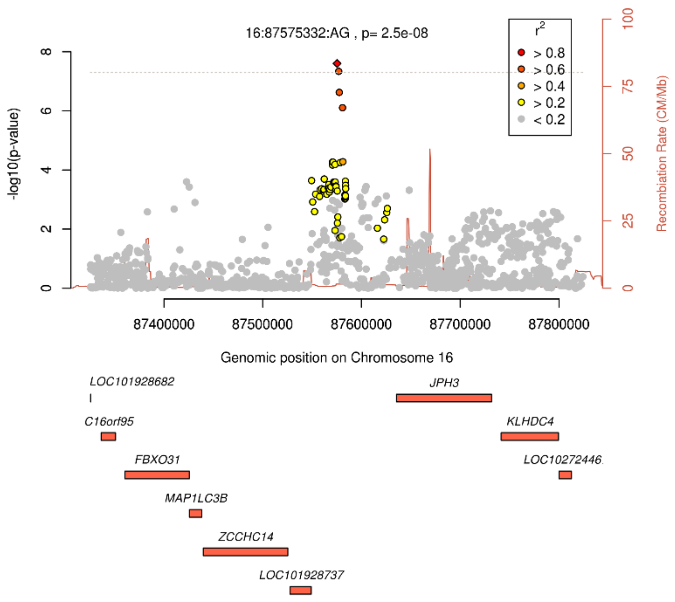

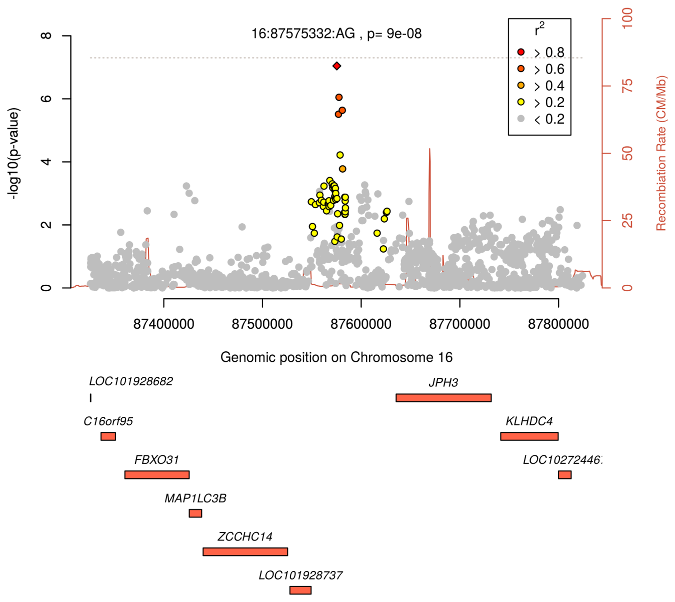

Supplementary Figure 6. ULK4 Region in European (left) and Transethnic (right) analysis

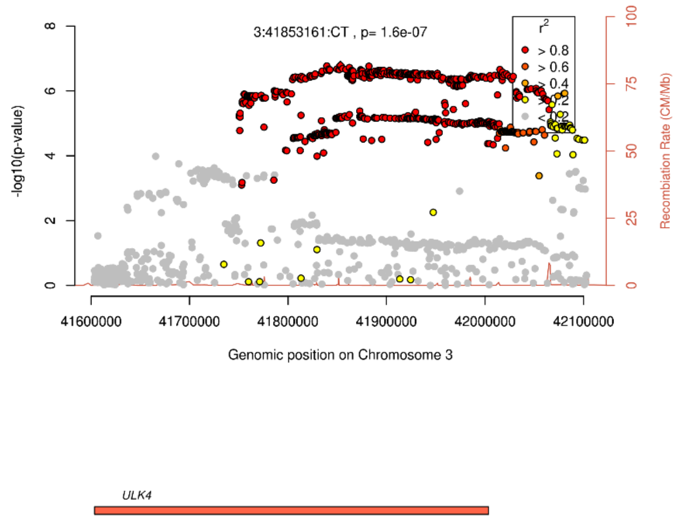

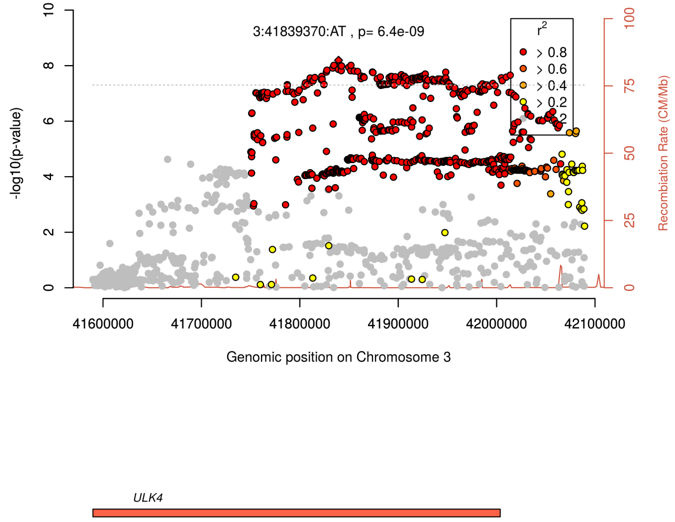

Supplementary Figure 7. ZBTB14-EPB41L3 Region in European (left) and Transethnic (right) analysis

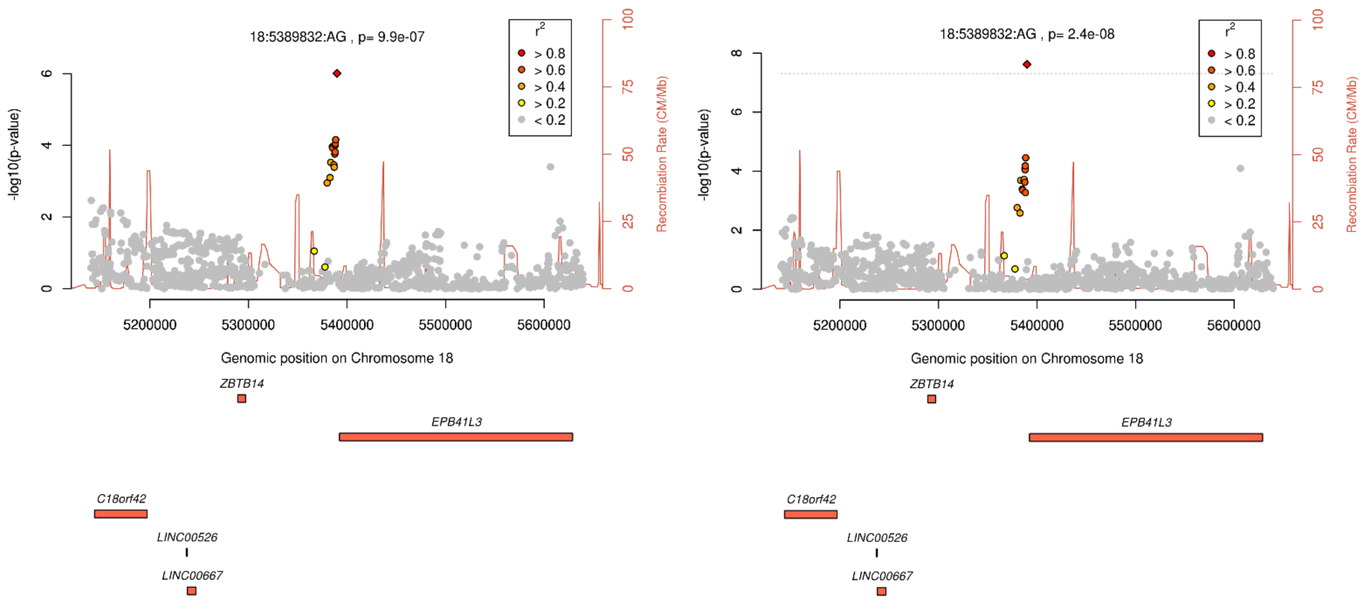

Supplementary Figures 8-11. Regions achieving genome-wide significance in multi-trait analysis

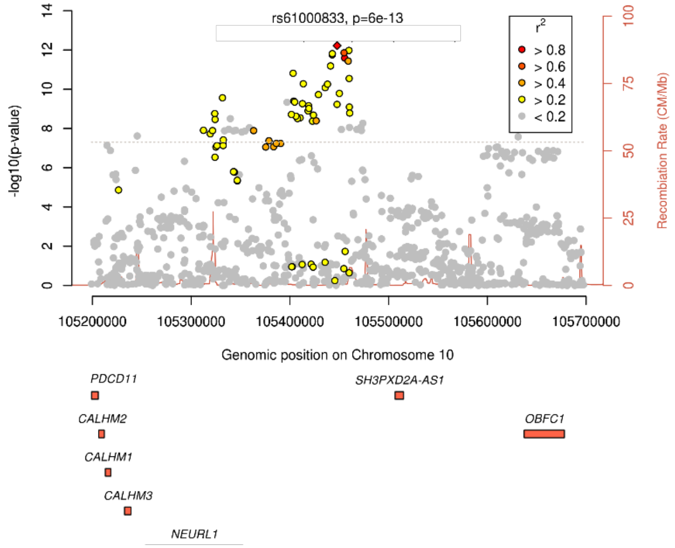

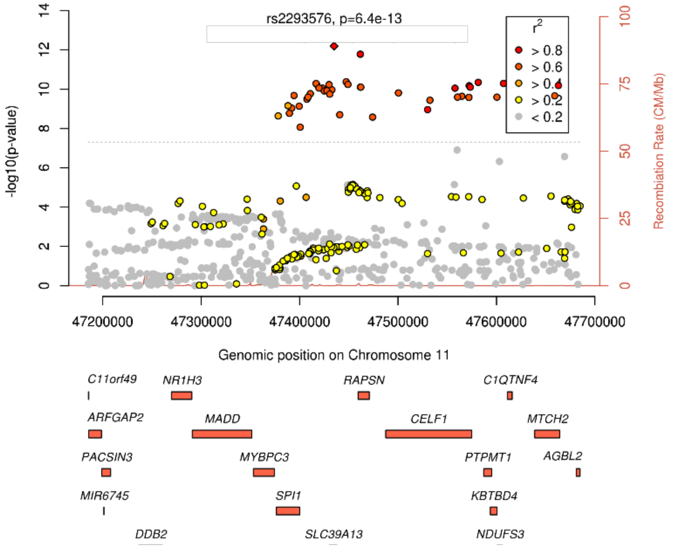

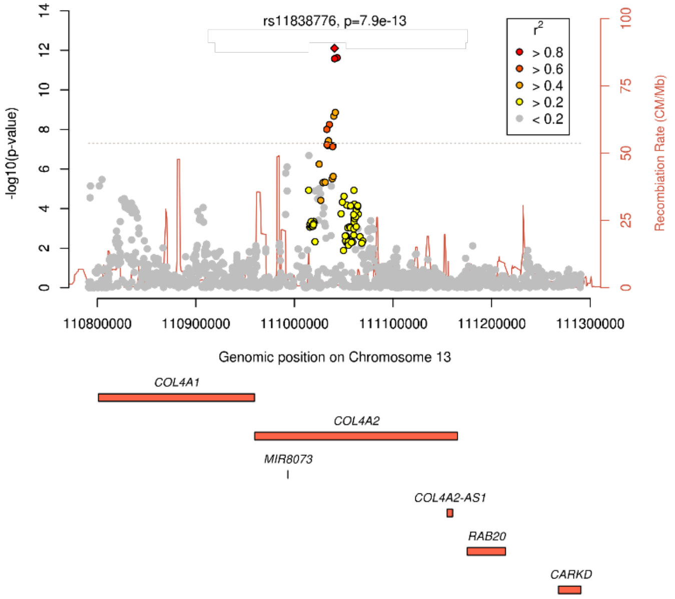

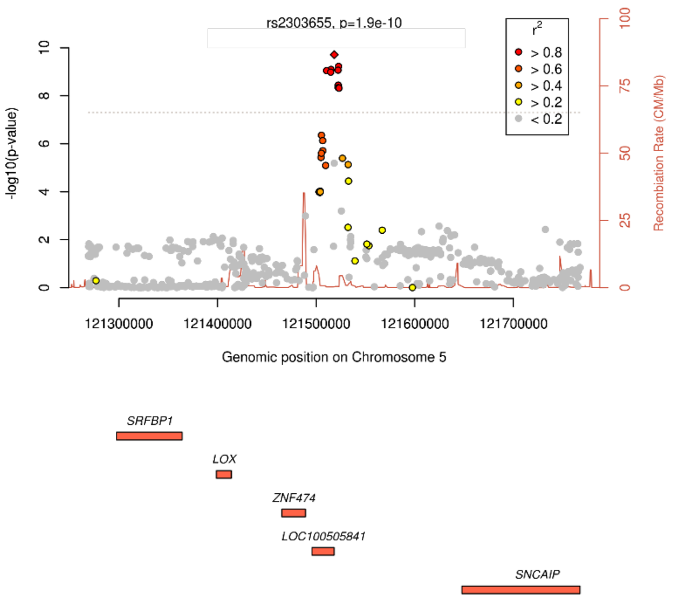

Supplementary Figures 12-15. Regions achieving genome-wide significance in multi-trait analysis

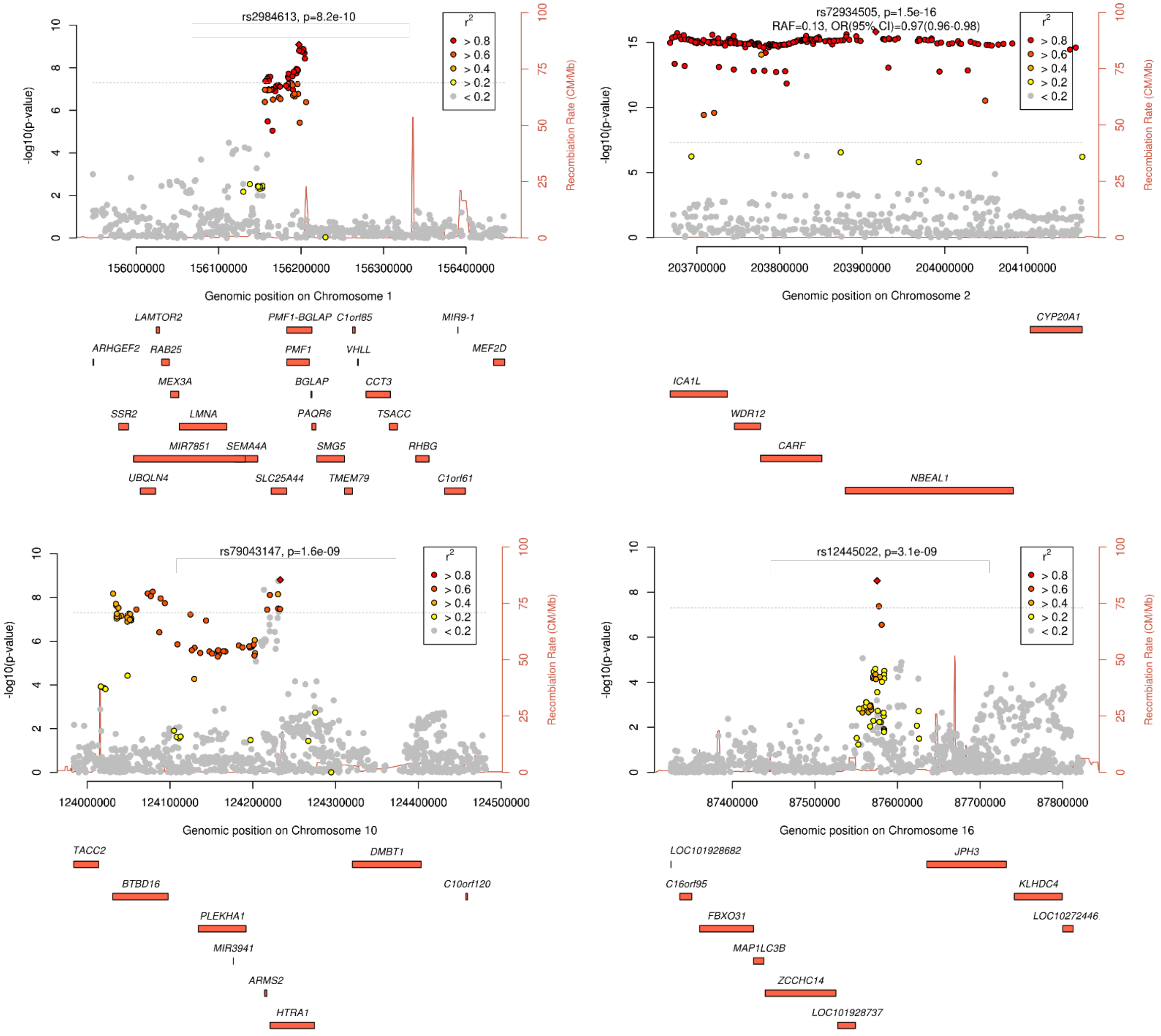

Supplementary Figures 16-17. Regions achieving genome-wide significance in multi-trait analysis

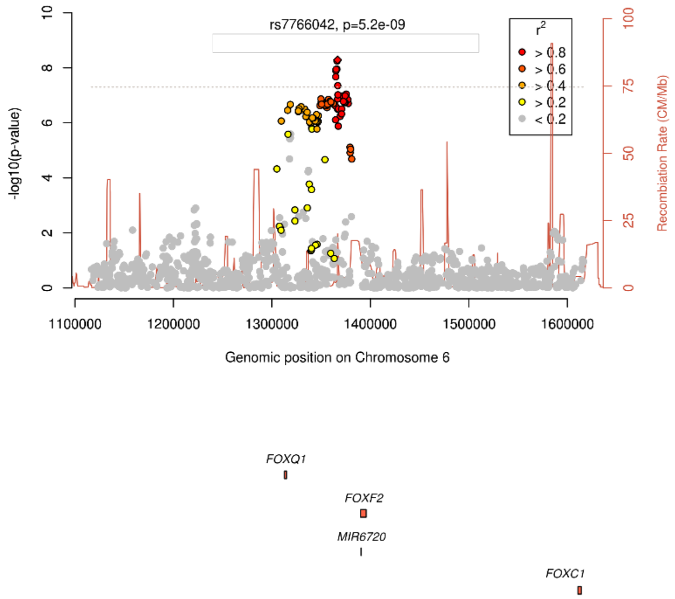

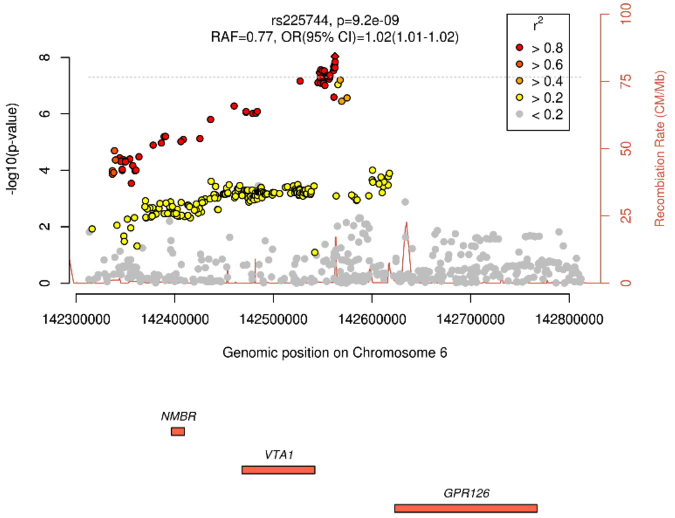

Supplementary Figure 18 – Forest Plot for Association of rs72934535 at ICA1L-WDR12-CARF-NBEAL1 locus

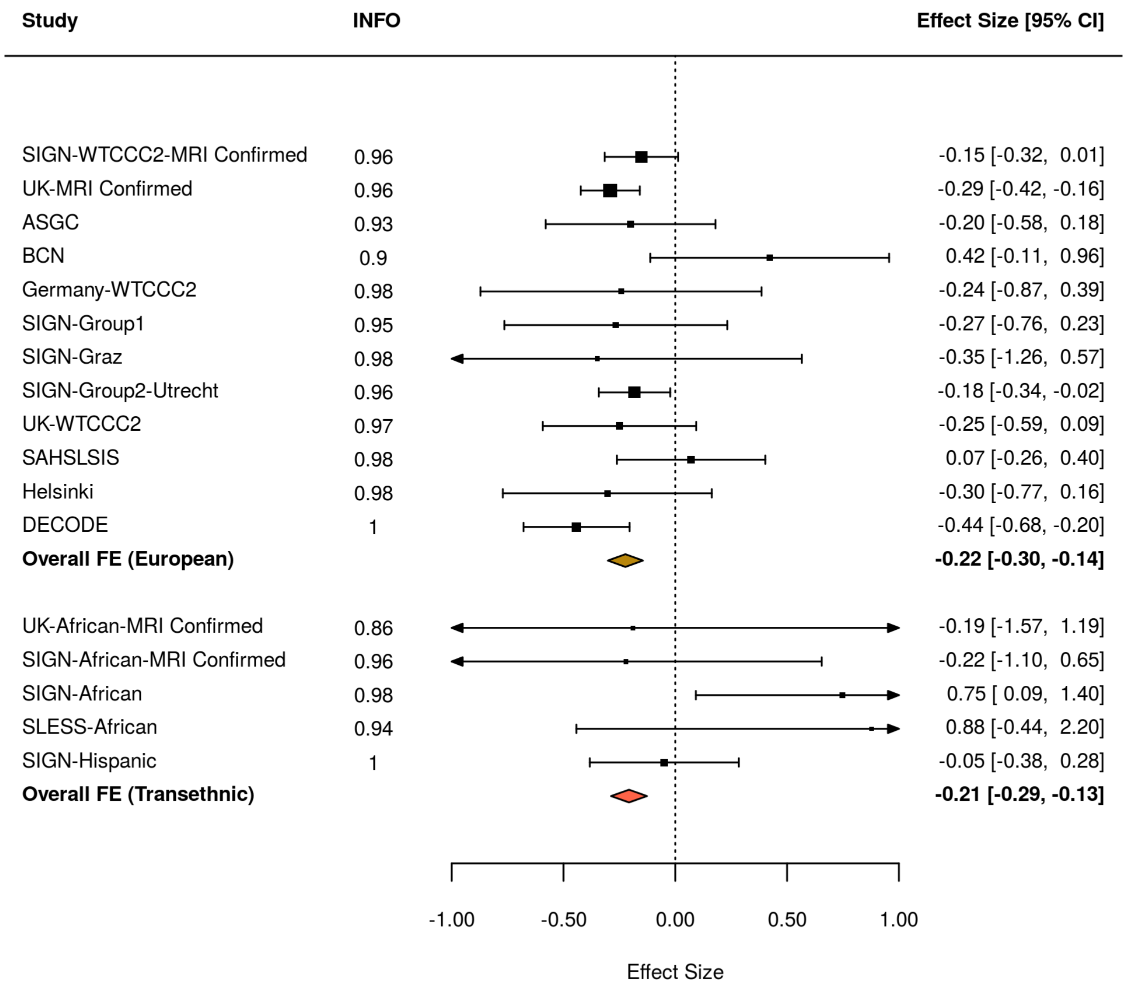

Heterogeneity I^2, Cochran's Q p-values: 19.6, 0.25 (European); 40.6, 0.05 (Transethnic)

Supplementary Figure 19 – Forest Plot for Association of rs9958650 at ZBTB14-EPB41L3 locus

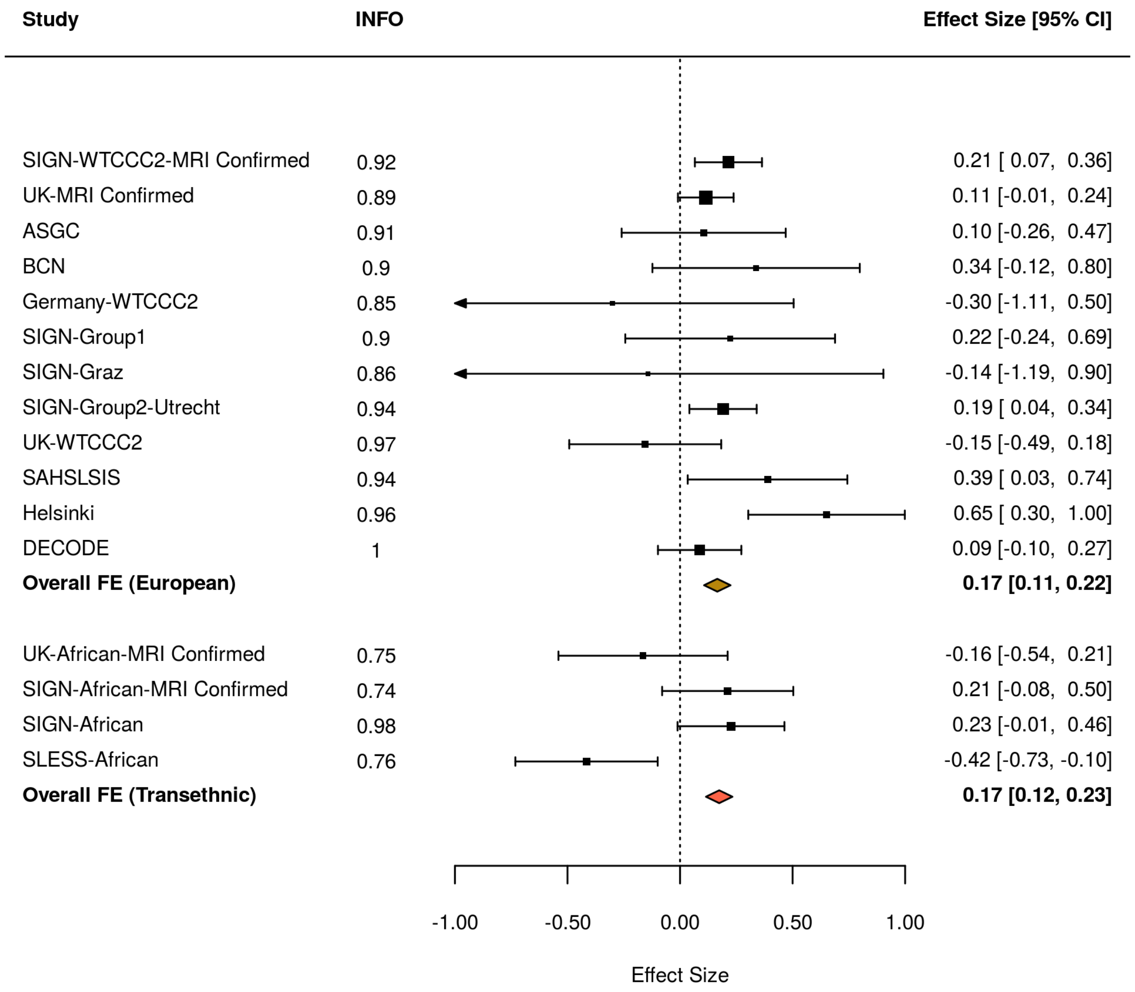

Heterogeneity I^2, Cochran's Q p-values: 33.2, 0.12 (European); 27.6, 0.14 (Transethnic)

Supplementary Figure 20 – Forest Plot for Association of rs12445022 at ZCCHC14 locus

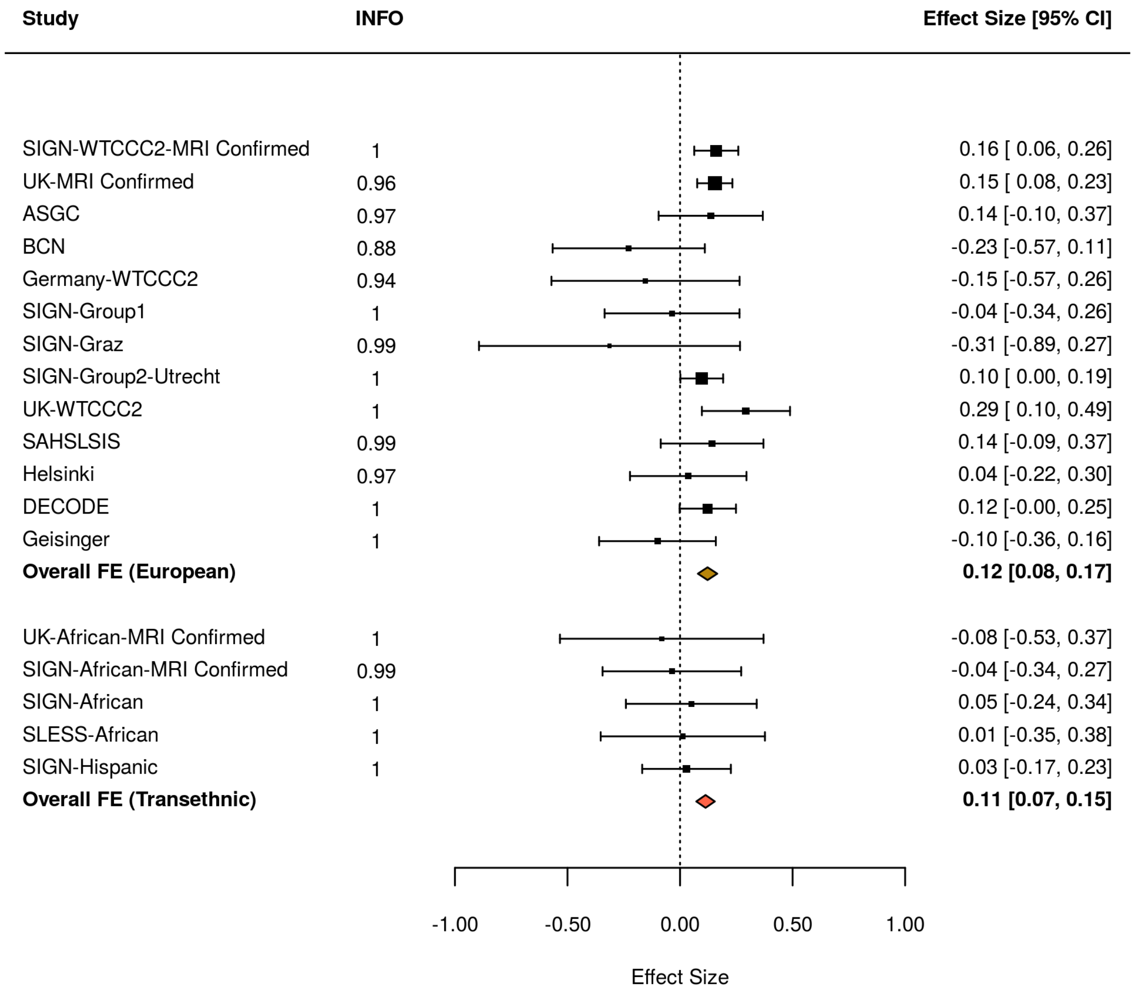

Heterogeneity I^2, Cochran's Q p-values: 26.6, 0.18 (European); 13.7, 0.29 (Transethnic)

Supplementary Figure 21 – Forest Plot for Association of rs2293576 at SPI1-SLC39A13-PSMC3-RAPSN locus

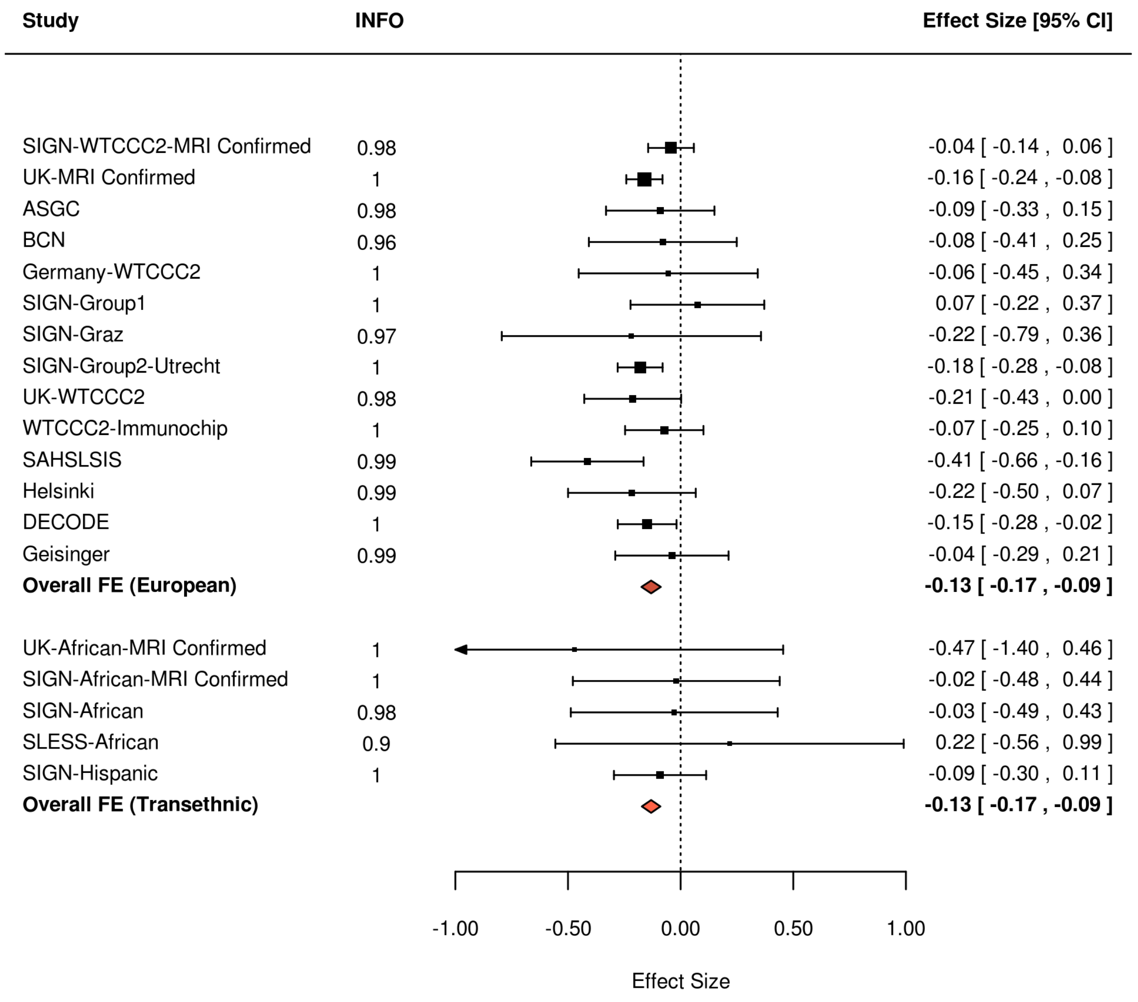

Heterogeneity I^2, Cochran's Q p-values: 1.8, 0.43 (European); 0.0, 0.65 (Transethnic)

Supplementary Figure 22 – Forest Plot for Association of rs4621303 at ULK4 locus

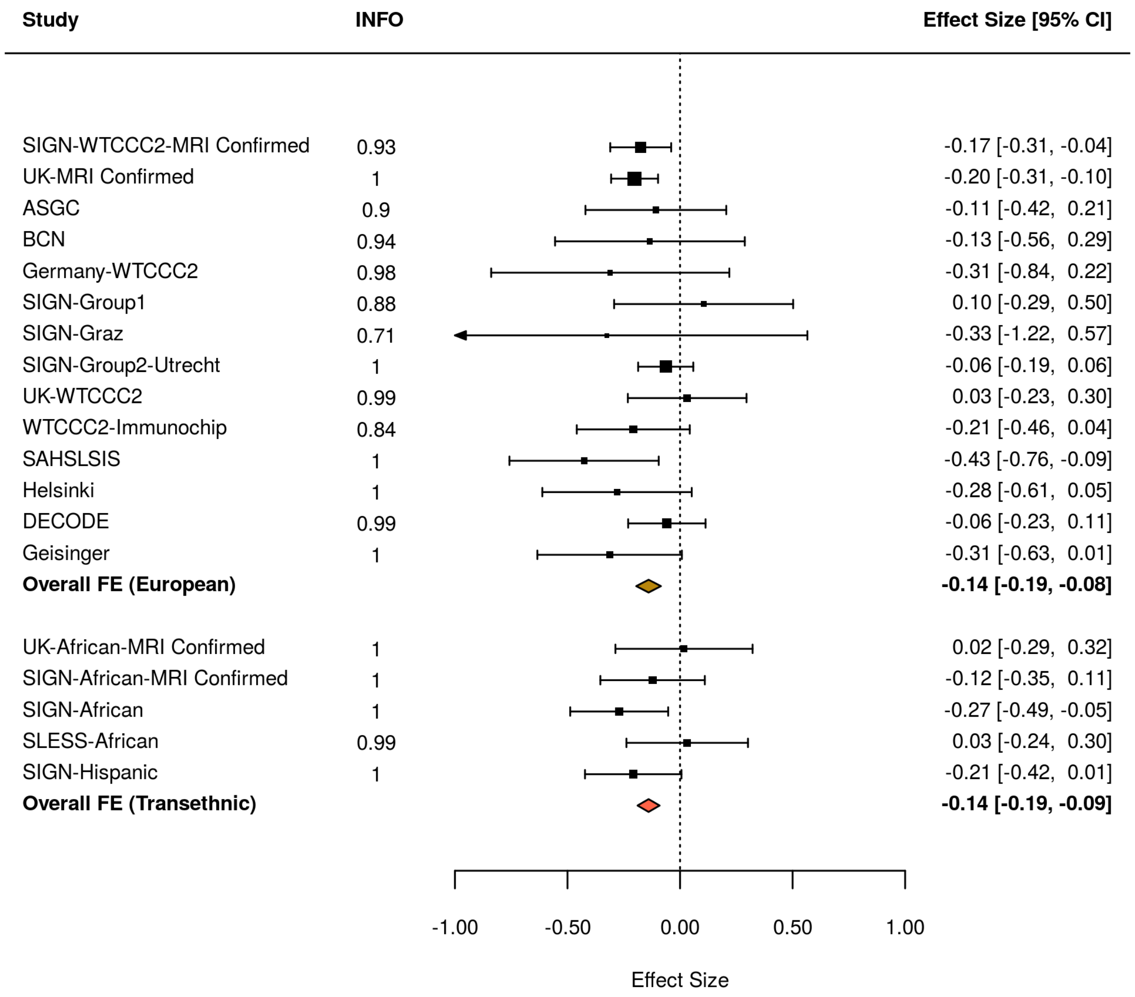

Heterogeneity I^2, Cochran's Q p-values: 0.0, 0.50 (European); 0.0, 0.56 (Transethnic)

Supplementary Figure 23 – Forest Plot for Association of rs2984613 at SLC25A44-PMF1-BGLAP locus

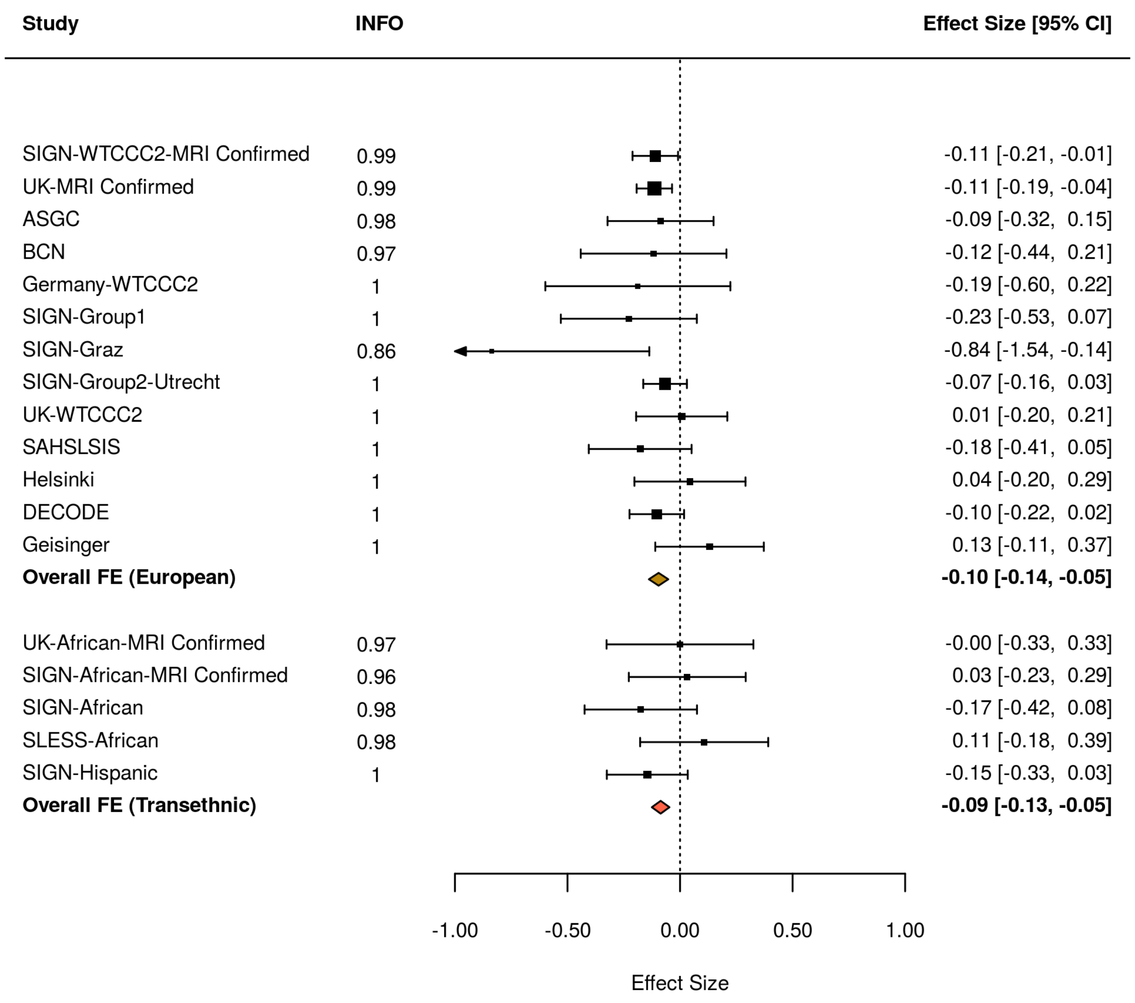

Heterogeneity I^2, Cochran's Q p-values: 0.0, 0.46 (European); 0.0, 0.57 (Transethnic)

Supplementary Figure 24 – Forest Plot for Association of rs2303655 at LOX-LOC10058541-ZNF474 locus

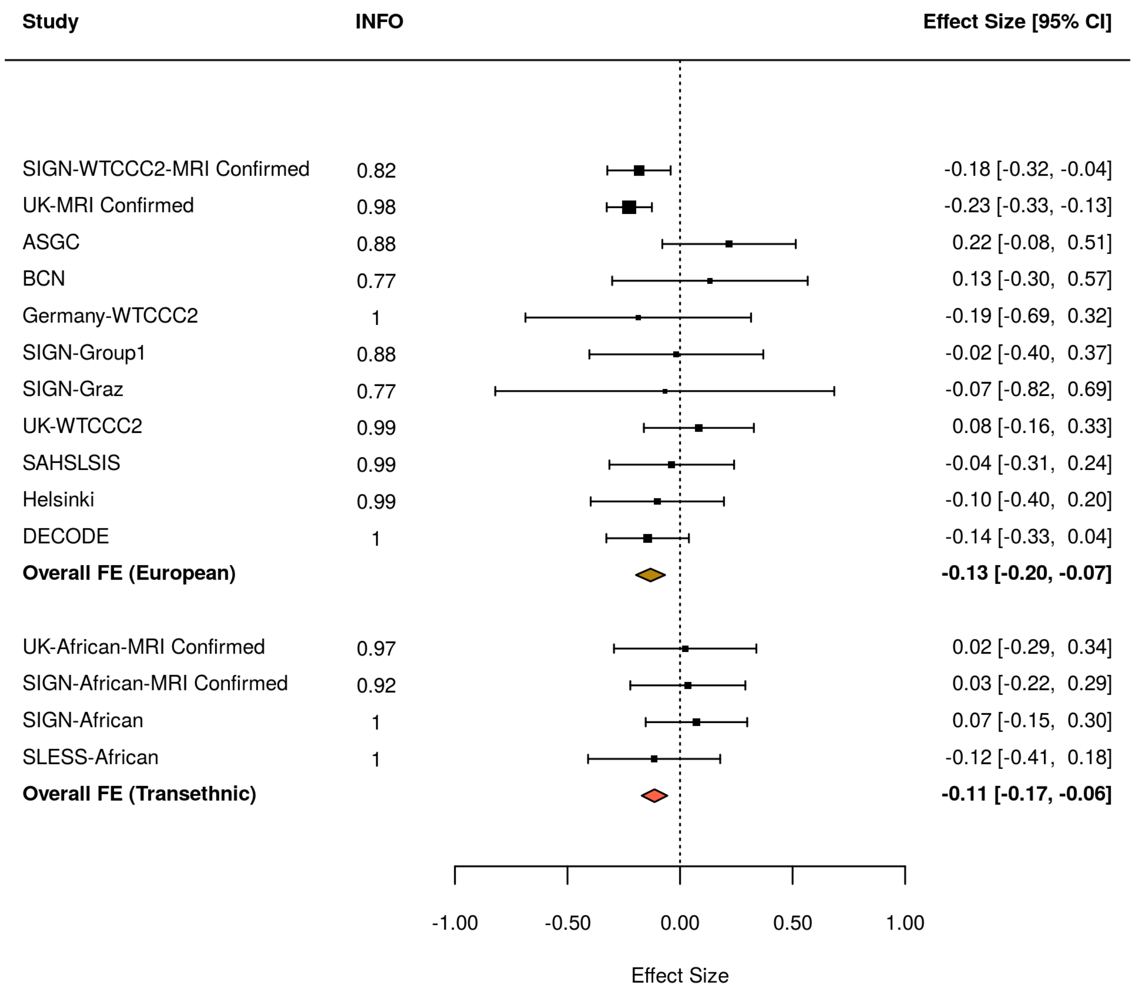

Heterogeneity I^2, Cochran's Q p-values: 29.6, 0.16 (European); 16.0, 0.27 (Transethnic)

Supplementary Figure 25 – Forest Plot for Association of rs12524544 at FOXF2-FOXQ1 locus

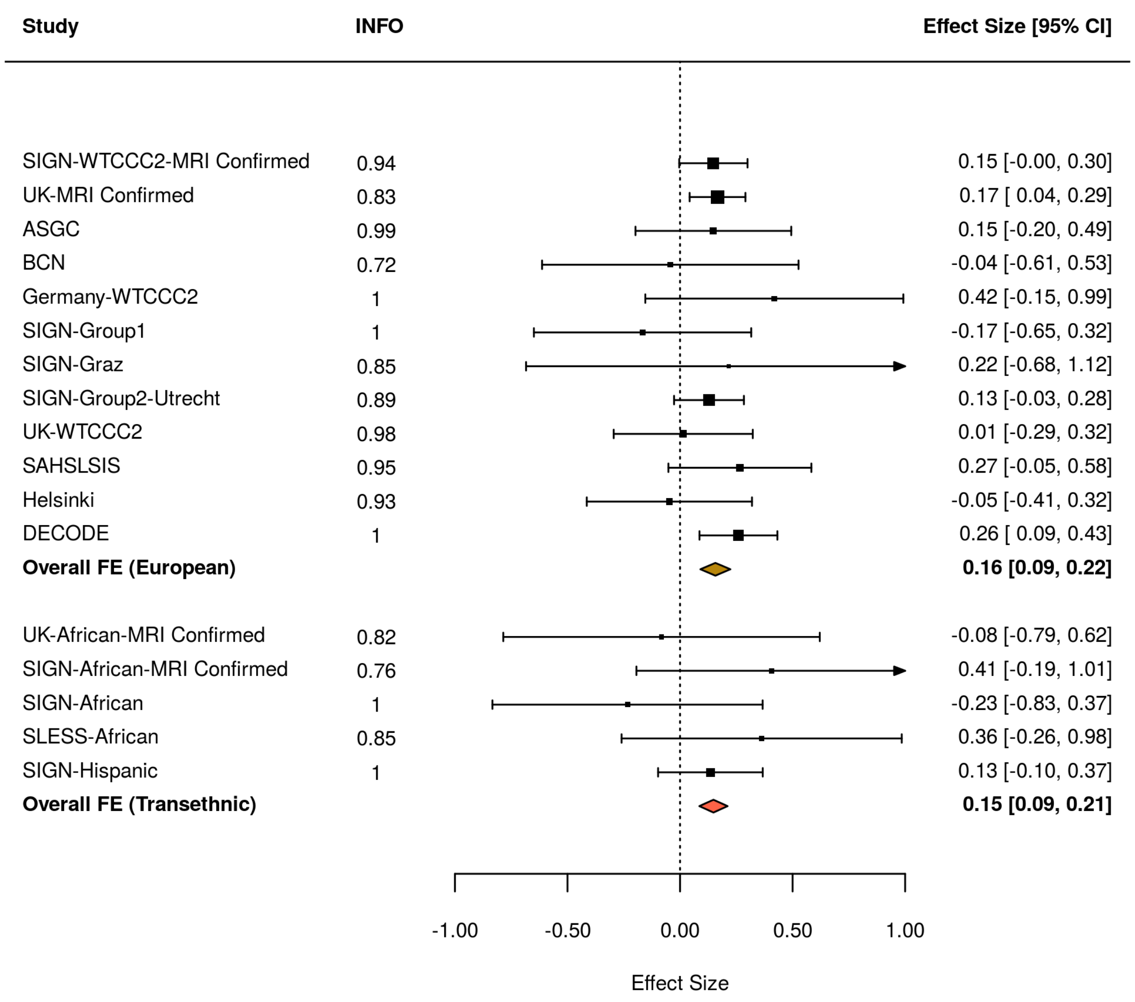

Heterogeneity I^2, Cochran's Q p-values: 0.0, 0.81 (European); 0.0, 0.82 (Transethnic)

Supplementary Figure 26 – Forest Plot for Association of rs225744 at GPR126-VTA1 locus

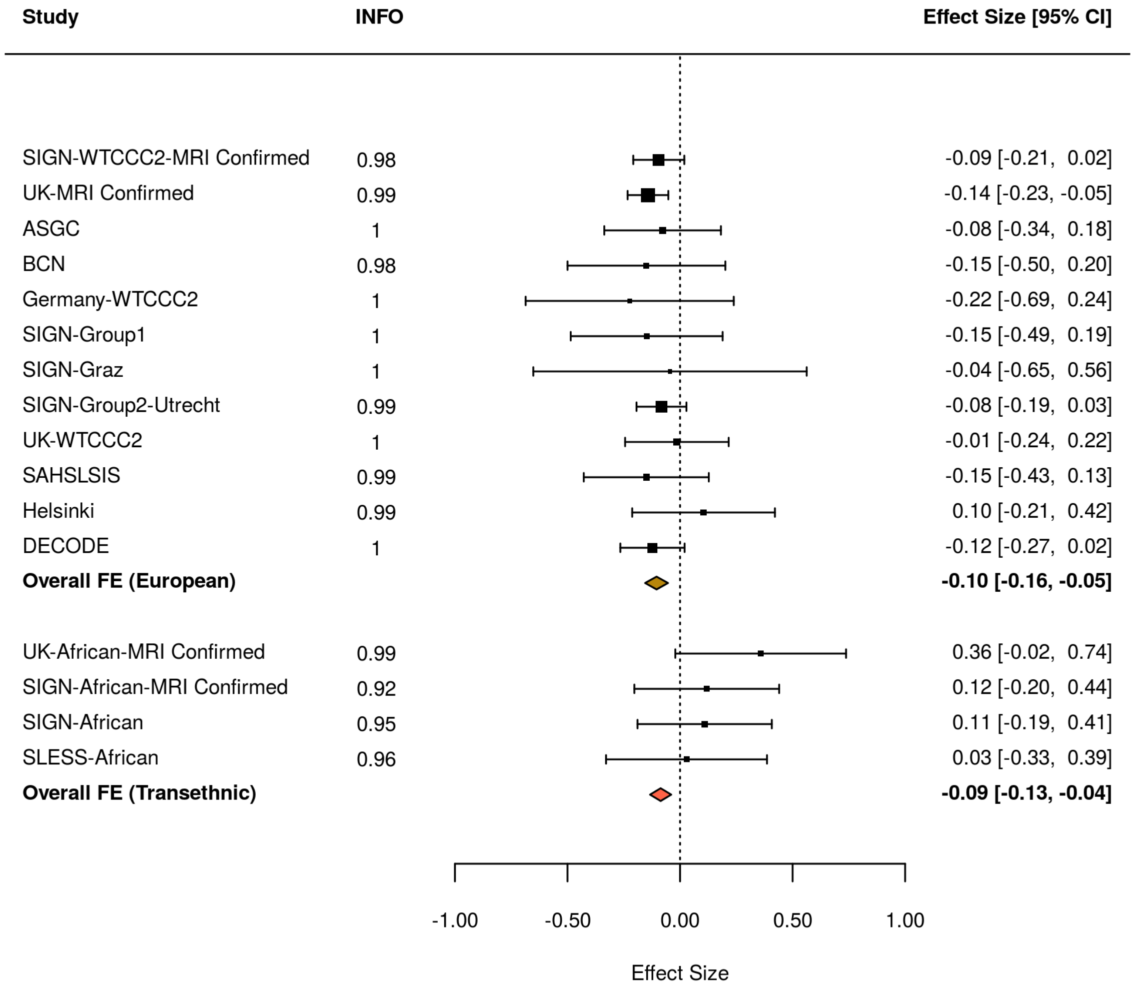

Heterogeneity I^2, Cochran's Q p-values: 0.0, 0.98 (European); 0.0, 0.64 (Transethnic)

Supplementary Figure 27 – Forest Plot for Association of rs79043147 at HTRA1/ARMS2 locus

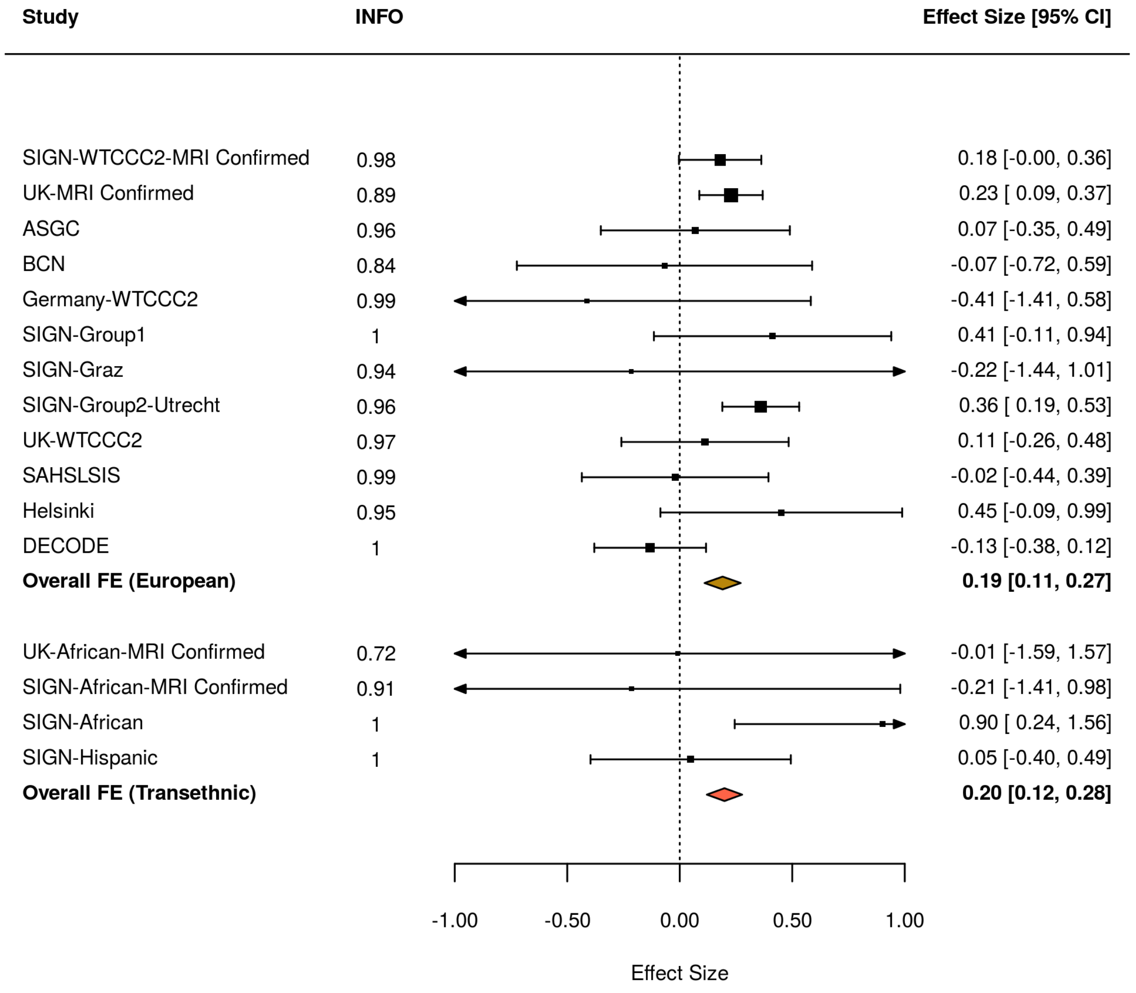

Heterogeneity I^2, Cochran's Q p-values: 34.6, 0.12 (European); 40.4, 0.06 (Transethnic)

Supplementary Figure 28 – Forest Plot for Association of rs11838776 at COL4A2 locus

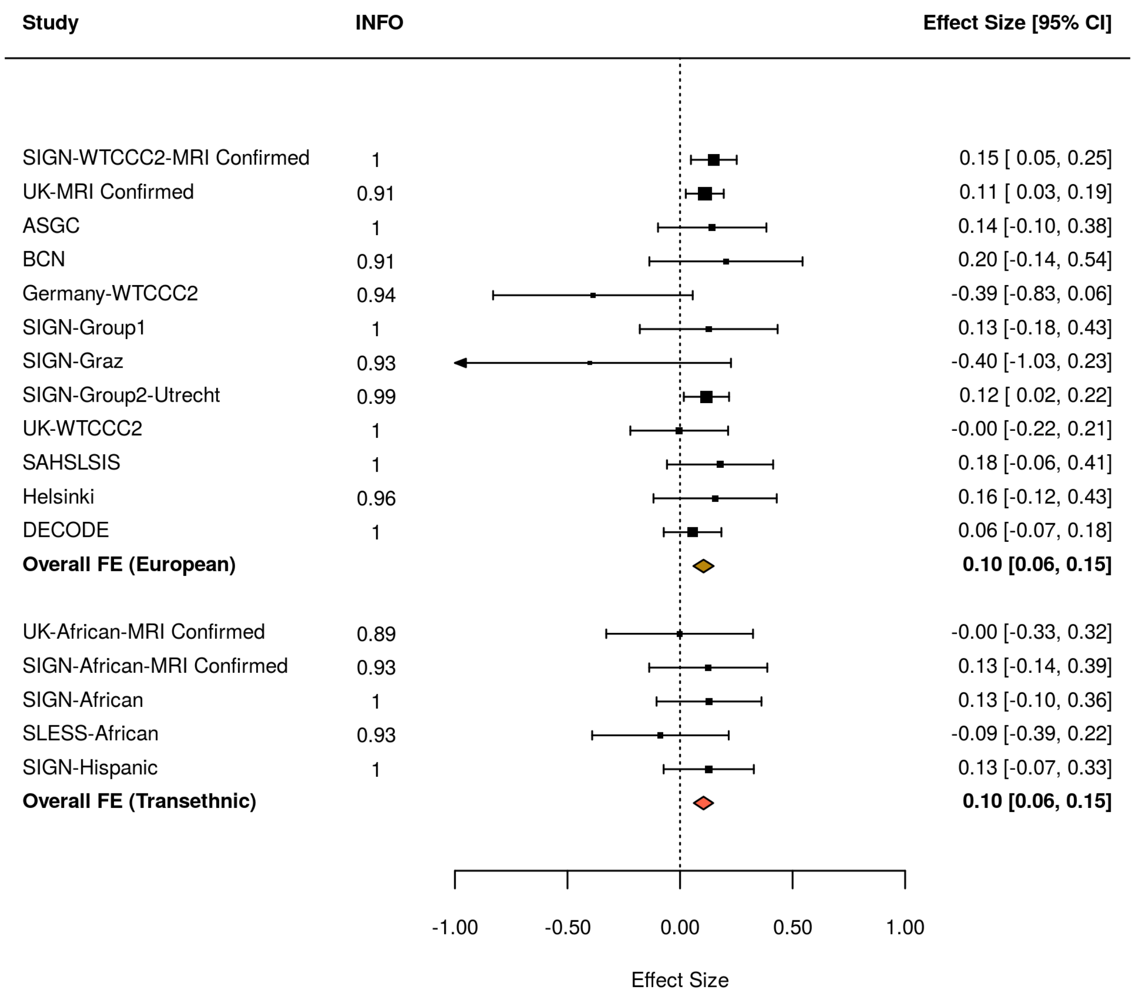

Heterogeneity I^2, Cochran's Q p-values: 0.0, 0.50 (European); 0.0, 0.66 (Transethnic)

Supplementary Figure 29 - Forest Plot for Association of rs61000833 at SH3PXD2A locus

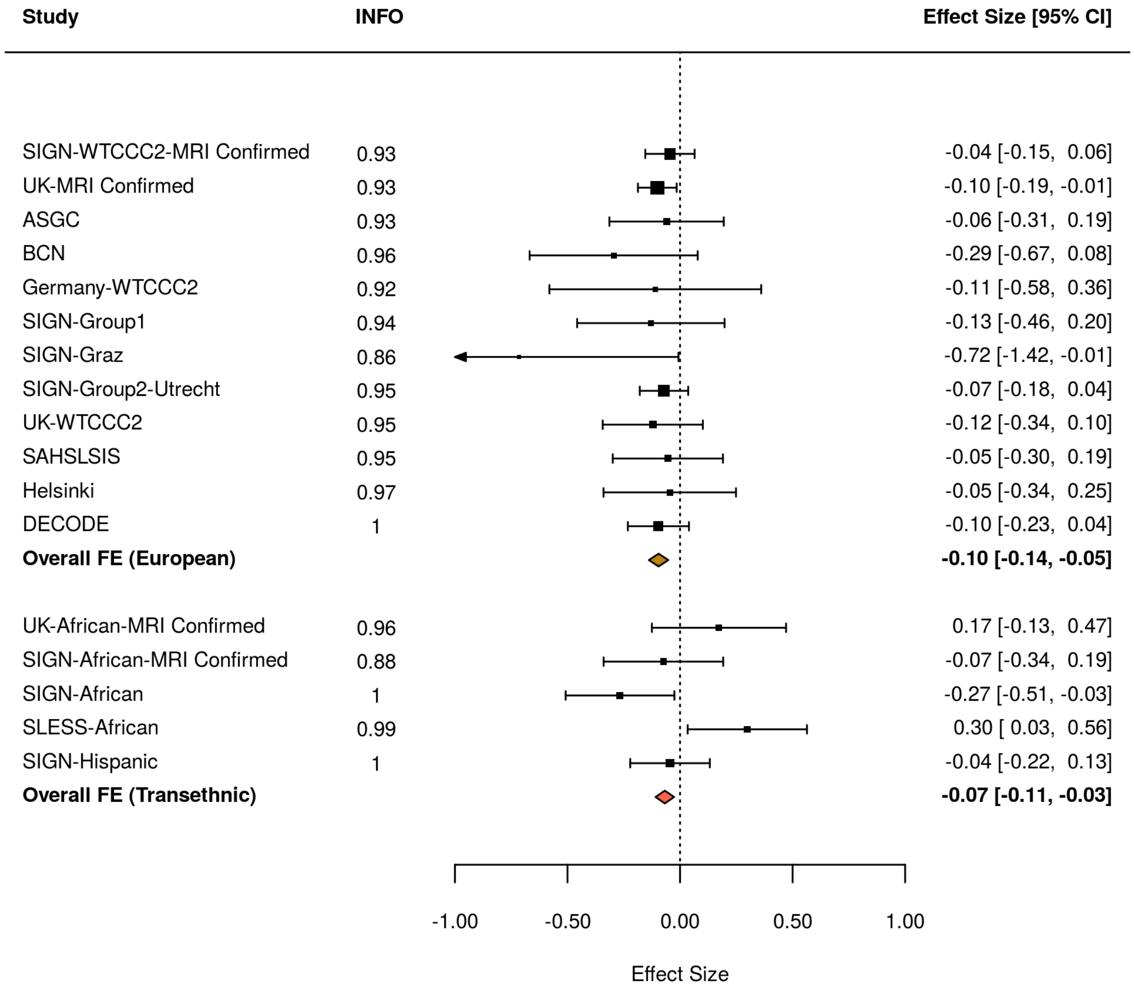

Heterogeneity I^2, Cochran's Q p-values: 0.0, 0.78 (European); 51.5, 0.009 (Transethnic)

Supplementary Figure 30 – Mendelian Randomization Results for Causal Impact of Body Mass Index on Lacunar Stroke

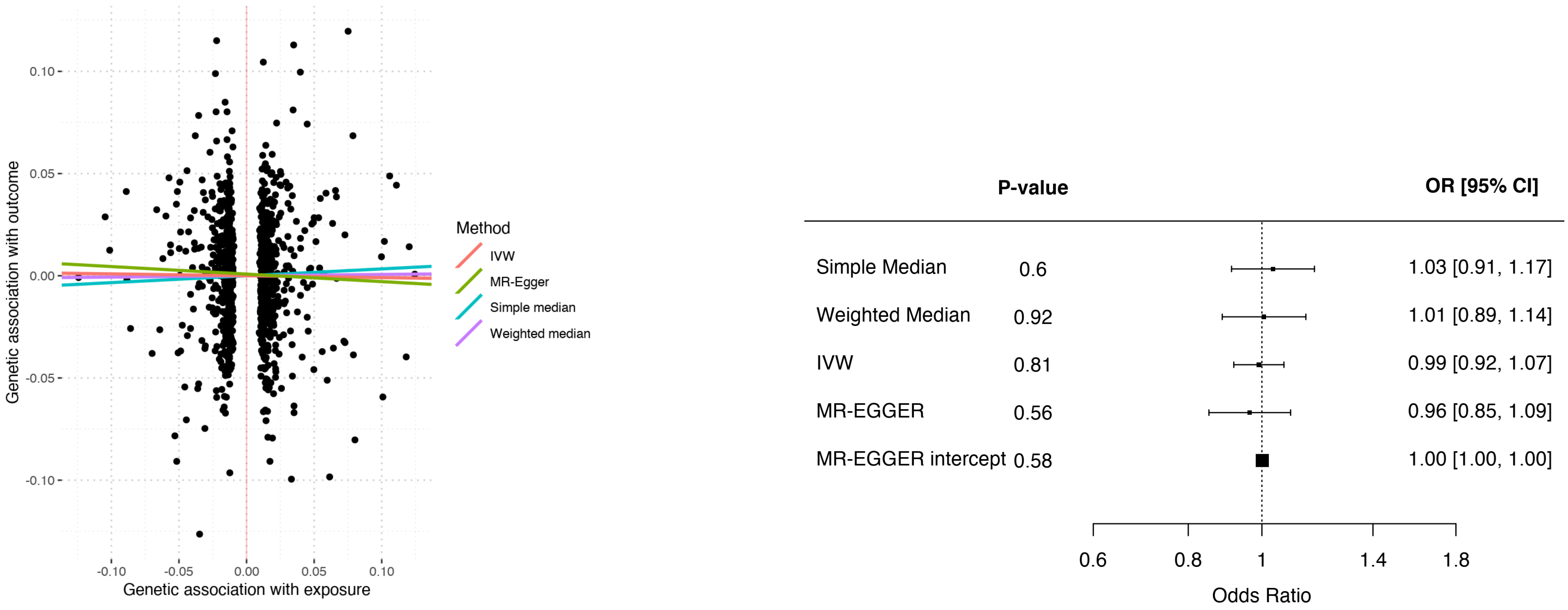

Supplementary Figure 31 – Mendelian Randomization Results for Causal Impact of Diastolic Blood Pressure on Lacunar Stroke

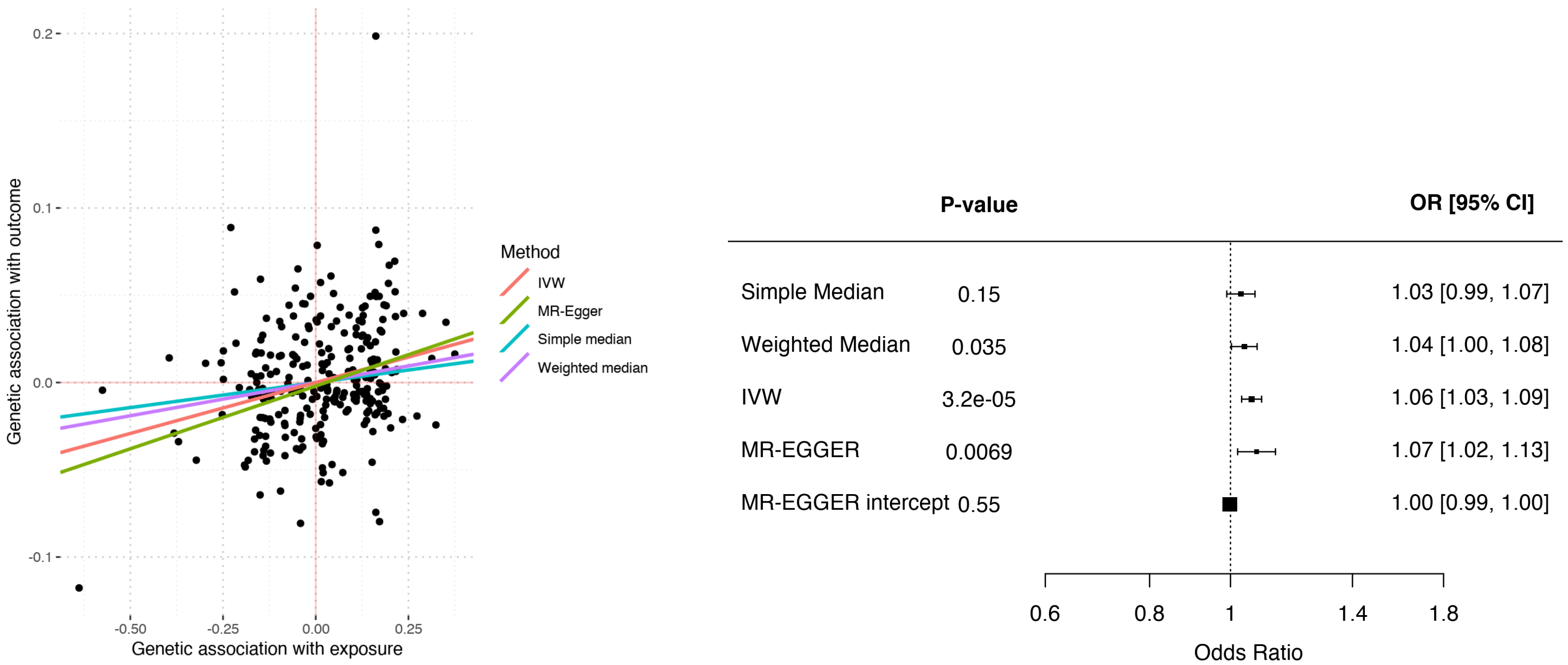

Supplementary Figure 32 – Mendelian Randomization Results for Causal Impact of High Density Lipoprotein on Lacunar Stroke

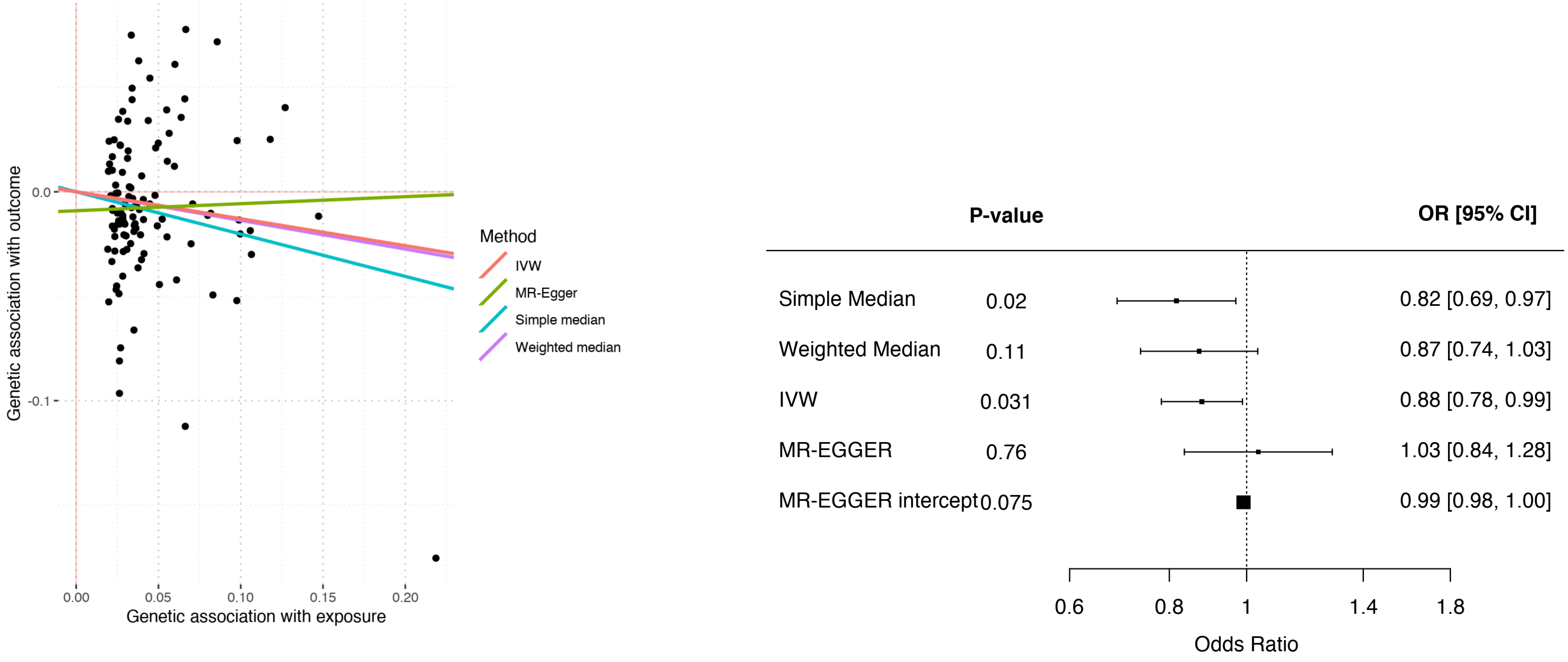

Supplementary Figure 33 – Mendelian Randomization Results for Causal Impact of Low Density Lipoprotein on Lacunar Stroke

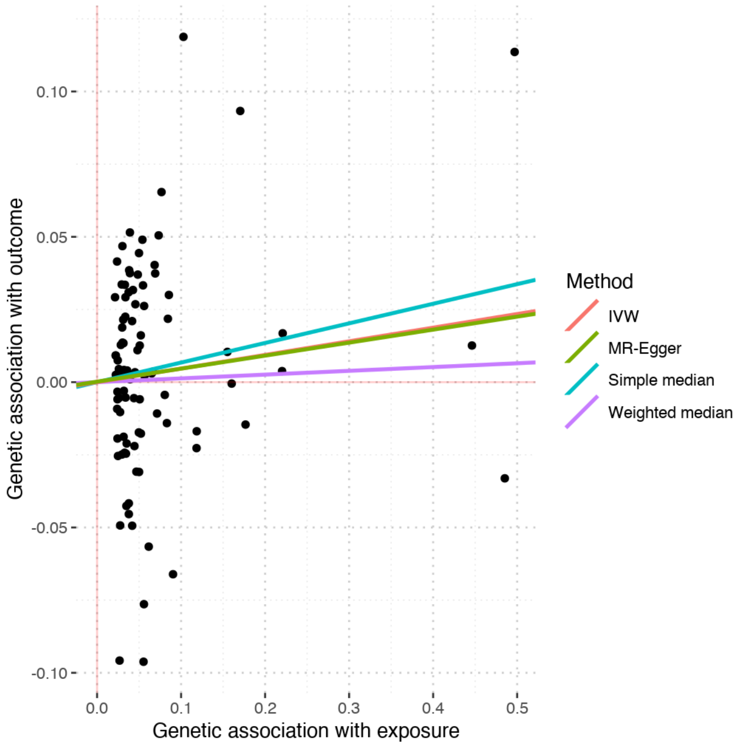

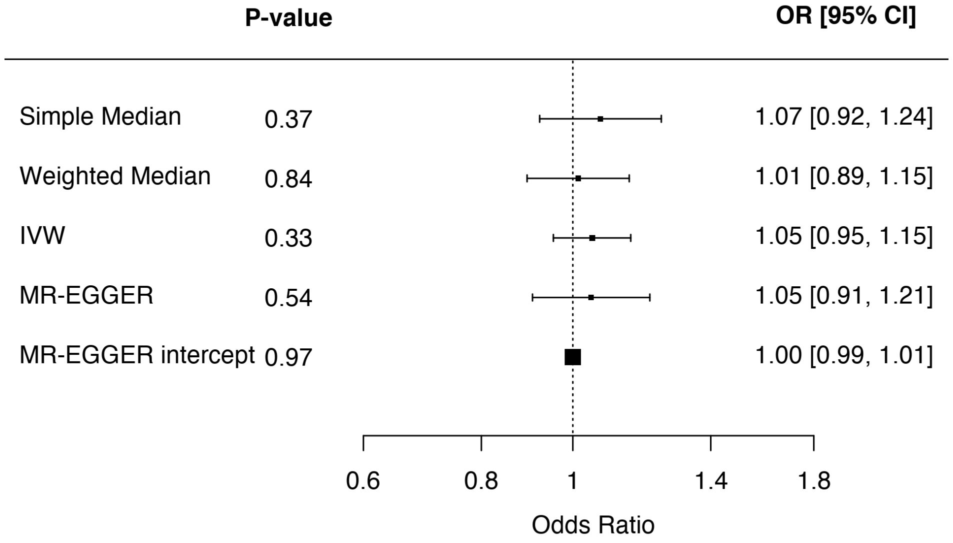

Supplementary Figure 34 – Mendelian Randomization Results for Causal Impact of Pulse Pressure on Lacunar Stroke

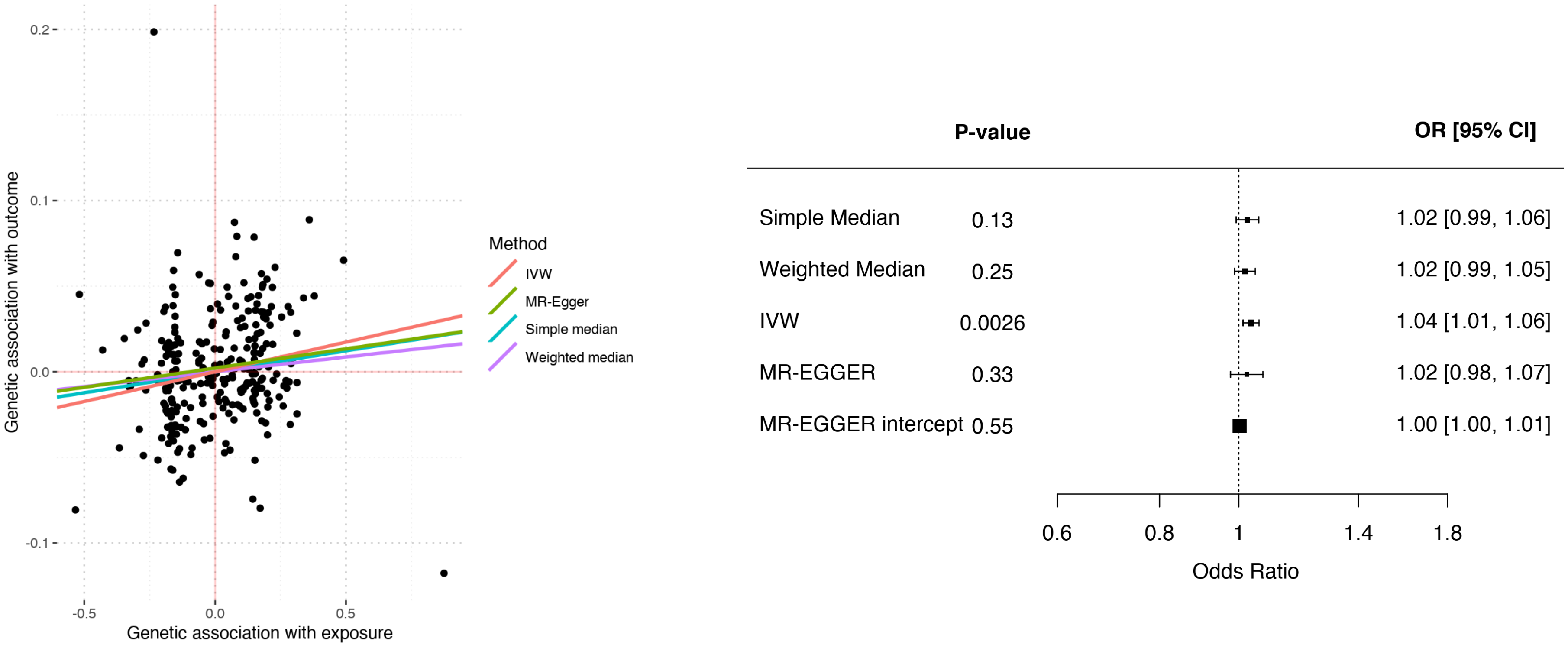

Supplementary Figure 35 – Mendelian Randomization Results for Causal Impact of Systolic Blood Pressure on Lacunar Stroke

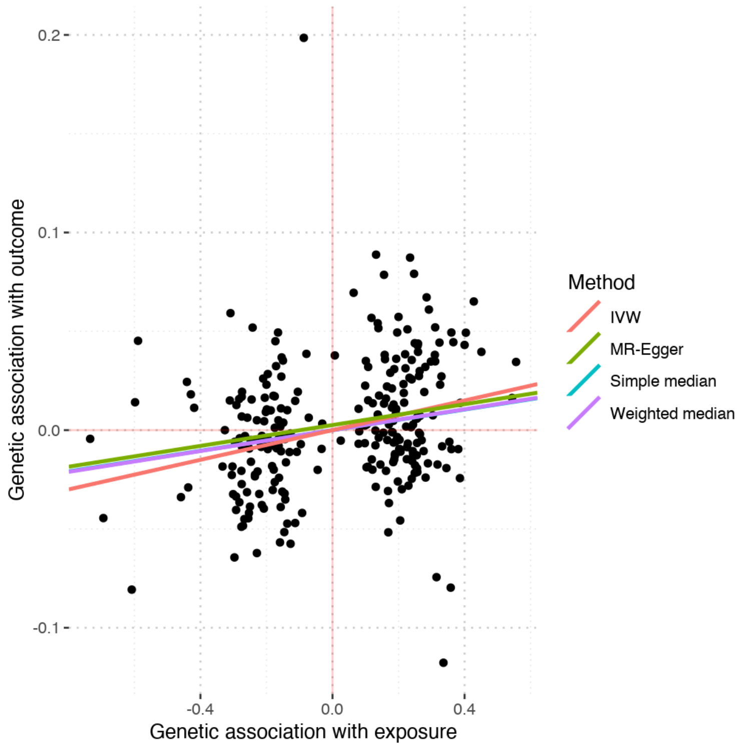

	P-value	OR [95% CI]
Simple Median	0.016	1.03 [1.00, 1.05]
Weighted Median	0.011	1.03 [1.01, 1.05]
IVW	3.3e-06	1.04 [1.02, 1.05]
MR-EGGER	0.26	1.03 [0.98, 1.07]
MR-EGGER intercept	0.61	1.00 [0.99, 1.01]

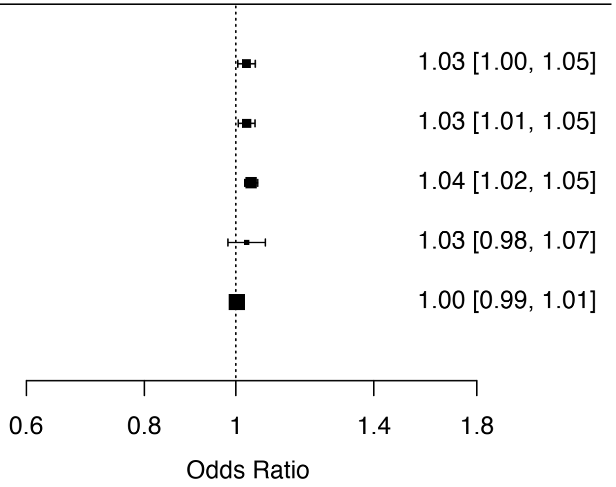

Supplementary Figure 36 – Mendelian Randomization Results for Causal Impact of Ever Smoking on Lacunar Stroke

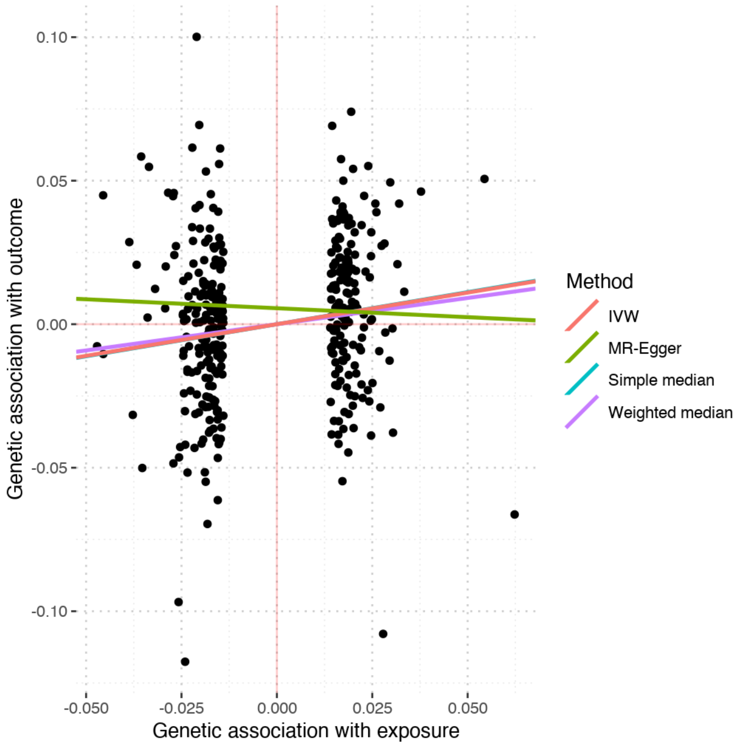

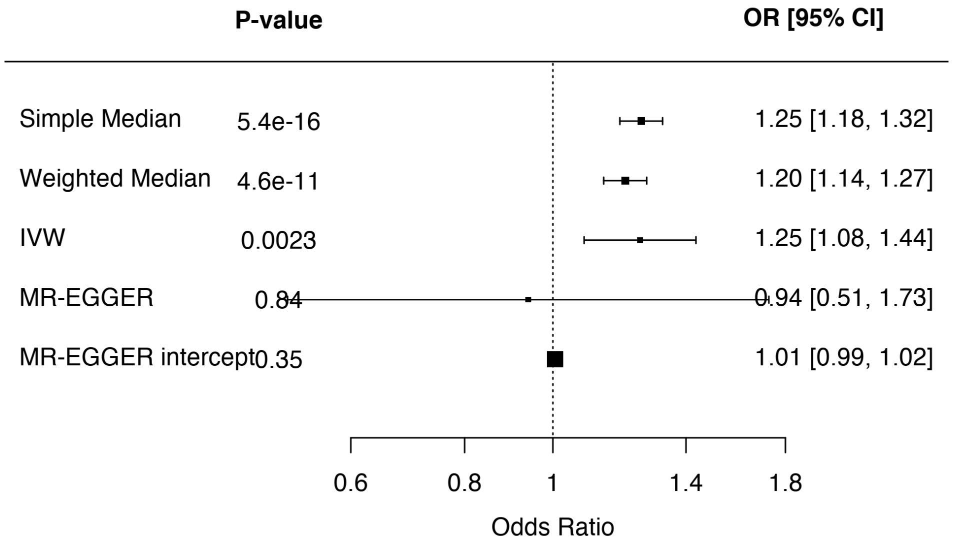

Supplementary Figure 37 – Mendelian Randomization Results for Causal Impact of Triglycerides on Lacunar Stroke

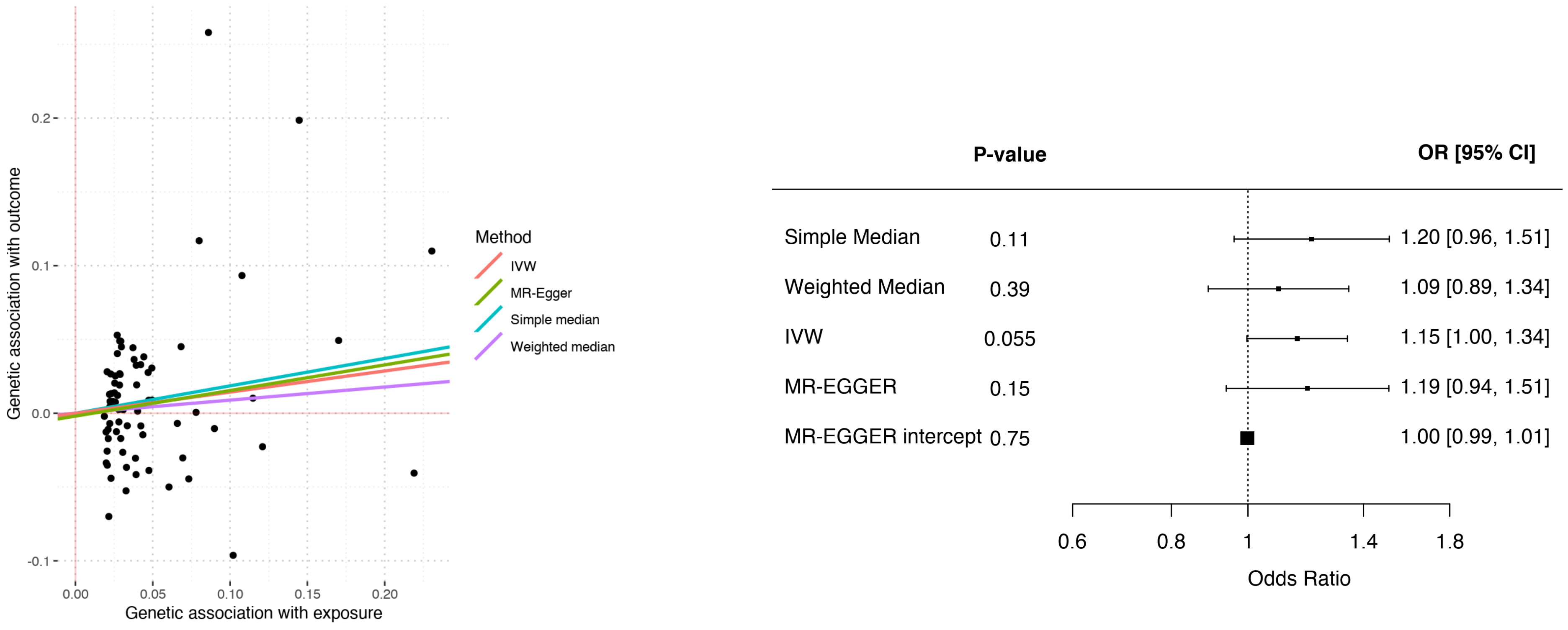

Supplementary Figure 38 – Mendelian Randomization Results for Causal Impact of Type 2 Diabetes on Lacunar Stroke

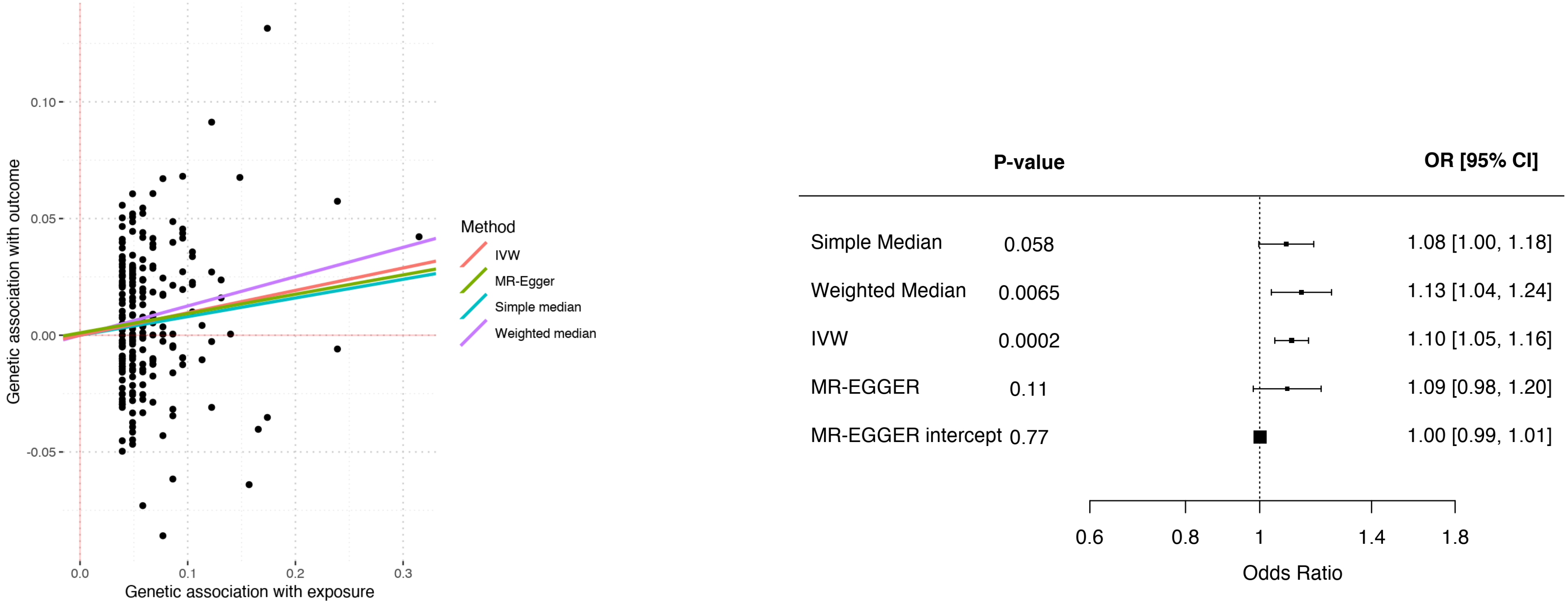

UK DNA Lacunar 2 Stroke Study Investigators

Participating Centres:

SITE ID	Hospital, Trust	PI
UK01	**Addenbrooke's Hospital**, Cambridge University Hospitals NHS Foundation Trust	Prof. Hugh Markus
UK02	**James Paget Hospital**, James Paget University Hospitals NHS Foundation Trust	Dr Carlo Canepa
UK03	**Peterborough City Hospital**, North West Anglia NHS Foundation Trust	Dr Santhosh Subramonian
UK04	**North Middlesex Hospital**, North Middlesex University Hospital NHS Trust	Dr Robert Luder
UK05	**Royal Stoke University Hospital**, University Hospital of North Midlands NHS Trust	Dr Janaka Weerathunga
UK06	**Royal London Hospital**, Barts Health NHS Trust	Dr Roser Icart
UK07	**West Suffolk Hospital**, West Suffolk NHS Foundation Trust	Dr Abul Azim
UK08	**Watford General Hospital**, West Hertfordshire Hospital Trust	Dr Mohit Bhandari
UK09	**Royal Cornwall Hospital**, Royal Cornwall Hospitals NHS Trust	Dr Frances Harrington
UK10	**New Cross Hospital**, The Royal Wolverhampton NHS Trust	Dr Nasar Ahmad
UK12	**King's Mill Hospital**, Sherwood Forest Hospitals Foundation Trust	Dr Martin Cooper
UK13	**King’s College Hospital (Denmark Hill) & Princess Royal University Hospital**, Kings College Hospital NHS Foundation Trust	Dr James Teo
UK14	**The Queen Elizabeth Hospital, Kings Lynn**, Queen Elizabeth Hospital Kings Lynn NHS Trust	Dr Umesh Rai
UK15	**Countess of Chester Hospital**, Countess of Chester Hospital NHS Foundation Trust	Dr Kausik Chatterjee
UK16	**Leeds General Infirmary**, Leeds Teaching Hospitals NHS Trust	Dr Ahammad Hassan
UK17	**Royal Hallamshire Hospital**, Sheffield Teaching Hospitals NHS Foundation Trust	Dr Kirsty Harkness
UK19	**University Hospital of North Tees and University Hospital of Hartlepool**, North Tees & Hartlepool Hospitals NHS Foundation Trust	Dr Ijaz Anwar
UK20	**Lister Hospital**, East & North Hertfordshire NHS Trust	Dr Aparna Pusalkar
UK21	**Royal Bournemouth General Hospital**, Royal Bournemouth & Christchurch NHS Foundation Trust	Dr Kamy Thavansan
UK22	**Royal Hampshire County Hospital**, Hampshire Hospitals Foundation Trust	Dr Nigel smyth
UK23	**Derriford Hospital**, Plymouth Hospitals NHS Trust	Dr Ahmed Nor
UK24*	**Torbay Hospital**, Torbay & South Devon NHS Foundation Trust	Dr Biju Bhaskaran
UK25	**Pinderfields Hospital**, Mid Yorkshire Hospitals NHS Trust	Dr Prabal Datta
UK27	**Royal Devon & Exeter Hospital (Wonford)**, Royal Devon & Exeter NHS Foundation Trust	Dr Martin James
UK28	**University College Hospital**, University College London Hospitals NHS Foundation Trust	Prof David Werring
UK29	**Nottingham City Hospital Campus**, Nottingham University Hospitals NHS Trust	Dr Kailash Krishnan
UK30	**Salford Royal Hospital**, Salford Royal NHS Foundation Trust	Dr Jane Molloy
UK31	**James Cook University Hospital**, South Tees Hospitals NHS Foundation Trust	Dr Adrian Bergin
UK32	**University Hospital of North Durham**, County Durham and Darlington NHS Foundation Trust	Dr Yogish Pai
UK33	**Yeovil District Hospital**, Yeovil District Hospital NHS Foundation Trust	Dr Khalid Rashed
UK34	**St Peter’s Hospital**, Ashford & St Peters Hospitals NHS Foundation Trust	Dr Brendan Affley
UK35	**Sandwell General Hospital**, Sandwell and West Birmingham Hospitals NHS Trust	Dr David Gull
UK36	**St Thomas’ Hospital**, Guys & St Thomas' Hospitals NHS Foundation Trust	Dr Jonathan Birns
UK37*	**Royal Sussex County Hospital**, Brighton & Sussex University Hospitals NHS Trust	Prof Raj Rajkumar
UK38	**Southampton General Hospital**, University Hospital Southampton NHS Foundation Trust	Dr Emma Battersby-Wood
UK39	**Doncaster Royal Infirmary**, Doncaster & Bassetlaw Teaching Hospitals NHS Foundation Trust	Dr Dinesh Chada
UK40	**St George's Hospital (Tooting)**, St George’s University Hospitals NHS Foundation Trust	Dr Bhavini Patel
UK41	**Wycombe Hospital**, Buckinghamshire Healthcare NHS Trust	Dr Matthew Burn
UK42	**Musgrove Park Hospital**, Somerset NHS Foundation Trust	Dr Malik Hussain
UK43	**Scunthorpe General Hospital**, Northern Lincolnshire and Goole NHS Foundation Trust	Dr Amit Banerjee
UK44	**Ipswich Hospital**, East Suffolk and North Essex NHS Foundation Trust	Dr Sajid Alam
UK45	**Southend University Hospital**, Southend University Hospital NHS Foundation Trust	Dr Paul Guyler
UK46	**Gloucestershire Royal Hospital**, Gloucestershire Hospitals NHS Foundation Trust	Dr Dipankar Dutta
UK47	**Queen Elizabeth The Queen Mother Hospital**, East Kent Hospitals University NHS Foundation Trust	Dr Saidu Abubakar
UK48	**Colchester Hospital**, East Suffolk and North Essex NHS Foundation Trust	Dr Rajesh Kumar Saksena

SITE ID	Hospital, Trust	PI
UK49	**Royal Derby Hospital**, University Hospitals of Derby and Burton NHS Foundation Trust	Dr Tim England
UK50	**Croydon University Hospital**, Croydon Health Services NHS Trust	Dr Karen Kee
UK51	**Norfolk & Norwich University Hospital**, Norfolk and Norwich University Hospitals NHS Foundation Trust	Dr Kneale Metcalf
UK52	**Leicester Royal Infirmary**, University Hospitals of Leicester NHS Trust	Dr Amit Mistri
UK53	**William Harvey Hospital**, East Kent Hospitals University NHS Foundation Trust	Dr Ibrahim Balogun
UK54	**Medway Maritime Hospital**, Medway NHS Foundation Trust	Dr Samuel Sanmuganathan

UK DNA Lacunar Stroke Study Investigators

Study managers: Josie Monaghan; Alan Zanich, Samantha Febrey, Eithne Smith, Jenny Lennon, St George's University of London

Database cleaning: Loes Rutten-Jacobs, University of Cambridge

Participating centres (number of enrolled patients per centre; local investigators):

Aberdeen Royal Infirmary, Aberdeen (12; Mary Macleod). Addenbrooke's Hospital, Cambridge (54; Jean-Claude Baron, Elizabeth Warburton, Diana J Day, Julie White). Airedale General Hospital, Steeton (4; Samantha Mawer). Barnsley Hospital, Barnsley (3; Mohammad Albazzaz, Pravin Torane, Keith Elliott, Kay Hawley). Bart's and the London, London (2; Patrick Gompertz). Basingstoke and North Hampshire Hospital, Basingstoke (13; Elio Giallombardo, Deborah Dellafera). Blackpool Victoria Hospital, Blackpool (11; Mark O'Donnell). Bradford Royal Infirmary, Bradford (1; Chris Patterson). Bristol Royal Infirmary, Bristol (8; Sarah Caine). Charing Cross Hospital, London (12; Pankaj Sharma). Cheltenham General and Gloucester Royal Hospitals, Cheltenham and Gloucester (10; Dipankar Dutta). Chesterfield Royal Hospital, Chesterfield (4; Sunil Punnoose, Mahmud Sajid). Countess of Chester Hospital, Chester (22; Kausik Chatterjee). Derriford Hospital, Plymouth (4; Azlisham Mohd Nor). Dorset County Hospital NHS Foundation Trust, Dorchester (6; Rob Williams). East Kent Hospitals University NHS Foundation Trust, Kent (22; Hardeep Baht, Guna Gunathilagan). Eastbourne District General Hospital, Eastbourne (4; Conrad Athulathmudali). Frenchay Hospital, Bristol (1; Neil Baldwin). Frimley Park Hospital NHS Foundation Trust, Frimley (6; Brian Clarke). Guy's and St Thomas' Hospital, London (14; Tony Rudd). Institute of Neurology, London (25; Martin Brown). James Paget University Hospital, Great Yarmouth (1; Peter Harrison). King's College Hospital, London (16; Lalit Kalra). Leeds Teaching Hospitals NHS Trust, London (125; Ahamad Hassan). Leicester General Hospital and Royal Infirmary, Leicester (9; Tom Robinson, Amit Mistri). Luton and Dunstable NHSFT University Hospital, Luton (16; Lakshmanan Sekaran, Sakthivel Sethuraman, Frances Justin). Maidstone and Tunbridge Wells NHS Trust (3; Peter Maskell). Mayday University Hospital, Croydon (14; Enas Lawrence). Medway Maritime Hospital, Gillingham (5; Sam Sanmuganathan). Milton Keynes Hospital, Milton Keynes (1; Yaw Duodu). Musgrove Park Hospital, Taunton (9; Malik Hussain). Newcastle Hospitals NHS Foundation Trust, Newcastle upon Tyne (12; Gary Ford). Ninewells Hospital, Dundee (5; Ronald MacWalter). North Devon District Hospital, Barnstaple (8; Mervyn Dent). Nottingham University Hospitals, Nottingham (17; Philip Bath, Fiona Hammonds). Perth Royal Infirmary, Perth (2; Stuart Johnston). Peterborough City Hospital, Peterborough (1; Peter Owusu-Agyei). Queen Elizabeth Hospital, Gateshead (5; Tim Cassidy, Maria Bokhari). Radcliffe Infirmary, Oxford (5; Peter Rothwell). Rochdale Infirmary, Rochdale (4; Robert Namushi). Rotherham General Hospital, Rotherham (1; James Okwera). Royal Cornwall Hospitals NHS Trust, Truro (11; Frances Harrington, Gillian Courtauld). Royal Devon and Exeter Hospital, Exeter (22; Martin James). Royal Hallamshire Hospital, Sheffield (1; Graham Venables). Royal Liverpool University Hospital and Broadgreen Hospital, Liverpool (9; Aravind Manoj). Royal Preston Hospital, Preston (18; Shuja Punekar). Royal Surrey County Hospital, Guildford (23; Adrian Blight, Kath Pasco). Royal Sussex County Hospital, Brighton (14; Chakravarthi Rajkumar, Joanna Breeds). Royal United Hospital, Bath (6; Louise Shaw, Barbara Madigan). Salford Royal Hospital, Salford (16; Jane Molloy). Southampton General Hospital, Southampton (1; Giles Durward). Southend Hospital, Westcliff-on-Sea (26; Paul Guyler). Southern General Hospital, Glasgow (34; Keith Muir, Wilma Smith). St George's Hospital, London (108; Hugh Markus). St Helier Hospital, Carshalton (10; Val Jones). Stepping Hill Hospital, Stockport (4; Shivakumar Krishnamoorthy). Sunderland Royal Hospital, Sunderland (1; Nikhil Majumdar). The Royal Bournemouth Hospital, Bournemouth (15; Damian Jenkinson). The Walton Centre, Liverpool (15; Richard White). Torbay Hospital, Torquay (19; Debs Kelly). University Hospital Aintree, Liverpool (19; Ramesh Durairaj). University Hospital of North Staffordshire, Stoke-on-trent (16; David Wilcock). Wansbeck General Hospital and North Tyneside Hospital, Ashington and North Shields (6; Christopher Price). West Cumberland Hospital, Whitehaven (6; Olu Orugun, Rachel Glover). West Hertfordshire Hospital, Watford (20; David Collas). Western General Hospital, Edinburgh (12; Cathie Sudlow). Western Infirmary, Glasgow (33; Kennedy R. Lees, Jesse Dawson). Wycombe Hospital and Stoke Mandeville, High Wycombe (20; Dennis Briley and Matthew Burn). Yeovil District Hospital, Yeovil (46; Khalid Rashed). York Teaching Hospital, York (1; John Coyle).

The Dutch Parelsnoer Institute-Cerebrovascular accident (CVA) Study Group, The Netherlands

Ewoud J. van Dijk, Department of Neurology, Radboud University Medical Center, Nijmegen, The Netherlands
Peter J. Koudstaal, Department of Neurology, Erasmus University Medical Center, Rotterdam, The Netherlands
Gert-Jan Luijckx, Department of Neurology, Maastricht University Medical Center, Maastricht, The Netherlands
Paul J. Nederkoorn, Department of Neurology, Amsterdam UMC, University of Amsterdam, Amsterdam, The Netherlands
Robert J. van Oostenbrugge, Department of Neurology, Maastricht University Medical Center, Maastricht, The Netherlands
Marieke C. Visser, Department of Neurology, Amsterdam UMC, Vrije Universiteit Amsterdam, Amsterdam, the Netherlands
Marieke J.H. Wermer, Department of Neurology, Leiden University Medical Center, Leiden, the Netherlands
L. Jaap Kappelle, Department of Neurology and Neurosurgery, University Medical Center Utrecht Brain Center, Utrecht University, Utrecht, The Netherlands

Members of the Stroke Genetics Network (SiGN)

Administrative:
Steven J. Kittner, MD, MPH (chair; Department of Neurology, University of Maryland School of Medicine and Veterans Affairs Maryland Health Care System, Baltimore, MD, USA); Cameron A. Dell, BS (Department of Neurology, University of Maryland School of Medicine, Baltimore, MD, USA);
Dale M. Gamble, MHSc, CCRP (Department of Neurology, Mayo Clinic, Jacksonville, FL, USA);
Mary J. Sparks, RN, BSN (Department of Neurology, University of Maryland School of Medicine, Baltimore, MD, USA)

Steering/PIs of Discovery Studies:
Donna K. Arnett, PhD, MSPH (Department of Epidemiology, School of Public Health, University of Alabama at Birmingham, Birmingham, AL, USA); Oscar Benavente, MD, FRCP (Department of Neurology, University of British Columbia, Vancouver, British Columbia, Canada); John W. Cole, MD, MS (Department of Neurology, University of Maryland School of Medicine and Veterans Affairs Maryland Health Care System, Baltimore, MD, USA); Martin Dichgans, MD (Institute for Stroke and Dementia Research, Klinikum der Universität München, Ludwig-Maximilians University, Munich, Germany; Munich Cluster for Systems Neurology (SyNergy), Munich, Germany); Raji P. Grewal, MD (Neuroscience Institute, Saint Francis Medical Center, School of Health and Medical Sciences, Seton Hall University, South Orange, New Jersey, USA); Christina Jern, MD, PhD (Institute of Biomedicine, the Sahlgrenska Academy at University of Gothenburg, Gothenburg, Sweden); Jordi Jiménez Conde, MD, PhD (Department of Neurology, Neurovascular Research Group (NEUVAS) IMIM-Hospital del Mar (Institut Hospital del Mar d'Investigacions Mèdiques), Universitat Autonoma de Barcelona/DCEXS-Universitat Pompeu Fabra, Barcelona, Spain); Julie A. Johnson, PharmD (Department of Pharmacotherapy and Translational Research and Center for Pharmacogenomics, College of Pharmacy, University of Florida, Gainesville, FL, USA; Division of Cardiovascular Medicine, College of Medicine, University of Florida, Gainesville, FL, USA);
Jin-Moo Lee, MD, PhD (Stroke Center, Department of Neurology, Washington University School of Medicine, St. Louis, MO, USA); Christopher Levi, MBBS, BMed Sci, FRACP (John Hunter Hospital, Hunter Medical Research Institute and University of Newcastle, NSW, Australia);
Arne Lindgren, MD PhD (Department of Clinical Sciences Lund, Neurology, Lund University, Lund, Sweden; Department of Neurology and Rehabilitation Medicine, Neurology, Skåne, University Hospital, Lund, Sweden); Hugh S. Markus, DM (Department of Clinical Neurosciences, University of Cambridge, Cambridge, UK); Olle Melander, MD, PhD (Lund University, Department of Clinical Sciences, Malmö University Hospital, Malmö, Sweden); James F. Meschia, MD (Department of Neurology, Mayo Clinic, Jacksonville, FL, USA); Kathryn Rexrode, MD, MPH (Brigham and Women's Hospital, Boston, MA, USA); Jonathan Rosand, MD, MSc (Program in Medical and Population Genetics, Broad Institute of MIT and Harvard, Cambridge MA, USA; Department of Neurology and Center for Human Genetic Research, Massachusetts General Hospital, Boston, MA, USA; Department of Neurology, Harvard Medical School, Boston, MA, USA);

Peter M. Rothwell, FMedSci (Stroke Prevention Research Unit, Nuffield Department of Clinical Neurosciences, University of Oxford, John Radcliffe Hospital, Oxford, UK);
Tatjana Rundek, MD, PhD, FANA (Department of Neurology, Miller School of Medicine, University of Miami, Miami, FL, USA); Ralph L. Sacco, MD, MS, FAHA, FAAN, FANA (Department of Neurology, Miller School of Medicine, University of Miami, Miami, FL, USA); Reinhold Schmidt, MD (Department of Neurology, Clinical Division of Neurogeriatrics, Medical University Graz, Graz, Austria); Pankaj Sharma, MD, PhD, FRCP (Institute of Cardiovascular Research, Royal Holloway University of London (ICR2UL), Egham, UK; St Peter's and Ashford Hospitals, UK); Agnieszka Slowik, MD, PhD (Department of Neurology, Jagiellonian University Medical College, Krakow, Poland); Cathie LM Sudlow, DPhil, FRCP(E) (Centre for Clinical Brain Sciences & Institute of Genomic and Molecular Medicine, University of Edinburgh, UK); Vincent Thijs, MD, PhD (KU Leuven - University of Leuven, Department of Neurosciences, Experimental Neurology and Leuven Research Institute for Neuroscience and Disease (LIND), Leuven, Belgium; VIB, Vesalius Research Center, Laboratory of Neurobiology, Leuven, Belgium;

University Hospitals Leuven, Department of Neurology, Leuven, Belgium); Sylvia Wassertheil-Smoller, PhD (Department of Epidemiology and Population Health, Albert Einstein College of Medicine, Bronx, NY, USA);
Daniel Woo, MD, MS (University of Cincinnati College of Medicine, Cincinnati, OH, USA); Bradford B. Worrall, MD, MSc (Departments of Neurology and Public Health Sciences, University of Virginia, Charlottesville, VA, USA)

PIs of Control Studies:
Rebecca D. Jackson, MD (Division of Endocrinology, Diabetes and Metabolism, Department of Internal Medicine and the Center for Clinical and Translational Science, The Ohio State University, Columbus, OH);
Martina Müller-Nurasyid, PhD (Institute of Genetic Epidemiology, Helmholtz Zentrum München – Germany; Research Center for Environmental Health, Neuherberg, Germany; Department of Medicine I, Ludwig-Maximilians-University Munich, Munich, Germany; DZHK (German Centre for Cardiovascular Research), partner site Munich; Heart Alliance, Munich, Germany);
Mike A. Nalls, PhD (Laboratory of Neurogenetics, National Institute on Aging, National Institutes of Health, Bethesda, MD, USA);
Marta Ribasés, PhD, BSc (Psychiatric Genetics Unit, Group of Psychiatry, Mental Health and Addictions, Vall d'Hebron Research Institute (VHIR), Universitat Autònoma de Barcelona, Barcelona, Spain; Department of Psychiatry, Hospital Universitari Vall d'Hebron, Barcelona, Spain; Biomedical Network Research Centre on Mental Health (CIBERSAM), Barcelona, Spain); David R. Weir, PhD (Survey Research Center, University of Michigan, Ann Arbor, MI, USA)

Data Management:
Patrick F. McArdle, PhD (chair; Department of Medicine and Program for Personalized and Genomic Medicine, University of Maryland School of Medicine, Baltimore, MD);
Tushar Dave, MS (Department of Medicine and Program for Personalized and Genomic Medicine, University of Maryland School of Medicine, Baltimore, MD, USA)

Analysis:
Braxton D. Mitchell, PhD, MPH (chair; Division of Endocrinology, Diabetes and Nutrition, University of Maryland School of Medicine, Baltimore, MD, USA; Geriatric Research and Education Clinical Center, Veterans Administration Medical Center, Baltimore, MD, USA); Yu-Ching Cheng, PhD (Department of Medicine, University of Maryland School of Medicine, Baltimore, MD, USA);
Paul I.W. de Bakker, PhD (Department of Medical Genetics, University Medical Center Utrecht, Utrecht, The Netherlands; Department of Epidemiology, University Medical Center Utrecht, Utrecht, The Netherlands);

Myriam Fornage, PhD (Institute of Molecular Medicine, University of Texas Health Science Center at Houston, Houston, TX, USA);
Cathy C. Laurie, PhD (Department of Biostatistics, University of Washington, Seattle, WA, USA);

Ani Manichaikul, PhD (Center for Public Health Genomics, Biostatistics Section, Department for Public Health Sciences, University of Virginia, Charlottesville, VA, USA);
Jeffrey R. O'Connell, DPhil (Division of Endocrinology, Diabetes and Nutrition, University of Maryland School of Medicine, Baltimore, MD, USA); Sara L. Pulit, BA (Department of Medical Genetics, Institute for Molecular Medicine, University Medical Center Utrecht, Utrecht, The Netherlands);
Stephen S. Rich, PhD (Center for Public Health Genomics, University of Virginia, Charlottesville, VA, USA); Quenna Wong, MS (Department of Biostatistics, University of Washington, Seattle, WA, USA);
Huichun Xu, MD, PhD (Department of Medicine, University of Maryland School of Medicine, Baltimore, MD, USA)

Phenotype:
James F. Meschia, MD (co-chair; Department of Neurology, Mayo Clinic, Jacksonville, FL, USA);
Bradford B. Worrall, MD, MSc (co-chair; Departments of Neurology and Public Health Sciences,

University of Virginia, Charlottesville, VA, USA);
Hakan Ay, MD (AA Martinos Center for Biomedical Imaging, Department of Radiology, Massachusetts General Hospital, Harvard Medical School, Boston, MA, USA; Stroke Service, Department of Neurology, Massachusetts General Hospital, Harvard Medical School, Boston, MA, USA);
Robert D. Brown Jr., MD, MPH (Department of Neurology, Mayo Clinic, Rochester, MN, USA)

Imaging:
Jonathan Rosand, MD, MSc (chair; Program in Medical and Population Genetics, Broad Institute of MIT and Harvard, Cambridge MA, USA; Department of Neurology and Center for Human Genetic Research, Massachusetts General Hospital, Boston, MA, USA; Department of Neurology, Harvard Medical School, Boston, MA, USA);
Natalia S. Rost, MD, MPH (Stroke Division, Department of Neurology, Massachusetts General Hospital, Harvard Medical School, Boston, MA 02114);
Ona Wu, PhD (Athinoula A. Martinos Center for Biomedical Imaging, Department of Radiology, Massachusetts General Hospital, Charlestown, MA, USA; Department of Radiology, Harvard Medical School, Boston, MA, USA)

Publication:
Kathryn Rexrode, MD, MPH (chair Brigham and Women's Hospital, Boston, MA, USA); Tatjana Rundek, MD, PhD, FANA (prior chair; Department of Neurology, Miller School of Medicine, University of Miami, Miami, FL, USA);
Agnieszka Slowik, MD, PhD (prior co-chair; Department of Neurology, Jagiellonian University Medical College, Krakow, Poland); Hakan Ay, MD (AA Martinos Center for Biomedical Imaging, Department of Radiology, Massachusetts General Hospital, Harvard Medical School, Boston, MA, USA; Stroke Service, Department of Neurology, Massachusetts General Hospital, Harvard Medical School, Boston, MA, USA);

Oscar R. Benavente, MD, FRCP (Department of Neurology, University of British Columbia, Vancouver, British Columbia, Canada); Steve Bevan, PhD (Department of Clinical Neurosciences, University of Cambridge, Cambridge, UK); Katrina Gwinn, MD (National Institute of Neurological Disorders and Stroke, National Institutes of Health, Bethesda, MD, USA);
Steven J. Kittner, MD, MPH (chair; Department of Neurology, University of Maryland School of Medicine and Veterans Affairs Maryland Health Care System, Baltimore, MD, USA); Jin-Moo Lee, MD, PhD (Stroke Center, Department of Neurology, Washington University School of

Medicine, St. Louis, MO, USA);
Patrick F. McArdle, PhD (Department of Medicine and Program for Personalized and Genomic Medicine, University of Maryland School of Medicine, Baltimore, MD);
James F. Meschia, MD (Department of Neurology, Mayo Clinic, Jacksonville, FL, USA); Braxton D. Mitchell, PhD, MPH (Division of Endocrinology, Diabetes and Nutrition, University of Maryland School of Medicine, Baltimore, MD, USA; Geriatric Research and Education Clinical Center, Veterans Administration Medical Center, Baltimore, MD, USA); Jonathan Rosand, MD, MSc (Program in Medical and Population Genetics, Broad Institute of MIT and Harvard, Cambridge MA, USA; Department of Neurology and Center for Human Genetic Research,
Massachusetts General Hospital, Boston, MA, USA; Department of Neurology, Harvard Medical School, Boston, MA, USA);
Sylvia Wasssertheil-Smoller, PhD (Department of Epidemiology and Population Health, Albert Einstein College of Medicine, Bronx, NY, USA);
Daniel Woo, MD, MS (University of Cincinnati College of Medicine, Cincinnati, OH, USA); Bradford B. Worrall, MD, MSc (Departments of Neurology and Public Health Sciences, University of Virginia, Charlottesville, VA, USA)

CIDR:
Kimberly F. Doheny, PhD (Center for Inherited Disease Research, Institute of Genetic Medicine, Johns Hopkins School of Medicine, Baltimore, MD)

NINDS staff:
Roderick Corriveau, PhD (National Institutes of Health, Bethesda, MD, USA);
Katrina Gwinn, MD (National Institute of Neurological Disorders and Stroke, National Institutes of Health, Bethesda, MD, USA)

Membership of The International Stroke Genetics Consortium

Full Name	Country	Institution	Department	City
Joan Montaner	Spain	Universitat Autònoma de Barcelona, Vall d'Hebron Institute of Research	Neurovascular Research Laboratory	Barcelona
Allard Hauer	Netherlands	University Medical Center Utrecht, Brain Center Rudolf Magnus	Department of Neurology and Neurosurgery	Utrecht
Alessandro Biffi	USA	Massachusetts General Hospital	Division of Behavioral Neurology, Department of Neurology	Boston, MA
Adam DeHavenon	USA	University of Utah	Department of Neurology	Salt Lake City, UT
Alessandra Granata	UK	University of Cambridge	Department of Clinical Neurosciences	Cambridge
Anne-Katrin Giese	USA	Massachusetts General Hospital, Harvard Medical School	Department of Neurology	Boston, MA
Alessandro Pezzini	Italy	University of Brescia, Neurology Clinic	Department of Clinical and Experimental Sciences	Brescia
Alexis Simpkins	USA	University of Florida	Department of Neurology	Gainesville, FL
Andreea Ilinca	Sweden	Lund University	Neurology	Lund
Aniket Mishra	France	INSERM U1219 Bordeaux Population Health Research Center, Bordeaux, France		
anna bersano	Italy	IRCCS Foundation C. Besta Neurological Institute	Cerebrovascular Unit	Milan
Anna Drazyk	UK	University of Cambridge	Department of Clinical Neurosciences	Cambridge
Annie Pedersen	Sweden	Sahlgrenska Academy at University of Gothenburg	Department of Pathology and Genetics	Gothenburg
Antonio Arauz	Mexico	Instituto Nacional de Neurologia y Neurocirugia, Manuel Velasco Suarez	Stroke Clinic	Mexico City
Alex Reiner	USA	University of Washington	Department of Epidemiology	Seattle, WA
Arne Lindgren	Sweden	Lund University	Department of Clinical Sciences Lund, Neurology	Lund
Andrew Southerland	USA	University of Virginia	Departments of Neurology and Public Health Sciences	Charlottesville, VA
Alexis N. Simpkins	USA			
Jose Florez	USA	Massachusetts General Hospital	Center for Human Genetic Research, MGH	Boston, MA

Bradford Worrall	USA	University of Virginia School of Medicine	Departments of Neurology and Public Health Sciences	Charlottesvill e, VA
Silvia Bione	Italy	Institute of Molecular Genetics-National Research Council	Computational Biology Unit	Pavia
Bjorn Hansen	Sweden	Lund University	Department of Clinical Sciences Lund, Neurology	Lund
Braxton Mitchell	USA	University of Maryland School of Medicine	Department of Medicine	Baltimore, MD
Bo Norrving	Sweden	Lund University	Sahlgrenska Academy at University of Gothenburg	Lund
Bernard Chan	Singapore	National University Hospital		
Robert Brown	USA	Mayo Clinic	Department of Neurology	Rochester, MN
Tan Wei Liang Byorn	Singapore	Nanyang Technological University	Lee Kong Chian School of Medicine	Singapore
caspar Grond-Ginsbach	Germany	Heidelberg University Hospital	Department of Neurology	Heidelberg
Catherine Sudlow	UK	University of Edinburgh	Department of Neurology	Edinburgh
Cathryn Lewis	UK	King's College London	Department of Medical and Molecular Genetics	London
Caty Carrera	Spain	Universitat Autònoma de Barcelona, Vall d'Hebrón Hospital	Neurovascular Research Laboratory, Vall d'Hebron Institut of Research, Neurology and Medicine Departments	
Carlos Cruchaga	USA	Washington University School of Medicine	Department of Psychiatry,The Hope Center Program on Protein Aggregation and Neurodegeneration (HPAN)	St. Louis, MO
Chris Anderson	USA	Massachusetts General Hospital (MGH)	Center for Genomic Medicine	Boston, MA
Chaur-Jong Hu	Taiwan			
Chloé Sarnowski	USA	Boston University	Department of Biostatistics	Boston
Chris Riley	USA	American Heart Association	Institute for Precision Cardiovascular Medicine Stroke Genetics	
Christina Jern	Sweden	The Sahlgrenska Academy at University of Gothenburg, Institute of Biomedicine	Department of Pathology and Genetics	Gothenburg
Christopher Levi	Australia	John Hunter Hospital, Hunter Medical Research Institute and University of Newcastle		Newcastle
Christina Kourkoulis	USA	Massachusetts General Hospital	Department of Neurology	Boston, MA

Carl Langefeld	USA	Wake Forest School of Medicine	Center for Public Health Genomics and Department of Biostatistical Sciences	Winston-Salem, NC
Chaeyoung Lee	Korea	Soongsil University	Department of Bioinformatics and Life Science	Seoul
Caitrin Wheeler McDonough	USA	University of Florida	Department of Pharmacotherapy and Translational Research	Gainesville, FL
Chia-Ling Phuah	USA	Center for Human Genetic Research, Massachusetts General Hospital (MGH)		Boston, MA
Christopher Chen	Singapore	National University of Singapore	Department of Pharmacology	Singapore
Carolina Soriano	Spain	IMIM-Hospital del Mar	Department of Neurology, Neurovascular Research Group	Barcelona
Dina Vojinovic	Netherlands	Erasmus MC, University Medical Center Rotterdam	Department of Epidemiology	Rotterdam
Daniel Strbian	Finland	Helsinki University Hospital	Department of Neurology	Helsinki
Daniel Woo	USA	University of Cincinnati College of Medicine		Cincinnati, OH
David Tregouet	France	Sorbonne Universités, UPMC Univ. Paris 06, INSERM, UMR_S 1166	Team Genomics and Pathophysiology of Cardiovascular Diseases	Paris
David Liebeskind	USA	University of California Los Angeles	Department of Neurology	Los Angeles, CA
Daniel Chasman	USA	Brigham and Women's Hospital	Division of Preventive Medicine	Boston, MA
Raj Dhar	USA	Washington University School of Medicine	Department of Neurology	St. Louis, MO
Dipender Gill	UK	Imperial College London	Centre for Pharmacology and Therapeutics	London
Debra Owens	USA	University of Virginia Children's Hospital	Newborn Intensive Care Units	Charlottesville, VA
Amanda Donatti	Brazil	University of Campinas, UNICAMP	School of Medical Sciences, FCM	São Paulo
Dorothea Pfeiffer	Germany	Hiedelberg University Hospital	Department of Neurology	Heidelberg
Kameshwar Prasad	India	All India Institute of Medical Sciences (AIIMS)	Department of Neurology and Stroke Unit;<br>and Director of Clinical Epidemiology,	New Delhi
Thomas Leung	Hong-Kong	The Chinese University of Hong Kong	Division of Neurology, Department of Medicine and Therapeutics	Hong-Kong
Eva Giralt	Spain	Parc de Salut Mar		Barcelona
Eric Harshfield	UK			

Elsa Valdés Márquez	UK	University of Oxford	CTSU-Clinical Trial Service Unit and Epidemiological Studies Unit	Oxford
Erik Lorentzen	Sweden	University of Gothenburg	Bioinformatics Core Facility	Gothenburg
Farrah Mateen	USA	Massachusetts General Hospital	Department of Neurology	Boston, MA
Foad Abd-Allah	Egypt	Cairo University	Department of Neurology and Stroke Medicine	Cairo
Frank-Erik de Leeuw	Netherlands	Radboud University Medical Center	Department of Neurology	Nijmegen
Gabriel Rinkel	Netherlands	UMC Utrecht, Brain Center Rudolf Magnus	Department of Neurology and Neurosurgery	Utrecht
Ganesh Chauhan	India	Indian Institute of Science	Centre for Brain Research	Bangalore
Matthew Walters	UK	University of Glasgow	School of Medicine, Dentistry and Nursing	Glasgow
Guido Falcone	USA	Yale University School of Medicine	Department of Neurology	New Haven, CT
Giorgio Boncoraglio	Italy	Fondazione IRCCS Istituto Neurologico "Carlo Besta"	Department of Cerebrovascular Diseases	Milan
Hideaki Suzuki	Japan			
Hanne Sallinen	Finland	Helsinki University Hospital	Department of Neurology	Helsinki
Hakan Ay	USA	Massachusetts General Hospital	Department of Neurology	Boston, MA
Laura Heitsch	USA	Washington University School of Medicine	Department of Neurology, Radiology, and Biomedical Engineering	St. Louis, MO
Hyacinth I Hyacinth	USA	Emory University School of Medicine	Aflac Cancer and Blood Disorder Center, Department of Pediatrics	Atlanta, GA
Hirofumi Nakatomi	Japan			
Atsushi Hozawa	Japan			
Hugh Markus	UK	University of Cambridge	Stroke Research Group, Division of Clinical Neurosciences,	Cambridge
Chung Hsu	Taiwan	China Medical University		
Hugo Javier Aparicio	USA	Boston University School of Medicine	Department of Neurology	Boston, MA
Huichun Xu	USA	School of Medicine, University of Maryland	Medicine department	Baltimore
Iain Turnbull	UK	University of Oxford	Clinical Trial Service Unit and Epidemiological Studies Unit, Nuffield Department of Population Health	Oxford

Laura Garcia Ibenez	USA	University of Columbia	Institute for Cancer Genetics	New York
Iscia Cendes	Brazil	Universidade Estadual de Campinas, Faculdade de Ciências Médicas	Molecular Genetics Laboratory	Campinas
Masafumi Ihara	Japan	National Cerebral and Cardiovascular Center	Department of Neurology	Osaka
I-Hui Lee	Taiwan			
Ynte Ruigrok	Netherlands	University Medical Center Utrecht, Brain Center Rudolf Magnus	Department of Neurology and Neurosurgery	Utrecht
Ioana Cotlatciuc	UK	University of London	Institute of Cardiovascular Research Royal Holloway	London
Iona Millwood	UK	University of Oxford	Nuttfield Department of Population Health	Oxford
Ryan Irvin	USA	University of Alabama at Birmingham	Department of Epidemiology	Birmingham, AL
Israel Fernandez-Cadenas	Spain	Universitat Autònoma de Barcelona, Vall d'Hebrón Hospital	Neurovascular Research Laboratory, Vall d'Hebron Institut of Research, Neurology and Medicine Departments	Barcelona
Juan José Martín	Argentina	Sanatorio Allende	Department of Neurology	Cordoba
Jane Maguire	Australia	University of Technology Sydney	Faculty of Health	Ultimo
Janika Korv	Estonia	University of Tartu	Department of Neurology and Neurosurgery	Tartu
John Cole	USA	University of Maryland School of Medicine and Baltimore VAMC	Department of Neurology	Baltimore, MD
Jill Stewart	USA	University of South Carolina	Department of Exercise Science	Columbia, SC
John Danesh	UK	University of Cambridge	MRC/BHF Cardiovascular Epidemiology Unit, Department of Public Health and Primary Care,	Cambridge
James E Faber	USA	University of North Carolina	Cell Biology and Physiology	Chapel Hill
Jemma Hopewell	UK	University of Oxford	Clinical Trial Service Unit and Epidemiological Studies Unit, Nuffield Department of Population Health	Oxford
Jennifer Majersik	USA	University of Utah	Neurology	Salt Lake City, UT
Jim Koenig	USA	NINDS	Division of Neuroscience, Stroke Program in the Neural Environment	Bethesda, MA
Jordi Jimenez-Conde	Spain	IMIM - Hospital del Mar	Neurovascular Research Group (NEUVAS). Neurology Department.	Barcelona

Joanna Howson	UK	University of Cambridge	MRC/BHF Cardiovascular Epidemiology Unit, Department of Public Health and Primary Care	Cambridge
Joanna Wardlaw	UK	University of Edinburgh	Neuroimaging Sciences	Edinburgh
Johan Wasselius	Sweden	Lund University	Department of Clinical Sciences Malmö	Malmö
John Attia	Australia	University of Newcastle	School of Medicine and Public Health	New Lambton
Joseph Broderick	USA	University of Cincinnati	Gardner Neuroscience Institute, Comprehensive Stroke Center	Cincinnati, OH
Josh Bis	USA	University of Washington	Cardiovascular Health Research Unit, Department of Medicine	Seattle, WA
Jonathan Rosand	USA	Massachusetts General Hospital	Department of Neurology	Boston, MA
Joohon Sung				
Jer-Yuarn Wu	Taiwan	Institute of Biomedical Sciences, Academia Sinica		Taipei
Karin Klijn	Netherlands	Radboud University Medical Centre, Donders Institute for Brain, Cognition and Behaviour	Department of Neurology	Nijmegen
Katarina Jood	Sweden	Institute of Neuroscience and Physiology, Sahlgrenska Academy at University of Gothenburg	Department of Clinical Neuroscience	Gothenburg
Katrina Gwinn	USA	National Institutes of Health	National Institute of Neurological Disorders and Stroke	Bethesda, MD
Katherine Crawford	USA	Massachusetts General Hospital	Center for Genomic Medicine	Boston, MA
Kevin Sheth	USA	Yale School of Medicine	Department of Neurology	New Haven, CT
Gyeong-Moon Kim	Korea	Samsung Medical Center, Sungkyunkwan University School of Medicine	Department of Neurology	Seoul
Helen Kim	USA	Université de Californie	Institute for Human Genetics, The Center for Cerebrovascular Research (CCR)	San Francisco
Kathleen Donahue	USA	Massachusetts General Hospital	J. Philip Kistler Stroke Research Center	Boston, MA

Jan Konrad	USA	Washington University, School of Medicine	Department of Psychiatry,The Hope Center Program on Protein Aggregation and Neurodegeneration (HPAN)	St. Louis, MO
Kathryn Rexrode	USA	Brigham and Women's Hospital	Department of Medicine	Boston
Kristiina Rannikmae	UK	University of Edinburgh	Department of Neurology	Edinburgh
Kwang-Yeol Park	Korea			Seoul, South Korea
Kazuo Washida	Japan			
Martin Stenman	Sweden	Lund University	Geriatric Medicine	Lund
Marcos Christiano Lange	Brazil	Federal University of Parana	Clinical Hospital	
Jin-Moo Lee	USA	Washington University School of Medicine	Department of Neurology, Radiology, and Biomedical Engineering	St. Louis, MO
Leslie Lange	USA	University of Colorado Denver - Anschutz Medical Campus		Denver, CO
Saskia Lesnik Oberstein	Netherlands	Leiden University Medical Center	Department of Clinical Genetics	Leiden
Liisa Tomppo	Finland	Helsinki University Hospital	Department of Neurology	Helsinki
Guiyou Liu	China	Harbin Institue of Technology	School of Life Science and Technology	Harbin
Elizabeth Holliday	Australia	Hunter Medical Research Institute	Public Health Stream	New Lambton
Loes Rutten-Jacobs	UK	University of Cambridge, Division of Clinical Neurosciences	Stroke Research Group	Cambdrige
Lukas Holmegaard	Sweden	Institute of Neuroscience and Physiology, Sahlgrenska Academy at University of Gothenburg	Department of Clinical Neuroscience	Gothenburg
Mark Bakker	Netherlands	University Medical Center Utrecht		Utrecht
Maja Olsson	Sweden	Sahlgrenska Academy at University of Gothenburg	Department of Medical and Clinical Genetics, Institute of Biomedicine	Gothenburg
Michelangelo Mancuso	Italy	University of Pisa	Department of Neurology	Pisa
Martin Dichgans	Germany	Ludwig-Maximilians-University, Klinikum der Universität München	Institute for Stroke and Dementia Research	Munich
Martin Söderholm	Sweden	University of Gothenburg	Bioinformatics Core Facility	Gothenburg

Martina Olsson	Sweden	University of Gothenburg		Gothenburg
Matthew Alexander	USA	University of Utah School of Medicine	Radiology and Imaging Sciences	Salt Lake City, UT
Mattias Drake	Sweden	Lund University	Diagnostic Radiology	Lund
Mayowa Owolabi	Nigeria	University College Hospital	Department of Neurology	Ibadan
James Meschia	USA	Mayo Clinic	Department of Neurology	Jacksonville
Mike Chong	Canada	McMaster University	Population Health Research Institute	Hamilton
Michael James	USA	Duke University	Brain Injury Translational Research Center	Durham
Michiaki Kubo	Japan	RIKEN Center for Integrative Medical Sciences		Yokohama
Miguel Barboza	Costa Rica	Hospital Rafael A. Calderon Guardia	Department of Neurology	San Jose
Michele Sale	USA	University of Virginia	Center for Public Health Genomics	Charlottesvill e, VA
Markus Schirmer	USA	Massachusetts General Hospital and Harvard Medical School	Department of Neurology, J. Philip Kistler Stroke Research Centre	Boston
Mitchell Elkind	USA	Columbia University	Department of Neurology	New York, NY
Magdy Selim	USA	Harvard Medical School	Beth Israel Deaconess Medical Center	Boston
Matthew Traylor	UK	University of Cambridge	Stroke Research Group, Division of Clinical Neurosciences	Cambridge
Murali Sargurupremraj	France	University of Bordeaux	INSERM U1219 Bordeaux Population Health Research Center	Bordeaux
Myriam Fornage	USA	University of Texas Health Science Center at Houston	Brown Foundation Institute of Molecular Medicine // Human Genetics Center	Houston, TX
Nuria Torres	Spain	Hospital Mútua de Terrassa, Fundació Docència i Recerca Mútua Terrassa	Stroke Pharmacogenomics and Genetics	Terrassa
Jong-Won Chung	USA	Samsung Medical Center, Sungkyunkwan University School of Medicine	Department of Neurology	Seoul
Toshiharu Ninomiya	Japan	Kyushu University, Graduate School of Medical Sciences	Department of Epidemiology and Public Health	Fukuoka
Natalia Rost	USA	Department of Neurology, Massachusetts General Hospital, Harvard Medical School, Boston, MA, USA		Boston, MA

Olle Melander	sweden	Lund University	Department of Clinical Sciences	Lund
Ona Wu	USA	Massachusetts General Hospital and Harvard Medical School	Department of Neurology, JPK Stroke Research Center	Boston, MA
Pankaj Sharma	UK	Royal Holloway University of London	Institute of Cardiovascular Research	London
Eugenio Parati	Italy	Fondazione IRCCS Istituto Neurologico "C. Besta", Cerebrovascular Disease Unit	Laboratory of Cellular Neurobiology	Milan
Guillaume Pare	Canada	McMaster University	Population Health Research Institute	Hamilton
Joanna Pera	Poland	Jagiellonian University Medical College	Department of Neurology	Krakow
Petrea Frid	Sweden	Skåne University Hospital, Lund university	Critical Care Neurology	Malmö
Paulo Franca	Brazil	Universidade da Região de Joinville – Univille	Department of Medicine	Joinville
Phil Clatworthy	UK	University of Bristol	Stroke Association	Bristol, UK
Philippe Bijlenga	Switzerland	Geneva University Hospital	Departement des neurosciences cliniques, Clinique de neurochirurgie	Geneva
Patrick McArdle	USA	University of Maryland School of Medicine	Division of Endocrinology, Diabetes and Nutrition	Baltimore, MD
Patricia Musolino	USA	Massachusetts General Hospital		
Paul Nyquist	USA	Johns Hopkins Medicine	Department of Anesthesiology and Critical Care Medicine	Baltimore, MD
Polina Golland	USA	Massachusetts Institute of Technology	The Computer Science and Artificial Intelligence Laboratory	Cambridge, MA
Bruce Psaty	USA	University of Washington	Cardiovascular Health Research Unit, Department of Medicine	Seattle, WA
Qiong Yang	USA	Boston University School of Public Health		Boston
Reecha Sofat	UK	University College London	Metabolism & Experi Therapeutics, Div of Medicine, Faculty of Medical Sciences	London
Rainer Malik	Germany	Ludwig-Maximilians-University, Klinikum der Universität München	Institute for Stroke and Dementia Research	Munich

Ramin Zand	USA	Geisinger Health System	Department of Neuroscience	Danville
Richard Choy	Hong-Kong	The Chinese University of Hong Kong	Department of Obstetrics and Gynecology	Hong-Kong
Riina Vibo	Estonia	University of Tartu	Department of Neurology and Neurosurgery	Tartu
Roberta Carriero	Italy	Humanitas Clinical Institute	Inflammation and immunology laboratory	Pavia
Robin Lemmens	Belgium	KU Leuven – University of Leuven	Department of Neurosciences, Experimental Neurology	Leuven
Robin Walters	UK	University of Oxford	Clinical Trial Service Unit and Epidemiological Studies Unit, Nuffield Department of Population Health	Oxford
romain bourcier	France	University Hospital of Nantes	Department of Neuroradiology	Nantes
Owen A. Ross	USA	Mayo Clinic	Department of Medical Genetics and Neuroscience	Jacksonville
Ralph Sacco	USA	Miller School of Medicine, University of Miami	Department of Neurology	Miami, FL
Rufus Akinyemi	Nigeria	University of Ibadan, College of Medicine	Institute for Advanced Medical Research and Training	Ibadan
Rustam Al-Shahi Salman	UK	University of Edinburgh	Department of Clinical Neurology	Edinburgh
Sara L. Pulit	Netherlands	University Medical Center Utrecht, Brain Center Rudolf Magnus	Department of Neurology	Utrecht
Sander W. van der Laan	Netherlands	University Medical Center Utrecht, Division of Heart and Lungs	Laboratory of Experimental Cardiology	Utrecht
Danish Saleheen	USA	Perelman School of Medicine, University of Pennsylvania	Department of Genetics	Pennsylvania, PA
Sandra Olsson	Sweden	University of Gothenburg		Gothenburg
Sandro Marini	USA	Harvard Medical School	Center for Human Genetic Research	Boston, MA
Steve Bevan	UK	School of Life Science, University of Lincoln		Lincoln
Kristina Schlicht	Germany	Kiel University	Institut für Medizinische Informatik und Statistik	Kiel
Steve Cramer	USA	University of California Irvine	Neurology, Anatomy & Neurobiology, and Physical Medicine & Rehabilitation	Irvine
Stephanie Debette	France	INSERM U1219 Bordeaux Population Health Research Center, Bordeaux, France		
Shahbaz Zamani	Australia	University of Newcastle		Newcastle

Steven Kittner	USA	University of Maryland School of Medicine and Baltimore VAMC, MD, USA	Department of Neurology	Baltimore, MD
Soo Ji Lee	South Korea	Seoul National University		
Agnieszka Slowik	Poland	Jagiellonian University Medical College	Department of Neurology	Krakow
Steven Lubitz	USA	Massachusetts General Hospital, Harvard Medical School	Department of Cardiology	Boston, MA
Stacey Quintero Wolfe	USA	Wake Forest Baptist Health		Winston-Salem, NC
Stephen Rich	USA	University of Virginia School of Medicine	Center for Public Health Genomics	Charlottesville, VA
Suo Yue	China	Beijing Tiantan Hospital	Department of Neurology	Beijing
Sudha Seshadri	USA	Boston University School of Medicine	Department of Neurology	Boston, MA
Tai-Ming Ko	Taiwan	Institute of Biomedical Sciences, Academia Sinica		Taipei
Eng King Tan	Singapore	Singapore General Hospital, National Neuroscience Institute	Department of Neurology	Singapore
Rhea Tan	England	University of Cambridge	Department of Clinical Neurosciences	Cambridge
Tara Stanne	Sweden	University of Gothenburg		Gothenburg
Tushar Dave	USA	University of Maryland School of Medicine	Bioinformatics	Baltimore, MD
Tsong-Hai Lee	Taiwan	Linkou Chang Gung Memorial Hospital	Neurology department and Stroke center	Taipei
Tom van Agtmael	UK	University of Glasgow	Insittute of Cardiovascular and Medical Sciences	Glasgow
Tatjana Rundek	USA	Miller School of Medicine, University of Miami	Department of Neurology	Miami, FL
Turgut Tatlisumak	Sweden	Sahlgrenska Academy at University of Gothenburg, Institute of Neuroscience and Physiology	Department of Clinical Neurosciences/Neurology	Gothenburg
Umme Lena	USA	Massachusetts General Hospital		South Hadley, MA
Vincent Thijs	Australia	Florey Institute of Neuroscience and Mental Health	Stroke Division	Heidelberg
Katie Vo	USA	Washington University School of Medicine		

Wagner Avelar	Brazil	University of Campinas, UNICAMP	Department of Neurology	São Paulo
Will Longstreth	USA	University of Washington	Department of Epidemiology	Seattle
Keat Wei Loo	Malaysia	Tunku Abdul Rahman University, UTAR	Department of Biological Science	Kampar Perak
Yoichiro Kamatani	Japan	RIKEN Center for Integrative Medical Sciences	Laboratory for Statistical Analysis	Yokohama
Yukinori Okada	Japan	RIKEN Center for Integrative Medical Sciences	Laboratory for Statistical Analysis	Yokohama
Jaeyoon Chung	USA	Massachusetts General Hospital	Center for Genomic Medicine	Boston, MA
Patryk Kubiszewski	USA	Massachusetts General Hospital	Center for Genomic Medicine	Boston, MA
Christhunesa Christudass	India	Christian Medical College	Department of Neurochemistry	Vellore
Simone Uniken Venema	Netherlands	Utrecht University Medical School		Utrecht
Sanjith Aaron	India	Christian Medical College	Department of Neurochemistry	Vellore
Brady Gaynor	USA	University of Maryland School of Medicine	Division of Endocrinology, Diabetes and Nutrition	Baltimore, MD
Pei-Ying Chuang	USA	University of Maryland School of Medicine		Baltimore, MD
Ugur Erickson	USA	University of Maryland School of Medicine		Baltimore, MD
Kay Sin Tan	Malaysia	University of Malaya	Division of Neurology, Department of Medicine	Kuala Lumpur
Kathy Ryan	USA	University of Maryland Schoolof Medicine	Division of Endocrinology, Diabetes, and Nutrtion	Baltimore, MD
Zurab Nadareishvili	USA	George Washington University	Department of Neurology	Washington, DC
Elodie Persyn	UK	King's College London		London
Thomas Jaworek	USA	University of Maryland	Department of Medicine	Baltimore, MD
Eric Ho	USA	Lafayette College	Department of Biology	Easton, PA
David Spence	Canada	Robarts Research Institute, Western University	Department of Neurology and Clinical Pharmacology	London, Ontario
Katherine Kirkwood	USA	Mount Sinai		
Lingyan Chen	UK	University of Cambridge	Department of Public Health and Primary Care	Cambridge
Marco Nardin	USA	Massachusetts General Hospital	J. Philip Kistler Stroke Research Center	Boston, MA
Haley Lopez	USA	University of Maryland School of Medicine	Department of Neurology	Baltimore, MD

Cecilia Lagging	Sweden	Gotenburg University		Gothenburg
Kyle Walsh	USA	University of Cincinnati	Department of Emergency Medicine	Cincinnati, OH
Samantha Speroni	USA	Holy Cross		Worcester, MA
Chris Levi	Australia	University of Newcastle	Department of Neurology	Newcastle
Nicole Dueker	USA	Miller School of Medicine, University of Miami		Miami, FL
Graham Hamilton	UK	University of Glasgow	Research Institute of Molecular, Cell & Systems Biology, College of Medical, Veterinary & Life Sciences	Glasgow
Doris A. Taylor	USA	Texas Heart Insitute	Regenerative Medicine	Houston, TX
Susan Halloran Blanton	USA	University of Miami Miller School of Medicine	Human Genetics and Genomics	Miami, FL
Keith L. Keene	USA	East Carolina University		Greenville, NC
Nicole Davis Armstrong	USA	East Carolina University		Greenville, NC
Francesca Bosetti	USA		Neural Environment	
Nicole James	UK	Imperial College London		London
Lawrence Latour	USA	National Institute of Neurological Disorders and Stroke		Bethesda, MD
Hee-Joon Bae	Korea	Seoul National University College of medicine	Cerebrovascular Disease center	Seoul
Thomas "Nate" Person	USA	Geisinger Health System	Genomic Program	Danville, PA
Jeff O'Connell	USA	University of Maryland School of Medicine	Division of Endocrinology, Diabetes and Nutrition	Baltimore, MD
Monique Stins	USA	Johns Hopkins Medicine	Molecular Microbiology and Immunology	Baltimore, MD
Keon-Joo Lee	Korea	Seoul National University College of medicine	Department of neurology	Seoul
Matthew Pase	Australia	Florey Institute of Neuroscience and Mental Health		Melbourne
Robynne Braun	USA	University of Maryland Rehabilitation Institute	Neurology and Stroke Rehabilitaiton Unit	Baltimore, MD
Nicola Armstrong	Australia	Murdoch University	Mathematics and Statistics	Perth
Wendy Ziai	USA	Johns Hopkins University School of Medicine	Neurocritical Care	Baltimore, MD
Meiyun Wang	China	Henan Provincial People's Hospital	Department of Radiology	Henan Sheng

Xiaoyue Ma	China	Henan Provincial People's Hospital	Department of Radiology	Henan Sheng
Toni Pollin	USA	University of Maryland School of Medicine	Epidemiology and Public Health	Baltimore, MD
Steve Cramer	USA	University of California Irvine	Department Anatomy and Neurobiology	Irvine, CA
Steve Messe	USA	Universty of Pennsylvania	Department of Neurology	Philadelphia, PA
David Hasan	USA	University of Iowa	Department of Neurosurgery	Iowa City, IA
Brandon Hancock	USA	Massachusetts General Hospital		Boston, MA
Christoph Griessenauer	USA	Geisinger Commonwealth School of Medicine	Department of Neurosurgery	Scranton, PA
Zhang Li-Qun	USA	University of Maryland School of Medicine	Department of Orthopaedics	Baltimore, MD
Remco Jan Hack	Netherlands	Leiden University Medical Center	Department of Clinical Genetics	Leiden
Yan Guo	USA	Pacific Biosciences	Field Applications	Menlo Park, CA
Yu-Ching Cheng	USA	University of Maryland School of Medicine	Department of Neurology	Baltimore, MD
Mithilesh Siddu	USA	George Washington University	Department of Neurology	Washington, DC
Glen Jickling	Canada	University of Alberta	Department of Medicine, Division of Neurology	Alberta
Fahad S. Al-Ajlan	Saudi Arabia	King Faisal Specialist Hospital an Research Center	Department of Neurosciences	Riyadh
Satoru Miyawaki	Japan	The University of Tokyo	Department of Neurosurgery	Tokyo
Nita A Limdi	USA	University of Alabama at Birmingham	Department of Neurology	Birmingham, AL
Hiroki Hongo	Japan	The University of Tokyo	Department of Neurosurgery	Tokyo
Hong-Kyun Park	Korea	Inje University Ilsan Paik Hospital	Department of Neurology	Goyang
Ruchira Menka Jha	USA	University of Pittsburgh	Departments of Critical Care Medicine, Neurology, Neurological Surgery	Pittsburgh, PA
Jennifer E Huffman	USA	Boston VA Healthcare System	Center for Population Genomics	Boston, MA
Remco Jan Hack	Netherlands	Leiden University Medical Center	Department of Clinical Genetics	Leiden
Gido Gravesteijn	Netherlands	Leiden University Medical Center	Department of Clinical Genetics and Department of Human Genetics	Leiden
Zhengming Chen	UK	University of Oxford	Department of Population Health	Oxford
Robert Clarke	UK	University of Oxford	Department of Population Health	Oxford

Chih--Kuang Cheng	Taiwan	Chang-Gung Memorial Hospital	Department of Neurology	Taoyuan
Najibah Ali Galadanci	USA	University of Alabama at Birmingham		Birmingham, AL
Joanna von Berg	Netherlands	Universitair Medisch Centrum Utrecht	Division of Laboratories, Pharmacy, and Biomedical Genetics	Utrecht
Sali Farhan	USA	Massachusetts General Hospital	Center for Genomic Medicine	Boston, MA
Hye-Yeon Choi	USA	Weill Cornell Medicine	Englander institute of Precision Medicine	New York, NY
Marios Georgakis	Germany	Ludwig-Maximilians-University, Klinikum der Universität München	Institute for Stroke and Dementia Research	Munich
Marie-Gabrielle Duperron	France	Bordeaux University	Bordeaux Population Health	Bordeaux
Olivia Skrobot	UK	Bristol Medical School	Translational Health Sciences	Bristol
Rozan Attili	Germany	Medizinische Hochschule Hannover	Institut für Klinische Chemie	Hannover
Luanluan Sun	UK	University of Cambridge	Department of Public Health and Primary Care	Cambridge
Yasaman Saba	Austria	Medical University Graz	Division of Molecular Biology and Biochemistry	Graz
Stanley Zimba	Zambia	University Teaching Hospital in Lusaka	Department of Neurology	Lusaka
Shadi Yaghi	USA	Lifespan	Department of Neurology	Providence, RI
Chi-kwan Tsang	China	The Clinical Neuroscience Insititute of Jinan University	Department of Neuroscience	Guangzhou
Jae Guk Kim	Korea	Eulji University Daejeon Eulji Medical Center	Department of Neurology	Daejeon
Christoph Nowak	Sweden	Karolinska Institutet Care Sciences and Society	Department of Neurobiology	Huddinge
Dharambir Sanghera	USA	University of Oklahoma Health Sciences Center	Department of Pediatrics, Section of Genomics	Oklahoma City, OK
Karen Albright	USA	Upstate Medical Unversity	Department of Neurology	Syracuse, NY
Shanshan Sheehy	USA	Boston University School of Medicine	Department of Medicine	Boston, MA
